# Promzea: a pipeline for discovery of co-regulatory motifs in maize and other plant species and its application to the anthocyanin and phlobaphene biosynthetic pathways and the Maize Development Atlas

**DOI:** 10.1186/1471-2229-13-42

**Published:** 2013-03-15

**Authors:** Christophe Liseron-Monfils, Tim Lewis, Daniel Ashlock, Paul D McNicholas, François Fauteux, Martina Strömvik, Manish N Raizada

**Affiliations:** 1 Department of Plant Agriculture, University of Guelph, Guelph, ON N1G 2W1, Canada; 2 Department of Mathematics and Statistics, University of Guelph, Guelph, ON N1G 2W1, Canada; 3 Department of Plant Sciences, McGill University, Ste. Anne de Bellevue, QC H9X 3V9, Canada

**Keywords:** Promoter, *cis*-acting, Motif, Maize, Anthocyanin, Phlobaphene, Bioprospector, MEME, Weeder, C1, P

## Abstract

**Background:**

The discovery of genetic networks and *cis*-acting DNA motifs underlying their regulation is a major objective of transcriptome studies. The recent release of the maize genome (*Zea mays* L.) has facilitated *in silico* searches for regulatory motifs. Several algorithms exist to predict *cis*-acting elements, but none have been adapted for maize.

**Results:**

A benchmark data set was used to evaluate the accuracy of three motif discovery programs: BioProspector, Weeder and MEME. Analysis showed that each motif discovery tool had limited accuracy and appeared to retrieve a distinct set of motifs. Therefore, using the benchmark, statistical filters were optimized to reduce the false discovery ratio, and then remaining motifs from all programs were combined to improve motif prediction. These principles were integrated into a user-friendly pipeline for motif discovery in maize called Promzea, available at http://www.promzea.org and on the Discovery Environment of the iPlant Collaborative website. Promzea was subsequently expanded to include rice and Arabidopsis. Within Promzea, a user enters cDNA sequences or gene IDs; corresponding upstream sequences are retrieved from the maize genome. Predicted motifs are filtered, combined and ranked. Promzea searches the chosen plant genome for genes containing each candidate motif, providing the user with the gene list and corresponding gene annotations. Promzea was validated *in silico* using a benchmark data set: the Promzea pipeline showed a 22% increase in nucleotide sensitivity compared to the best standalone program tool, Weeder, with equivalent nucleotide specificity. Promzea was also validated by its ability to retrieve the experimentally defined binding sites of transcription factors that regulate the maize anthocyanin and phlobaphene biosynthetic pathways. Promzea predicted additional promoter motifs, and genome-wide motif searches by Promzea identified 127 non-anthocyanin/phlobaphene genes that each contained all five predicted promoter motifs in their promoters, perhaps uncovering a broader co-regulated gene network. Promzea was also tested against tissue-specific microarray data from maize.

**Conclusions:**

An online tool customized for promoter motif discovery in plants has been generated called Promzea. Promzea was validated *in silico* by its ability to retrieve benchmark motifs and experimentally defined motifs and was tested using tissue-specific microarray data. Promzea predicted broader networks of gene regulation associated with the historic anthocyanin and phlobaphene biosynthetic pathways. Promzea is a new bioinformatics tool for understanding transcriptional gene regulation in maize and has been expanded to include rice and Arabidopsis.

## Background

A key objective of global gene expression studies is the identification of transcription factors and their DNA binding sites responsible for co-expression of genes. DNA binding sites can be predicted *in silico* by searching regulatory regions of co-expressed genes for overrepresented motifs [1,2]. Recently, the genome sequence of maize (*Zea mays* L.) was released [3], facilitating searches for *cis*-acting motifs in one of the world’s most important crops. Useful motif discovery tools already exist for maize including Grassius [4] and PlantPAN [5], but they retrieve only known, experimentally defined motifs from databases such as PLACE [6] or PlantTFDB [7]. There remains a need for software that predicts *de novo* motifs from co-expressed genes in maize including from microarray data.

In general, two major types of algorithms exist to search co-regulated genes for *de novo* motifs. The first approach, consensus searching, consists of searching sets of genes for similar sequences. This consensus method limits motif searches to 12 bases in length (because of the calculation time necessary to search longer motifs) and allows for a few substitutions [8]. Weeder [8] is a widely used program that applies consensus-based sampling. The second type of search algorithm is probabilistic and uses a position weight matrix (PWM) to define a motif [9]. In the PWM, the probability of occurrence of each of the four possible nucleotides is calculated for every position within a predicted motif. Motif PWMs are first identified by scanning regulatory sequences for similar motifs. Predicted motifs are reported if the probability of the motif occurrence is statistically non-random compared to the background. Widely used software programs that apply a probabilistic algorithm are BioProspector [10] and MEME (Multiple Expectation-maximization for Motif Elicitation) [11]. These programs employ different statistical approaches. BioProspector uses Gibbs sampling [12] which randomly picks subsequences of a defined length and iteratively searches within input promoters until a high probability match is found, defined as having PWM values that are significantly different from the input background sequences. By contrast, MEME divides sequences into sub-segments, and all sub-segments are systematically processed as a possible motif. The probability that each sub-segment occurs non-randomly within input promoters is calculated based on its PWM values (Expectation, E) which is then refined based on the probability of occurrence of each nucleotide at each position within the sub-segment (Maximization, M). The sub-segment with the highest probability after EM is chosen and modified by iterating the EM algorithm until a candidate motif cannot be improved [11].

The various motif discovery programs have significant limitations. For example, one limit of Gibbs sampling and hence BioProspector [10], is that different motifs are often obtained at each run. In contrast, MEME predictions are consistent [11]. The main problem with all the current motif discovery programs is their low accuracy. The best motif discovery program thus far was shown to be only 17.4% accurate, in *E.coli*, with many known motifs being missed [13]. In order to overcome the problem of low prediction accuracy, motif discovery programs have been combined to increase their effectiveness, creating what has been termed an ensemble algorithm [13]. One of the first ensemble algorithms was the BEST program [14] which combined the advantages of three motif discovery programs. Other ensemble tools also exist to define *de novo* motifs in Arabidopsis and rice, for example MotifVoter [15] that clusters the best motifs from 10 motif discovery tools. However, most ensemble algorithms are conservative because they report only motifs that are retrieved by more than one of the motif discovery programs [15]. To help researchers evaluate motif discovery programs objectively, benchmark data sets have been created, in which known motifs are embedded into diverse sequences [16]. Each motif discovery program can then be compared based on the rate of true and false predictions.

Ideally, a motif discovery program for maize should be validated by its ability to retrieve transcription factor binding sites that have been experimentally validated. Some of the best studied transcription factor targets in maize are those of C1 and P, transcription factors which upregulate the biosynthetic enzymes responsible for production of the red-purple pigments, anthocyanin and phlobaphene, respectively [17-20]. C1 and P are homologous proteins belonging to the R2R3 Myb family of regulators [21], and they have been shown to interact with identical *cis*-acting motifs in the *A1* promoter [18,22].

In this study, first, a benchmark data set was used to compare and evaluate the accuracy of the three most used motif discovery programs, Weeder, BioProspector and MEME. Improvements were then created to reduce the limitations of each program. These improvements were incorporated into a comprehensive motif discovery pipeline customized for maize called Promzea. Promzea was then validated by asking whether it could retrieve known binding sites of maize C1 and P transcription factors [18-20,22].

Promzea accurately identified these binding sites, in particular those for P, using only a small number of input genes from these pathways. Interestingly, in a genome-wide scan, Promzea retrieved these binding sites in additional genes, including upstream genes that may help to regulate these pathways. Promzea was also tested against the Maize Development Atlas, a tissue-specific microarray dataset resource for maize [23].

## Implementation

### Overview of Promzea

**Figure 1:**
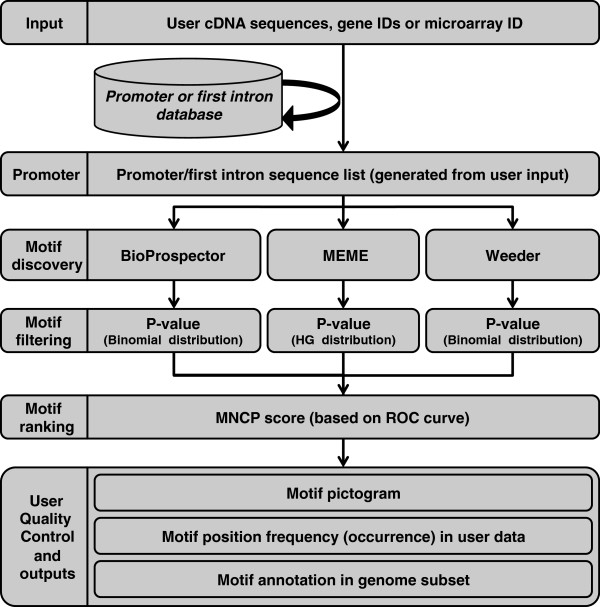
**Flow chart of the Promzea motif discovery pipeline.** Abbreviations: HG, hypergeometric distribution; MNCP, Mean Normalized Conditional Probability score.

An online pipeline called Promzea was developed to discover *de novo cis*-acting elements in maize (Figure 1) using a user-friendly interface created in Perl. Promzea is publicly available at http://www.promzea.org. The tool was subsequently expanded to include rice and Arabidopsis. For rationale and complete methodological details, see Additional file 1. Here only an overview of Promzea is provided, along with key parameters below. Briefly, using the online interface, the user first submits either a list of co-expressed cDNA FASTA sequence files, a microarray probe-set ID (in the case of maize), gene ID list or a BED file [24], for example with chromosome coordinates corresponding to peaks from ChIP-seq experiments [25]. In the case of a cDNA file, the sequences are BLAST searched against the chosen plant genome. A list of corresponding promoters to the user input is retrieved from a maize promoter database (Additional file 1). A command line version of the program is also available in the Discovery Environment of the iPlant Collaborative [26]; in this version, users can use as input a BED file allowing them to search for motifs within peaks discovered by ChIP-seq or ChIP-chip experiments [25]. The promoter data set is then searched for shared motifs using three motif discovery programs: MEME, BioProspector and Weeder (Table 1). These motif discovery programs were chosen based on using algorithms that allowed for fast and accurate and/or complimentary searching. The justification for combining multiple motif discovery programs is described in Additional file 1. The motif results are filtered, combined from all three programs, ranked and then displayed for the user along with a ranking score (MNCP, see below; Additional file 1). Finally, Promzea searches the chosen plant genome for genes containing each candidate motif, providing the user with the complete gene list and corresponding gene annotations, along with other forms of validation for the user to analyze (see Generating Promzea, below).

**Table 1:** Software programs used in Promzea

**Tool**	**Description and download site**
MEME	Multiple EM (Expectation Maximixation) for Motif Elicitation is a probabilistic *de novo* motif finding algorithm. It divides sequences into substrings and calculates the probability of each substring being a motif compared to the background. Each motif probability is recalculated during re-running using an expectation-maximisation algorithm. (http://meme.nbcr.net/downloads/meme_4.6.0.tar.gz)
Bioprospector	Gibbs sampling algorithm. Motif width is user-defined. The sequences are randomly searched to find similar motifs. Newly discovered PWM motifs are scored relative to the background. The operation is repeated until conversion of the results. Results are different at each run. (http://motif.stanford.edu/distributions/bioprospector)
Weeder	Consensus enumeration program; finds similar consensus sequences in data allowing 1 to 3 mismatches. The search is extended to the adjacent bases of the word to define the final motif. (http://159.149.160.51/modtools)
PSCAN	Determines the probability that a defined PWM motif exists in each database sequence relative to its best score. (http://159.149.160.51/pscan/)
FIMO	Finds occurrence of each defined PWM in a sequence database using a p-value calculation relative to the Markov background. (http://meme.nbcr.net/downloads/meme_4.6.0.tar.gz)
Clover	Finds occurrence of each defined PWM in a sequence database using PWM best scores compared to the background. (http://zlab.bu.edu/clover)

### Parameters of motif discovery programs used in Promzea

MEME was set to search for ten motifs with a maximum length of 10 nucleotides on both DNA strands. BioProspector was set to search for 10-nucleotide long motifs and retain only the first ten motifs found. Weeder was set to search for motifs ranging in length from 6–10 nucleotides (medium option). In addition, FIMO [27], PSCAN [28] and Clover [29] were used to retrieve motifs from the maize genome.

### Defining filters for each standalone program within Promzea using benchmark data sets

**Figure 2:**
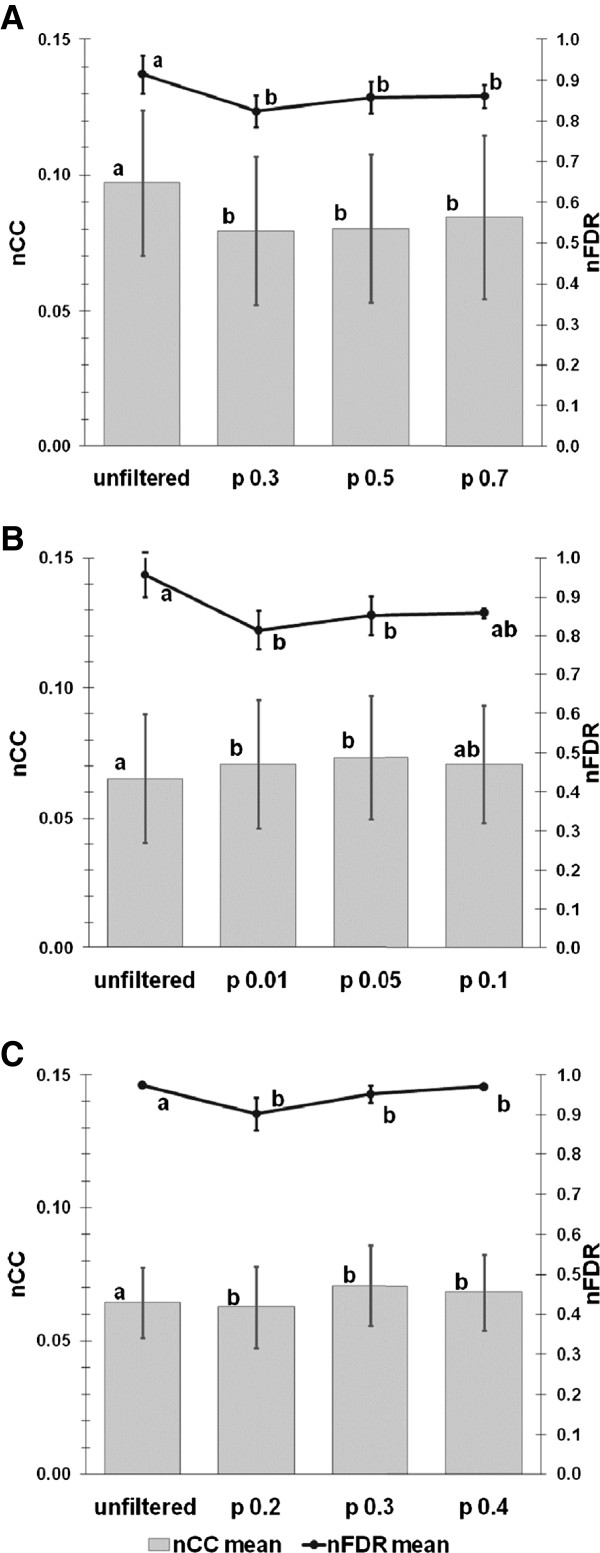
**Optimization of motif filtering for each standalone motif discovery program.** The performance of each motif discovery program, applied to the Sandve et al. (2007) benchmark data set, was measured using the nucleotide Correlation Coefficient score mean (nCC, grey bar) and the nucleotide False Discovery Ratio mean (nFDR, black line). Shown is the performance of each original program (unfiltered) and after motif filtering at three probability cut-offs (p) for: (**A**) BioProspector, using the binomial distribution; (**B**) MEME using the hypergeometric distribution; and (**C**) Weeder using the binomial distribution. FDR and nCC error bars indicate the mean confidence intervals.

As noted above, within Promzea, a custom filter was designed for each of the three motif discovery programs employed; the purpose was to reduce the false discovery ratio (nFDR) while preserving the true positives as measured using the nucleotide Correlation Coefficient (nCC score). Both nFDR and nCC are defined in Additional file 1. The filter parameters were optimized using the Sandve et al. (2007) benchmark data set [16] based on limiting the probability (pB or pH, respectively for Binomial or hypergeometric test p-values - see Additional file 1) that a motif prediction could occur randomly; the best filters were chosen based on their impact on the nFDR and nCC scores. For BioProspector, pB thresholds at 0.3, 0.5 and 0.7 significantly reduced the average nFDR score (from 0.92 with unfiltered motif discovery data to 0.82, 0.86 and 0.86, respectively, Friedman’s test p-value <0.01; Figure 2A). Though the average nCC scores between the filtered data were not significantly different from one another, the filter pB = 0.7 was chosen for BioProspector as it caused the least absolute reduction in the nCC score average compared to the unfiltered data (from 0.097 to 0.084; Figure 2A). For MEME, a significance level of 0.05 was chosen as it achieved the best balance between a significant reduction in the nFDR average (from 0.96 to 0.85, Friedman’s test p-value < 0.05) and a significant increase in the nCC average (from 0.065 to 0.073, p-value < 0.01; Figure 2B). For Weeder, a significance level of 0.3 was selected as it similarly achieved the best balance between a significant reduction in the average nFDR score (from 0.97 to 0.95, p-value < 0.001) and the largest absolute increase in the average nCC score (from 0.054 to 0.071, p-value < 0.001; Figure 2C).

### Defining the ranking of post-filtered motifs

In order to rank the predicted remaining motifs after filtering and then combining the results of all three motif discovery programs, Promzea incorporates a published metric, the Mean Normalized Conditional Probability or MNCP [30] (for details, see Additional file 1). Briefly MNCP is based on the biological principle that if a promoter/first intron contains multiple occurrences of a given motif, then the chance that motif is non-random is higher. Specifically, the MNCP score allows one to determine if the mean occurrence of any given motif in the data set (where the motif has been defined) is higher than its mean occurrence in a random set of promoters/first introns (e.g. whole genome). A motif with a higher MNCP score has a lower probability of being false.

### Generating the Promzea software pipeline

The above filtering and ranking principles were integrated into the Promzea software pipeline (Figure 1; Additional file 1: Supplementary materials and methods). To match the user input cDNA to the maize genome, full-length cDNAs were retrieved from the maize, rice and Arabidopsis genomes using their GFF files and respective genome data [3,31,32]. For each predicted gene, the corresponding promoters were compiled into a list: the flat file containing ≤1 kb of upstream sequences consisted of 39,656 predicted promoters in the case of maize, 27,416 promoters for Arabidopsis and 58,058 promoters for rice (in Additional file 2: Table S1). At least 70% of the maize genome and 35% of the rice genome are composed of transposable elements [3,31] which could generate false-positives. In order to overcome this problem, repeat-masked sequences were used to create the promoter flat files. Another problem in motif prediction is the presence of distal *cis*-acting elements possibly located up to 50 kb from the transcription starting site [33,34]. However, a maximum length of 1 kb was chosen because motif discovery algorithms struggle with larger search spaces which dilute the signal strength, and it is difficult to anticipate the exact position of a distal *cis*-acting element. Taking these limitations into account, for motif discovery in Promzea, we applied the same parameters for motif discovery and filtering as used in the Sandve et al. (2007) benchmark validation (Additional file 1: Supplementary materials and methods). In Promzea, the final filtered set of motifs is represented for the user as consensus sequence logos using Weblogo Software [35]. The predicted motifs are ranked using their MNCP scores (see above, and Additional file 1). As false positives were observed in the predictions using the benchmark data set, Promzea gives the user quality control visualizations to validate each predicted motif. One such validation is whether the motif is located at a similar position(s) within promoters of different genes. The frequency of motif occurrence at each position, as defined by each motif discovery program, is shown as a graphic using the Chart: Clicker Perl module [36]. Another validation is whether Promzea retrieves promoters of genes consistent with a common genetic pathway, by searching the maize genome for promoters containing each candidate motif. For this form of validation using gene annotations, all the genes having a defined Gene Ontology annotation were compiled into flat files using data from the Gene Ontology project of each genome.

## Results

### *In silico* validation of filtering then combining motif discovery programs using benchmark data sets

**Figure 3:**
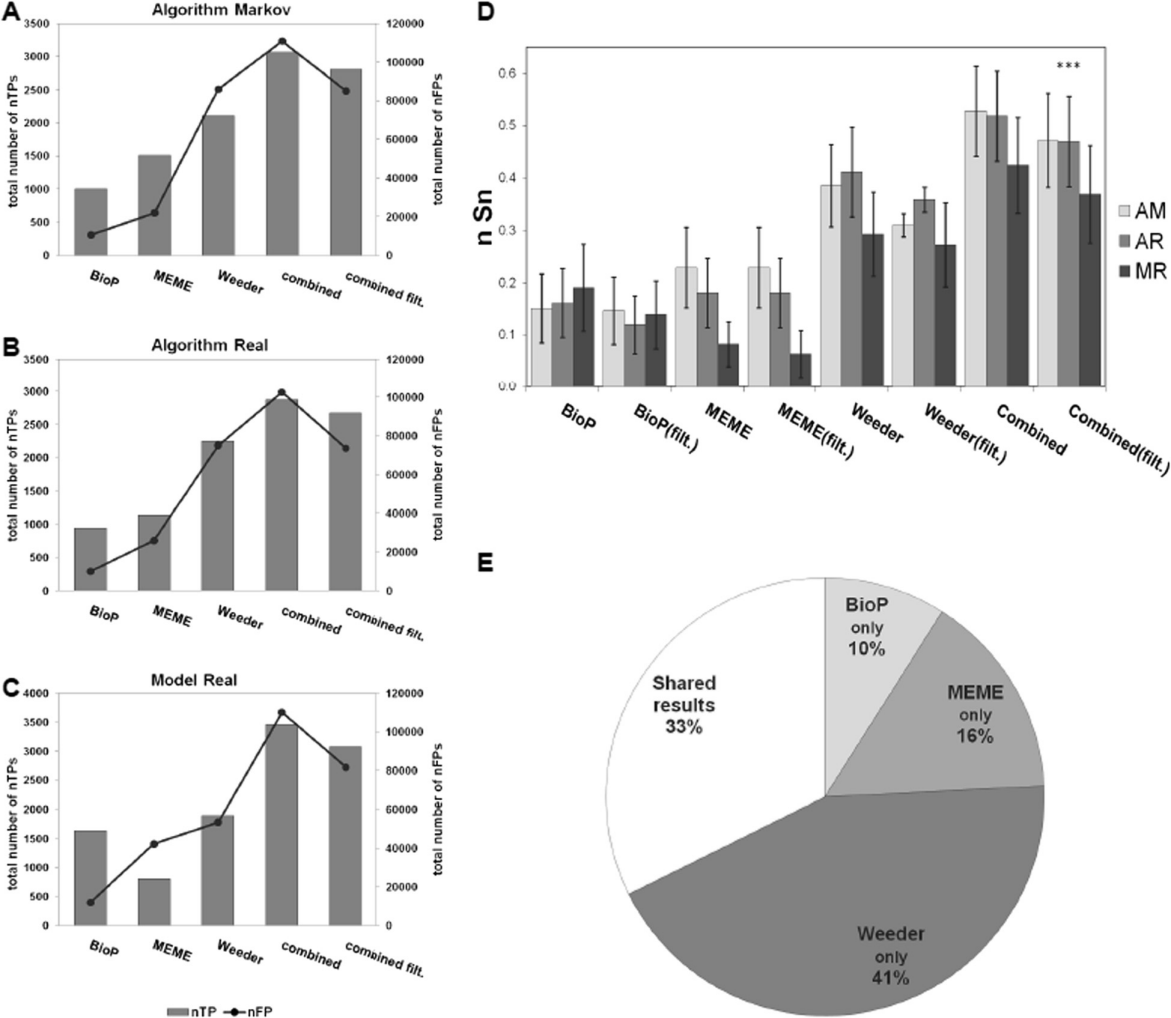
**Effectiveness of combining different motif discovery programs.** (**A**-**C**) The performance of each motif discovery program, applied to the Sandve et al. (2007) benchmark data set, was measured using the total number of true positive nucleotides (nTP, grey bars) and the total number of false positive nucleotides (nFP, black lines). Shown are scores for the three types of data sets that comprise the Sandve dataset: (**A**) synthetic (Algorithm Markov), (**B**) semi-synthetic (Algorithm Real), and (**C**) real promoters (Model Real). Shown are the scores of each standalone unfiltered program, as well as the scores after combining the outputs of the three programs without filtering (combined) or with filtering (combined filt). (**D**) The performance of each standalone program or the combined programs was compared using the average nucleotide sensitivity (nSn). Shown are the mean nSn scores for the synthetic data (AM: Algorithm Markov), semi-synthetic data (AR: Algorithm Real) and real data (MR: Model Real). The asterisks (***) indicate that the average nSn score of the combined filtered programs is statistically higher than the average nSn score using Weeder alone at p < 0.01. Each error bar represents the 95% mean confidence interval. (**E**) The partition of final true positives found by the three motif discovery tools after filtering is shown. Shared results are motif nucleotides retrieved by at least two of the standalone programs. Filtering and combining the standalone programs are the basis of Promzea.

To generate a motif discovery tool, the effectiveness of existing motif discovery tools was first analyzed using benchmark data sets containing known motifs from Sandve et al. (2007). When BioProspector (alone, unfiltered) was applied to the three types of benchmark data sets from Sandve et al. (2007), the average number of true positive motifs (nTPs) predicted was 1191 while the number of false positives (nFPs) was 10,785 (Figure 3A-C, Table 2). Unfiltered MEME predicted an average of 1145 nTPs correctly, but also 29,982 nFPs. By contrast, unfiltered Weeder predicted two-fold more nTPs (2083 on average) but a very high average number of nFPs (99,561; Table 2). However, each of the three standalone motif discovery programs appeared to identify different sets of motifs (see Additional file 3). It was thus hypothesized that combining the programs (an ensemble-type algorithm) would increase the total number of true positives. In fact, combining the programs increased the number of nTPs to 3185, a >50% increase compared to the best standalone program, Weeder, under the software parameters chosen (Figure 3A-C, Table 2). However, combining the programs also increased the number of nFPs compared to each standalone program. Filtering each motif discovery program separately (from Figure 2, earlier) before combining the results reduced the average nFPs by 25.7% compared to the combined unfiltered data yet only reduced nTPs by 8.7% (Figure 3A-C, Table 2). The nCC score after combining all three filtered programs was not significantly different compared to each standalone program, likely because nTPs and nFPs both increased (Additional file 4).

**Table 2:** Combination of motif discovery programs based on measures of true positive and false positive nucleotides

**Tools**	**Synthetic data (AM)**		**Semi-synthetic data (AR)**		**Real data (MR)**		**Averages**	
	**nTP**	**nFP**	**nTP**	**nFP**	**nTP**	**nFP**	**Average nTP**	**Average nFP**
Bioprospector	995	10668	940	9889	1638	11797	1191	10785
MEME	1503	21861	1134	25832	798	42253	1145	29982
Weeder	2104	86064	2251	74945	1895	53365	2083	99561
Combined	3067	110825	2876	102531	3462	110089	3135	107815
Combined filt.	2813	85186	2676	73534	3078	81756	2856	80159

The Table shows the numbers illustrated in Figure 3A-C. Each value is the average result of three runs for each standalone unfiltered program, as well as the scores after combining the outputs of the three programs without filtering (combined) or with filtering (combined filt).

Compared to each standalone program, combining all three filtered programs also significantly improved the ratio of software-predicted true positives versus the actual number of real motif nucleotides (sensitivity, nSn; Dunn’s Multiple Comparisons Test, p < 0.01). The nSn increased by 22% compared to the most sensitive standalone program, Weeder, under the conditions used (Figure 3D; in Additional file 2: Table S2).

The effectiveness of our strategy was further demonstrated by examining the origin of the final predicted nTPs after all three filtered results had been combined. Of the final number of nTPs retrieved from the benchmark data set, 41% were found to have been discovered by Weeder alone, 16% from MEME alone and 10% from BioProspector alone (Figure 3E). Only 33% of nTPs had been found by two or three of the standalone programs. This result confirms that widely used motif discovery programs retrieve distinct sets of motifs and that combining the predictions increases the chance of discovering new regulatory motifs.

Concerning motif ranking using the MNCP score, the analysis using the benchmark Model Real data set showed that as the MNCP score of a predicted motif increased, the chance that it was composed of nucleotide false positives decreased (in Additional file 2: Table S3).

### Validation of Promzea by comparing motif predictions to experimentally defined motifs in the maize anthocyanin and phlobaphene biosynthetic pathways

**Figure 4:**
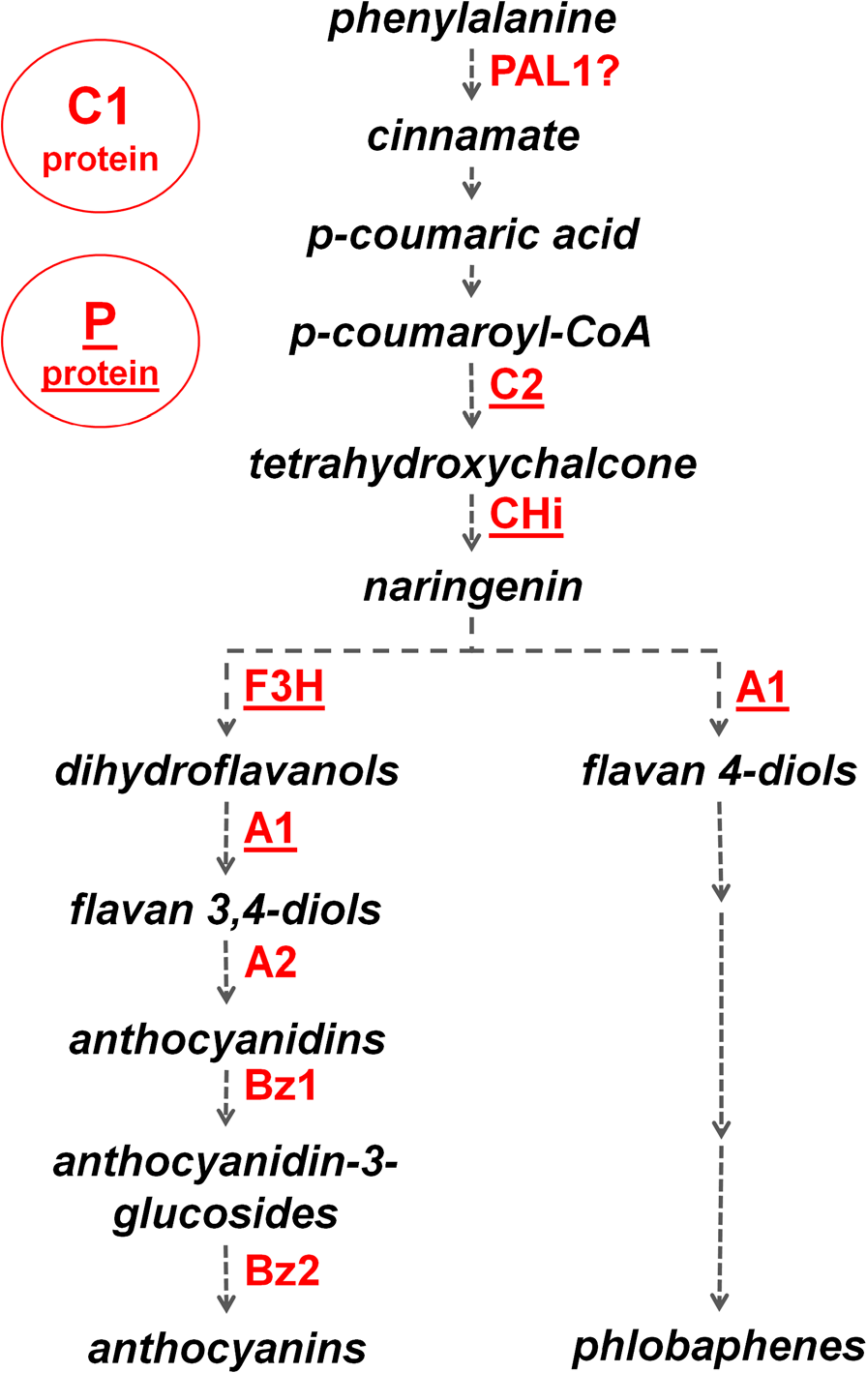
**The maize anthocyanin and phlobaphene biosynthesis pathways regulated by transcription factors C1 and P.** Genes encoding biosynthetic enzymes regulated by C1 are shown in red text; those also regulated by P are underlined. C1 and P are homologous proteins [21], and they have been shown to interact with identical binding sites in the *A1* promoter [18,22].

**Figure 5:**
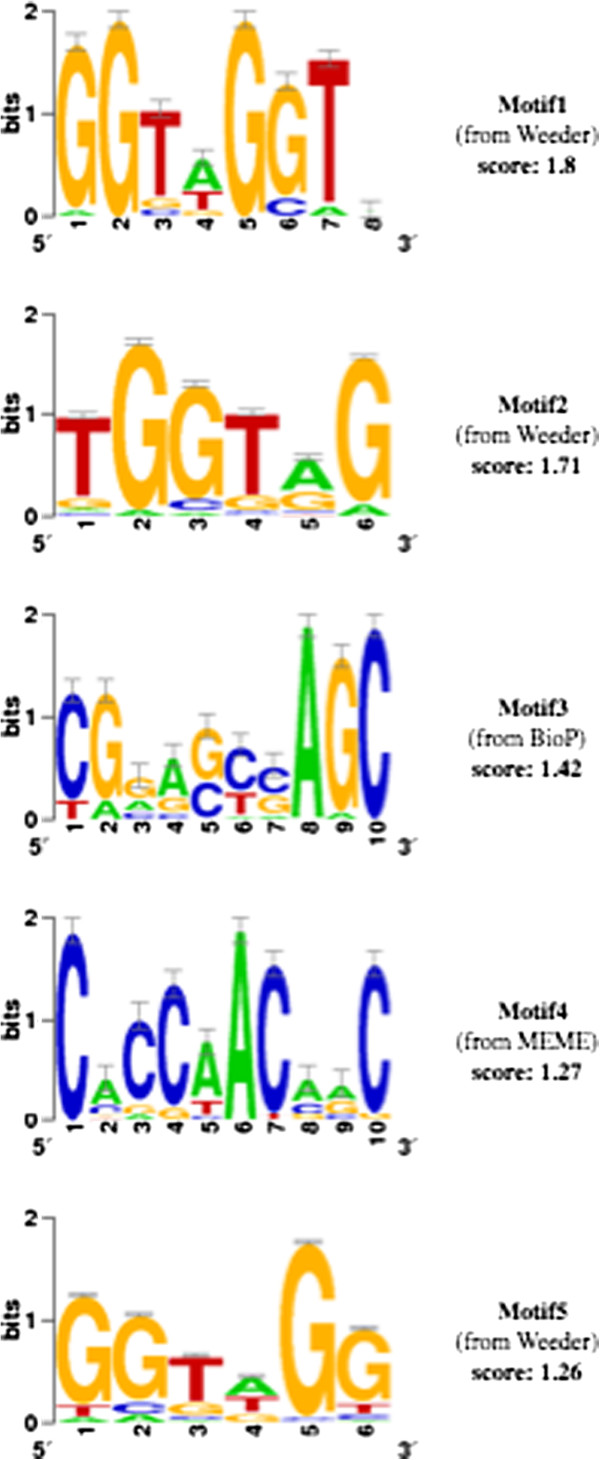
**Motifs predicted by Promzea for genes encoding the maize anthocyanin biosynthesis pathway.** Promzea searched for motifs in sequences upstream (−200 bp to +1) of the genes indicated in Figure 4 as well as their closest DNA sequence paralogs (see Methods). Shown are the sequence logos, the motif discovery program that identified each motif and the corresponding MNCP score. BioP, BioProspector.

The effectiveness of Promzea was tested based on its ability to detect experimentally defined binding sites for the maize transcription factors, C1 and P, which upregulate enzymes responsible for the biosynthesis of anthocyanin and phlobaphene, respectively (Figure 4) [17-20]. Eight gene promoters containing the C1 and P binding sites were selected (Figure 4, red labels). The corresponding cDNAs (including all close homologs, 12 in total; see Additional file 5 for a list of sequences), were used as input into Promzea following the parameters described (Additional file 1: supplementary materials and methods). Promzea retrieved 29 genes that matched these cDNAs after BLAST searching (in Additional file 2: Table S4); from the corresponding promoters, five motifs were identified along with their MNCP scores (Figure 5).

**Figure 6:**
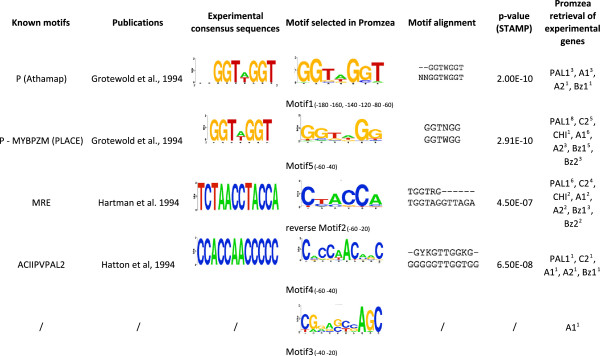
**Motifs predicted by Promzea compared to experimentally defined motifs in the literature.** Shown are the motif binding sites for transcription factor P (and C1, see text) in the phlobaphene and anthocyanin biosynthetic pathways. The preferential position of each motif predicted by Promzea is indicated in the fourth column from the right. The e-value for STAMP is indicated by the False Discovery Ratio (FDR). The superscript number in the extreme right column represents the number of motif copies present in the promoter of the indicated gene (−200 bp to +1).

Of the five motifs predicted by Promzea with MNCP scores >1, two matched the experimentally defined P binding sites (Motif1 and Motif5, Figure 6). The partially related C1 motif was found in Motif4 as described below. Based on STAMP [37], Promzea Motif1 and Motif5 were found to be highly similar to the two versions of the experimentally defined binding site of the P-protein (e-value = 2.00e-10 and 2.91e-10; Figure 6) [18,20,38]. Interestingly, Motif1 and Motif5 were overrepresented in the −60 to −40 and −80 to −60 promoter regions respectively (Figure 6), consistent with the experimentally defined −65 to −55 binding site of P in the *A1* promoter [18]. Motif1 was also overrepresented in the −120 to −100 promoter region (Figure 6), which was consistent with the other experimentally binding sites of P in the *A1* promoter at −123 to −88 [18,20]. Promzea-predicted Motif1 or Motif5 were also retrieved in four out of the five input promoters shown experimentally to contain a P binding site in their promoters (Figure 4, underlined red labels); copies of the P binding site were also predicted in the first 200 bp of the promoter of *PAL1*, encoding phenylalanine ammonia lyase (Figure 6).

Promzea-predicted Motif2 was statistically close (e-value = 4.50e-07) to the MRE binding site identified in an Arabidopsis chalcone synthase promoter [19,39] (Figure 6). In Arabidopsis, the MRE motif mediates light responsiveness [39]. Motif2 was retrieved by Promzea in the maize chalcone synthase (*C2*) promoter but also in six out of seven other input gene promoters, validating this Promzea prediction (Figure 6).

Promzea-predicted Motif4 was similar to motif ACIIPVPAL2 (e-value = 6.50e-08; Figure 6) discovered in beans [40]. The ACIIPVPAL2-like element was found in the promoter of *PAL2* (*Phenylalanine Ammonia Lyase 2*), an ortholog of the maize PAL genes necessary for the biosynthesis of phenylpropanoid secondary metabolites including anthocyanins. PAL1 is the rate-limiting step in anthocyanin biosynthesis. Promzea retrieved the ACIIPVPAL2-like motif in the promoters of *PAL1* and four additional anthocyanin genes (*C2*, *A1*, *A2* and *Bz1*), again validating Promzea predictions. Interestingly, the CA-rich region at the beginning of Motif4 was related to the C1 consensus binding site (CAACCACCAGTCAA GAC) that was previously defined experimentally [20].

The ability of Promzea to retrieve promoter motifs associated with the anthocyanin pathway that were defined experimentally not only in maize, but in also in other plant species, validates Promzea as an accurate tool for motif discovery.

### A novel candidate motif in the anthocyanin pathway and expansion of the regulatory network to the branched amino acid metabolic pathway

**Figure 7:**
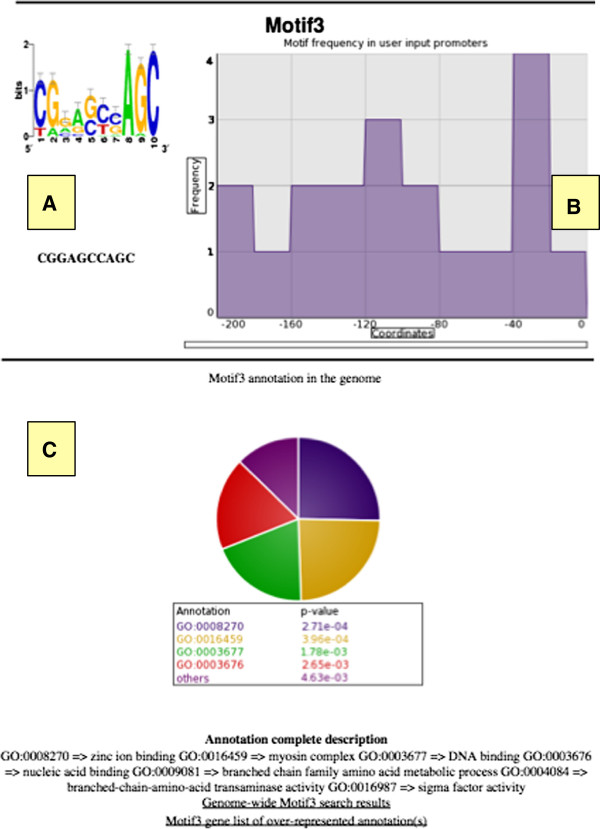
**Example of the Promzea output for anthocyanin pathway Motif3.** For each predicted motif, the following outputs are displayed: (**A**) the sequence logo (upper) and the plain consensus sequence (lower); (**B**) the frequency of occurrence of the motif at each upstream position range from the user input data set; (**C**) summary of annotations of genes containing the motif from the genome-wide retrieval (when applicable). A user can click on the Gene List link and Over-Represented Annotation link to retrieve lists of genes containing the motif and detailed gene annotations, respectively.

Promzea also retrieved Motif3 as a candidate motif in the anthocyanin biosynthetic pathway, a motif not previously defined experimentally (Figure 6). Promzea Motif3 was retrieved from the promoter of *A1* and additional paralogs of genes in the anthocyanin pathway (in Additional file 2: Table S4). Motif 3 was over-represented in the −40 to −20 promoter regions of these promoters (Figures 6 and 7). In a subsequent search of the maize genome, Motif 3 was retrieved in a total of 762 promoters (in Additional file 2: Table S5); the over-represented GO annotations of the corresponding genes, based on the hypergeometric test, identified these genes as being related to zinc ion binding (p =2.71e-04) and branched chain family amino acid metabolic processes (p = 4.63e-03) (Figure 7; Additional file 6). The latter annotation was also enriched in the four other predicted motifs (Additional file 6). As anthocyanin and phlobaphene are derived from phenylalanine, a branched amino acid, this finding appears to validate novel Motif3 as well as the Promzea pipeline, and predicts that anthocyanin biosynthesis may be transcriptionally coordinated with branched chain amino acid biosynthesis.

### Promzea retrieved additional genes that contain the same candidate motifs as the anthocyanin input promoters

As noted above for Motif3, each motif predicted by Promzea from the anthocyanin pathway was used to search the genome to retrieve genes containing that motif (Additional file 6; in Additional file 2: Table S5, anthocyanin pathway genes removed). Interestingly, the five motifs were associated with the same GO annotations: branched chain family amino acid metabolic process, heat shock protein binding, myosin complex or motor activity (Additional file 6). In total, Promzea retrieved between 131 genes (Motif1) and 762 genes (Motif3) with promoters enriched for any one of these motifs (in Additional file 2: Table S5).

Interestingly, Promzea retrieved 127 genes with promoters that contained all five motifs in the −200 bp regions of their promoters (Table 3; Additional file 6; in Additional file 2: Table S6). This list included genes encoding: PAL1, the rate-limiting step in phenylpropanoid biosynthesis which includes anthocyanins; branched amino acid enzymes (as already noted anthocyanin is derived from the branched amino acid phenylalanine); ABC-type transporters (which have been implicated in anthocyanin transport across vacuolar membranes); and regulatory proteins including transcription factors and kinases. Intriguingly, all five anthocyanin promoter motifs were also predicted in the promoters of genes similar to those involved in coordinating sugar, light, cold-temperature and low phosphate dependent activation of anthocyanin biosynthesis, namely: genes similar to gibberellin receptor GID1L2 and gibberellin 20 oxidase; genes similar to those encoding the light-regulatory pathway proteins COP1 and PIF3 (Phytochrome Interacting Factor 3) and numerous sugar transfer/modification enzymes (Table 3; in Additional file 2: Table S6).

**Table 3:** Annotated list of non-anthocyanin pathway genes in the maize genome with promoters containing all 5 of the anthocyanin/phlobaphene-related motifs predicted by Promzea (Motifs 1–5)

**Maize ID**	**Annotation (PFAM ID, Maize GDB)**
**Branched amino acid phenylpropanoid pathway**	
GRMZM2G153536	Aminotransferase class IV -- Branched-chain-amino-acid aminotransferase
GRMZM2G055899	Aminotransferase class IV (branched-chain amino acid aminotransferase 5)
GRMZM2G074604	Phenylalanine ammonia lyase 1 (PAL1)
**Putative light signaling**	
GRMZM2G104920	COP1, putative; Zinc finger, C3HC4 type (RING finger)
GRMZM2G062541	HLH DNA-binding domain related to phytochrome interacting factor 3 (PIF3)
**Putative gibberellin**	
GRMZM2G013016	Gibberellin response modulator protein (GRAS family transcription factor)
GRMZM2G021051	2OG-Fe(II) oxygenase superfamily related to gibberellin 20 oxidase
GRMZM2G026095	Carboxylesterase family related to gibberellin receptor GID1L2
**Sugar**	
AC211474.3_FG006	GDP-fucose protein O-fucosyltransferase
GRMZM2G018022	UTP-glucose-1-phosphate uridylyltransferase
GRMZM2G021243	GDP-fucose protein O-fucosyltransferase
GRMZM2G035749	Glycosyl hydrolase family 14
GRMZM2G050273	Raffinose synthase or seed inhibition protein Sip1
GRMZM2G074462	Starch binding domain
GRMZM2G082037	UDP-glucoronosyl and UDP-glucosyl transferase related to Flavonol 3-O- glucosyltransferase
GRMZM2G176630	Galactosyltransferase
GRMZM2G178278	Galactosyltransferase
GRMZM2G368827	Sugar efflux transporter for intercellular exchange/MTN3 family protein
**Transporter**	
AC206030.4_FG001	Drug transmembrane transporter
GRMZM2G094490	ABC-2 type transporter domain containing protein
GRMZM2G361066	ABC-2 type transporter
**Regulatory**	
GRMZM2G074373	bZIP transcription factor
GRMZM2G366434	AP2-like ethylene-responsive transcription factor PLETHORA 2
GRMZM2G459540	C2H2-like zinc finger protein
GRMZM2G018631	Zinc finger, C3HC4 type (RING finger)
AC196161.3_FG002	Transcription factor
GRMZM2G356718	Myb-like DNA-binding domain and Protein Phosphatase 2C
GRMZM2G398758	Myb-like DNA-binding domain
GRMZM2G027253	B3 DNA binding domain
GRMZM2G109627	No apical meristem (NAM) protein
AC203972.3_FG001	NB-ARC domain
GRMZM2G088140	G-box binding protein MFMR
GRMZM2G063961	Protein kinase domain
GRMZM2G142390	Protein kinase domain
GRMZM2G166719	Protein kinase domain
GRMZM2G163297	RNA recognition motif.
GRMZM2G459746	RNA recognition motif
GRMZM2G005622	F-box family protein
AC209810.3_FG002	Cysteine protease
**Ribosomal**	
GRMZM2G018403	Ribosomal prokaryotic L21 protein
GRMZM2G135095	Ribosomal protein S18
GRMZM2G170420	Ribosomal family S4e
GRMZM5G861978	Chloroplast 50S ribosomal protein L22
**Chaperone**	
GRMZM2G005753	DnaJ domain (Chaperone)
GRMZM2G085934	Hsp20/alpha crystallin family chaperone
GRMZM2G434839	DnaJ central domain (Chaperone)
**Cell trafficking**	
AC155377.1_FG001	Myosin family protein
GRMZM2G044348	Signal peptide peptidase
GRMZM2G047214	Nuclear Pore Localization 4 (NPL4) family protein
GRMZM2G077696	Regulator of Vps4 ATPase activity in the MVB sorting pathway
GRMZM2G095441	Syntaxin
GRMZM2G113319	Myosin family protein
GRMZM2G115775	SNARE domain
**Cytochrome P450 oxidoreductase**	
GRMZM2G394783	Oxidoreductase
AC217947.4_FG002	NADPH cytochrome P450 reductase
GRMZM2G106650	Cytochrome P450
GRMZM2G147245	Cytochrome P450 related to cinnamate-4-hydroxylase
GRMZM2G415579	NAD(P)H-dependent oxidoreductase
**Heme**	
GRMZM2G025031	Uroporphyrinogen decarboxylase (URO-D), 5th step in heme biosynthesis
GRMZM2G071745	Cytochrome b5-like Heme/Steroid binding domain
GRMZM2G028986	Cytochrome b5-like Heme/Steroid binding domain
**Cell wall or modification**	
GRMZM2G110145	Cellulose synthase
GRMZM2G113057	Hydroxyproline-rich glycoprotein family protein
GRMZM2G336879	Pectinacetylesterase
GRMZM2G352381	Pectinacetylesterase
**Other**	
AC209810.3_FG002	Cysteine protease
GRMZM2G312061	Cystatin domain and phloem filament protein PP1, proteinase inhibitor
GRMZM2G325008	Cystatin domain and phloem filament protein PP1, proteinase inhibitor
GRMZM2G004188	Nuclear excision repair XPG N-terminal domain
GRMZM2G021277	Pyridoxal-dependent decarboxylase conserved domain
GRMZM2G027241	Abscisic acid responsive TB2/DP1, HVA22 family
GRMZM2G027851	Sodium/hydrogen exchanger family
GRMZM2G043749	Uncharacterised protein family (UPF0041)
GRMZM2G047412	Chromosome segregation protein Spc25
GRMZM2G070279	Short chain dehydrogenase
GRMZM2G125448	Transferase family
GRMZM2G129979	G10 protein
GRMZM2G143703	Hydrolase, alpha/beta fold family protein
GRMZM2G146207	Tetratricopeptide repeat containing protein
GRMZM2G152370	WD domain, G-beta repeat
GRMZM2G168675	Late embryogenesis abundant protein
GRMZM2G176129	NADH dehydrogenase transmembrane subunit
GRMZM2G325575	Ferritin-1, iron storage, chloroplastic precursor
GRMZM2G348039	Mitochondrial fission ELM1
GRMZM2G465046	GDSL-like Lipase/Acylhydrolase
GRMZM2G472236	Seed maturation protein/LEA
GRMZM5G838435	Hydrolase, alpha/beta fold family domain
GRMZM5G890241	Leucine rich repeat containing protein

These data demonstrate that the genome-wide motif retrieval function of Promzea may allow researchers to predict new genes that may be part of a broader co-regulated network.

### Testing of Promzea using the maize development atlas

**Figure 8:**
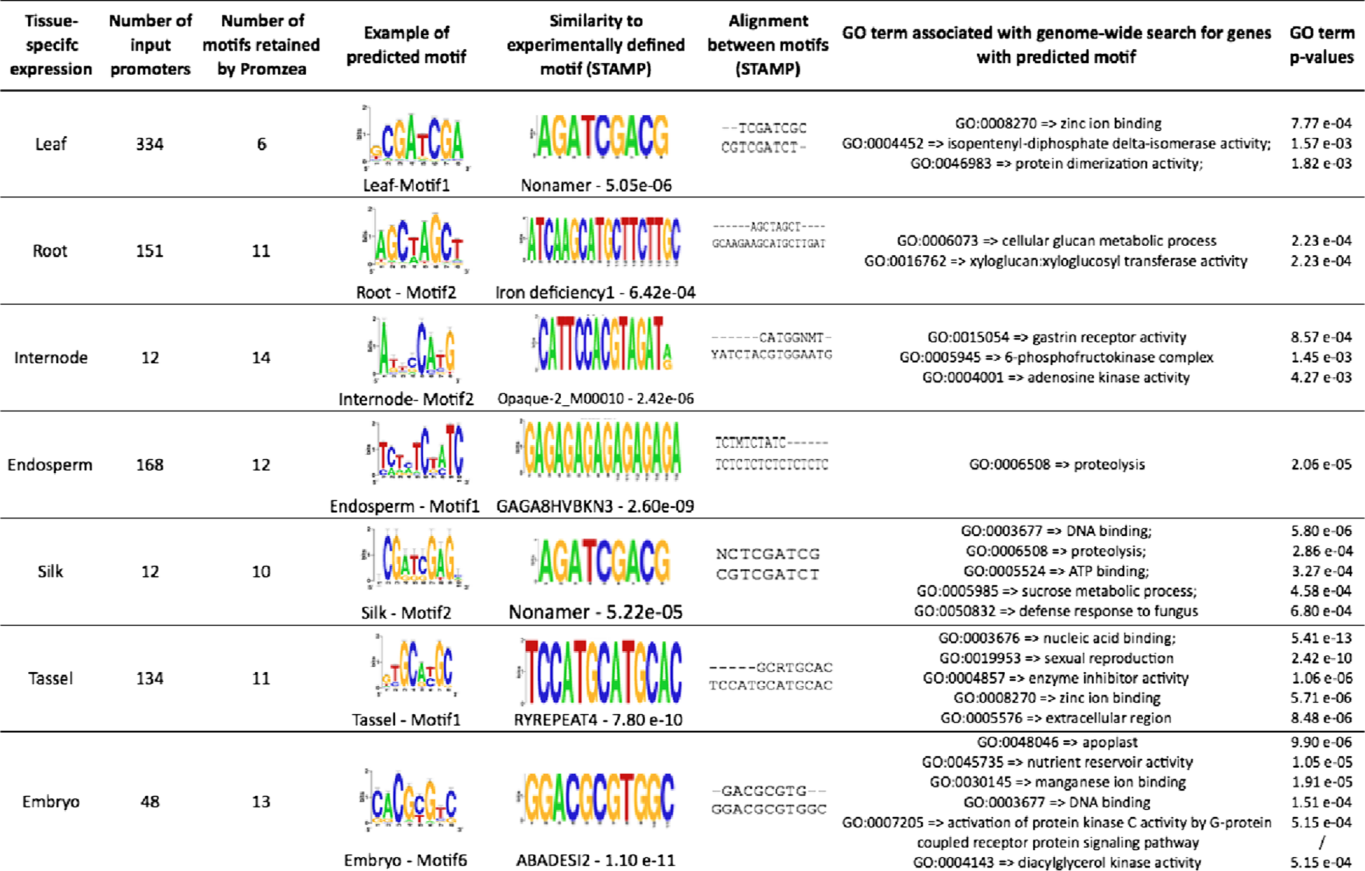
**Promzea predictions of promoter motifs associated with tissue-specific gene expression from the maize development atlas **[23]**.** Tissue-specific microarray data was used as input into Promzea, and selected motif predictions are shown and compared to previously identified promoter motifs. Please see Additional file 7 for all input sequence data and results.

To further test the Promzea pipeline using data similar to a typical user, microarray data was used from the Maize Development Atlas, a microarray data set of tissue-specific gene expression [23]. Select motifs associated with each tissue are presented (Figure 8) as well as all predicted motifs (Additional file 7).

As one case study, a list of 48 embryo-specific transcripts was used as input into Promzea (Additional file 7) from which 13 associated promoter motifs were predicted (Additional file 7). Using Clover, Promzea then retrieved genes associated with promoters in the genome that contained these motifs along with their associated GO annotation terms: genes enriched with any one of nine of the 13 motifs were annotated as having nutrient reservoir activity (Figure 8; Additional file 7), consistent with the embryo being part of the seed. Predicted embryo Motif2 and Motif6 were highly similar to the ABADESI2 *cis*-acting element (p = 5.06e-08 and p = 1.10e-11 respectively, Figure 8), known to be involved in ABA dependent desiccation during seed maturation [41].

As another case study, a total of 134 tassel-specific transcripts were investigated using Promzea, from which 11 motifs were predicted (Additional file 7). Genes enriched with any one of 9 out of the 11 motifs in their promoters were annotated as being involved in sexual reproduction (GO:0019953) consistent with the function of the tassel (Figure 8; Additional file 7).

From another reproductive tissue, the silk, 12 tissue-specific transcripts were entered into Promzea (Additional file 7). Promzea predicted 10 promoter motifs enriched in the promoters of the associated genes, of which six motifs were enriched in promoters retrieved from genome-wide searches, associated with genes involved in sucrose metabolism; other motifs were enriched in genes associated with defence responses to fungi (Figure 8), which is consistent with this tissue (e.g. against *Fusarium* which can enter through silks).

Interestingly, motifs similar to the Nonamer motif or NONAMERATH4 motif (AGATCGACG) were most frequently predicted by Promzea in silks (four out of 10 motifs), roots (3 out of 10 motifs) and leaves (one out of six motifs) (Figure 8; Additional file 7 - STAMP outputs). This motif was discovered in the promoter of the Arabidopsis gene encoding Histone 4 [42]. A mutation in Histone 4 was shown to be deleterious to cell specificity of gene expression [42].

These results appear to confirm that Promzea retrieves meaningful motifs associated with co-expressed, tissue-specific genes in data sets that would be typical of users.

## Discussion

Promzea provides the plant community with a customized interface to detect *de novo cis*-acting motifs that are over-represented in the promoters or introns of co-expressed maize genes. By filtering and combining the results of multiple standalone motif discovery programs, Promzea predicts more true motifs than current individual programs without increasing the false discovery ratio (Figure 3). For each run output, Promzea provides a ranking of the predicted motifs based on their MNCP scores (Figure 5). An MNCP score of ≤1 means that the motif is more frequently present in a random set of maize sequences than the user data set of co-expressed genes. MNCP scores can help eliminate motifs that have a general function in the plant and that are not necessary specific to a condition (e.g. tissue specificity). False positives caused by transposons and retro-elements, which are abundant in the maize and rice genomes [43], were reduced by the use of repeat masked promoter data in addition to the use of MNCP scores. False positives are a problem in any motif discovery program; furthermore, *cis-*acting motifs regulate genes at different biological levels that may or may not be of interest (e.g. developmental cue versus an environmental stimulus). Given these caveats, Promzea generates additional outputs to help a user decide which motif(s) to pursue, placing the emphasis back on the user. Promzea searches the maize genome for genes that contain each predicted motif; the corresponding gene annotations are summarized so that a user can decide whether the predicted motif is relevant to the input gene cluster (e.g. belongs to the biological pathway of interest; Figure 7C; in Additional file 2: Table S5). As gene annotations can be limiting, Promzea also generates the complete list of genes that contain each predicted motif (in Additional file 2: Table S5); a user can then search the list using relevant keywords to determine whether a predicted motif retrieves expected genes. Promzea thus narrows the number of candidate *cis-* acting motifs for subsequent experimental validation. Promzea should be especially useful to molecular biologists for the prediction of specific promoters for transgene research and targeted maize improvement; few such promoters currently exist for the maize community.

Users can maximize the utility of Promzea. First, prior to using Promzea, it is critical for the user to define robust clusters of co-expressed genes since motif discovery can be diluted by the presence of extra genes that are not part of the real gene network of interest [44,45]. Second, it is important for the user to know that Promzea employs algorithms that are stochastic in nature, including BioProspector and the selection of random background sequences required for the filtering process. As a result, each Promzea run can generate slightly different outputs. Users are recommended to run Promzea multiple times to verify the uniformity of their results. Finally, Promzea does not compare predicted motifs to motifs previously defined by the research community; for this, the user is encouraged to use STAMP to match a motif to online databases [37], or Matalign [38] for comparisons to motifs found in the literature (Figures 6 and 8). Matalign may also be used to compare the different motifs predicted by Promzea to determine if there are likely duplicates.

In this study, the Promzea pipeline was validated, first, by its ability to retrieve experimentally defined binding sites for transcription factors that regulate the maize anthocyanin and phlobaphene biosynthetic pathways (Figure 4) [18-22,46-48]. Our case study revealed that Promzea could potentially identify motifs not only from co-expression data, but also from a virtual data set, which might be expected to have a common *cis-*acting motif, such as in promoters of genes belonging to a specific biochemical pathway (Figure 4). Our case study also demonstrated that Promzea could not only retrieve valid *cis-*acting motifs, but could make novel predictions about the corresponding biological network, as 127 genes in the maize genome had promoters containing all five predicted motifs in the first 200 bp of their promoters (Table 3; in Additional file 2: Table S6). Promzea has thus predicted a broader putative co-regulated gene network than has been identified experimentally, a finding that will need further investigation.

Promzea was also tested using tissue-specific microarray data from the Maize Development Atlas [23] since this type of data is similar to that of a typical Promzea user (Figure 8). GO annotations of genes enriched for promoter motifs predicted by Promzea appeared to be logical for the specific tissue (Figure 8; Additional file 7): for instance, the GO term ‘sexual reproduction’ was over-represented in 9 out of 11 motifs predicted for tassel-specific transcripts, while the GO term ‘nutrient reserve’ was over-represented in 11 out of 13 embryo predicted motifs. Motifs in some tissues were associated with GO annotations that were not expected, or else there were multiple GO annotations, perhaps suggesting the importance of biological sampling: for example, separating cell types may be critical for software to predict meaningful *cis*-acting elements.

As a final lesson, it is noteworthy that mutants in maize transcription factors C1 and P were isolated and characterized 100 years ago [49]. The genes encoding these transcription factors began to be isolated 70–80 years later [48,50]. The binding sites for C1 and P were defined biochemically one decade later [18,20,22]. Our study shows that the bioinformatics prediction of *cis-*acting motifs may help to uncover genetic relationships even in well-studied biological pathways, in this case additional genes that are putatively co-regulated with genes encoding anthocyanin and phlobaphene biosynthetic enzymes.

## Conclusions

There was a need for a software program to help maize researchers identify *de novo cis-*acting motifs underlying co-expressed suites of genes. Here, we analyzed the accuracy of the most widely used motif discovery programs and showed that they had limited accuracy and retrieved distinct sets of motifs. We applied statistical filters to reduce the false discovery ratios of these programs and then combined the search results to improve motif prediction, and validated this approach using benchmark data. These principles were integrated into an online software program for motif discovery that was customized for maize called Promzea. Promzea was subsequently expanded to include rice and Arabidopsis. Promzea was able to retrieve experimentally defined binding sites of maize transcription factors known to regulate the anthocyanin and phlobaphene biosynthetic pathways. Interestingly, the genome-wide motif discovery function of Promzea predicted a broader network of co-regulated genes. Promzea was also tested using tissue specific microarray data from maize as input. Promzea should be a useful tool for *de novo* predictions of *cis-*acting motifs from transcriptome data. Promzea is publicly available at http://www.Promzea.org and on the Discovery Environment of the iPlant Collaborative website.

## Availability and requirements

Promzea is accessible at http://www/promzea.org and was tested on Firefox web browsers.

**Project Name:** Promzea

**Project Home Page:**http://www.promzea.org

**Operating system(s):** Platform independent

**Other requirements:** None

**Programming language:** Perl

**License:** Freely available for use

Any restrictions to use by non-academics: Promzea uses programs that require a licence for non-academics users; refer to the individual program licences.

## Abbreviations

HG: Hypergeometric distribution; MEME: Multiple Expectation-maximization for Motif Elicitation; MNCP: Mean Normalized Conditional Probability; nCC: Score, nucleotide correlation coefficient; nFDR: Nucleotide false discovery ratio; nFP: Nucleotide false positive; nTP: Nucleotide true positive; PWM: Position weight matrix

## Competing interests

The authors declare that they have no competing interests.

## Authors’ contributions

CLM developed and implemented Promzea software. CLM, MNR, DA, PDM, FF, MS, participated in the pipeline design. CLM and TL have tested and optimized Promzea Software. CLM and MNR wrote the manuscript. All authors read and approved the final manuscript.

## Supplementary Material

Additional file 1 **Supplemental materials and methods, and supplemental results.** Supplementary materials and methods describing the details of the Promzea pipeline including the calculations and optimization of the parameters for filtering, ranking and visualizations. Additional File 1 also contains the supplementary results. Click here for file

Additional file 2: Table S1 Summary of promoters and GO annotated genes incorporated into Promzea from maize, Arabidopsis and rice. This table shows the compilation of numbers of promoters, GO annotations and GO-annotated genes retrieved for each plant genome. **Table S2**. Effectiveness of combining different motif discovery programs based on nucleotide sensitivity scores (nSn). **Table S3**. The effect of applying different MNCP score cut-offs. **Table S4**. List of input cDNAs and their corresponding genes from the maize anthocyanin and phlobaphene pathways used for Promzea motif searches. Identification of additional paralogs of genes associated with the maize anthocyanin and phlobaphene biosynthetic pathways. Homologous gene sequences were retrieved that also contained similar promoter motifs, following genome-wide searches by Promzea using the motifs as input. The cDNA sequences were retrieved from Genbank. This list shows corresponding genes from MaizeSequence.org (red text, true loci; blue text, closest paralogs) and additional functional paralogs (extreme right column). **Table S5**. Gene lists and annotations found in genome-wide searches for Promzea-predicted Motifs 1–5 from promoters of the maize anthocyanin and phlobaphene biosynthetic pathways. **Table S6**. List of the 127 genes in the maize genome with promoters containing all five of the anthocyanin/phlobaphene-related motifs predicted by Promzea. Click here for file

Additional file 3 **Comparison of standalone motif discovery programs.** Different motif discovery programs predicted motifs embedded in 125 sets of sequences belonging to the Sandve et al. (2007) benchmark data set. The benchmark software calculated the nucleotide Correlation Coefficient scores (nCC scores), a measure of the correlation between the known nucleotide positions and the predicted nucleotide positions. The nCC scores are compared for: (A) BioProspector and MEME, (B) Weeder and MEME, and (C) Weeder and BioProspector. The Spearman correlation (r) between the sets of nCC scores is indicated. Click here for file

Additional file 4 **Effectiveness of combining different motif discovery programs.** The output of each motif discovery program, applied to the Sandve et al. (2007) benchmark data set, was measured using the Nucleotide Correlation Coefficient (nCC) and the nucleotide Sensitivity (nSn). Shown are scores for the three data sets that comprise the Sandve data set: (A) synthetic (Algorithm Markov), (B) semi-synthetic (Algorithm Real), and (C) real promoters (Model Real). Shown are the scores of each standalone, unfiltered program, as well as the scores after combining the outputs of the three programs with filtering (combined). The error bars represent the 95% mean confidence interval. Click here for file

Additional file 5 **Anthocyanin and phlobaphene pathway gene sequences.** The sequences of the cDNAs encoding the enzymes involved in the maize anthocyanin and phlobaphene biosynthetic pathways. A subset of these cDNAs is known to contain experimentally defined *cis*-acting elements in their promoters that permit co-expression. Click here for file

Additional file 6 **Promzea output for searches of the maize genome with the anthocyanin/phlobaphene-related motifs predicted by Promzea.** Shown is the user output from the Promzea website or command line. Click here for file

Additional file 7 **Supplemental files for testing Promzea with data sets from the Maize Development Atlas.** The zip folder contains 3 folders. The first contains the promoter input for Promzea for each maize tissue; the second folder has all the outputs from Promzea; the third folder contains the STAMP website outputs for comparisons of the predicted motifs with experimentally defined motifs. Click here for file
