# Supplementary material for: Promzea: a pipeline for discovery of co-regulatory motifs in maize and other plant species and its application to the anthocyanin and phlobaphene biosynthetic pathways and the Maize Development Atlas
Source: BMC Plant Biol. 2013 Mar 15;13:42. doi: 10.1186/1471-2229-13-42 (PMC3658923; doi:10.1186/1471-2229-13-42)
Supplement: Additional file 1 — Supplemental materials and methods, and supplemental results. Supplementary materials and methods describing the details of the Promzea pipeline including the calculations and optimization of the parameters for filtering, ranking and visualizations. Additional File 1 also contains the supplementary results. [file 1471-2229-13-42-S1.pdf]

Supplemental Materials and Methods and Results

Supplemental materials and methods

Construction of databases of upstream and first-intron sequences from maize

Flat files of putative maize promoter and first-intron sequences were created using custom Perl script programs. For the promoter database, the 5' end of each predicted immature mRNA from the maize, rice or Arabidopsis genomes were used to define the transcriptional start site (+1 - TSS) of each gene. The TSS are defined from the predicted cDNAs for each gene in the 3 plant genomes. For each gene, 1 kb of upstream sequence was extracted and used to create a flat file of predicted maize, rice and Arabidopsis promoters. Whenever a sequence gap was identified, only the relevant downstream sequence was extracted. If an upstream sequences available from a genome was less than 40 bp, it was discarded from the flat file, as the motif discovery algorithms need a minimal sequence size to accurately discover motifs. Three upstream sequence databases were created, each representing different sequence lengths (1000 bp, 500 bp, 200 bp). For the database of first-intron sequences (for maize only), a Perl script was written to recognize and retrieve predicted intron 1 sequences (based on lower case annotation).

The sequences used to generate the databases were extracted from: MaizeSequence.org (release 5b.60 Working Set - http://ftp.maizesequence.org), the rice sequence from MSU.6.14 (downloaded from Gramene – www.gramene.org) and the Arabidopsis sequence from TAIR 10.14 (ftp://ftp.arabidopsis.org/home/tair/Genes/). The sequence data originated from the Genome Sequencing Center at the Washington University School of Medicine, St. Louis, USA [1].

Comparisons of current motif discovery tools

In order to determine whether existing motif discovery programs give equivalent results, Weeder, MEME and BioProspector programs were compared. Each program was used to discover 213 known

TRANSFAC® motifs [2] embedded in 125 promoter data sets known as the benchmark data set, previously generated to help researchers make direct comparisons of the effectiveness of different motif discovery tools [3]. The data sets are grouped into three types: synthetic (Algorithm Markov, AM), semi-synthetic (Algorithm Real, AR), and real biological promoters (Model Real, MR). The success rate of each motif discovery program for each benchmark data set was measured using the following statistical outputs either generated by the benchmark web application or calculated:

- the nucleotide level sensitivity (nSn):

$$nSn = \frac{nTP}{nTP + nFN}$$

- the nucleotide False Discovery Ratio (nFDR):

$$nFDR = \frac{nFP}{nTP + nFP}$$

- the nucleotide level correlation coefficient (nCC):

$$nCC = \frac{nTP.nTN - nFN.nFP}{\sqrt{(nTP + nFN)(nTN + nFP)(nTP + nFP)(nTN + nFN)}}$$

Where nTP is the number of true positive motif nucleotides found; nTP, the number of true negative motif nucleotides found; nFP, the number of false positive motif nucleotides found; nFN, the number of false negative motif nucleotides found. nCC is a measure of the correlation between the known nucleotide positions and the predicted nucleotide positions [4].

For each benchmark data set, the nucleotide level correlation coefficient score (nCC) of motif prediction was compared between pairs of motif discovery programs. The results of all data sets were plotted and compared using the Spearman correlation coefficient (Prism 5, GraphPad Software, USA).

Filters for each standalone program

As each program generates different sets of false-positives, a custom filter was designed for each motif discovery tool to reduce the nFDR while preserving nTPs. In order to optimize the filter parameters, candidate filters were applied to the Sandve et al. (2007) benchmark data set described above and the best filters were chosen based on comparisons of nFDR and nCC (see above) using the Friedman test (non-parametric repeated measures ANOVA) (Prism 5, GraphPad Software, USA).

Each filter was based on limiting the probability (*p*) that the frequency of the candidate motif in the user data set (with sample size *N*) could occur randomly if a genome was repeatedly sampled using sample size *N*. Two sampling algorithms were tested. The first one was the motif "enrichment", without replacement of the subject (promoter sequences) that uses the hypergeometric distribution [5-7]. The second was with replacement of the sample subject following the binomial distribution [7, 8].

For MEME, the significance level (p_H) based on the hypergeometric distribution was used for the motif filtering and was calculated as follows:

$$p_H = \sum_{i=n}^{\min(N,g)} \frac{\binom{N}{i}\binom{G-N}{g-i}}{\binom{G}{g}}$$

Where *n* is the number of benchmark data set sequences containing the predicted motif out of the total number of sequences (*N)* belonging to that data set; *G* is the number of random sequences from the organism that is the most overrepresented in the data set (*G* was set at 300); *g* is the number of random sequences containing the predicted motif; *n* is the size of the motif in base pairs; and *i* is the position within the motif. To retrieve predicted motifs in both the benchmark and random data sets, FIMO was used [9]; the FIMO e-value threshold was set at $1e^{-4}$. Predicted motifs were filtered out when p_H was > 0.05. These threshold levels (FIMO value and $p_H)$ were chosen based on optimization runs using the benchmark data sets that increased the nSn but decreased nFDR.

For Weeder, there were two filters applied. The first filter removed predicted motifs with low complexity DNA stretches (> 75% of the same nucleotide). The second filter was based on the binomial distribution (p_B) based on previous works [7, 8], which gives an estimate of the probability that a motif is non-random, calculated as follows:

$$p_B = \binom{N}{n} p^n . (1-p)^{N-n}$$

Where *n* is the number of benchmark data set sequences containing the predicted motif out of the total number of sequences (*N)* belonging to that data set; and *P* is the ratio of the number of random sequences containing the predicted motif compared to the total number of random promoter sequences (set at 300). To retrieve predicted motifs in both the benchmark and random data sets, Pscan was used [10]; the Pscan score threshold was set at 0.97. Predicted motifs were filtered out when p_B was > 0.3. Pscan and p_B significance levels were also selected after optimization runs using the benchmark data sets that increased the nSn and decreased nFDR.

For BioProspector, the same binomial probability (p_B) used for Weeder was applied, except that the Pscan score threshold was set at 0.90, and predicted motifs were filtered out when p_B was >0.7. The significance levels were selected using the same method as Weeder.

To test each potential filter threshold, the average of three run results was used; for each run, a new set of random promoter sequences was generated. This multiple-run method also helped to buffer against the fact that BioProspector uses a stochastic algorithm, each run generating a different prediction.

Combining multiple programs

As each standalone program appeared to predict different but overlapping sets of motifs, the effect of combining all three filtered motif discovery programs was tested using the Sandve et al. (2007) benchmark data set. The performance of the filtered combination against each standalone program was

compared using the nSn, nCC and nFDR scores (see above) with the Friedman test (non-parametric repeated measures ANOVA) (Prism 5, GraphPad Software, USA).

Motif ranking using the MNCP score

The occurrence of motif m_x is determined in each of the promoters/first introns of the regulated user data set u belonging to the regulated promoter/first intron population N_u. Each promoter/first intron within the regulated data set is ranked according to the occurrence of motif m_x: promoter(s)/first intron(s) with the highest motif occurrence are given the 1st rank. In parallel, the occurrence of motif m_x is also determined in the random promoter/first intron data set r (regulated and non-regulated) belonging to the random promoter/first intron population N_r. If the motif m_x is a regulator of the user data set N_u, its occurrences should be higher than in the random data set N_r. Each promoter/first intron (p_x) in the regulated data set has a rank $R_u(p_x)$ and another rank in the random data set $R_r(p_x)$ A normalized ratio of the two ranks (C) for each promoter/first intron p_x is hence:

$$C(p_x) = \frac{R_u(p_x)/N_u}{R_r(p_x)/N_r}$$

C is calculated for each promoter/first introns containing motif m_x in the user data set. MNCP is the mean of all the C values. If MNCP for motif m_x is greater than 1, that motif is more represented in the regulated data set compared to the random data set. In Promzea, each motif is ranked according to its relative MNCP score. Clover software is used to retrieve the motif and estimate its occurrence in the user and random data sets from the maize genome.

Using user input sequences to extract corresponding promoter (and first-intron) regions

The Perl script was written to allow each user to generate a list of promoters (and first-intron sequences) corresponding to only those genes of interest (e.g. promoters of co-expressed genes or genes in the same biochemical pathway). The program accepts the following inputs: cDNA FASTA sequence files, microarray probe-set ID or Gramene maize ID list (Figure 1). The GeneChip Maize

Genome Array (Affymetrix) is currently supported by Promzea. In the case of the cDNA FASTA files, the Perl script matches each input cDNA to its corresponding MaizeSequence.org cDNA using standalone BLAST (NCBI, version 2.2.23) .The BLAST parameters were chosen empirically based on training data (data not shown). A cDNA sequence in the genome is considered similar to an input cDNA if the percentage identity is > 85% and the e-value is < 1e-50. The selected cDNAs from MaizeSequence.org are then used to retrieve the corresponding upstream (or first intron sequences). For microarray/Gramene ID inputs, the Perl script generates a list of the corresponding upstream (or intron 1) sequences directly.

Motif discovery, filtering, ranking and graphical output in Promzea

The user lists of promoters and first intron sequences generated above were used by our Perl script as inputs into three complimentary motif discovery programs shown to retrieve different types of motifs**:** MEME [11], BioProspector [12] and Weeder [13]. Predicted motifs from each standalone program were filtered using the parameters described above. All the filtered results are regrouped.

Understanding motif function in Promzea using functional gene annotation

Gene annotations (e.g. anthocyanin pathway) can be used to help users understand the biological function of a motif. The annotation can also be used by the user as a second form of motif validation as the annotation-defined trait should relate to the user experiment. A flat file of well-described gene annotations was first created using the “Functional-Annotations” files from MaizesSequence.org. Clover [14] is used to search the maize promoter/first-intron flat file for each predicted motif which is then matched to its corresponding gene annotation (Figure 1). The iGA Perl program [15] is used to calculate if an annotation is overrepresented for a given motif. Retrieved annotations are represented as a pie chart for the user using Chart:Clicker where each slice is - *log (annotation p-value)*. For a p-value equal

to zero, -log(p-value) is equal to *infinite* which cannot be represented on a pie-chart. To circumvent this problem, the choice has been made to replace zero p-values with p-values equal to 10^{-8}.

Validation of Promzea predictions using experimentally defined motifs

Promzea was further validated by searching a data set of promoters regulated by transcription factors *C1* and *P* which activate the maize anthocyanin and phlobaphene biosynthetic pathways, respectively [16]. To generate the input for Promzea, all cDNAs from Genbank that were annotated as corresponding to the co-regulated genes were gathered in a FASTA file (Additional File 2); a promoter sequence list was generated as described above. The 200 base promoter option was used for Promzea analysis, as the literature shows that motifs important for the expression of anthocyanin biosynthetic enzymes are within the first 200 bases of the promoter [17].

Testing of Promzea with co-expression data from the Maize Development Atlas

This gene expression data are available from the GEO database (Gene Expression Omnibus - http://www.ncbi.nlm.nih.gov/geo/). The normalized microarray data were extracted from the GSE27004 experiment of the GEO database. The data for 60 different tissues were normalized using the RMA method. The authors had produced tissue specific clusters of gene expression using the unweighted pair-group method with the arithmetic mean (UPGMA) approach and Pearson's correlation. The cluster gene lists were used from the Additional Table S4 of the publication {Sekhon, 2011 #80}. Promzea was fed with the different tissue-specific gene lists. The similarities between each predicted Promzea motif with experimentally defined motifs were determined by using the default setting of the STAMP software used with the plant databases PLACE, Athamap and Agris.

Supplemental Results

Comparisons of current motif discovery programs using benchmark data sets

The first objective was to evaluate whether each standalone motif discovery program predicted the known motif nucleotides in each data set to a similar extent. For this analysis, a previously generated benchmark data set was used consisting of 213 known motifs embedded into sets of promoter sequences [3]. For each data set, the nCC score was calculated, a measure of the correlation between the known motif nucleotide positions and the predicted motif nucleotide positions [4]. When software predicted nucleotides that exactly matched with the known binding sites (true positives, nTP), the nCC score was +1, whereas an nCC score of ≤0 indicated a random prediction. For every paired program comparison, Spearman correlations (r) of the benchmark nCC scores ranged from 0.14 to 0.36 (Additional Figure S1). In a large subset of benchmark data sets, Weeder predicted known motifs effectively (nCC>0), whereas BioProspector and MEME did not (nCC ≤0) (Additional Figure 1B, C). These results suggest that each motif discovery program retrieves a distinct set of motifs.

Supplemental References

1. Schnable PS, Ware D, Fulton RS, Stein JC, Wei F, Pasternak S, Liang C, Zhang J, Fulton L, Graves TA *et al*: **The B73 maize genome: complexity, diversity, and dynamics**. *Science* 2009, **326**(5956):1112‑1115.
2. Matys V, Kel‑Margoulis OV, Fricke E, Liebich I, Land S, Barre‑Dirrie A, Reuter I, Chekmenev D, Krull M, Hornischer K *et al*: **TRANSFAC® and its module TRANSCompel®: transcriptional gene regulation in eukaryotes**. *Nucleic Acids Research* 2006, **34**(suppl 1):D108‑D110.
3. Sandve G, Abul O, Walseng V, Drablos F: **Improved benchmarks for computational motif discovery**. *BMC Bioinformatics* 2007, **8**(1):193.
4. Tompa M, Li N, Bailey TL, Church GM, De Moor B, Eskin E, Favorov AV, Frith MC, Fu Y, Kent WJ *et al*: **Assessing computational tools for the discovery of transcription factor binding sites**. *Nature Biotechnology* 2005, **23**(1):137‑144.
5. Sinha S, Ling X, Whitfield CW, Zhai C, Robinson GE: **Genome scan for cis‑regulatory DNA motifs associated with social behavior in honey bees**. *Proceedings of the National Academy of Sciences USA* 2006, **103**(44):16352‑16357.
6. Harbison CT, Gordon DB, Lee TI, Rinaldi NJ, Macisaac KD, Danford TW, Hannett NM, Tagne J‑B, Reynolds DB, Yoo J *et al*: **Transcriptional regulatory code of a eukaryotic genome**. *Nature* 2004, **431**(7004):99‑104.
7. Linhart C, Halperin Y, Shamir R: **Transcription factor and microRNA motif discovery: The Amadeus platform and a compendium of metazoan target sets**. *Genome Research* 2008, **18**(7):1180‑1189.
8. Van Helden J, André B, Collado‑Vides J: **Extracting regulatory sites from the upstream region of yeast genes by computational analysis of oligonucleotide frequencies**. *Journal of Molecular Biology* 1998, **281**(5):827‑842.
9. Grant CE, Bailey TL, Noble WS: **FIMO: scanning for occurrences of a given motif**. *Bioinformatics* 2011, **27**(7):1017‑1018.
10. Zambelli F, Pesole G, Pavesi G: **Pscan: finding over‑represented transcription factor binding site motifs in sequences from co‑regulated or co‑expressed genes**. *Nucleic Acids Research* 2009, **37**(suppl 2):W247‑W252.
11. Bailey TL, Elkan C: **Fitting a mixture model by expectation maximization to discover motifs in biopolymers**. In: *Proceedings of the Second International Conference on Intelligent Systems for Molecular Biology; Menlo Park, California*. AAAI Press 1994: 28‑36.
12. Liu X, Brutlag D, Liu J: **BioProspector: discovering conserved DNA motifs in upstream regulatory regions of co‑expressed genes.** In: *Pacific Symposium on Biocomputing 2001*: Edited by Altman RB, Dunker AK, Hunter L, Klein TE. 2001: 127‑138.
13. Pavesi G, Zambelli F, Pesole G: **WeederH: an algorithm for finding conserved regulatory motifs and regions in homologous sequences**. *BMC Bioinformatics* 2007, **8**(1):46.
14. Frith MC, Fu Y, Yu L, Chen JF, Hansen U, Weng Z: **Detection of functional DNA motifs via statistical over‑representation**. *Nucleic Acids Research* 2004, **32**(4):1372‑1381.
15. Breitling R, Amtmann A, Herzyk P: **Iterative Group Analysis (iGA): A simple tool to enhance sensitivity and facilitate interpretation of microarray experiments**. *BMC Bioinformatics* 2004, **5**(1):34.
16. Dooner HK, Robbins TP, Jorgensen RA: **Genetic and developmental control of anthocyanin biosynthesis**. *Annual Review of Genetics* 1991, **25**(1):173‑199.
17. Bodeau JP, Walbot V: **Structure and regulation of the maize Bronze2 promoter**. *Plant Molecular Biology* 1996, **32**(4):599‑609.
