# Supplementary material for: Promzea: a pipeline for discovery of co-regulatory motifs in maize and other plant species and its application to the anthocyanin and phlobaphene biosynthetic pathways and the Maize Development Atlas
Source: BMC Plant Biol. 2013 Mar 15;13:42. doi: 10.1186/1471-2229-13-42 (PMC3658923; doi:10.1186/1471-2229-13-42)
Supplement: Additional file 2: Table S1 — Summary of promoters and GO annotated genes incorporated into Promzea from maize, Arabidopsis and rice. This table shows the compilation of numbers of promoters, GO annotations and GO-annotated genes retrieved for each plant genome. Table S2. Effectiveness of combining different motif discovery programs based on nucleotide sensitivity scores (nSn). Table S3. The effect of applying different MNCP score cut-offs. Table S4. List of input cDNAs and their corresponding genes from the maize anthocyanin and phlobaphene pathways used for Promzea motif searches. Identification of additional paralogs of genes associated with the maize anthocyanin and phlobaphene biosynthetic pathways. Homologous gene sequences were retrieved that also contained similar promoter motifs, following genome-wide searches by Promzea using the motifs as input. The cDNA sequences were retrieved from Genbank. This list shows corresponding genes from MaizeSequence.org (red text, true loci; blue text, closest paralogs) and additional functional paralogs (extreme right column). Table S5. Gene lists and annotations found in genome-wide searches for Promzea-predicted Motifs 1–5 from promoters of the maize anthocyanin and phlobaphene biosynthetic pathways. Table S6. List of the 127 genes in the maize genome with promoters containing all five of the anthocyanin/phlobaphene-related motifs predicted by Promzea. [file 1471-2229-13-42-S2.pdf]

Supplementary Table S1: Summary of promoters and GO annotated genes incorporated into Promzea from maize, Arabidopsis and rice.

species	number of protein sequences	number of promoters	number of GO annotated genes in the genome	number of GO terms found in genome
Maize	63540	39656	26737	1791
Arabidopsis	35386	27416	6534	286
Rice	68619	58058	25430	98

Supplementary Table S2. Effectiveness of combining different motif discovery programs based on nucleotide sensitivity scores.

tools	average nSn	synthetic datasets (AM)		semi-synthetic datasets (AR)		real datasets (MR)	
		nSn	C.I.	nSn	C.I.	nSn	C.I.
Bioprospector	0.17	0.15	0.07	0.16	0.07	0.19	0.08
Bioprospector filt.	0.14	0.15	0.06	0.12	0.05	0.14	0.07
MEME	0.16	0.23	0.08	0.18	0.07	0.08	0.04
MEME filt.	0.16	0.23	0.08	0.18	0.07	0.06	0.04
Weeder	0.36	0.39	0.08	0.41	0.09	0.29	0.08
Weeder filt.	0.31	0.31	0.02	0.36	0.02	0.27	0.08
Combined	0.49	0.53	0.09	0.52	0.09	0.42	0.09
Combined filt.	0.44	0.47	0.09	0.47	0.09	0.37	0.09

Note: The Table shows the numbers illustrated in Figure 3D. C.I. is the 95% confidence interval of the mean.

Supplementary Table S3. The effect of applying different MNCP score cut-offs.

MNCP threshold	nTP	nFN	nFP	nSn	nPPV	nSP	nPC	nCC	nASP	nFDR
combined	128	202	3341	0.39	0.04	0.92	0.03	0.0942	0.21	0.96
mncp 1.5	112	218	3031	0.36	0.04	0.92	0.03	0.0896	0.20	0.92
mncp 2	104	226	2602	0.35	0.04	0.93	0.04	0.0931	0.19	0.92
mncp 3	82	248	1813	0.31	0.05	0.95	0.04	0.0944	0.18	0.83
mncp 4	47	283	1151	0.22	0.03	0.96	0.02	0.0612	0.12	0.61
mncp 5	40	290	769	0.18	0.05	0.97	0.04	0.0724	0.11	0.51

Note: The motifs were predicted from the Sandve et al. (2007) benchmark dataset using the filtered, combined motif discovery program. Each statistic represents the average of three runs. The abbreviations and corresponding formulas for each statistic are described in the Methods and in Sandve et al. (2007).

Supplementary Table S4. List of input cDNAs from the maize anthocyanin pathway used for Promzea searches.

gene name	gene description	cDNA genbank ID	cDNA annotation	genes Identified by BLAST in maize genome (www.maizesequences.org)	known gene not identified
PAL1	phenylalanine ammonia-lyase	M95077.1	PAL1, Phenylalanine Ammonia Lyase	GRMZM2G074604, GRMZM2G441347, GRMZM2G170692, GRMZM2G334660, GRMZM2G063917, GRMZM2G081582, GRMZM2G029048, GRMZM2G118345	
C2	Chalcone synthase C2 (Naringenin-chalcone synthase C2)	BT042976.1	C2, chalcone synthase	GRMZM2G422750, GRMZM2G151227, GRMZM2G175812, GRMZM2G34609	
C2	Chalcone synthase C2 (Naringenin-chalcone synthase C2)	BT061979.1	C2, chalcone synthase	GRMZM2G422750, GRMZM2G151227, GRMZM2G175812, GRMZM2G34609	
CHI	chalcone isomerase	NM001156113.1	CHi, Chalcone Isomerase	GRMZM2G060011, GRMZM2G357656	
CHI	chalcone isomerase	EU963832.1	Chalcone isomerase	GRMZM2G060011, GRMZM2G357656	
F3H	flavanone 3-hydroxylase1	NM_001112225.1	F3H, putative flavonoid 3hydroxylase	GRMZM2G062396, GRMZM2G088795, GRMZM2G702514	GRMZM2G175343
A1	Anthocyanidin 3-O-glucosyltransferase - anthocyaninless1	BT034095.1	A1, dihydroflavonol-4-reductase	GRMZM2G026930, GRMZM2G013726	
A1	Anthocyanidin 3-O-glucosyltransferase - anthocyaninless1	BT043174.1	dihydroflavonol-4-reductase, putative	GRMZM2G026930, GRMZM2G013726	
A1	Anthocyanidin 3-O-glucosyltransferase - anthocyaninless1	BT067630.1	dihydroflavonol-4-reductase, putative	GRMZM2G026930, GRMZM2G013726	
A2	Leucoanthocyanidin dioxygenase - anthocyaninless2	NM001112604.1	A2	GRMZM2G345717, GRMZM2G058340, GRMZM2G057891, GRMZM5G869840	
Bz1	UDP-glucose flavonoid 3-O-glucosyltransferase - Bronze-1	DQ335126.1	Bz1, Bronze1	GRMZM2G165390, GRMZM2G022242, GRMZM2G371345	
Bz2	Probable glutathione S-transferase BZ2 -Bronze-2	AF377543.1	Bz2, Bronze2	GRMZM2G016241, GRMZM2G146887, GRMZM2G308687	

The cDNA sequences were retrieved from Genbank. In this list, corresponding genes from MaizeSequence.org (red text, true loci ; blue text, closest paralogs). The BLAST cut off threshold was set at a percentage identity > 85% and a e-value is < 1e-50

Additional Table S5. Gene lists and annotations found in genome-wide searches for Promzea-predicted motifs (M1-M5) from the maize anthocyanin biosynthetic pathway: Motif1

Gene ID	Coordinate motif start	Annotation
GRMZM2G465046	-180	lipid metabolic process|hydrolase activity, acting on ester bonds
GRMZM2G465046	-176	lipid metabolic process|hydrolase activity, acting on ester bonds
GRMZM2G077696	-75	No Description
GRMZM2G434839	-101	protein folding|heat shock protein binding|unfolded protein binding
GRMZM5G869587	-147	No Description
GRMZM5G820788	-146	No Description
GRMZM2G095441	-6	SNAP receptor activity|protein binding|intracellular protein transport|membrane|vesicle-mediated transport
GRMZM2G035749	-182	polysaccharide catabolic process|structural constituent of ribosome|intracellular|ribosome|translation|beta-amylase activity
GRMZM2G371030	-108	No Description
GRMZM2G178278	-79	vesicle-mediated transport
GRMZM2G348039	-197	No Description
GRMZM2G113057	-172	No Description
GRMZM2G055899	-57	catalytic activity|branched-chain-amino-acid transaminase activity|metabolic process|branched chain family amino acid metabolic process
GRMZM2G472236	-44	homoiothermy|ice binding|response to freezing
GRMZM2G432566	-25	protein binding|intracellular|zinc ion binding
GRMZM5G890241	-93	protein binding
GRMZM2G153536	-6	catalytic activity|branched-chain-amino-acid transaminase activity|metabolic process|branched chain family amino acid metabolic process
AC196161.3_FG002	-150	No Description
GRMZM2G142390	-120	protein kinase activity|protein serine/threonine kinase activity|ATP binding|protein amino acid phosphorylation
GRMZM2G142390	-116	protein kinase activity|protein serine/threonine kinase activity|ATP binding|protein amino acid phosphorylation
GRMZM2G143703	-139	No Description
GRMZM2G058630	-178	phosphatidylcholine-sterol O-acyltransferase activity|lipid metabolic process
GRMZM2G315862	-157	No Description
GRMZM2G459540	-11	intracellular|zinc ion binding
GRMZM2G050273	-67	No Description
GRMZM2G368827	-178	membrane
AC206030.4_FG001	-147	drug transmembrane transport|drug transmembrane transporter activity|antiporter activity|membrane|transmembrane transport
GRMZM2G352381	-67	No Description
GRMZM2G356718	-169	DNA binding|catalytic activity
GRMZM2G176630	-137	sugar binding|protein amino acid glycosylation|galactosyltransferase activity|membrane|homoiothermy|ice binding|response to freezing
GRMZM2G085303	-19	No Description
GRMZM2G312061	-20	cysteine-type endopeptidase inhibitor activity
GRMZM2G394783	-121	monooxygenase activity|iron ion binding|electron carrier activity|heme binding|oxidation reduction
AC191692.3_FG001	-136	No Description
GRMZM2G115775	-121	protein binding|membrane|vesicle-mediated transport
AC217947.4_FG002	-100	iron ion binding|FMN binding|oxidoreductase activity|oxidation reduction
GRMZM2G459746	-7	nucleic acid binding
GRMZM2G146207	-105	No Description
GRMZM2G129979	-154	nucleus
GRMZM2G325575	-47	cellular iron ion homeostasis|ferric iron binding|oxidoreductase activity|transition metal ion binding|oxidation reduction
GRMZM2G109680	-142	No Description
GRMZM2G163277	-124	No Description
GRMZM2G071745	-58	heme binding
GRMZM2G031996	-87	No Description
GRMZM2G011383	-183	No Description
GRMZM2G166719	-195	protein kinase activity|protein serine/threonine kinase activity|ATP binding|protein amino acid phosphorylation
GRMZM5G894340	-197	No Description
GRMZM2G044348	-20	aspartic-type endopeptidase activity|integral to membrane
GRMZM2G018403	-95	RNA binding|structural constituent of ribosome|intracellular|ribosome|translation
GRMZM2G366434	-191	DNA binding|transcription factor activity|regulation of transcription, DNA-dependent
GRMZM2G074604	-170	catalytic activity|cytoplasm|L-phenylalanine catabolic process|biosynthetic process|ammonia-lyase activity
GRMZM2G075366	-131	No Description
GRMZM2G038279	-62	chromatin|DNA binding|nucleus|regulation of transcription, DNA-dependent
GRMZM2G104920	-193	ubiquitin ligase complex|protein phosphatase type 2A complex|ubiquitin-protein ligase activity|protein binding|signal transduction|zinc ion binding|protein phosphatase type 2A regulator activity|protein ubiquitination|homoiothermy|ice binding|response to freezing
GRMZM5G861978	-163	structural constituent of ribosome|intracellular|ribosome|translation
GRMZM2G402797	-65	No Description
AC197340.3_FG004	-43	No Description
GRMZM2G422147	-45	No Description
GRMZM2G106650	-116	monooxygenase activity|iron ion binding|electron carrier activity|heme binding|oxidation reduction
GRMZM2G176129	-173	oxidoreductase activity, acting on NADH or NADPH, quinone or similar compound as acceptor|oxidation reduction
GRMZM2G026095	-27	metabolic process|zinc ion binding|hydrolase activity
GRMZM2G004188	-189	DNA binding|catalytic activity|nuclease activity|DNA repair
GRMZM2G163297	-171	nucleic acid binding|RNA binding
GRMZM2G146292	-170	No Description
AC202534.2_FG007	-191	No Description
GRMZM2G106604	-89	No Description
GRMZM2G021051	-165	iron ion binding|oxidoreductase activity|oxidation reduction
GRMZM2G398758	-66	DNA binding
GRMZM2G152370	-118	No Description
GRMZM2G021243	-9	No Description
GRMZM2G428905	-176	No Description
GRMZM2G336879	-19	ribonucleoside-diphosphate reductase activity|ribonucleoside-diphosphate reductase complex|DNA replication
GRMZM2G074373	-19	DNA binding|transcription factor activity|nucleus|transcription, DNA-dependent|regulation of transcription, DNA-dependent|sequence-specific DNA binding|regulation of transcription|protein dimerization activity
GRMZM2G005753	-103	protein folding|heat shock protein binding|unfolded protein binding
GRMZM2G016668	-102	protein binding|zinc ion binding
GRMZM2G018022	-99	metabolic process|nucleotidyltransferase activity
GRMZM2G135095	-103	structural constituent of ribosome|intracellular|ribosome|translation
GRMZM2G125216	-59	No Description
AC209810.3_FG002	-22	cysteine-type endopeptidase activity|proteolysis|cysteine-type peptidase activity
GRMZM2G013016	-107	No Description
GRMZM2G113319	-176	nucleotide binding|motor activity|ATP binding|myosin complex|nucleoside-triphosphatase activity
AC155377.1_FG001	-131	nucleotide binding|motor activity|ATP binding|myosin complex|nucleoside-triphosphatase activity
GRMZM2G094490	-114	nucleotide binding|ATP-dependent peptidase activity|serine-type endopeptidase activity|ATP binding|proteolysis|membrane|ATPase activity|nucleoside-triphosphatase activity

GRMZM2G552418	-128	No Description
GRMZM2G100568	-189	No Description
GRMZM2G005622	-11	No Description
GRMZM2G027253	-197	DNA binding|regulation of transcription, DNA-dependent
GRMZM2G147245	-35	monooxygenase activity|iron ion binding|electron carrier activity|heme binding|oxidation reduction
GRMZM2G063961	-199	protein kinase activity|protein serine/threonine kinase activity|ATP binding|protein amino acid phosphorylation
GRMZM2G082037	-13	metabolic process|transferase activity, transferring hexosyl groups
GRMZM2G381709	-32	small conjugating protein ligase activity|post-translational protein modification|regulation of protein metabolic process
GRMZM2G036837	-125	nucleic acid binding|zinc ion binding
GRMZM2G024674	-136	No Description
GRMZM2G109627	-10	DNA binding|regulation of transcription
AC211474.3_FG006	-132	No Description
AC211474.3_FG006	-78	No Description
GRMZM2G074824	-78	catalytic activity
GRMZM2G099052	-131	No Description
GRMZM2G168675	-38	No Description
GRMZM2G347471	-197	No Description
GRMZM2G047412	-134	No Description
GRMZM2G028986	-10	heme binding
GRMZM2G361066	-24	nucleotide binding|ATP-dependent peptidase activity|serine-type endopeptidase activity|ATP binding|proteolysis|membrane|ATPase activity|nucleoside-triphosphatase activity
GRMZM2G025031	-172	uroporphyrinogen decarboxylase activity|porphyrin biosynthetic process
GRMZM2G169584	-24	ATP binding|apoptosis
GRMZM2G047214	-112	No Description
GRMZM2G074462	-199	catalytic activity|carbohydrate metabolic process|carbohydrate binding
GRMZM2G085934	-119	No Description
GRMZM2G170420	-181	No Description
GRMZM2G125448	-195	transferase activity, transferring acyl groups other than amino-acyl groups
GRMZM2G041388	-178	No Description
GRMZM2G325008	-122	hydrolase activity, hydrolyzing O-glycosyl compounds|carbohydrate metabolic process|cellular process
GRMZM2G083555	-195	No Description
GRMZM2G027851	-173	voltage-gated potassium channel activity|cation transport|potassium ion transport|sodium ion transport|regulation of pH|voltage-gated potassium channel complex|solute:hydrogen antiporter activity|sodium:hydrogen antiporter activity|integral to membrane|transmembrane transport
GRMZM5G838435	-45	catalytic activity
GRMZM2G115579	-51	No Description
AC203972.3_FG001	-59	structural constituent of ribosome|ATP binding|intracellular|ribosome|translation|apoptosis|defense response
GRMZM2G088140	-51	DNA binding|transcription factor activity|nucleus|transcription, DNA-dependent|regulation of transcription, DNA-dependent|sequence-specific DNA binding|regulation of transcription|protein dimerization activity
GRMZM2G405170	-198	DNA binding|regulation of transcription, DNA-dependent
GRMZM2G043749	-160	No Description
GRMZM2G314593	-99	No Description
GRMZM2G110145	-163	membrane|cellulose synthase (UDP-forming) activity|cellulose biosynthetic process
GRMZM2G021277	-123	cellular amino acid and derivative metabolic process|carboxy-lyase activity|carboxylic acid metabolic process|pyridoxal phosphate binding
GRMZM2G062541	-88	nucleus|transcription regulator activity|regulation of transcription
GRMZM2G062541	-84	nucleus|transcription regulator activity|regulation of transcription
GRMZM2G415579	-103	oxidoreductase activity|oxidation reduction
GRMZM2G027241	-138	No Description
GRMZM2G107299	-22	No Description
GRMZM2G070279	-60	alcohol dehydrogenase (NAD) activity|metabolic process|2,3-dihydro-2,3-dihydroxybenzoate dehydrogenase activity|enterobactin biosynthetic process|oxidoreductase activity|oxidation reduction
GRMZM2G018631	-34	protein binding|zinc ion binding
GRMZM5G871174	-138	No Description

Supplementary Table S5 (Continued) - Motif 2

Gene ID	Coordinate motif start	Annotation
GRMZM2G465046	-180	lipid metabolic process|hydrolase activity, acting on ester bonds
GRMZM2G465046	-176	lipid metabolic process|hydrolase activity, acting on ester bonds
GRMZM2G077696	-75	No Description
GRMZM2G434839	-101	protein folding|heat shock protein binding|unfolded protein binding
GRMZM5G869587	-147	No Description
GRMZM5G820788	-146	No Description
GRMZM2G095441	-6	SNAP receptor activity|protein binding|intracellular protein transport|membrane|vesicle-mediated transport
GRMZM2G035749	-182	polysaccharide catabolic process|structural constituent of ribosome|intracellular|ribosome|translation|beta-amylase activity
GRMZM2G371030	-108	No Description
GRMZM2G178278	-79	vesicle-mediated transport
GRMZM2G348039	-197	No Description
GRMZM2G113057	-172	No Description
GRMZM2G055899	-57	catalytic activity|branched-chain-amino-acid transaminase activity|metabolic process|branched chain family amino acid metabolic process
GRMZM2G472236	-44	homoiothermy|ice binding|response to freezing
GRMZM2G432566	-25	protein binding|intracellular|zinc ion binding
GRMZM5G890241	-93	protein binding
GRMZM2G153536	-6	catalytic activity|branched-chain-amino-acid transaminase activity|metabolic process|branched chain family amino acid metabolic process
AC196161.3_FG002	-150	No Description
GRMZM2G142390	-120	protein kinase activity|protein serine/threonine kinase activity|ATP binding|protein amino acid phosphorylation
GRMZM2G142390	-116	protein kinase activity|protein serine/threonine kinase activity|ATP binding|protein amino acid phosphorylation
GRMZM2G143703	-139	No Description
GRMZM2G058630	-178	phosphatidylcholine-sterol O-acyltransferase activity|lipid metabolic process
GRMZM2G315862	-157	No Description
GRMZM2G459540	-11	intracellular|zinc ion binding
GRMZM2G050273	-67	No Description
GRMZM2G368827	-178	membrane
AC206030.4_FG001	-147	drug transmembrane transport|drug transmembrane transporter activity|antiporter activity|membrane|transmembrane transport
GRMZM2G352381	-67	No Description
GRMZM2G356718	-169	DNA binding|catalytic activity
GRMZM2G176630	-137	sugar binding|protein amino acid glycosylation|galactosyltransferase activity|membrane|homoiothermy|ice binding|response to freezing

GRMZM2G085303	-19	No Description
GRMZM2G312061	-20	cysteine-type endopeptidase inhibitor activity
GRMZM2G394783	-121	monooxygenase activity|iron ion binding|electron carrier activity|heme binding|oxidation reduction
AC191692.3_FG001	-136	No Description
GRMZM2G115775	-121	protein binding|membrane|vesicle-mediated transport
AC217947.4_FG002	-100	iron ion binding|FMN binding|oxidoreductase activity|oxidation reduction
GRMZM2G459746	-7	nucleic acid binding
GRMZM2G146207	-105	No Description
GRMZM2G129979	-154	nucleus
GRMZM2G325575	-47	cellular iron ion homeostasis|ferric iron binding|oxidoreductase activity|transition metal ion binding|oxidation reduction
GRMZM2G109680	-142	No Description
GRMZM2G163277	-124	No Description
GRMZM2G071745	-58	heme binding
GRMZM2G031996	-87	No Description
GRMZM2G011383	-183	No Description
GRMZM2G166719	-195	protein kinase activity|protein serine/threonine kinase activity|ATP binding|protein amino acid phosphorylation
GRMZM5G894340	-197	No Description
GRMZM2G044348	-20	aspartic-type endopeptidase activity|integral to membrane
GRMZM2G018403	-95	RNA binding|structural constituent of ribosome|intracellular|ribosome|translation
GRMZM2G366434	-191	DNA binding|transcription factor activity|regulation of transcription, DNA-dependent
GRMZM2G074604	-170	catalytic activity|cytoplasm|L-phenylalanine catabolic process|biosynthetic process|ammonia-lyase activity
GRMZM2G075366	-131	No Description
GRMZM2G038279	-62	chromatin|DNA binding|nucleus|regulation of transcription, DNA-dependent
GRMZM2G104920	-193	ubiquitin ligase complex|protein phosphatase type 2A complex|ubiquitin-protein ligase activity|protein binding|signal transduction|zinc ion binding|protein phosphatase type 2A regulator activity|protein ubiquitination|homoiothermy|ice binding|response to freezing
GRMZM5G861978	-163	structural constituent of ribosome|intracellular|ribosome|translation
GRMZM2G402797	-65	No Description
AC197340.3_FG004	-43	No Description
GRMZM2G422147	-45	No Description
GRMZM2G106650	-116	monooxygenase activity|iron ion binding|electron carrier activity|heme binding|oxidation reduction
GRMZM2G176129	-173	oxidoreductase activity, acting on NADH or NADPH, quinone or similar compound as acceptor|oxidation reduction
GRMZM2G026095	-27	metabolic process|zinc ion binding|hydrolase activity
GRMZM2G004188	-189	DNA binding|catalytic activity|nuclease activity|DNA repair
GRMZM2G163297	-171	nucleic acid binding|RNA binding
GRMZM2G146292	-170	No Description
AC202534.2_FG007	-191	No Description
GRMZM2G106604	-89	No Description
GRMZM2G021051	-165	iron ion binding|oxidoreductase activity|oxidation reduction
GRMZM2G398758	-66	DNA binding
GRMZM2G152370	-118	No Description
GRMZM2G021243	-9	No Description
GRMZM2G428905	-176	No Description
GRMZM2G336879	-19	ribonucleoside-diphosphate reductase activity|ribonucleoside-diphosphate reductase complex|DNA replication
GRMZM2G074373	-19	DNA binding|transcription factor activity|nucleus|transcription, DNA-dependent|regulation of transcription, DNA-dependent|sequence-specific DNA binding|regulation of transcription|protein dimerization activity
GRMZM2G005753	-103	protein folding|heat shock protein binding|unfolded protein binding
GRMZM2G016668	-102	protein binding|zinc ion binding
GRMZM2G018022	-99	metabolic process|nucleotidyltransferase activity
GRMZM2G135095	-103	structural constituent of ribosome|intracellular|ribosome|translation
GRMZM2G125216	-59	No Description
AC209810.3_FG002	-22	cysteine-type endopeptidase activity|proteolysis|cysteine-type peptidase activity
GRMZM2G013016	-107	No Description
GRMZM2G113319	-176	nucleotide binding|motor activity|ATP binding|myosin complex|nucleoside-triphosphatase activity
AC155377.1_FG001	-131	nucleotide binding|motor activity|ATP binding|myosin complex|nucleoside-triphosphatase activity
GRMZM2G094490	-114	nucleotide binding|ATP-dependent peptidase activity|serine-type endopeptidase activity|ATP binding|proteolysis|membrane|ATPase activity|nucleoside-triphosphatase activity
GRMZM2G552418	-128	No Description
GRMZM2G100568	-189	No Description
GRMZM2G005622	-11	No Description
GRMZM2G027253	-197	DNA binding|regulation of transcription, DNA-dependent
GRMZM2G147245	-35	monooxygenase activity|iron ion binding|electron carrier activity|heme binding|oxidation reduction
GRMZM2G063961	-199	protein kinase activity|protein serine/threonine kinase activity|ATP binding|protein amino acid phosphorylation
GRMZM2G082037	-13	metabolic process|transferase activity, transferring hexosyl groups
GRMZM2G381709	-32	small conjugating protein ligase activity|post-translational protein modification|regulation of protein metabolic process
GRMZM2G036837	-125	nucleic acid binding|zinc ion binding
GRMZM2G024674	-136	No Description
GRMZM2G109627	-10	DNA binding|regulation of transcription
AC211474.3_FG006	-132	No Description
AC211474.3_FG006	-78	No Description
GRMZM2G074824	-78	catalytic activity
GRMZM2G099052	-131	No Description
GRMZM2G168675	-38	No Description
GRMZM2G347471	-197	No Description
GRMZM2G047412	-134	No Description
GRMZM2G028986	-10	heme binding
GRMZM2G361066	-24	nucleotide binding|ATP-dependent peptidase activity|serine-type endopeptidase activity|ATP binding|proteolysis|membrane|ATPase activity|nucleoside-triphosphatase activity
GRMZM2G025031	-172	uroporphyrinogen decarboxylase activity|porphyrin biosynthetic process
GRMZM2G169584	-24	ATP binding|apoptosis
GRMZM2G047214	-112	No Description
GRMZM2G074462	-199	catalytic activity|carbohydrate metabolic process|carbohydrate binding
GRMZM2G085934	-119	No Description
GRMZM2G170420	-181	No Description
GRMZM2G125448	-195	transferase activity, transferring acyl groups other than amino-acyl groups
GRMZM2G041388	-178	No Description
GRMZM2G325008	-122	hydrolase activity, hydrolyzing O-glycosyl compounds|carbohydrate metabolic process|cellular process
GRMZM2G083555	-195	No Description
GRMZM2G027851	-173	voltage-gated potassium channel activity|cation transport|potassium ion transport|sodium ion transport|regulation of pH|voltage-gated potassium channel complex|solute:hydrogen antiporter activity|sodium:hydrogen antiporter activity|integral to membrane|transmembrane transport
GRMZM5G838435	-45	catalytic activity
GRMZM2G115579	-51	No Description

AC203972.3_FG001	-59	structural constituent of ribosome|ATP binding|intracellular|ribosome|translation|apoptosis|defense response
GRMZM2G088140	-51	DNA binding|transcription factor activity|nucleus|transcription, DNA-dependent|regulation of transcription, DNA-dependent|sequence-specific DNA binding|regulation of transcription|protein dimerization activity
GRMZM2G405170	-198	DNA binding|regulation of transcription, DNA-dependent
GRMZM2G043749	-160	No Description
GRMZM2G314593	-99	No Description
GRMZM2G110145	-163	membrane|cellulose synthase (UDP-forming) activity|cellulose biosynthetic process
GRMZM2G021277	-123	cellular amino acid and derivative metabolic process|carboxy-lyase activity|carboxylic acid metabolic process|pyridoxal phosphate binding
GRMZM2G062541	-88	nucleus|transcription regulator activity|regulation of transcription
GRMZM2G062541	-84	nucleus|transcription regulator activity|regulation of transcription
GRMZM2G415579	-103	oxidoreductase activity|oxidation reduction
GRMZM2G027241	-138	No Description
GRMZM2G107299	-22	No Description
GRMZM2G070279	-60	alcohol dehydrogenase (NAD) activity|metabolic process|2,3-dihydro-2,3-dihydroxybenzoate dehydrogenase activity|enterobactin biosynthetic process|oxidoreductase activity|oxidation reduction
GRMZM2G018631	-34	protein binding|zinc ion binding
GRMZM5G871174	-138	No Description
GRMZM2G352381	-67	No Description
GRMZM2G161368	-188	No Description
GRMZM2G169207	-173	No Description
GRMZM5G836606	-5	No Description
GRMZM2G003307	-58	No Description
GRMZM2G064773	-90	protein serine/threonine kinase activity|ATP binding|protein amino acid phosphorylation
GRMZM2G053631	-11	nucleic acid binding|intracellular|nucleobase, nucleoside, nucleotide and nucleic acid metabolic process|3'-5' exonuclease activity
GRMZM2G398758	-66	DNA binding
GRMZM5G899656	-86	catalytic activity|phosphoribosylamine-glycine ligase activity|ATP binding|purine base biosynthetic process
GRMZM2G413044	-16	hydrolase activity, hydrolyzing O-glycosyl compounds|cell wall|carbohydrate metabolic process|cellular glucan metabolic process|xyloglucan:xyloglucosyl transferase activity|apoplast

Supplementary Table S5 (Continued) - Motif 3

Gene ID	Coordinate motif start	Annotation
GRMZM2G352381	-67	No Description
GRMZM2G131332	-4	No Description
GRMZM2G111300	-56	zinc ion transmembrane transporter activity|zinc ion transport|membrane|integral to membrane|oxidoreductase activity, acting on paired donors, with incorporation or reduction of molecular oxygen, reduced pteridine as one donor, and incorporation of one atom of oxygen|metal ion transport|metal ion transmembrane transporter activity|transmembrane transport|oxidation reduction
GRMZM2G169207	-173	No Description
GRMZM2G430029	-89	No Description
GRMZM2G012127	-115	No Description
GRMZM2G355940	-43	translation release factor activity|translational termination
GRMZM2G398758	-66	DNA binding
GRMZM2G089343	-13	No Description
GRMZM2G134389	-14	protein tyrosine phosphatase activity|dephosphorylation|phosphatase activity
GRMZM2G028986	-10	heme binding
GRMZM2G027241	-138	No Description
GRMZM2G465046	-180	lipid metabolic process|hydrolase activity, acting on ester bonds
GRMZM2G465046	-176	lipid metabolic process|hydrolase activity, acting on ester bonds
GRMZM2G077696	-75	No Description
GRMZM2G434839	-101	protein folding|heat shock protein binding|unfolded protein binding
GRMZM5G869587	-147	No Description
GRMZM5G820788	-146	No Description
GRMZM2G095441	-6	SNAP receptor activity|protein binding|intracellular protein transport|membrane|vesicle-mediated transport
GRMZM2G035749	-182	polysaccharide catabolic process|structural constituent of ribosome|intracellular|ribosome|translation|beta-amylase activity
GRMZM2G371030	-108	No Description
GRMZM2G178278	-79	vesicle-mediated transport
GRMZM2G348039	-197	No Description
GRMZM2G113057	-172	No Description
GRMZM2G055899	-57	catalytic activity|branched-chain-amino-acid transaminase activity|metabolic process|branched chain family amino acid metabolic process
GRMZM2G472236	-44	homoiothermy|ice binding|response to freezing
GRMZM2G432566	-25	protein binding|intracellular|zinc ion binding
GRMZM5G890241	-93	protein binding
GRMZM2G153536	-6	catalytic activity|branched-chain-amino-acid transaminase activity|metabolic process|branched chain family amino acid metabolic process
AC196161.3_FG002	-150	No Description
GRMZM2G142390	-120	protein kinase activity|protein serine/threonine kinase activity|ATP binding|protein amino acid phosphorylation
GRMZM2G142390	-116	protein kinase activity|protein serine/threonine kinase activity|ATP binding|protein amino acid phosphorylation
GRMZM2G143703	-139	No Description
GRMZM2G058630	-178	phosphatidylcholine-sterol O-acyltransferase activity|lipid metabolic process
GRMZM2G315862	-157	No Description
GRMZM2G459540	-11	intracellular|zinc ion binding
GRMZM2G050273	-67	No Description
GRMZM2G368827	-178	membrane
AC206030.4_FG001	-147	drug transmembrane transport|drug transmembrane transporter activity|antiporter activity|membrane|transmembrane transport
GRMZM2G352381	-67	No Description
GRMZM2G356718	-169	DNA binding|catalytic activity
GRMZM2G176630	-137	sugar binding|protein amino acid glycosylation|galactosyltransferase activity|membrane|homoiothermy|ice binding|response to freezing
GRMZM2G085303	-19	No Description
GRMZM2G312061	-20	cysteine-type endopeptidase inhibitor activity
GRMZM2G394783	-121	monooxygenase activity|iron ion binding|electron carrier activity|heme binding|oxidation reduction
AC191692.3_FG001	-136	No Description
GRMZM2G115775	-121	protein binding|membrane|vesicle-mediated transport
AC217947.4_FG002	-100	iron ion binding|FMN binding|oxidoreductase activity|oxidation reduction
GRMZM2G459746	-7	nucleic acid binding
GRMZM2G146207	-105	No Description
GRMZM2G129979	-154	nucleus
GRMZM2G325575	-47	cellular iron ion homeostasis|ferric iron binding|oxidoreductase activity|transition metal ion binding|oxidation reduction
GRMZM2G109680	-142	No Description
GRMZM2G163277	-124	No Description
GRMZM2G071745	-58	heme binding

GRMZM2G031996	-87	No Description
GRMZM2G011383	-183	No Description
GRMZM2G166719	-195	protein kinase activity|protein serine/threonine kinase activity|ATP binding|protein amino acid phosphorylation
GRMZM5G894340	-197	No Description
GRMZM2G044348	-20	aspartic-type endopeptidase activity|integral to membrane
GRMZM2G018403	-95	RNA binding|structural constituent of ribosome|intracellular|ribosome|translation
GRMZM2G366434	-191	DNA binding|transcription factor activity|regulation of transcription, DNA-dependent
GRMZM2G074604	-170	catalytic activity|cytoplasm|L-phenylalanine catabolic process|biosynthetic process|ammonia-lyase activity
GRMZM2G075366	-131	No Description
GRMZM2G038279	-62	chromatin|DNA binding|nucleus|regulation of transcription, DNA-dependent
GRMZM2G104920	-193	ubiquitin ligase complex|protein phosphatase type 2A complex|ubiquitin-protein ligase activity|protein binding|signal transduction|zinc ion binding|protein phosphatase type 2A regulator activity|protein ubiquitination|homoiothermy|ice binding|response to freezing
GRMZM5G861978	-163	structural constituent of ribosome|intracellular|ribosome|translation
GRMZM2G402797	-65	No Description
AC197340.3_FG004	-43	No Description
GRMZM2G422147	-45	No Description
GRMZM2G106650	-116	monooxygenase activity|iron ion binding|electron carrier activity|heme binding|oxidation reduction
GRMZM2G176129	-173	oxidoreductase activity, acting on NADH or NADPH, quinone or similar compound as acceptor|oxidation reduction
GRMZM2G026095	-27	metabolic process|zinc ion binding|hydrolase activity
GRMZM2G004188	-189	DNA binding|catalytic activity|nuclease activity|DNA repair
GRMZM2G163297	-171	nucleic acid binding|RNA binding
GRMZM2G146292	-170	No Description
AC202534.2_FG007	-191	No Description
GRMZM2G106604	-89	No Description
GRMZM2G021051	-165	iron ion binding|oxidoreductase activity|oxidation reduction
GRMZM2G398758	-66	DNA binding
GRMZM2G152370	-118	No Description
GRMZM2G021243	-9	No Description
GRMZM2G428905	-176	No Description
GRMZM2G336879	-19	ribonucleoside-diphosphate reductase activity|ribonucleoside-diphosphate reductase complex|DNA replication
GRMZM2G074373	-19	DNA binding|transcription factor activity|nucleus|transcription, DNA-dependent|regulation of transcription, DNA-dependent|sequence-specific DNA binding|regulation of transcription|protein dimerization activity
GRMZM2G005753	-103	protein folding|heat shock protein binding|unfolded protein binding
GRMZM2G016668	-102	protein binding|zinc ion binding
GRMZM2G018022	-99	metabolic process|nucleotidyltransferase activity
GRMZM2G135095	-103	structural constituent of ribosome|intracellular|ribosome|translation
GRMZM2G125216	-59	No Description
AC209810.3_FG002	-22	cysteine-type endopeptidase activity|proteolysis|cysteine-type peptidase activity
GRMZM2G013016	-107	No Description
GRMZM2G113319	-176	nucleotide binding|motor activity|ATP binding|myosin complex|nucleoside-triphosphatase activity
AC155377.1_FG001	-131	nucleotide binding|motor activity|ATP binding|myosin complex|nucleoside-triphosphatase activity
GRMZM2G094490	-114	nucleotide binding|ATP-dependent peptidase activity|serine-type endopeptidase activity|ATP binding|proteolysis|membrane|ATPase activity|nucleoside-triphosphatase activity
GRMZM2G552418	-128	No Description
GRMZM2G100568	-189	No Description
GRMZM2G005622	-11	No Description
GRMZM2G027253	-197	DNA binding|regulation of transcription, DNA-dependent
GRMZM2G147245	-35	monooxygenase activity|iron ion binding|electron carrier activity|heme binding|oxidation reduction
GRMZM2G063961	-199	protein kinase activity|protein serine/threonine kinase activity|ATP binding|protein amino acid phosphorylation
GRMZM2G082037	-13	metabolic process|transferase activity, transferring hexosyl groups
GRMZM2G381709	-32	small conjugating protein ligase activity|post-translational protein modification|regulation of protein metabolic process
GRMZM2G036837	-125	nucleic acid binding|zinc ion binding
GRMZM2G024674	-136	No Description
GRMZM2G109627	-10	DNA binding|regulation of transcription
AC211474.3_FG006	-132	No Description
AC211474.3_FG006	-78	No Description
GRMZM2G074824	-78	catalytic activity
GRMZM2G099052	-131	No Description
GRMZM2G168675	-38	No Description
GRMZM2G347471	-197	No Description
GRMZM2G047412	-134	No Description
GRMZM2G028986	-10	heme binding
GRMZM2G361066	-24	nucleotide binding|ATP-dependent peptidase activity|serine-type endopeptidase activity|ATP binding|proteolysis|membrane|ATPase activity|nucleoside-triphosphatase activity
GRMZM2G025031	-172	uroporphyrinogen decarboxylase activity|porphyrin biosynthetic process
GRMZM2G169584	-24	ATP binding|apoptosis
GRMZM2G047214	-112	No Description
GRMZM2G074462	-199	catalytic activity|carbohydrate metabolic process|carbohydrate binding
GRMZM2G085934	-119	No Description
GRMZM2G170420	-181	No Description
GRMZM2G125448	-195	transferase activity, transferring acyl groups other than amino-acyl groups
GRMZM2G041388	-178	No Description
GRMZM2G325008	-122	hydrolase activity, hydrolyzing O-glycosyl compounds|carbohydrate metabolic process|cellular process
GRMZM2G083555	-195	No Description
GRMZM2G027851	-173	voltage-gated potassium channel activity|cation transport|potassium ion transport|sodium ion transport|regulation of pH|voltage-gated potassium channel complex|solute:hydrogen antiporter activity|sodium:hydrogen antiporter activity|integral to membrane|transmembrane transport
GRMZM5G838435	-45	catalytic activity
GRMZM2G115579	-51	No Description
AC203972.3_FG001	-59	structural constituent of ribosome|ATP binding|intracellular|ribosome|translation|apoptosis|defense response
GRMZM2G088140	-51	DNA binding|transcription factor activity|nucleus|transcription, DNA-dependent|regulation of transcription, DNA-dependent|sequence-specific DNA binding|regulation of transcription|protein dimerization activity
GRMZM2G405170	-198	DNA binding|regulation of transcription, DNA-dependent
GRMZM2G043749	-160	No Description
GRMZM2G314593	-99	No Description
GRMZM2G110145	-163	membrane|cellulose synthase (UDP-forming) activity|cellulose biosynthetic process
GRMZM2G021277	-123	cellular amino acid and derivative metabolic process|carboxy-lyase activity|carboxylic acid metabolic process|pyridoxal phosphate binding
GRMZM2G062541	-88	nucleus|transcription regulator activity|regulation of transcription
GRMZM2G062541	-84	nucleus|transcription regulator activity|regulation of transcription
GRMZM2G415579	-103	oxidoreductase activity|oxidation reduction
GRMZM2G027241	-138	No Description
GRMZM2G107299	-22	No Description

GRMZM2G070279	-60	alcohol dehydrogenase (NAD) activity|metabolic process|2,3-dihydro-2,3-dihydroxybenzoate dehydrogenase activity|enterobactin biosynthetic process|oxidoreductase activity|oxidation reduction
GRMZM2G018631	-34	protein binding|zinc ion binding
GRMZM5G871174	-138	No Description
GRMZM2G352381	-67	No Description
GRMZM2G161368	-188	No Description
GRMZM2G169207	-173	No Description
GRMZM5G836606	-5	No Description
GRMZM2G003307	-58	No Description
GRMZM2G064773	-90	protein serine/threonine kinase activity|ATP binding|protein amino acid phosphorylation
GRMZM2G053631	-11	nucleic acid binding|intracellular|nucleobase, nucleoside, nucleotide and nucleic acid metabolic process|3'-5' exonuclease activity
GRMZM2G398758	-66	DNA binding
GRMZM5G899656	-86	catalytic activity|phosphoribosylamine-glycine ligase activity|ATP binding|purine base biosynthetic process
GRMZM2G413044	-16	hydrolase activity, hydrolyzing O-glycosyl compounds|cell wall|carbohydrate metabolic process|cellular glucan metabolic process|xyloglucan:xyloglucosyl transferase activity|apoplast
GRMZM2G082198	-43	nucleic acid binding
GRMZM2G062318	-99	No Description
GRMZM2G402156	-48	DNA binding
AC197699.3_FG008	-21	No Description
GRMZM2G448656	-32	No Description
GRMZM2G083716	-100	protein binding|ATP binding|cytoplasm|protein folding|protein refolding|cellular protein metabolic process
AC187789.3_FG001	-46	No Description
GRMZM2G455687	-158	No Description
GRMZM2G138370	-179	No Description
GRMZM2G134049	-114	No Description
GRMZM2G047867	-55	catalytic activity
GRMZM2G081474	-22	histone deacetylase activity|histone deacetylation|acetoin metabolic process
GRMZM2G305238	-51	No Description
GRMZM2G029690	-186	protein binding|zinc ion binding
GRMZM2G028254	-19	No Description
GRMZM2G451746	-99	No Description
GRMZM2G116452	-176	peroxidase activity|response to oxidative stress|heme binding|oxidation reduction
GRMZM2G704352	-113	No Description
GRMZM2G121360	-179	metal ion transport|metal ion binding
GRMZM5G828123	-18	No Description
GRMZM5G828987	-59	hydrolase activity, hydrolyzing O-glycosyl compounds|carbohydrate metabolic process
GRMZM2G126821	-155	structural constituent of ribosome|intracellular|ribosome|translation|small ribosomal subunit
GRMZM2G358931	-174	structural constituent of ribosome|cyclin-dependent protein kinase inhibitor activity|histamine receptor activity|intracellular|nucleus|ribosome|translation|cell cycle arrest|G-protein coupled receptor protein signaling pathway|integral to membrane
GRMZM5G809743	-135	No Description
GRMZM2G022907	-18	No Description
GRMZM2G139852	-93	No Description
GRMZM2G153536	-8	catalytic activity|branched-chain-amino-acid transaminase activity|metabolic process|branched chain family amino acid metabolic process
GRMZM2G085427	-131	DNA binding
AC231441.1_FG004	-152	No Description
GRMZM2G161337	-105	tryptophan biosynthetic process|anthranilate synthase activity|biosynthetic process|oxo-acid-lyase activity
GRMZM2G345330	-164	No Description
GRMZM2G174284	-66	transcription factor activity|regulation of transcription, DNA-dependent|sequence-specific DNA binding
GRMZM2G013299	-16	glutathione peroxidase activity|response to oxidative stress|oxidation reduction
GRMZM2G552278	-164	No Description
GRMZM5G828488	-17	No Description
GRMZM2G030636	-192	No Description
GRMZM2G089291	-78	iron ion binding|sodium ion transport|regulation of pH|electron carrier activity|sodium:hydrogen antiporter activity|membrane|integral to membrane|oxidoreductase activity|FAD binding|oxidation reduction
GRMZM2G001895	-120	binding|heat shock protein binding
GRMZM5G841743	-48	No Description
GRMZM2G030436	-136	transferase activity, transferring acyl groups other than amino-acyl groups
GRMZM2G123418	-20	No Description
GRMZM2G034680	-59	No Description
GRMZM2G303010	-190	No Description
GRMZM2G119339	-22	No Description
GRMZM2G008250	-116	No Description
GRMZM2G042313	-44	structural constituent of ribosome|intracellular|ribosome|translation|transport|amino acid transport|amino acid transmembrane transporter activity|membrane|transmembrane transport
GRMZM2G093809	-60	protein kinase activity|protein serine/threonine kinase activity|protein binding|ATP binding|protein amino acid phosphorylation
GRMZM2G009117	-190	metal ion transport|metal ion binding
GRMZM2G472671	-90	nucleus|type I hypersensitivity|transcription regulator activity|regulation of transcription
GRMZM2G340305	-56	DNA binding|regulation of transcription
GRMZM2G016439	-106	two-component signal transduction system (phosphorelay)|signal transducer activity
GRMZM2G449163	-112	protein tyrosine phosphatase activity|dephosphorylation|phosphatase activity
GRMZM2G160560	-21	membrane
GRMZM2G564469	-174	No Description
GRMZM2G101105	-41	RNA binding|double-stranded RNA binding|ribonuclease III activity|intracellular|RNA processing
GRMZM2G703910	-101	No Description
GRMZM2G067626	-18	No Description
GRMZM2G131591	-187	protein binding|zinc ion binding
GRMZM2G135866	-189	protein binding|zinc ion binding
GRMZM2G459291	-22	catalytic activity
GRMZM5G830585	-77	No Description
GRMZM2G145412	-112	No Description
GRMZM2G163761	-19	transcription factor activity|catalytic activity|nucleus|regulation of transcription, DNA-dependent|transcription regulator activity|sequence-specific DNA binding|cellular metabolic process
GRMZM2G036505	-151	No Description
GRMZM2G416426	-134	No Description
GRMZM2G174083	-115	motor activity|ATP binding|myosin complex
GRMZM2G053023	-176	cell redox homeostasis
GRMZM2G022662	-147	translation elongation factor activity|translational elongation
GRMZM2G104125	-10	protein kinase activity|protein serine/threonine kinase activity|calcium ion binding|ATP binding|protein amino acid phosphorylation
GRMZM2G347056	-54	methylenetetrahydrofolate reductase (NADPH) activity|methionine metabolic process|oxidation reduction
GRMZM2G016084	-163	RNA binding
GRMZM2G023557	-66	DNA binding
GRMZM2G339848	-151	transcription factor activity|regulation of transcription, DNA-dependent|sequence-specific DNA binding

GRMZM2G140527	-121	copper ion binding|oxidoreductase activity|oxidation reduction
GRMZM2G122498	-180	monooxygenase activity|iron ion binding|electron carrier activity|heme binding|oxidation reduction
AC218998.2_FG001	-160	monooxygenase activity|iron ion binding|electron carrier activity|heme binding|oxidation reduction
GRMZM2G434299	-51	No Description
GRMZM2G011896	-21	protein kinase activity|protein serine/threonine kinase activity|protein binding|ATP binding|protein amino acid phosphorylation
GRMZM2G074293	-28	No Description
GRMZM2G171420	-73	No Description
AC209029.2_FG002	-173	No Description
GRMZM2G702379	-148	No Description
GRMZM2G170829	-98	manganese ion binding|nutrient reservoir activity|apoplast
AC196475.3_FG004	-175	methyltransferase activity|O-methyltransferase activity|protein dimerization activity
GRMZM2G055520	-99	No Description
GRMZM2G354618	-182	nucleus|transcription regulator activity|regulation of transcription
GRMZM2G702419	-190	No Description
GRMZM2G036217	-8	No Description
GRMZM2G425999	-181	potassium ion transport|potassium ion transmembrane transporter activity|membrane
AC205664.3_FG001	-78	No Description
AC191692.3_FG001	-138	No Description
GRMZM2G013214	-131	metabolic process|oxidoreductase activity|homoiothermy|ice binding|response to freezing|oxidation reduction
GRMZM2G168171	-124	No Description
AC205269.3_FG003	-19	No Description
GRMZM2G394945	-49	No Description
GRMZM2G122979	-138	No Description
GRMZM2G135132	-198	6-phosphofructokinase activity|adenosine kinase activity|ribokinase activity|6-phosphofructokinase complex|D-ribose metabolic process|glycolysis|purine ribonucleoside salvage
GRMZM2G129675	-66	No Description
GRMZM2G147780	-70	Rho guanyl-nucleotide exchange factor activity
GRMZM2G076631	-46	catalytic activity
GRMZM2G063276	-9	hydrolase activity
GRMZM2G081843	-163	transporter activity|transport|membrane|integral to membrane|transmembrane transport
GRMZM2G108147	-174	catalytic activity
GRMZM2G008819	-158	copper ion binding|electron carrier activity
GRMZM2G138949	-61	No Description
GRMZM2G038479	-24	nucleus|transcription regulator activity|regulation of transcription
GRMZM2G406552	-116	No Description
GRMZM2G131957	-21	nucleic acid binding|structural constituent of cell wall
GRMZM2G332505	-198	nucleosome|DNA binding|nucleus|nucleosome assembly|amino acid transport|membrane
GRMZM2G051541	-55	No Description
GRMZM2G049839	-138	aspartic-type endopeptidase activity|proteolysis
GRMZM2G336761	-30	protein binding|zinc ion binding
GRMZM2G143445	-159	No Description
GRMZM2G010152	-108	DNA binding|catalytic activity|DNA topoisomerase (ATP-hydrolyzing) activity|copper-exporting ATPase activity|copper ion binding|ATP binding|chromosome|DNA topological change|ATP biosynthetic process|copper ion transport|metabolic process|mercury ion transmembrane transporter activity|ATPase activity, coupled to transmembrane movement of ions, phosphorylative mechanism|mercury ion transport|membrane|integral to membrane|hydrolase activity, acting on acid anhydrides, catalyzing transmembrane movement of substances|metal ion transport|metal ion binding|metal ion transmembrane transporter activity
GRMZM2G088258	-11	oxidoreductase activity
GRMZM2G168888	-195	thiosulfate sulfurtransferase activity|sulfate transport
GRMZM2G119640	-8	nucleic acid binding|zinc ion binding
GRMZM5G862317	-196	lipid metabolic process|hydrolase activity, acting on ester bonds
AC190636.3_FG005	-116	oxidoreductase activity
GRMZM2G133481	-56	catalytic activity
GRMZM2G002765	-164	catalytic activity
GRMZM2G002765	-161	catalytic activity
GRMZM2G110881	-119	catalytic activity|3-beta-hydroxy-delta5-steroid dehydrogenase activity|steroid biosynthetic process|metabolic process|oxidoreductase activity|cellular metabolic process|coenzyme binding
GRMZM5G810054	-55	No Description
GRMZM2G701300	-41	No Description
GRMZM2G171953	-103	No Description
GRMZM2G077295	-28	protein binding|intracellular protein transport|ER to Golgi vesicle-mediated transport|zinc ion binding|COPII vesicle coat
GRMZM2G019958	-196	No Description
GRMZM2G160664	-20	DNA binding|ATP binding|nucleus|DNA replication|DNA-dependent DNA replication initiation
GRMZM2G102601	-131	two-component sensor activity|ATP binding|signal transduction|membrane|phosphorylation|transferase activity, transferring phosphorus-containing groups
AC194402.2_FG004	-110	No Description
GRMZM2G018943	-166	cellular metabolic process
GRMZM2G388576	-116	No Description
GRMZM2G403218	-17	No Description
GRMZM2G162544	-16	SNAP receptor activity|protein binding|intracellular protein transport|membrane|vesicle-mediated transport
GRMZM2G462081	-14	No Description
GRMZM2G701448	-177	DNA binding|DNA topoisomerase (ATP-hydrolyzing) activity|ATP binding|chromosome|DNA topological change
GRMZM2G123097	-186	No Description
GRMZM2G054745	-191	structural molecule activity|viral capsid|heat shock protein binding
GRMZM2G102447	-37	No Description
AC185261.4_FG002	-12	No Description
GRMZM2G109070	-154	No Description
GRMZM2G023798	-19	catalytic activity|intramolecular transferase activity
GRMZM2G041878	-127	No Description
GRMZM2G105331	-176	No Description
GRMZM2G138423	-53	transporter activity|binding|mitochondrial inner membrane|transport|membrane|transmembrane transport
GRMZM2G039089	-15	protein folding|heat shock protein binding|unfolded protein binding
GRMZM2G101523	-43	zinc ion binding
GRMZM2G079682	-100	beta-1,4-mannosylglycoprotein 4-beta-N-acetylglucosaminyltransferase activity|protein amino acid N-linked glycosylation|membrane
GRMZM2G128902	-190	No Description
GRMZM5G859330	-33	No Description
GRMZM2G463711	-90	No Description
GRMZM2G702126	-41	No Description
GRMZM2G056762	-40	No Description
GRMZM2G090419	-25	hydrolase activity
GRMZM2G130548	-99	No Description
GRMZM2G072300	-76	heat shock protein binding

GRMZM2G086027	-41	No Description
GRMZM2G119769	-57	homoiothermy|ice binding|response to freezing
GRMZM2G465769	-150	No Description
GRMZM2G017869	-80	lipid metabolic process|hydrolase activity, acting on ester bonds
GRMZM2G385818	-163	chromatin|DNA binding|RNA binding|structural constituent of ribosome|nucleus|regulation of transcription, DNA-dependent|translation
GRMZM2G076212	-22	protein kinase activity|protein serine/threonine kinase activity|ATP binding|protein amino acid phosphorylation
GRMZM2G081886	-182	phosphoglycerate dehydrogenase activity|L-serine biosynthetic process|potassium ion transport|metabolic process|cation transmembrane transporter activity|amino acid binding|oxidoreductase activity, acting on the CH-OH group of donors, NAD or NADP as acceptor|cofactor binding|NAD or NADH binding|oxidation reduction
GRMZM2G055844	-194	protein kinase activity|protein serine/threonine kinase activity|protein binding|ATP binding|protein amino acid phosphorylation
GRMZM2G106010	-99	No Description
GRMZM5G825909	-68	No Description
GRMZM2G039711	-20	No Description
GRMZM2G178709	-64	No Description
GRMZM2G147268	-171	cellular amino acid metabolic process|biosynthetic process|transferase activity, transferring nitrogenous groups|lyase activity|pyridoxal phosphate binding
GRMZM2G457697	-52	No Description
GRMZM2G138770	-104	nucleotide binding|ATP binding|nucleoside-triphosphatase activity
GRMZM2G441748	-118	No Description
GRMZM2G050925	-19	No Description
GRMZM2G147685	-56	nucleus|transcription regulator activity|regulation of transcription
GRMZM2G025042	-66	No Description
GRMZM2G509030	-154	No Description
GRMZM2G013357	-99	monooxygenase activity|iron ion binding|electron carrier activity|heme binding|oxidation reduction
GRMZM2G178199	-32	No Description
GRMZM2G703185	-70	No Description
GRMZM2G145666	-49	structural constituent of cell wall
GRMZM2G703193	-31	No Description
GRMZM2G180691	-188	hydrolase activity
GRMZM2G503606	-138	No Description
GRMZM2G066638	-115	motor activity|ATP binding|myosin complex
GRMZM2G067756	-39	No Description
GRMZM2G146685	-113	No Description
GRMZM2G037574	-8	intracellular|zinc ion binding
GRMZM2G002053	-27	type I hypersensitivity|homoiothermy|ice binding|response to freezing
GRMZM2G015409	-87	protein binding|zinc ion binding
GRMZM2G379563	-118	No Description
GRMZM2G158629	-163	cysteine-type endopeptidase activity|fatty acid synthase activity|fatty acid synthase complex|proteolysis|fatty acid biosynthetic process|metabolic process|oxidoreductase activity|oxidation reduction
GRMZM2G703338	-142	No Description
GRMZM2G016106	-12	nucleic acid binding
AC219035.2_FG006	-136	No Description
GRMZM2G128771	-29	GTPase activity|protein binding|GTP binding|intracellular|intracellular protein transport|nucleocytoplasmic transport|signal transduction|small GTPase mediated signal transduction|protein transport
GRMZM2G350773	-12	No Description
GRMZM2G103881	-131	No Description
GRMZM2G062019	-134	metabolic process|hydrolase activity
GRMZM2G062019	-128	metabolic process|hydrolase activity
GRMZM2G062019	-122	metabolic process|hydrolase activity
GRMZM2G062019	-116	metabolic process|hydrolase activity
GRMZM2G056329	-82	ATP binding
GRMZM2G022763	-137	nucleic acid binding|RNA binding|nucleus|mRNA processing
GRMZM2G001388	-63	protein binding
GRMZM2G398758	-68	DNA binding
GRMZM2G345270	-11	sugar:hydrogen symporter activity|transport|phosphoenolpyruvate-dependent sugar phosphotransferase system
GRMZM2G035482	-61	No Description
GRMZM2G308910	-11	DNA binding|structural constituent of ribosome|ligand-dependent nuclear receptor activity|intracellular|nucleus|ribosome|regulation of transcription, DNA-dependent|translation
GRMZM2G390236	-180	No Description
GRMZM2G336879	-21	ribonucleoside-diphosphate reductase activity|ribonucleoside-diphosphate reductase complex|DNA replication
GRMZM2G336929	-12	transferase activity, transferring glycosyl groups
GRMZM2G013471	-30	No Description
GRMZM2G170307	-31	heat shock protein binding
AC200904.3_FG003	-42	No Description
GRMZM2G013971	-135	ubiquitin ligase complex|ubiquitin-protein ligase activity|protein binding|zinc ion binding|protein ubiquitination
GRMZM2G046924	-40	transferase activity
GRMZM2G010384	-151	No Description
GRMZM2G081912	-151	ubiquitin ligase complex|ubiquitin-protein ligase activity|protein binding|zinc ion binding|protein ubiquitination
GRMZM2G701533	-191	No Description
GRMZM2G038356	-71	GTP binding|intracellular|intracellular protein transport|small GTPase mediated signal transduction
AC205833.3_FG002	-52	No Description
GRMZM2G457411	-39	protein binding|zinc ion binding
GRMZM2G053692	-61	structural molecule activity|protein binding|retrograde vesicle-mediated transport, Golgi to ER|COPI vesicle coat
GRMZM2G138268	-21	nucleus|regulation of transcription
GRMZM2G366142	-91	No Description
GRMZM2G013016	-109	No Description
GRMZM2G053284	-49	protein binding|ATP binding
GRMZM2G177518	-179	No Description
GRMZM2G004314	-184	No Description
GRMZM2G065290	-72	transcription factor activity|sequence-specific DNA binding|regulation of transcription
GRMZM2G021704	-17	dihydroorotase activity|hydrolase activity|hydrolase activity, acting on carbon-nitrogen (but not peptide) bonds, in cyclic amides|pyrimidine base biosynthetic process
GRMZM2G039246	-131	two-component signal transduction system (phosphorelay)|signal transducer activity
GRMZM2G702144	-71	No Description
GRMZM2G159724	-17	malic enzyme activity|malate metabolic process|oxidoreductase activity|oxidoreductase activity, acting on the CH-OH group of donors, NAD or NADP as acceptor|metal ion binding|NAD or NADH binding|oxidation reduction
GRMZM5G814782	-186	No Description
GRMZM5G814803	-24	No Description
AC155377.1_FG002	-55	aspartic-type endopeptidase activity|proteolysis
GRMZM2G481362	-119	hydrolase activity
GRMZM2G467147	-154	protein binding|defense response
GRMZM2G066978	-152	No Description
GRMZM2G130173	-117	structural constituent of ribosome|intracellular|ribosome|translation|homoiothermy|metal ion binding|ice binding|response to freezing

GRMZM5G844094	-138	zinc ion binding
AC203768.3_FG005	-153	metalloendopeptidase activity|proteolysis|membrane
GRMZM2G027253	-199	DNA binding|regulation of transcription, DNA-dependent
GRMZM2G119865	-9	DNA binding|transcription factor activity|structural constituent of ribosome|intracellular|ribosome|regulation of transcription, DNA-dependent|translation
GRMZM2G169333	-146	nucleic acid binding|damaged DNA binding|DNA-(apurinic or apyrimidinic site) lyase activity|DNA repair|base-excision repair|nucleotide-excision repair|zinc ion binding|oxidized purine base lesion DNA N-glycosylase activity|hydrolase activity, hydrolyzing N-glycosyl
GRMZM2G076370	-156	electron carrier activity
GRMZM2G406413	-140	DNA binding|DNA-directed RNA polymerase activity|nucleus|transcription
GRMZM2G095404	-63	peroxidase activity|response to oxidative stress|heme binding|oxidation reduction
AC187885.3_FG002	-72	No Description
GRMZM2G127080	-181	No Description
GRMZM2G061492	-30	cysteine-type endopeptidase activity|protein binding|proteolysis
GRMZM2G358491	-90	No Description
GRMZM2G378167	-60	No Description
GRMZM2G047607	-190	No Description
GRMZM2G322220	-196	transporter activity|oligopeptide transport|membrane
GRMZM2G070075	-141	No Description
GRMZM2G371058	-152	DNA binding|zinc ion binding|regulation of transcription
GRMZM5G804893	-123	DNA binding|intracellular|nucleus|regulation of transcription, DNA-dependent|sequence-specific DNA binding
GRMZM2G039173	-63	membrane
AC226373.2_FG009	-124	No Description
GRMZM2G375904	-14	protein kinase activity|protein serine/threonine kinase activity|ATP binding|protein amino acid phosphorylation
GRMZM2G440452	-73	type I hypersensitivity
GRMZM2G036837	-125	nucleic acid binding|zinc ion binding
GRMZM2G017285	-171	catalytic activity|3-beta-hydroxy-delta5-steroid dehydrogenase activity|steroid biosynthetic process|biosynthetic process|cellular metabolic process|coenzyme binding
GRMZM2G056145	-181	nucleic acid binding|zinc ion binding|homoiothermy|ice binding|response to freezing
GRMZM2G473485	-76	No Description
GRMZM2G111898	-99	No Description
GRMZM2G450228	-197	No Description
GRMZM2G107556	-193	No Description
GRMZM2G477221	-186	binding
GRMZM2G035395	-182	binding
GRMZM2G389510	-10	No Description
GRMZM2G372985	-183	No Description
GRMZM5G860590	-26	No Description
GRMZM2G154881	-82	hydrolase activity
GRMZM2G078723	-21	No Description
GRMZM2G142919	-132	tumor necrosis factor receptor binding|immune response|membrane|transmembrane transport
GRMZM2G334105	-22	No Description
GRMZM2G447005	-22	No Description
GRMZM2G700569	-18	No Description
GRMZM2G349074	-95	No Description
GRMZM2G303168	-19	No Description
GRMZM5G856391	-190	No Description
GRMZM2G139574	-152	transferase activity, transferring glycosyl groups
GRMZM2G162868	-151	No Description
GRMZM2G479869	-95	No Description
GRMZM2G375222	-171	transcription elongation regulator activity|membrane|positive regulation of RNA elongation from RNA polymerase II promoter|protein homooligomerization
GRMZM2G141873	-17	No Description
GRMZM2G456835	-189	response to stress
GRMZM2G100146	-197	ossification|intracellular|cell adhesion|zinc ion binding
GRMZM2G361066	-26	nucleotide binding|ATP-dependent peptidase activity|serine-type endopeptidase activity|ATP binding|proteolysis|membrane|ATPase activity|nucleoside-triphosphatase activity
GRMZM2G063672	-121	nucleotide binding|ATP binding|nucleoside-triphosphatase activity
GRMZM2G014066	-167	No Description
GRMZM2G307756	-75	No Description
GRMZM5G873546	-88	No Description
GRMZM2G029292	-189	transcription factor activity|sequence-specific DNA binding|regulation of transcription
GRMZM5G806622	-108	No Description
GRMZM2G173868	-198	mannosyl-oligosaccharide 1,2-alpha-mannosidase activity|calcium ion binding|membrane
GRMZM2G076450	-82	protein binding
GRMZM2G116273	-22	No Description
GRMZM2G110714	-136	catalytic activity|tricarboxylic acid cycle|phosphoenolpyruvate carboxylase activity|carbon fixation
GRMZM2G420772	-184	No Description
GRMZM2G555108	-16	No Description
GRMZM2G012031	-88	protein binding
GRMZM2G107408	-163	No Description
GRMZM2G344323	-161	No Description
GRMZM5G884407	-18	No Description
GRMZM2G410033	-189	amino acid transport|membrane
GRMZM5G874483	-165	No Description
GRMZM2G396540	-17	No Description
GRMZM2G125175	-185	No Description
GRMZM2G109299	-158	No Description
GRMZM2G355631	-102	No Description
GRMZM2G024615	-66	catalytic activity|membrane
GRMZM2G315431	-63	ubiquitin ligase complex|ubiquitin-protein ligase activity|binding|protein ubiquitination
GRMZM2G414813	-30	transporter activity|transport|membrane|transmembrane transport
GRMZM2G339214	-135	No Description
GRMZM2G146673	-17	protein folding|heat shock protein binding|unfolded protein binding
GRMZM2G307588	-131	DNA binding|nucleus
AC225790.2_FG003	-86	structural molecule activity|cell adhesion|actin cytoskeleton|homoiothermy|ice binding|response to freezing
GRMZM2G435015	-164	nucleus|transcription regulator activity|regulation of transcription
AC212732.4_FG005	-175	No Description
AC191264.3_FG003	-122	No Description
GRMZM2G150683	-17	extracellular region|iron ion transport|cellular iron ion homeostasis|ferric iron binding
GRMZM2G434696	-180	No Description
GRMZM2G702283	-41	No Description

GRMZM2G443939	-33	protein binding|ATP binding|apoptosis|defense response
GRMZM2G003625	-52	protein binding|ATP binding|apoptosis|defense response
GRMZM2G163550	-184	protein kinase activity|ATP binding|sugar binding|protein amino acid phosphorylation|recognition of pollen
GRMZM2G571844	-121	binding
AC203316.3_FG003	-108	No Description
GRMZM2G142565	-24	No Description
GRMZM2G420774	-14	nucleic acid binding
GRMZM2G123644	-141	DNA binding|RNA binding
GRMZM2G109142	-154	No Description
AC203570.4_FG005	-186	No Description
GRMZM2G481009	-28	No Description
GRMZM2G133233	-81	No Description
GRMZM2G396049	-54	RNA binding|RNA processing
GRMZM2G396049	-47	RNA binding|RNA processing
GRMZM2G315885	-53	RNA binding|RNA processing
GRMZM2G025243	-188	alcohol dehydrogenase (NAD) activity|metabolic process|2,3-dihydro-2,3-dihydroxybenzoate dehydrogenase activity|enterobactin biosynthetic process|oxidoreductase activity|oxidation reduction
GRMZM5G896983	-18	No Description
GRMZM2G005456	-9	No Description
GRMZM2G059308	-60	monooxygenase activity|iron ion binding|electron carrier activity|heme binding|oxidation reduction
GRMZM2G087806	-88	zinc ion binding
GRMZM2G316827	-196	No Description
GRMZM2G426229	-50	nucleic acid binding|ATP binding
GRMZM2G438722	-178	calcium ion binding|zinc ion binding
GRMZM2G024718	-8	ATP binding
GRMZM2G086934	-180	nucleic acid binding|DNA binding|nucleus|DNA replication
GRMZM2G386998	-36	acid phosphatase activity|hydrolase activity|metal ion binding
GRMZM2G065259	-18	cysteine-type endopeptidase inhibitor activity|hormone activity|structural molecule activity|structural constituent of cell wall|extracellular region|cell adhesion|actin cytoskeleton
GRMZM2G174070	-177	DNA binding|regulation of transcription
GRMZM2G064600	-39	catalytic activity|binding|mitochondrial inner membrane|metabolic process|protein transporter activity|protein transport
GRMZM2G092723	-76	DNA binding|DNA topoisomerase activity|ATP binding|chromosome|DNA topological change
GRMZM2G413337	-35	No Description
GRMZM2G464818	-26	No Description
GRMZM2G148855	-14	No Description
GRMZM2G457118	-181	No Description
GRMZM2G160428	-58	protein kinase activity|protein serine/threonine kinase activity|ATP binding|protein amino acid phosphorylation
GRMZM2G100086	-36	DNA binding|transcription factor activity|structural constituent of ribosome|intracellular|ribosome|transcription initiation|regulation of transcription, DNA-dependent|translation|sigma factor activity|sequence-specific DNA binding
GRMZM2G441827	-108	No Description
GRMZM2G084940	-16	catalytic activity
GRMZM2G180889	-138	L-arabinose metabolic process|alpha-N-arabinofuranosidase activity
GRMZM2G703650	-87	No Description
GRMZM2G119249	-9	shikimate kinase activity|ATP binding
GRMZM2G546081	-9	biosynthetic process
GRMZM2G127739	-88	signal transducer activity|signal transduction|G-protein coupled receptor protein signaling pathway|zinc ion binding|guanyl nucleotide binding
GRMZM2G151236	-107	protein kinase activity|protein serine/threonine kinase activity|ATP binding|protein amino acid phosphorylation
AC208564.3_FG004	-121	transcription factor activity|nucleus|regulation of transcription, DNA-dependent
GRMZM2G003004	-76	No Description
AC206030.4_FG001	-96	drug transmembrane transport|drug transmembrane transporter activity|antiporter activity|membrane|transmembrane transport
GRMZM2G134735	-84	nucleus|transcription regulator activity|regulation of transcription
GRMZM2G117189	-19	No Description
GRMZM2G180575	-83	integral to membrane
GRMZM2G429940	-195	protein kinase activity|protein serine/threonine kinase activity|ATP binding|protein amino acid phosphorylation|signal transduction
GRMZM2G124281	-135	mitochondrial matrix
GRMZM2G078412	-17	RNA binding
GRMZM2G068639	-100	catalytic activity|D-arabinono-1,4-lactone oxidase activity|serine-type carboxypeptidase activity|proteolysis|membrane|oxidoreductase activity|FAD binding|oxidation reduction
GRMZM2G067626	-13	No Description
GRMZM2G113053	-141	No Description
GRMZM2G113053	-131	No Description
GRMZM2G383338	-142	No Description
GRMZM2G039639	-104	No Description
GRMZM2G436429	-145	intracellular protein transport|membrane
GRMZM2G351304	-42	transcription factor activity|signal transducer activity|regulation of transcription, DNA-dependent|signal transduction
GRMZM2G164084	-36	DNA binding|transcription factor activity|structural constituent of ribosome|intracellular|ribosome|transcription initiation|regulation of transcription, DNA-dependent|translation|sigma factor activity|sequence-specific DNA binding
AC197054.3_FG002	-67	No Description
GRMZM2G164088	-33	nucleic acid binding
GRMZM2G070381	-110	No Description
GRMZM2G312091	-39	No Description
GRMZM2G301805	-73	No Description
GRMZM2G018254	-10	No Description
GRMZM5G881498	-129	No Description
GRMZM2G009854	-56	No Description
GRMZM2G118534	-145	No Description
GRMZM2G023152	-30	serine-type endopeptidase inhibitor activity|methyltransferase activity|O-methyltransferase activity|protein dimerization activity
GRMZM2G165137	-50	nucleic acid binding|ATP binding|cytoplasm|metabolic process|kinase activity|phosphotransferase activity, alcohol group as acceptor
GRMZM2G026724	-117	protein binding
GRMZM2G123257	-53	No Description
GRMZM2G059325	-47	No Description
GRMZM2G113252	-94	No Description
AC185305.3_FG003	-186	No Description
GRMZM2G070422	-42	metabolic process|oxidoreductase activity|oxidation reduction
GRMZM5G822573	-57	No Description
GRMZM2G112228	-13	integral to membrane|vesicle-mediated transport
GRMZM2G045573	-16	No Description
GRMZM2G106308	-76	DNA binding|binding
GRMZM2G106308	-31	DNA binding|binding
GRMZM2G161731	-12	No Description
GRMZM2G070343	-25	glycerol ether metabolic process|electron carrier activity|protein disulfide oxidoreductase activity|cell redox homeostasis

GRMZM2G150706	-161	type I hypersensitivity|homoiothermy|ice binding|response to freezing
GRMZM2G164854	-116	No Description
GRMZM2G475867	-28	No Description
GRMZM2G181359	-20	nucleotide binding|ATP binding|cytoplasm|hydrolase activity|nucleoside-triphosphatase activity|protein catabolic process
GRMZM2G433528	-68	hydrolase activity, hydrolyzing O-glycosyl compounds|carbohydrate metabolic process
GRMZM2G005452	-94	ATP binding|apoptosis|defense response
GRMZM2G363437	-157	nucleus
GRMZM2G305446	-45	transporter activity|transport|membrane|integral to membrane|transmembrane transport
GRMZM2G123480	-105	No Description
GRMZM2G701459	-126	No Description
GRMZM2G341747	-16	calmodulin binding|nucleus|transcription regulator activity|regulation of transcription
GRMZM5G829728	-43	No Description
GRMZM2G169699	-119	No Description
GRMZM2G030013	-60	RNA binding
GRMZM2G406165	-17	No Description
GRMZM2G340762	-168	homoserine kinase activity|ATP binding|threonine metabolic process
GRMZM2G105494	-66	nucleus|transcription regulator activity|regulation of transcription
GRMZM2G021110	-49	aminopeptidase activity|cellular process|manganese ion binding
GRMZM2G136143	-66	binding|homoiothermy|ice binding|response to freezing
GRMZM2G412136	-167	monooxygenase activity|iron ion binding|G-protein coupled receptor protein signaling pathway|electron carrier activity|integral to membrane|heme binding
GRMZM2G124577	-77	No Description
GRMZM2G081955	-25	No Description
GRMZM2G702112	-129	No Description
GRMZM2G064695	-37	catalytic activity|metabolic process
GRMZM2G130167	-97	nucleic acid binding|protein binding|zinc ion binding
GRMZM2G046244	-93	No Description
GRMZM2G140739	-34	structural constituent of ribosome|intracellular|ribosome|translation
GRMZM2G020040	-77	ATP binding|G-protein coupled receptor protein signaling pathway|integral to membrane
GRMZM2G020799	-162	transcription factor activity|nucleus|regulation of transcription, DNA-dependent|sequence-specific DNA binding|protein dimerization activity
GRMZM2G357443	-107	exocyst|exocytosis
GRMZM2G105180	-46	No Description
GRMZM2G380582	-63	No Description
GRMZM2G009413	-14	No Description
GRMZM2G159161	-10	signal transducer activity|protein binding|nitrogen fixation|response to light stimulus|nitrogenase activity
GRMZM2G115598	-38	translation initiation factor activity|eukaryotic translation initiation factor 3 complex|translational initiation
GRMZM5G851753	-43	No Description
GRMZM2G478701	-192	No Description
GRMZM5G831795	-97	metabolic process|hydrolase activity
GRMZM2G041876	-82	nucleic acid binding|DNA binding
GRMZM2G089596	-182	nucleus|transcription regulator activity|regulation of transcription
GRMZM2G057113	-50	No Description
GRMZM2G130230	-48	glucose-6-phosphate dehydrogenase activity|glucose metabolic process|NADP or NADPH binding|oxidation reduction
GRMZM2G094792	-61	No Description
GRMZM2G112039	-94	copper-exporting ATPase activity|transporter activity|ATP binding|ATP biosynthetic process|oligopeptide transport|membrane|metal ion transport|metal ion transmembrane transporter activity
GRMZM2G085260	-43	No Description
GRMZM2G306376	-23	No Description
GRMZM2G143242	-17	homoiothermy|ice binding|response to freezing
GRMZM5G891932	-85	No Description
GRMZM2G090122	-64	No Description
GRMZM2G172695	-63	No Description
GRMZM2G108686	-64	No Description
AC206611.3_FG003	-133	No Description
GRMZM2G430607	-151	catalytic activity|trehalose biosynthetic process
GRMZM2G091588	-79	monooxygenase activity|iron ion binding|electron carrier activity|heme binding|oxidation reduction
GRMZM2G459854	-101	protein kinase activity|protein serine/threonine kinase activity|ATP binding|protein amino acid phosphorylation|metabolic process|amino acid binding|homoiothermy|ice binding|response to freezing
GRMZM2G146745	-35	cysteine-type endopeptidase activity|proteolysis|cysteine-type peptidase activity
GRMZM5G836167	-115	No Description
GRMZM2G046011	-49	structural constituent of ribosome|intracellular|ribosome|translation
GRMZM2G102903	-29	No Description
GRMZM2G357683	-10	nucleotide binding|ATP-dependent peptidase activity|serine-type endopeptidase activity|proteolysis|nucleoside-triphosphatase activity
GRMZM2G094955	-93	tetracycline:hydrogen antiporter activity|tetracycline transport|membrane|integral to membrane|homoiothermy|response to antibiotic|ice binding|response to freezing
GRMZM2G091956	-142	lipid metabolic process|metabolic process
GRMZM2G034271	-142	lipid metabolic process|metabolic process
GRMZM2G003182	-36	DNA binding|transcription factor activity|structural constituent of ribosome|intracellular|ribosome|transcription initiation|regulation of transcription, DNA-dependent|translation|sigma factor activity|sequence-specific DNA binding
GRMZM5G822237	-187	No Description
GRMZM2G398758	-67	DNA binding
GRMZM2G082974	-63	RNA binding|translation elongation factor activity|translational frameshifting|peptidyl-lysine modification to hypusine|ribosome binding|positive regulation of translational elongation|positive regulation of translational termination
GRMZM2G082974	-58	RNA binding|translation elongation factor activity|translational frameshifting|peptidyl-lysine modification to hypusine|ribosome binding|positive regulation of translational elongation|positive regulation of translational termination
GRMZM2G082974	-24	RNA binding|translation elongation factor activity|translational frameshifting|peptidyl-lysine modification to hypusine|ribosome binding|positive regulation of translational elongation|positive regulation of translational termination
GRMZM2G026783	-17	No Description
GRMZM2G701337	-21	No Description
GRMZM2G048305	-25	No Description
GRMZM5G879193	-79	No Description
GRMZM2G396773	-50	No Description
GRMZM2G173280	-106	No Description
GRMZM2G701767	-17	No Description
GRMZM2G136288	-176	ATP binding
GRMZM2G155464	-141	No Description
GRMZM2G127949	-16	No Description
GRMZM2G374030	-151	No Description
GRMZM2G320013	-172	No Description
GRMZM2G324886	-28	No Description
GRMZM2G324886	-23	No Description
GRMZM2G530440	-51	No Description
GRMZM2G098305	-23	single-stranded DNA binding
GRMZM2G122028	-11	No Description

GRMZM5G888620	-10	metabolic process|transferase activity, transferring hexosyl groups
AC198725.4_FG009	-106	transcription factor activity|sequence-specific DNA binding|regulation of transcription
GRMZM2G041015	-77	No Description
GRMZM2G090619	-51	No Description
GRMZM2G126603	-168	structural constituent of ribosome|intracellular|ribosome|translation
GRMZM2G100911	-9	protein kinase activity|protein serine/threonine kinase activity|protein binding|ATP binding|protein amino acid phosphorylation
GRMZM2G108118	-101	No Description
GRMZM2G152688	-8	protein binding|membrane|vesicle-mediated transport
GRMZM5G840946	-62	aspartic-type endopeptidase activity|proteolysis|integral to membrane
GRMZM2G334181	-81	No Description
AC213846.4_FG003	-138	homoiothermy|ice binding|response to freezing
GRMZM2G012273	-17	No Description
GRMZM2G068672	-37	DNA binding|transcription factor activity|nucleus|regulation of transcription, DNA-dependent|transcription regulator activity|homoiothermy|sequence-specific DNA binding|ice binding|response to freezing
GRMZM2G082874	-70	No Description
GRMZM2G100976	-21	iron ion binding|iron-sulfur cluster assembly|iron-sulfur cluster binding
AC209050.3_FG001	-88	catalytic activity|ATP binding|ATP biosynthetic process|cation transport|metabolic process|ATPase activity, coupled to transmembrane movement of ions, phosphorylative mechanism|membrane|integral to membrane|hydrolase activity, acting on acid anhydrides, catalyzing transmembrane movement of substances|ATPase activity
GRMZM2G144362	-158	No Description
GRMZM2G076318	-79	lipid transport|lipid binding
GRMZM2G076370	-40	electron carrier activity
GRMZM2G137108	-62	transporter activity|transport|membrane|integral to membrane|transmembrane transport
GRMZM5G847615	-21	No Description
GRMZM2G126582	-190	transporter activity|transport|membrane|integral to membrane|transmembrane transport
GRMZM2G024073	-48	cell redox homeostasis
GRMZM2G162702	-109	protein kinase activity|protein serine/threonine kinase activity|ATP binding|protein amino acid phosphorylation
GRMZM2G031210	-72	No Description
AC215201.3_FG003	-141	protein kinase activity|protein serine/threonine kinase activity|MAP kinase activity|ATP binding|protein amino acid phosphorylation
GRMZM2G133082	-49	isopentenyl-diphosphate delta-isomerase activity|isoprenoid biosynthetic process|hydrolase activity
GRMZM2G152051	-9	No Description
GRMZM2G077498	-190	transporter activity|oligopeptide transport|membrane|integral to membrane
GRMZM2G125976	-9	transcription factor activity|nucleus|regulation of transcription, DNA-dependent|transcription regulator activity|sequence-specific DNA binding
GRMZM2G701201	-88	protein kinase activity|protein serine/threonine kinase activity|iron ion binding|protein binding|ATP binding|protein amino acid phosphorylation|electron carrier activity|heme binding
GRMZM2G163459	-187	No Description
GRMZM2G163809	-137	isoprenoid biosynthetic process|cyanelle|photosynthesis|transferase activity, transferring alkyl or aryl (other than methyl) groups
GRMZM2G159028	-67	nucleic acid binding
GRMZM2G096812	-140	nucleotide binding|ATP binding|nucleobase, nucleoside, nucleotide and nucleic acid metabolic process|nucleoside-triphosphatase activity|nucleobase, nucleoside, nucleotide kinase activity
GRMZM2G107556	-196	No Description
GRMZM2G080588	-10	protein kinase activity|protein serine/threonine kinase activity|ATP binding|protein amino acid phosphorylation
GRMZM2G097878	-80	protein kinase activity|protein serine/threonine kinase activity|ATP binding|protein amino acid phosphorylation
GRMZM2G097141	-112	No Description
GRMZM2G035395	-70	binding
GRMZM2G023840	-29	peroxidase activity|response to oxidative stress|heme binding|oxidation reduction
GRMZM2G060467	-45	structural constituent of ribosome|intracellular|ribosome|translation
GRMZM2G001991	-23	catalytic activity|3-beta-hydroxy-delta5-steroid dehydrogenase activity|steroid biosynthetic process|metabolic process|oxidoreductase activity|cellular metabolic process|coenzyme binding
AC187235.3_FG003	-21	No Description
GRMZM2G128809	-9	nucleic acid binding|RNA binding
AC199363.4_FG003	-40	No Description
GRMZM2G449496	-67	No Description
GRMZM5G820808	-187	No Description
GRMZM2G415157	-130	No Description
GRMZM2G055684	-144	serine-type endopeptidase activity|proteolysis|identical protein binding|negative regulation of catalytic activity
AC233864.1_FG014	-24	No Description
GRMZM2G005710	-23	No Description
GRMZM2G028986	-10	heme binding
AC203855.2_FG001	-158	No Description
GRMZM2G013726	-61	catalytic activity|3-beta-hydroxy-delta5-steroid dehydrogenase activity|steroid biosynthetic process|metabolic process|oxidoreductase activity|cellular metabolic process|coenzyme binding
GRMZM2G134717	-194	DNA binding|regulation of transcription
GRMZM5G832994	-65	No Description
GRMZM2G301853	-10	No Description
GRMZM2G066024	-54	fructose-bisphosphate aldolase activity|glycolysis
GRMZM2G066024	-41	fructose-bisphosphate aldolase activity|glycolysis
GRMZM2G088828	-24	No Description
GRMZM2G112799	-147	intracellular|zinc ion binding
GRMZM2G069215	-30	ubiquitin ligase complex|ubiquitin-protein ligase activity|protein binding|zinc ion binding|protein ubiquitination
GRMZM2G139396	-30	No Description
GRMZM2G074479	-74	small conjugating protein ligase activity|post-translational protein modification|regulation of protein metabolic process
GRMZM5G830839	-111	serine-type endopeptidase activity|homoserine kinase activity|ATP binding|proteolysis|threonine metabolic process|identical protein binding|negative regulation of catalytic activity
GRMZM2G346701	-55	nucleic acid binding
AC235546.1_FG001	-139	structural molecule activity|cell adhesion|actin cytoskeleton
GRMZM2G093276	-12	zinc ion transmembrane transporter activity|zinc ion transport|membrane|integral to membrane|metal ion transport|metal ion transmembrane transporter activity|transmembrane transport
GRMZM2G158188	-68	No Description
GRMZM2G704448	-85	No Description
GRMZM2G146331	-19	DNA binding|DNA-directed RNA polymerase activity|transcription
GRMZM2G146331	-15	DNA binding|DNA-directed RNA polymerase activity|transcription
GRMZM2G146331	-11	DNA binding|DNA-directed RNA polymerase activity|transcription
GRMZM2G354615	-149	No Description
GRMZM2G040945	-174	serine-type peptidase activity|integral to membrane
GRMZM2G158636	-9	No Description
GRMZM2G011463	-157	No Description
GRMZM2G071869	-8	No Description
GRMZM2G397260	-10	No Description
GRMZM2G044822	-29	No Description
GRMZM2G006416	-60	catalytic activity|protein serine/threonine phosphatase activity|protein amino acid dephosphorylation|protein serine/threonine phosphatase complex
GRMZM2G180027	-38	No Description
GRMZM5G827186	-62	aspartic-type endopeptidase activity|proteolysis|integral to membrane
AC203986.3_FG013	-110	No Description

GRMZM2G000718	-11	No Description
GRMZM2G180205	-131	No Description
GRMZM2G180693	-79	lipid transport|lipid binding
GRMZM2G303536	-183	electron carrier activity|protein disulfide oxidoreductase activity|cell redox homeostasis
GRMZM2G401561	-193	nuclear mRNA splicing, via spliceosome|intracellular|nucleus|RNA processing
GRMZM2G074489	-58	catalytic activity
GRMZM2G084496	-32	magnesium ion binding|metabolic process|lyase activity
GRMZM2G063363	-73	structural molecule activity
GRMZM2G016254	-32	magnesium ion binding|metabolic process|lyase activity
GRMZM2G310453	-54	No Description
GRMZM2G155836	-153	protein kinase activity|protein serine/threonine kinase activity|ATP binding|protein amino acid phosphorylation
GRMZM5G863097	-60	potassium ion transport|metabolic process|cation transmembrane transporter activity|oxidoreductase activity, acting on the CH-OH group of donors, NAD or NADP as acceptor|cofactor binding|NAD or NADH binding
GRMZM2G174773	-60	potassium ion transport|metabolic process|cation transmembrane transporter activity|oxidoreductase activity, acting on the CH-OH group of donors, NAD or NADP as acceptor|cofactor binding|NAD or NADH binding
GRMZM2G126586	-111	membrane
GRMZM2G153456	-15	No Description
GRMZM2G414225	-51	No Description
GRMZM2G058573	-79	chromatin silencing|protein amino acid deacetylation|zinc ion binding|hydrolase activity, acting on carbon-nitrogen (but not peptide) bonds, in linear amides|regulation of transcription|NAD binding
GRMZM2G070111	-146	nucleosome|DNA binding|nucleosome assembly
GRMZM2G164743	-62	transmembrane transport
GRMZM2G065176	-122	No Description
GRMZM2G085236	-131	nucleotide binding|signal transducer activity|ATP binding|transport|G-protein coupled receptor protein signaling pathway|integral to membrane|ATPase activity|nucleoside-triphosphatase activity|guanyl nucleotide binding|ATPase activity, coupled to transmembrane movement of substances|transmembrane transport
GRMZM2G066469	-60	proteolysis|serine-type peptidase activity|membrane
GRMZM2G011513	-70	No Description
GRMZM2G011070	-78	protein kinase activity|protein serine/threonine kinase activity|ATP binding|protein amino acid phosphorylation

y Table S5 (Continued) - Motif 4

Gene ID	Coordinate motif start	Annotation
GRMZM2G465046	-180	lipid metabolic process|hydrolase activity, acting on ester bonds
GRMZM2G465046	-176	lipid metabolic process|hydrolase activity, acting on ester bonds
GRMZM2G077696	-75	No Description
GRMZM2G434839	-101	protein folding|heat shock protein binding|unfolded protein binding
GRMZM5G869587	-147	No Description
GRMZM5G820788	-146	No Description
GRMZM2G095441	-6	SNAP receptor activity|protein binding|intracellular protein transport|membrane|vesicle-mediated transport
GRMZM2G035749	-182	polysaccharide catabolic process|structural constituent of ribosome|intracellular|ribosome|translation|beta-amylase activity
GRMZM2G371030	-108	No Description
GRMZM2G178278	-79	vesicle-mediated transport
GRMZM2G348039	-197	No Description
GRMZM2G113057	-172	No Description
GRMZM2G055899	-57	catalytic activity|branched-chain-amino-acid transaminase activity|metabolic process|branched chain family amino acid metabolic process
GRMZM2G472236	-44	homoiothermy|ice binding|response to freezing
GRMZM2G432566	-25	protein binding|intracellular|zinc ion binding
GRMZM5G890241	-93	protein binding
GRMZM2G153536	-6	catalytic activity|branched-chain-amino-acid transaminase activity|metabolic process|branched chain family amino acid metabolic process
AC196161.3_FG002	-150	No Description
GRMZM2G142390	-120	protein kinase activity|protein serine/threonine kinase activity|ATP binding|protein amino acid phosphorylation
GRMZM2G142390	-116	protein kinase activity|protein serine/threonine kinase activity|ATP binding|protein amino acid phosphorylation
GRMZM2G143703	-139	No Description
GRMZM2G058630	-178	phosphatidylcholine-sterol O-acyltransferase activity|lipid metabolic process
GRMZM2G315862	-157	No Description
GRMZM2G459540	-11	intracellular|zinc ion binding
GRMZM2G050273	-67	No Description
GRMZM2G368827	-178	membrane
AC206030.4_FG001	-147	drug transmembrane transport|drug transmembrane transporter activity|antiporter activity|membrane|transmembrane transport
GRMZM2G352381	-67	No Description
GRMZM2G356718	-169	DNA binding|catalytic activity
GRMZM2G176630	-137	sugar binding|protein amino acid glycosylation|galactosyltransferase activity|membrane|homoiothermy|ice binding|response to freezing
GRMZM2G085303	-19	No Description
GRMZM2G312061	-20	cysteine-type endopeptidase inhibitor activity
GRMZM2G394783	-121	monooxygenase activity|iron ion binding|electron carrier activity|heme binding|oxidation reduction
AC191692.3_FG001	-136	No Description
GRMZM2G115775	-121	protein binding|membrane|vesicle-mediated transport
AC217947.4_FG002	-100	iron ion binding|FMN binding|oxidoreductase activity|oxidation reduction
GRMZM2G459746	-7	nucleic acid binding
GRMZM2G146207	-105	No Description
GRMZM2G129979	-154	nucleus
GRMZM2G325575	-47	cellular iron ion homeostasis|ferric iron binding|oxidoreductase activity|transition metal ion binding|oxidation reduction
GRMZM2G109680	-142	No Description
GRMZM2G163277	-124	No Description
GRMZM2G071745	-58	heme binding
GRMZM2G031996	-87	No Description
GRMZM2G011383	-183	No Description
GRMZM2G166719	-195	protein kinase activity|protein serine/threonine kinase activity|ATP binding|protein amino acid phosphorylation
GRMZM5G894340	-197	No Description
GRMZM2G044348	-20	aspartic-type endopeptidase activity|integral to membrane
GRMZM2G018403	-95	RNA binding|structural constituent of ribosome|intracellular|ribosome|translation
GRMZM2G366434	-191	DNA binding|transcription factor activity|regulation of transcription, DNA-dependent
GRMZM2G074604	-170	catalytic activity|cytoplasm|L-phenylalanine catabolic process|biosynthetic process|ammonia-lyase activity
GRMZM2G075366	-131	No Description
GRMZM2G038279	-62	chromatin|DNA binding|nucleus|regulation of transcription, DNA-dependent
GRMZM2G104920	-193	ubiquitin ligase complex|protein phosphatase type 2A complex|ubiquitin-protein ligase activity|protein binding|signal transduction|zinc ion binding|protein phosphatase type 2A regulator activity|protein ubiquitination|homoiothermy|ice binding|response to freezing
GRMZM5G861978	-163	structural constituent of ribosome|intracellular|ribosome|translation

GRMZM2G402797	-65	No Description
AC197340.3_FG004	-43	No Description
GRMZM2G422147	-45	No Description
GRMZM2G106650	-116	monooxygenase activity|iron ion binding|electron carrier activity|heme binding|oxidation reduction
GRMZM2G176129	-173	oxidoreductase activity, acting on NADH or NADPH, quinone or similar compound as acceptor|oxidation reduction
GRMZM2G026095	-27	metabolic process|zinc ion binding|hydrolase activity
GRMZM2G004188	-189	DNA binding|catalytic activity|nuclease activity|DNA repair
GRMZM2G163297	-171	nucleic acid binding|RNA binding
GRMZM2G146292	-170	No Description
AC202534.2_FG007	-191	No Description
GRMZM2G106604	-89	No Description
GRMZM2G021051	-165	iron ion binding|oxidoreductase activity|oxidation reduction
GRMZM2G398758	-66	DNA binding
GRMZM2G152370	-118	No Description
GRMZM2G021243	-9	No Description
GRMZM2G428905	-176	No Description
GRMZM2G336879	-19	ribonucleoside-diphosphate reductase activity|ribonucleoside-diphosphate reductase complex|DNA replication
GRMZM2G074373	-19	DNA binding|transcription factor activity|nucleus|transcription, DNA-dependent|regulation of transcription, DNA-dependent|sequence-specific DNA binding|regulation of transcription|protein dimerization activity
GRMZM2G005753	-103	protein folding|heat shock protein binding|unfolded protein binding
GRMZM2G016668	-102	protein binding|zinc ion binding
GRMZM2G018022	-99	metabolic process|nucleotidyltransferase activity
GRMZM2G135095	-103	structural constituent of ribosome|intracellular|ribosome|translation
GRMZM2G125216	-59	No Description
AC209810.3_FG002	-22	cysteine-type endopeptidase activity|proteolysis|cysteine-type peptidase activity
GRMZM2G013016	-107	No Description
GRMZM2G113319	-176	nucleotide binding|motor activity|ATP binding|myosin complex|nucleoside-triphosphatase activity
AC155377.1_FG001	-131	nucleotide binding|motor activity|ATP binding|myosin complex|nucleoside-triphosphatase activity
GRMZM2G094490	-114	nucleotide binding|ATP-dependent peptidase activity|serine-type endopeptidase activity|ATP binding|proteolysis|membrane|ATPase activity|nucleoside-triphosphatase activity
GRMZM2G552418	-128	No Description
GRMZM2G100568	-189	No Description
GRMZM2G005622	-11	No Description
GRMZM2G027253	-197	DNA binding|regulation of transcription, DNA-dependent
GRMZM2G147245	-35	monooxygenase activity|iron ion binding|electron carrier activity|heme binding|oxidation reduction
GRMZM2G063961	-199	protein kinase activity|protein serine/threonine kinase activity|ATP binding|protein amino acid phosphorylation
GRMZM2G082037	-13	metabolic process|transferase activity, transferring hexosyl groups
GRMZM2G381709	-32	small conjugating protein ligase activity|post-translational protein modification|regulation of protein metabolic process
GRMZM2G036837	-125	nucleic acid binding|zinc ion binding
GRMZM2G024674	-136	No Description
GRMZM2G109627	-10	DNA binding|regulation of transcription
AC211474.3_FG006	-132	No Description
AC211474.3_FG006	-78	No Description
GRMZM2G074824	-78	catalytic activity
GRMZM2G099052	-131	No Description
GRMZM2G168675	-38	No Description
GRMZM2G347471	-197	No Description
GRMZM2G047412	-134	No Description
GRMZM2G028986	-10	heme binding
GRMZM2G361066	-24	nucleotide binding|ATP-dependent peptidase activity|serine-type endopeptidase activity|ATP binding|proteolysis|membrane|ATPase activity|nucleoside-triphosphatase activity
GRMZM2G025031	-172	uroporphyrinogen decarboxylase activity|porphyrin biosynthetic process
GRMZM2G169584	-24	ATP binding|apoptosis
GRMZM2G047214	-112	No Description
GRMZM2G074462	-199	catalytic activity|carbohydrate metabolic process|carbohydrate binding
GRMZM2G085934	-119	No Description
GRMZM2G170420	-181	No Description
GRMZM2G125448	-195	transferase activity, transferring acyl groups other than amino-acyl groups
GRMZM2G041388	-178	No Description
GRMZM2G325008	-122	hydrolase activity, hydrolyzing O-glycosyl compounds|carbohydrate metabolic process|cellular process
GRMZM2G083555	-195	No Description
GRMZM2G027851	-173	voltage-gated potassium channel activity|cation transport|potassium ion transport|sodium ion transport|regulation of pH|voltage-gated potassium channel complex|solute:hydrogen antiporter activity|sodium:hydrogen antiporter activity|integral to membrane|transmembrane transport
GRMZM5G838435	-45	catalytic activity
GRMZM2G115579	-51	No Description
AC203972.3_FG001	-59	structural constituent of ribosome|ATP binding|intracellular|ribosome|translation|apoptosis|defense response
GRMZM2G088140	-51	DNA binding|transcription factor activity|nucleus|transcription, DNA-dependent|regulation of transcription, DNA-dependent|sequence-specific DNA binding|regulation of transcription|protein dimerization activity
GRMZM2G405170	-198	DNA binding|regulation of transcription, DNA-dependent
GRMZM2G043749	-160	No Description
GRMZM2G314593	-99	No Description
GRMZM2G110145	-163	membrane|cellulose synthase (UDP-forming) activity|cellulose biosynthetic process
GRMZM2G021277	-123	cellular amino acid and derivative metabolic process|carboxy-lyase activity|carboxylic acid metabolic process|pyridoxal phosphate binding
GRMZM2G062541	-88	nucleus|transcription regulator activity|regulation of transcription
GRMZM2G062541	-84	nucleus|transcription regulator activity|regulation of transcription
GRMZM2G415579	-103	oxidoreductase activity|oxidation reduction
GRMZM2G027241	-138	No Description
GRMZM2G107299	-22	No Description
GRMZM2G070279	-60	alcohol dehydrogenase (NAD) activity|metabolic process|2,3-dihydro-2,3-dihydroxybenzoate dehydrogenase activity|enterobactin biosynthetic process|oxidoreductase activity|oxidation reduction
GRMZM2G018631	-34	protein binding|zinc ion binding
GRMZM5G871174	-138	No Description
GRMZM2G352381	-67	No Description
GRMZM2G161368	-188	No Description
GRMZM2G169207	-173	No Description
GRMZM5G836606	-5	No Description
GRMZM2G003307	-58	No Description
GRMZM2G064773	-90	protein serine/threonine kinase activity|ATP binding|protein amino acid phosphorylation
GRMZM2G053631	-11	nucleic acid binding|intracellular|nucleobase, nucleoside, nucleotide and nucleic acid metabolic process|3'-5' exonuclease activity
GRMZM2G398758	-66	DNA binding
GRMZM5G899656	-86	catalytic activity|phosphoribosylamine-glycine ligase activity|ATP binding|purine base biosynthetic process

GRMZM2G413044	-16	hydrolase activity, hydrolyzing O-glycosyl compounds|cell wall|carbohydrate metabolic process|cellular glucan metabolic process|xyloglucan:xyloglucosyl transferase activity|apoplast
GRMZM2G082198	-43	nucleic acid binding
GRMZM2G062318	-99	No Description
GRMZM2G402156	-48	DNA binding
AC197699.3_FG008	-21	No Description
GRMZM2G448656	-32	No Description
GRMZM2G083716	-100	protein binding|ATP binding|cytoplasm|protein folding|protein refolding|cellular protein metabolic process
AC187789.3_FG001	-46	No Description
GRMZM2G455687	-158	No Description
GRMZM2G138370	-179	No Description
GRMZM2G134049	-114	No Description
GRMZM2G047867	-55	catalytic activity
GRMZM2G081474	-22	histone deacetylase activity|histone deacetylation|acetoin metabolic process
GRMZM2G305238	-51	No Description
GRMZM2G029690	-186	protein binding|zinc ion binding
GRMZM2G028254	-19	No Description
GRMZM2G451746	-99	No Description
GRMZM2G116452	-176	peroxidase activity|response to oxidative stress|heme binding|oxidation reduction
GRMZM2G704352	-113	No Description
GRMZM2G121360	-179	metal ion transport|metal ion binding
GRMZM5G828123	-18	No Description
GRMZM5G828987	-59	hydrolase activity, hydrolyzing O-glycosyl compounds|carbohydrate metabolic process
GRMZM2G126821	-155	structural constituent of ribosome|intracellular|ribosome|translation|small ribosomal subunit
GRMZM2G358931	-174	structural constituent of ribosome|cyclin-dependent protein kinase inhibitor activity|histamine receptor activity|intracellular|nucleus|ribosome|translation|cell cycle arrest|G-protein coupled receptor protein signaling pathway|integral to membrane
GRMZM5G809743	-135	No Description
GRMZM2G022907	-18	No Description
GRMZM2G139852	-93	No Description
GRMZM2G153536	-8	catalytic activity|branched-chain-amino-acid transaminase activity|metabolic process|branched chain family amino acid metabolic process
GRMZM2G085427	-131	DNA binding
AC231441.1_FG004	-152	No Description
GRMZM2G161337	-105	tryptophan biosynthetic process|anthranilate synthase activity|biosynthetic process|oxo-acid-lyase activity
GRMZM2G345330	-164	No Description
GRMZM2G174284	-66	transcription factor activity|regulation of transcription, DNA-dependent|sequence-specific DNA binding
GRMZM2G013299	-16	glutathione peroxidase activity|response to oxidative stress|oxidation reduction
GRMZM2G552278	-164	No Description
GRMZM5G828488	-17	No Description
GRMZM2G030636	-192	No Description
GRMZM2G089291	-78	iron ion binding|sodium ion transport|regulation of pH|electron carrier activity|sodium:hydrogen antiporter activity|membrane|integral to membrane|oxidoreductase activity|FAD binding|oxidation reduction
GRMZM2G001895	-120	binding|heat shock protein binding
GRMZM5G841743	-48	No Description
GRMZM2G030436	-136	transferase activity, transferring acyl groups other than amino-acyl groups
GRMZM2G123418	-20	No Description
GRMZM2G034680	-59	No Description
GRMZM2G303010	-190	No Description
GRMZM2G119339	-22	No Description
GRMZM2G008250	-116	No Description
GRMZM2G042313	-44	structural constituent of ribosome|intracellular|ribosome|translation|transport|amino acid transport|amino acid transmembrane transporter activity|membrane|transmembrane transport
GRMZM2G093809	-60	protein kinase activity|protein serine/threonine kinase activity|protein binding|ATP binding|protein amino acid phosphorylation
GRMZM2G009117	-190	metal ion transport|metal ion binding
GRMZM2G472671	-90	nucleus|type I hypersensitivity|transcription regulator activity|regulation of transcription
GRMZM2G340305	-56	DNA binding|regulation of transcription
GRMZM2G016439	-106	two-component signal transduction system (phosphorelay)|signal transducer activity
GRMZM2G449163	-112	protein tyrosine phosphatase activity|dephosphorylation|phosphatase activity
GRMZM2G160560	-21	membrane
GRMZM2G564469	-174	No Description
GRMZM2G101105	-41	RNA binding|double-stranded RNA binding|ribonuclease III activity|intracellular|RNA processing
GRMZM2G703910	-101	No Description
GRMZM2G067626	-18	No Description
GRMZM2G131591	-187	protein binding|zinc ion binding
GRMZM2G135866	-189	protein binding|zinc ion binding
GRMZM2G459291	-22	catalytic activity
GRMZM5G830585	-77	No Description
GRMZM2G145412	-112	No Description
GRMZM2G163761	-19	transcription factor activity|catalytic activity|nucleus|regulation of transcription, DNA-dependent|transcription regulator activity|sequence-specific DNA binding|cellular metabolic process
GRMZM2G036505	-151	No Description
GRMZM2G416426	-134	No Description
GRMZM2G174083	-115	motor activity|ATP binding|myosin complex
GRMZM2G053023	-176	cell redox homeostasis
GRMZM2G022662	-147	translation elongation factor activity|translational elongation
GRMZM2G104125	-10	protein kinase activity|protein serine/threonine kinase activity|calcium ion binding|ATP binding|protein amino acid phosphorylation
GRMZM2G347056	-54	methylenetetrahydrofolate reductase (NADPH) activity|methionine metabolic process|oxidation reduction
GRMZM2G016084	-163	RNA binding
GRMZM2G023557	-66	DNA binding
GRMZM2G339848	-151	transcription factor activity|regulation of transcription, DNA-dependent|sequence-specific DNA binding
GRMZM2G140527	-121	copper ion binding|oxidoreductase activity|oxidation reduction
GRMZM2G122498	-180	monooxygenase activity|iron ion binding|electron carrier activity|heme binding|oxidation reduction
AC218998.2_FG001	-160	monooxygenase activity|iron ion binding|electron carrier activity|heme binding|oxidation reduction
GRMZM2G434299	-51	No Description
GRMZM2G011896	-21	protein kinase activity|protein serine/threonine kinase activity|protein binding|ATP binding|protein amino acid phosphorylation
GRMZM2G074293	-28	No Description
GRMZM2G171420	-73	No Description
AC209029.2_FG002	-173	No Description
GRMZM2G702379	-148	No Description
GRMZM2G170829	-98	manganese ion binding|nutrient reservoir activity|apoplast
AC196475.3_FG004	-175	methyltransferase activity|O-methyltransferase activity|protein dimerization activity
GRMZM2G055520	-99	No Description

GRMZM2G354618	-182	nucleus|transcription regulator activity|regulation of transcription
GRMZM2G702419	-190	No Description
GRMZM2G036217	-8	No Description
GRMZM2G425999	-181	potassium ion transport|potassium ion transmembrane transporter activity|membrane
AC205664.3_FG001	-78	No Description
AC191692.3_FG001	-138	No Description
GRMZM2G013214	-131	metabolic process|oxidoreductase activity|homoiothermy|ice binding|response to freezing|oxidation reduction
GRMZM2G168171	-124	No Description
AC205269.3_FG003	-19	No Description
GRMZM2G394945	-49	No Description
GRMZM2G122979	-138	No Description
GRMZM2G135132	-198	6-phosphofructokinase activity|adenosine kinase activity|ribokinase activity|6-phosphofructokinase complex|D-ribose metabolic process|glycolysis|purine ribonucleoside salvage
GRMZM2G129675	-66	No Description
GRMZM2G147780	-70	Rho guanyl-nucleotide exchange factor activity
GRMZM2G076631	-46	catalytic activity
GRMZM2G063276	-9	hydrolase activity
GRMZM2G081843	-163	transporter activity|transport|membrane|integral to membrane|transmembrane transport
GRMZM2G108147	-174	catalytic activity
GRMZM2G008819	-158	copper ion binding|electron carrier activity
GRMZM2G138949	-61	No Description
GRMZM2G038479	-24	nucleus|transcription regulator activity|regulation of transcription
GRMZM2G406552	-116	No Description
GRMZM2G131957	-21	nucleic acid binding|structural constituent of cell wall
GRMZM2G332505	-198	nucleosome|DNA binding|nucleus|nucleosome assembly|amino acid transport|membrane
GRMZM2G051541	-55	No Description
GRMZM2G049839	-138	aspartic-type endopeptidase activity|proteolysis
GRMZM2G336761	-30	protein binding|zinc ion binding
GRMZM2G143445	-159	No Description
GRMZM2G010152	-108	DNA binding|catalytic activity|DNA topoisomerase (ATP-hydrolyzing) activity|copper-exporting ATPase activity|copper ion binding|ATP binding|chromosome|DNA topological change|ATP biosynthetic process|copper ion transport|metabolic process|mercury ion transmembrane transporter activity|ATPase activity, coupled to transmembrane movement of ions, phosphorylative mechanism|mercury ion transport|membrane|integral to membrane|hydrolase activity, acting on acid anhydrides, catalyzing transmembrane movement of substances|metal ion transport|metal ion binding|metal ion transmembrane transporter activity
GRMZM2G088258	-11	oxidoreductase activity
GRMZM2G168888	-195	thiosulfate sulfurtransferase activity|sulfate transport
GRMZM2G119640	-8	nucleic acid binding|zinc ion binding
GRMZM5G862317	-196	lipid metabolic process|hydrolase activity, acting on ester bonds
AC190636.3_FG005	-116	oxidoreductase activity
GRMZM2G133481	-56	catalytic activity
GRMZM2G002765	-164	catalytic activity
GRMZM2G002765	-161	catalytic activity
GRMZM2G110881	-119	catalytic activity|3-beta-hydroxy-delta5-steroid dehydrogenase activity|steroid biosynthetic process|metabolic process|oxidoreductase activity|cellular metabolic process|coenzyme binding
GRMZM5G810054	-55	No Description
GRMZM2G701300	-41	No Description
GRMZM2G171953	-103	No Description
GRMZM2G077295	-28	protein binding|intracellular protein transport|ER to Golgi vesicle-mediated transport|zinc ion binding|COPII vesicle coat
GRMZM2G019958	-196	No Description
GRMZM2G160664	-20	DNA binding|ATP binding|nucleus|DNA replication|DNA-dependent DNA replication initiation
GRMZM2G102601	-131	two-component sensor activity|ATP binding|signal transduction|membrane|phosphorylation|transferase activity, transferring phosphorus-containing groups
AC194402.2_FG004	-110	No Description
GRMZM2G018943	-166	cellular metabolic process
GRMZM2G388576	-116	No Description
GRMZM2G403218	-17	No Description
GRMZM2G162544	-16	SNAP receptor activity|protein binding|intracellular protein transport|membrane|vesicle-mediated transport
GRMZM2G462081	-14	No Description
GRMZM2G701448	-177	DNA binding|DNA topoisomerase (ATP-hydrolyzing) activity|ATP binding|chromosome|DNA topological change
GRMZM2G123097	-186	No Description
GRMZM2G054745	-191	structural molecule activity|viral capsid|heat shock protein binding
GRMZM2G102447	-37	No Description
AC185261.4_FG002	-12	No Description
GRMZM2G109070	-154	No Description
GRMZM2G023798	-19	catalytic activity|intramolecular transferase activity
GRMZM2G041878	-127	No Description
GRMZM2G105331	-176	No Description
GRMZM2G138423	-53	transporter activity|binding|mitochondrial inner membrane|transport|membrane|transmembrane transport
GRMZM2G039089	-15	protein folding|heat shock protein binding|unfolded protein binding
GRMZM2G101523	-43	zinc ion binding
GRMZM2G079682	-100	beta-1,4-mannosylglycoprotein 4-beta-N-acetylglucosaminyltransferase activity|protein amino acid N-linked glycosylation|membrane
GRMZM2G128902	-190	No Description
GRMZM5G859330	-33	No Description
GRMZM2G463711	-90	No Description
GRMZM2G702126	-41	No Description
GRMZM2G056762	-40	No Description
GRMZM2G090419	-25	hydrolase activity
GRMZM2G130548	-99	No Description
GRMZM2G072300	-76	heat shock protein binding
GRMZM2G086027	-41	No Description
GRMZM2G119769	-57	homoiothermy|ice binding|response to freezing
GRMZM2G465769	-150	No Description
GRMZM2G017869	-80	lipid metabolic process|hydrolase activity, acting on ester bonds
GRMZM2G385818	-163	chromatin|DNA binding|RNA binding|structural constituent of ribosome|nucleus|regulation of transcription, DNA-dependent|translation
GRMZM2G076212	-22	protein kinase activity|protein serine/threonine kinase activity|ATP binding|protein amino acid phosphorylation
GRMZM2G081886	-182	phosphoglycerate dehydrogenase activity|L-serine biosynthetic process|potassium ion transport|metabolic process|cation transmembrane transporter activity|amino acid binding|oxidoreductase activity, acting on the CH-OH group of donors, NAD or NADP as acceptor|cofactor binding|NAD or NADH binding|oxidation reduction
GRMZM2G055844	-194	protein kinase activity|protein serine/threonine kinase activity|protein binding|ATP binding|protein amino acid phosphorylation
GRMZM2G106010	-99	No Description
GRMZM5G825909	-68	No Description
GRMZM2G039711	-20	No Description

GRMZM2G178709	-64	No Description
GRMZM2G147268	-171	cellular amino acid metabolic process|biosynthetic process|transferase activity, transferring nitrogenous groups|lyase activity|pyridoxal phosphate binding
GRMZM2G457697	-52	No Description
GRMZM2G138770	-104	nucleotide binding|ATP binding|nucleoside-triphosphatase activity
GRMZM2G441748	-118	No Description
GRMZM2G050925	-19	No Description
GRMZM2G147685	-56	nucleus|transcription regulator activity|regulation of transcription
GRMZM2G025042	-66	No Description
GRMZM2G509030	-154	No Description
GRMZM2G013357	-99	monooxygenase activity|iron ion binding|electron carrier activity|heme binding|oxidation reduction
GRMZM2G178199	-32	No Description
GRMZM2G703185	-70	No Description
GRMZM2G145666	-49	structural constituent of cell wall
GRMZM2G703193	-31	No Description
GRMZM2G180691	-188	hydrolase activity
GRMZM2G503606	-138	No Description
GRMZM2G066638	-115	motor activity|ATP binding|myosin complex
GRMZM2G067756	-39	No Description
GRMZM2G146685	-113	No Description
GRMZM2G037574	-8	intracellular|zinc ion binding
GRMZM2G002053	-27	type I hypersensitivity|homoiothermy|ice binding|response to freezing
GRMZM2G015409	-87	protein binding|zinc ion binding
GRMZM2G379563	-118	No Description
GRMZM2G158629	-163	cysteine-type endopeptidase activity|fatty acid synthase activity|fatty acid synthase complex|proteolysis|fatty acid biosynthetic process|metabolic process|oxidoreductase activity|oxidation reduction
GRMZM2G703338	-142	No Description
GRMZM2G016106	-12	nucleic acid binding
AC219035.2_FG006	-136	No Description
GRMZM2G128771	-29	GTPase activity|protein binding|GTP binding|intracellular|intracellular protein transport|nucleocytoplasmic transport|signal transduction|small GTPase mediated signal transduction|protein transport
GRMZM2G350773	-12	No Description
GRMZM2G103881	-131	No Description
GRMZM2G062019	-134	metabolic process|hydrolase activity
GRMZM2G062019	-128	metabolic process|hydrolase activity
GRMZM2G062019	-122	metabolic process|hydrolase activity
GRMZM2G062019	-116	metabolic process|hydrolase activity
GRMZM2G056329	-82	ATP binding
GRMZM2G022763	-137	nucleic acid binding|RNA binding|nucleus|mRNA processing
GRMZM2G001388	-63	protein binding
GRMZM2G398758	-68	DNA binding
GRMZM2G345270	-11	sugar:hydrogen symporter activity|transport|phosphoenolpyruvate-dependent sugar phosphotransferase system
GRMZM2G035482	-61	No Description
GRMZM2G308910	-11	DNA binding|structural constituent of ribosome|ligand-dependent nuclear receptor activity|intracellular|nucleus|ribosome|regulation of transcription, DNA-dependent|translation
GRMZM2G390236	-180	No Description
GRMZM2G336879	-21	ribonucleoside-diphosphate reductase activity|ribonucleoside-diphosphate reductase complex|DNA replication
GRMZM2G336929	-12	transferase activity, transferring glycosyl groups
GRMZM2G013471	-30	No Description
GRMZM2G170307	-31	heat shock protein binding
AC200904.3_FG003	-42	No Description
GRMZM2G013971	-135	ubiquitin ligase complex|ubiquitin-protein ligase activity|protein binding|zinc ion binding|protein ubiquitination
GRMZM2G046924	-40	transferase activity
GRMZM2G010384	-151	No Description
GRMZM2G081912	-151	ubiquitin ligase complex|ubiquitin-protein ligase activity|protein binding|zinc ion binding|protein ubiquitination
GRMZM2G701533	-191	No Description
GRMZM2G038356	-71	GTP binding|intracellular|intracellular protein transport|small GTPase mediated signal transduction
AC205833.3_FG002	-52	No Description
GRMZM2G457411	-39	protein binding|zinc ion binding
GRMZM2G053692	-61	structural molecule activity|protein binding|retrograde vesicle-mediated transport, Golgi to ER|COPI vesicle coat
GRMZM2G138268	-21	nucleus|regulation of transcription
GRMZM2G366142	-91	No Description
GRMZM2G013016	-109	No Description
GRMZM2G053284	-49	protein binding|ATP binding
GRMZM2G177518	-179	No Description
GRMZM2G004314	-184	No Description
GRMZM2G065290	-72	transcription factor activity|sequence-specific DNA binding|regulation of transcription
GRMZM2G021704	-17	dihydroorotase activity|hydrolase activity|hydrolase activity, acting on carbon-nitrogen (but not peptide) bonds, in cyclic amides|pyrimidine base biosynthetic process
GRMZM2G039246	-131	two-component signal transduction system (phosphorelay)|signal transducer activity
GRMZM2G702144	-71	No Description
GRMZM2G159724	-17	malic enzyme activity|malate metabolic process|oxidoreductase activity|oxidoreductase activity, acting on the CH-OH group of donors, NAD or NADP as acceptor|metal ion binding|NAD or NADH binding|oxidation reduction
GRMZM5G814782	-186	No Description
GRMZM5G814803	-24	No Description
AC155377.1_FG002	-55	aspartic-type endopeptidase activity|proteolysis
GRMZM2G481362	-119	hydrolase activity
GRMZM2G467147	-154	protein binding|defense response
GRMZM2G066978	-152	No Description
GRMZM2G130173	-117	structural constituent of ribosome|intracellular|ribosome|translation|homoiothermy|metal ion binding|ice binding|response to freezing
GRMZM5G844094	-138	zinc ion binding
AC203768.3_FG005	-153	metalloendopeptidase activity|proteolysis|membrane
GRMZM2G027253	-199	DNA binding|regulation of transcription, DNA-dependent
GRMZM2G119865	-9	DNA binding|transcription factor activity|structural constituent of ribosome|intracellular|ribosome|regulation of transcription, DNA-dependent|translation
GRMZM2G169333	-146	nucleic acid binding|damaged DNA binding|DNA-(apurinic or apyrimidinic site) lyase activity|DNA repair|base-excision repair|nucleotide-excision repair|zinc ion binding|oxidized purine base lesion DNA N-glycosylase activity|hydrolase activity, hydrolyzing N-glycosyl
GRMZM2G076370	-156	electron carrier activity
GRMZM2G406413	-140	DNA binding|DNA-directed RNA polymerase activity|nucleus|transcription
GRMZM2G095404	-63	peroxidase activity|response to oxidative stress|heme binding|oxidation reduction
AC187885.3_FG002	-72	No Description
GRMZM2G127080	-181	No Description
GRMZM2G061492	-30	cysteine-type endopeptidase activity|protein binding|proteolysis
GRMZM2G358491	-90	No Description

GRMZM2G378167	-60	No Description
GRMZM2G047607	-190	No Description
GRMZM2G322220	-196	transporter activity|oligopeptide transport|membrane
GRMZM2G070075	-141	No Description
GRMZM2G371058	-152	DNA binding|zinc ion binding|regulation of transcription
GRMZM5G804893	-123	DNA binding|intracellular|nucleus|regulation of transcription, DNA-dependent|sequence-specific DNA binding
GRMZM2G039173	-63	membrane
AC226373.2_FG009	-124	No Description
GRMZM2G375904	-14	protein kinase activity|protein serine/threonine kinase activity|ATP binding|protein amino acid phosphorylation
GRMZM2G440452	-73	type I hypersensitivity
GRMZM2G036837	-125	nucleic acid binding|zinc ion binding
GRMZM2G017285	-171	catalytic activity|3-beta-hydroxy-delta5-steroid dehydrogenase activity|steroid biosynthetic process|biosynthetic process|cellular metabolic process|coenzyme binding
GRMZM2G056145	-181	nucleic acid binding|zinc ion binding|homoiothermy|ice binding|response to freezing
GRMZM2G473485	-76	No Description
GRMZM2G111898	-99	No Description
GRMZM2G450228	-197	No Description
GRMZM2G107556	-193	No Description
GRMZM2G477221	-186	binding
GRMZM2G035395	-182	binding
GRMZM2G389510	-10	No Description
GRMZM2G372985	-183	No Description
GRMZM5G860590	-26	No Description
GRMZM2G154881	-82	hydrolase activity
GRMZM2G078723	-21	No Description
GRMZM2G142919	-132	tumor necrosis factor receptor binding|immune response|membrane|transmembrane transport
GRMZM2G334105	-22	No Description
GRMZM2G447005	-22	No Description
GRMZM2G700569	-18	No Description
GRMZM2G349074	-95	No Description
GRMZM2G303168	-19	No Description
GRMZM5G856391	-190	No Description
GRMZM2G139574	-152	transferase activity, transferring glycosyl groups
GRMZM2G162868	-151	No Description
GRMZM2G479869	-95	No Description
GRMZM2G375222	-171	transcription elongation regulator activity|membrane|positive regulation of RNA elongation from RNA polymerase II promoter|protein homooligomerization
GRMZM2G141873	-17	No Description
GRMZM2G456835	-189	response to stress
GRMZM2G100146	-197	ossification|intracellular|cell adhesion|zinc ion binding
GRMZM2G361066	-26	nucleotide binding|ATP-dependent peptidase activity|serine-type endopeptidase activity|ATP binding|proteolysis|membrane|ATPase activity|nucleoside-triphosphatase activity
GRMZM2G063672	-121	nucleotide binding|ATP binding|nucleoside-triphosphatase activity
GRMZM2G014066	-167	No Description
GRMZM2G307756	-75	No Description
GRMZM5G873546	-88	No Description
GRMZM2G029292	-189	transcription factor activity|sequence-specific DNA binding|regulation of transcription
GRMZM5G806622	-108	No Description
GRMZM2G173868	-198	mannosyl-oligosaccharide 1,2-alpha-mannosidase activity|calcium ion binding|membrane
GRMZM2G076450	-82	protein binding
GRMZM2G116273	-22	No Description
GRMZM2G110714	-136	catalytic activity|tricarboxylic acid cycle|phosphoenolpyruvate carboxylase activity|carbon fixation
GRMZM2G420772	-184	No Description
GRMZM2G555108	-16	No Description
GRMZM2G012031	-88	protein binding
GRMZM2G107408	-163	No Description
GRMZM2G344323	-161	No Description
GRMZM5G884407	-18	No Description
GRMZM2G410033	-189	amino acid transport|membrane
GRMZM5G874483	-165	No Description
GRMZM2G396540	-17	No Description
GRMZM2G125175	-185	No Description
GRMZM2G109299	-158	No Description
GRMZM2G355631	-102	No Description
GRMZM2G024615	-66	catalytic activity|membrane
GRMZM2G315431	-63	ubiquitin ligase complex|ubiquitin-protein ligase activity|binding|protein ubiquitination
GRMZM2G414813	-30	transporter activity|transport|membrane|transmembrane transport
GRMZM2G339214	-135	No Description
GRMZM2G146673	-17	protein folding|heat shock protein binding|unfolded protein binding
GRMZM2G307588	-131	DNA binding|nucleus
AC225790.2_FG003	-86	structural molecule activity|cell adhesion|actin cytoskeleton|homoiothermy|ice binding|response to freezing
GRMZM2G435015	-164	nucleus|transcription regulator activity|regulation of transcription
AC212732.4_FG005	-175	No Description
AC191264.3_FG003	-122	No Description
GRMZM2G150683	-17	extracellular region|iron ion transport|cellular iron ion homeostasis|ferric iron binding
GRMZM2G434696	-180	No Description
GRMZM2G702283	-41	No Description
GRMZM2G443939	-33	protein binding|ATP binding|apoptosis|defense response
GRMZM2G003625	-52	protein binding|ATP binding|apoptosis|defense response
GRMZM2G163550	-184	protein kinase activity|ATP binding|sugar binding|protein amino acid phosphorylation|recognition of pollen
GRMZM2G571844	-121	binding
AC203316.3_FG003	-108	No Description
GRMZM2G142565	-24	No Description
GRMZM2G420774	-14	nucleic acid binding
GRMZM2G123644	-141	DNA binding|RNA binding
GRMZM2G109142	-154	No Description
AC203570.4_FG005	-186	No Description
GRMZM2G481009	-28	No Description
GRMZM2G133233	-81	No Description

GRMZM2G396049	-54	RNA binding|RNA processing
GRMZM2G396049	-47	RNA binding|RNA processing
GRMZM2G315885	-53	RNA binding|RNA processing
GRMZM2G025243	-188	alcohol dehydrogenase (NAD) activity|metabolic process|2,3-dihydro-2,3-dihydroxybenzoate dehydrogenase activity|enterobactin biosynthetic process|oxidoreductase activity|oxidation reduction
GRMZM5G896983	-18	No Description

Supplementary Table S5 (Continued) - Motif 5

Gene ID	Coordinate motif start	Annotation
GRMZM2G352381	-67	No Description
GRMZM2G131332	-4	No Description
GRMZM2G111300	-56	zinc ion transmembrane transporter activity|zinc ion transport|membrane|integral to membrane|oxidoreductase activity, acting on paired donors, with incorporation or reduction of molecular oxygen, reduced pteridine as one donor, and incorporation of one atom of oxygen|metal ion transport|metal ion transmembrane transporter activity|transmembrane transport|oxidation reduction
GRMZM2G169207	-173	No Description
GRMZM2G430029	-89	No Description
GRMZM2G012127	-115	No Description
GRMZM2G355940	-43	translation release factor activity|translational termination
GRMZM2G398758	-66	DNA binding
GRMZM2G089343	-13	No Description
GRMZM2G134389	-14	protein tyrosine phosphatase activity|dephosphorylation|phosphatase activity
GRMZM2G028986	-10	heme binding
GRMZM2G027241	-138	No Description
GRMZM2G465046	-180	lipid metabolic process|hydrolase activity, acting on ester bonds
GRMZM2G465046	-176	lipid metabolic process|hydrolase activity, acting on ester bonds
GRMZM2G077696	-75	No Description
GRMZM2G434839	-101	protein folding|heat shock protein binding|unfolded protein binding
GRMZM5G869587	-147	No Description
GRMZM5G820788	-146	No Description
GRMZM2G095441	-6	SNAP receptor activity|protein binding|intracellular protein transport|membrane|vesicle-mediated transport
GRMZM2G035749	-182	polysaccharide catabolic process|structural constituent of ribosome|intracellular|ribosome|translation|beta-amylase activity
GRMZM2G371030	-108	No Description
GRMZM2G178278	-79	vesicle-mediated transport
GRMZM2G348039	-197	No Description
GRMZM2G113057	-172	No Description
GRMZM2G055899	-57	catalytic activity|branched-chain-amino-acid transaminase activity|metabolic process|branched chain family amino acid metabolic process
GRMZM2G472236	-44	homoiothermy|ice binding|response to freezing
GRMZM2G432566	-25	protein binding|intracellular|zinc ion binding
GRMZM5G890241	-93	protein binding
GRMZM2G153536	-6	catalytic activity|branched-chain-amino-acid transaminase activity|metabolic process|branched chain family amino acid metabolic process
AC196161.3_FG002	-150	No Description
GRMZM2G142390	-120	protein kinase activity|protein serine/threonine kinase activity|ATP binding|protein amino acid phosphorylation
GRMZM2G142390	-116	protein kinase activity|protein serine/threonine kinase activity|ATP binding|protein amino acid phosphorylation
GRMZM2G143703	-139	No Description
GRMZM2G058630	-178	phosphatidylcholine-sterol O-acyltransferase activity|lipid metabolic process
GRMZM2G315862	-157	No Description
GRMZM2G459540	-11	intracellular|zinc ion binding
GRMZM2G050273	-67	No Description
GRMZM2G368827	-178	membrane
AC206030.4_FG001	-147	drug transmembrane transport|drug transmembrane transporter activity|antiporter activity|membrane|transmembrane transport
GRMZM2G352381	-67	No Description
GRMZM2G356718	-169	DNA binding|catalytic activity
GRMZM2G176630	-137	sugar binding|protein amino acid glycosylation|galactosyltransferase activity|membrane|homoiothermy|ice binding|response to freezing
GRMZM2G085303	-19	No Description
GRMZM2G312061	-20	cysteine-type endopeptidase inhibitor activity
GRMZM2G394783	-121	monooxygenase activity|iron ion binding|electron carrier activity|heme binding|oxidation reduction
AC191692.3_FG001	-136	No Description
GRMZM2G115775	-121	protein binding|membrane|vesicle-mediated transport
AC217947.4_FG002	-100	iron ion binding|FMN binding|oxidoreductase activity|oxidation reduction
GRMZM2G459746	-7	nucleic acid binding
GRMZM2G146207	-105	No Description
GRMZM2G129979	-154	nucleus
GRMZM2G325575	-47	cellular iron ion homeostasis|ferric iron binding|oxidoreductase activity|transition metal ion binding|oxidation reduction
GRMZM2G109680	-142	No Description
GRMZM2G163277	-124	No Description
GRMZM2G071745	-58	heme binding
GRMZM2G031996	-87	No Description
GRMZM2G011383	-183	No Description
GRMZM2G166719	-195	protein kinase activity|protein serine/threonine kinase activity|ATP binding|protein amino acid phosphorylation
GRMZM5G894340	-197	No Description
GRMZM2G044348	-20	aspartic-type endopeptidase activity|integral to membrane
GRMZM2G018403	-95	RNA binding|structural constituent of ribosome|intracellular|ribosome|translation
GRMZM2G366434	-191	DNA binding|transcription factor activity|regulation of transcription, DNA-dependent
GRMZM2G074604	-170	catalytic activity|cytoplasm|L-phenylalanine catabolic process|biosynthetic process|ammonia-lyase activity
GRMZM2G075366	-131	No Description
GRMZM2G038279	-62	chromatin|DNA binding|nucleus|regulation of transcription, DNA-dependent
GRMZM2G104920	-193	ubiquitin ligase complex|protein phosphatase type 2A complex|ubiquitin-protein ligase activity|protein binding|signal transduction|zinc ion binding|protein phosphatase type 2A regulator activity|protein ubiquitination|homoiothermy|ice binding|response to freezing
GRMZM5G861978	-163	structural constituent of ribosome|intracellular|ribosome|translation
GRMZM2G402797	-65	No Description
AC197340.3_FG004	-43	No Description
GRMZM2G422147	-45	No Description
GRMZM2G106650	-116	monooxygenase activity|iron ion binding|electron carrier activity|heme binding|oxidation reduction
GRMZM2G176129	-173	oxidoreductase activity, acting on NADH or NADPH, quinone or similar compound as acceptor|oxidation reduction
GRMZM2G026095	-27	metabolic process|zinc ion binding|hydrolase activity
GRMZM2G004188	-189	DNA binding|catalytic activity|nuclease activity|DNA repair

GRMZM2G163297	-171	nucleic acid binding|RNA binding
GRMZM2G146292	-170	No Description
AC202534.2_FG007	-191	No Description
GRMZM2G106604	-89	No Description
GRMZM2G021051	-165	iron ion binding|oxidoreductase activity|oxidation reduction
GRMZM2G398758	-66	DNA binding
GRMZM2G152370	-118	No Description
GRMZM2G021243	-9	No Description
GRMZM2G428905	-176	No Description
GRMZM2G336879	-19	ribonucleoside-diphosphate reductase activity|ribonucleoside-diphosphate reductase complex|DNA replication
GRMZM2G074373	-19	DNA binding|transcription factor activity|nucleus|transcription, DNA-dependent|regulation of transcription, DNA-dependent|sequence-specific DNA binding|regulation of transcription|protein dimerization activity
GRMZM2G005753	-103	protein folding|heat shock protein binding|unfolded protein binding
GRMZM2G016668	-102	protein binding|zinc ion binding
GRMZM2G018022	-99	metabolic process|nucleotidyltransferase activity
GRMZM2G135095	-103	structural constituent of ribosome|intracellular|ribosome|translation
GRMZM2G125216	-59	No Description
AC209810.3_FG002	-22	cysteine-type endopeptidase activity|proteolysis|cysteine-type peptidase activity
GRMZM2G013016	-107	No Description
GRMZM2G113319	-176	nucleotide binding|motor activity|ATP binding|myosin complex|nucleoside-triphosphatase activity
AC155377.1_FG001	-131	nucleotide binding|motor activity|ATP binding|myosin complex|nucleoside-triphosphatase activity
GRMZM2G094490	-114	nucleotide binding|ATP-dependent peptidase activity|serine-type endopeptidase activity|ATP binding|proteolysis|membrane|ATPase activity|nucleoside-triphosphatase activity
GRMZM2G552418	-128	No Description
GRMZM2G100568	-189	No Description
GRMZM2G005622	-11	No Description
GRMZM2G027253	-197	DNA binding|regulation of transcription, DNA-dependent
GRMZM2G147245	-35	monooxygenase activity|iron ion binding|electron carrier activity|heme binding|oxidation reduction
GRMZM2G063961	-199	protein kinase activity|protein serine/threonine kinase activity|ATP binding|protein amino acid phosphorylation
GRMZM2G082037	-13	metabolic process|transferase activity, transferring hexosyl groups
GRMZM2G381709	-32	small conjugating protein ligase activity|post-translational protein modification|regulation of protein metabolic process
GRMZM2G036837	-125	nucleic acid binding|zinc ion binding
GRMZM2G024674	-136	No Description
GRMZM2G109627	-10	DNA binding|regulation of transcription
AC211474.3_FG006	-132	No Description
AC211474.3_FG006	-78	No Description
GRMZM2G074824	-78	catalytic activity
GRMZM2G099052	-131	No Description
GRMZM2G168675	-38	No Description
GRMZM2G347471	-197	No Description
GRMZM2G047412	-134	No Description
GRMZM2G028986	-10	heme binding
GRMZM2G361066	-24	nucleotide binding|ATP-dependent peptidase activity|serine-type endopeptidase activity|ATP binding|proteolysis|membrane|ATPase activity|nucleoside-triphosphatase activity
GRMZM2G025031	-172	uroporphyrinogen decarboxylase activity|porphyrin biosynthetic process
GRMZM2G169584	-24	ATP binding|apoptosis
GRMZM2G047214	-112	No Description
GRMZM2G074462	-199	catalytic activity|carbohydrate metabolic process|carbohydrate binding
GRMZM2G085934	-119	No Description
GRMZM2G170420	-181	No Description
GRMZM2G125448	-195	transferase activity, transferring acyl groups other than amino-acyl groups
GRMZM2G041388	-178	No Description
GRMZM2G325008	-122	hydrolase activity, hydrolyzing O-glycosyl compounds|carbohydrate metabolic process|cellular process
GRMZM2G083555	-195	No Description
GRMZM2G027851	-173	voltage-gated potassium channel activity|cation transport|potassium ion transport|sodium ion transport|regulation of pH|voltage-gated potassium channel complex|solute:hydrogen antiporter activity|sodium:hydrogen antiporter activity|integral to membrane|transmembrane transport
GRMZM5G838435	-45	catalytic activity
GRMZM2G115579	-51	No Description
AC203972.3_FG001	-59	structural constituent of ribosome|ATP binding|intracellular|ribosome|translation|apoptosis|defense response
GRMZM2G088140	-51	DNA binding|transcription factor activity|nucleus|transcription, DNA-dependent|regulation of transcription, DNA-dependent|sequence-specific DNA binding|regulation of transcription|protein dimerization activity
GRMZM2G405170	-198	DNA binding|regulation of transcription, DNA-dependent
GRMZM2G043749	-160	No Description
GRMZM2G314593	-99	No Description
GRMZM2G110145	-163	membrane|cellulose synthase (UDP-forming) activity|cellulose biosynthetic process
GRMZM2G021277	-123	cellular amino acid and derivative metabolic process|carboxy-lyase activity|carboxylic acid metabolic process|pyridoxal phosphate binding
GRMZM2G062541	-88	nucleus|transcription regulator activity|regulation of transcription
GRMZM2G062541	-84	nucleus|transcription regulator activity|regulation of transcription
GRMZM2G415579	-103	oxidoreductase activity|oxidation reduction
GRMZM2G027241	-138	No Description
GRMZM2G107299	-22	No Description
GRMZM2G070279	-60	alcohol dehydrogenase (NAD) activity|metabolic process|2,3-dihydro-2,3-dihydroxybenzoate dehydrogenase activity|enterobactin biosynthetic process|oxidoreductase activity|oxidation reduction
GRMZM2G018631	-34	protein binding|zinc ion binding
GRMZM5G871174	-138	No Description
GRMZM2G352381	-67	No Description
GRMZM2G161368	-188	No Description
GRMZM2G169207	-173	No Description
GRMZM5G836606	-5	No Description
GRMZM2G003307	-58	No Description
GRMZM2G064773	-90	protein serine/threonine kinase activity|ATP binding|protein amino acid phosphorylation
GRMZM2G053631	-11	nucleic acid binding|intracellular|nucleobase, nucleoside, nucleotide and nucleic acid metabolic process|3'-5' exonuclease activity
GRMZM2G398758	-66	DNA binding
GRMZM5G899656	-86	catalytic activity|phosphoribosylamine-glycine ligase activity|ATP binding|purine base biosynthetic process
GRMZM2G413044	-16	hydrolase activity, hydrolyzing O-glycosyl compounds|cell wall|carbohydrate metabolic process|cellular glucan metabolic process|xyloglucan:xyloglucosyl transferase activity|apoplast
GRMZM2G082198	-43	nucleic acid binding
GRMZM2G062318	-99	No Description
GRMZM2G402156	-48	DNA binding
AC197699.3_FG008	-21	No Description
GRMZM2G448656	-32	No Description
GRMZM2G083716	-100	protein binding|ATP binding|cytoplasm|protein folding|protein refolding|cellular protein metabolic process

AC187789.3_FG001	-46	No Description
GRMZM2G455687	-158	No Description
GRMZM2G138370	-179	No Description
GRMZM2G134049	-114	No Description
GRMZM2G047867	-55	catalytic activity
GRMZM2G081474	-22	histone deacetylase activity|histone deacetylation|acetoin metabolic process
GRMZM2G305238	-51	No Description
GRMZM2G029690	-186	protein binding|zinc ion binding
GRMZM2G028254	-19	No Description
GRMZM2G451746	-99	No Description
GRMZM2G116452	-176	peroxidase activity|response to oxidative stress|heme binding|oxidation reduction
GRMZM2G704352	-113	No Description
GRMZM2G121360	-179	metal ion transport|metal ion binding
GRMZM5G828123	-18	No Description
GRMZM5G828987	-59	hydrolase activity, hydrolyzing O-glycosyl compounds|carbohydrate metabolic process
GRMZM2G126821	-155	structural constituent of ribosome|intracellular|ribosome|translation|small ribosomal subunit
GRMZM2G358931	-174	structural constituent of ribosome|cyclin-dependent protein kinase inhibitor activity|histamine receptor activity|intracellular|nucleus|ribosome|translation|cell cycle arrest|G-protein coupled receptor protein signaling pathway|integral to membrane
GRMZM5G809743	-135	No Description
GRMZM2G022907	-18	No Description
GRMZM2G139852	-93	No Description
GRMZM2G153536	-8	catalytic activity|branched-chain-amino-acid transaminase activity|metabolic process|branched chain family amino acid metabolic process
GRMZM2G085427	-131	DNA binding
AC231441.1_FG004	-152	No Description
GRMZM2G161337	-105	tryptophan biosynthetic process|anthranilate synthase activity|biosynthetic process|oxo-acid-lyase activity
GRMZM2G345330	-164	No Description
GRMZM2G174284	-66	transcription factor activity|regulation of transcription, DNA-dependent|sequence-specific DNA binding
GRMZM2G013299	-16	glutathione peroxidase activity|response to oxidative stress|oxidation reduction
GRMZM2G552278	-164	No Description
GRMZM5G828488	-17	No Description
GRMZM2G030636	-192	No Description
GRMZM2G089291	-78	iron ion binding|sodium ion transport|regulation of pH|electron carrier activity|sodium:hydrogen antiporter activity|membrane|integral to membrane|oxidoreductase activity|FAD binding|oxidation reduction
GRMZM2G001895	-120	binding|heat shock protein binding
GRMZM5G841743	-48	No Description
GRMZM2G030436	-136	transferase activity, transferring acyl groups other than amino-acyl groups
GRMZM2G123418	-20	No Description
GRMZM2G034680	-59	No Description
GRMZM2G303010	-190	No Description
GRMZM2G119339	-22	No Description
GRMZM2G008250	-116	No Description
GRMZM2G042313	-44	structural constituent of ribosome|intracellular|ribosome|translation|transport|amino acid transport|amino acid transmembrane transporter activity|membrane|transmembrane transport
GRMZM2G093809	-60	protein kinase activity|protein serine/threonine kinase activity|protein binding|ATP binding|protein amino acid phosphorylation
GRMZM2G009117	-190	metal ion transport|metal ion binding
GRMZM2G472671	-90	nucleus|type I hypersensitivity|transcription regulator activity|regulation of transcription
GRMZM2G340305	-56	DNA binding|regulation of transcription
GRMZM2G016439	-106	two-component signal transduction system (phosphorelay)|signal transducer activity
GRMZM2G449163	-112	protein tyrosine phosphatase activity|dephosphorylation|phosphatase activity
GRMZM2G160560	-21	membrane
GRMZM2G564469	-174	No Description
GRMZM2G101105	-41	RNA binding|double-stranded RNA binding|ribonuclease III activity|intracellular|RNA processing
GRMZM2G703910	-101	No Description
GRMZM2G067626	-18	No Description
GRMZM2G131591	-187	protein binding|zinc ion binding
GRMZM2G135866	-189	protein binding|zinc ion binding
GRMZM2G459291	-22	catalytic activity
GRMZM5G830585	-77	No Description
GRMZM2G145412	-112	No Description
GRMZM2G163761	-19	transcription factor activity|catalytic activity|nucleus|regulation of transcription, DNA-dependent|transcription regulator activity|sequence-specific DNA binding|cellular metabolic process
GRMZM2G036505	-151	No Description
GRMZM2G416426	-134	No Description
GRMZM2G174083	-115	motor activity|ATP binding|myosin complex
GRMZM2G053023	-176	cell redox homeostasis
GRMZM2G022662	-147	translation elongation factor activity|translational elongation
GRMZM2G104125	-10	protein kinase activity|protein serine/threonine kinase activity|calcium ion binding|ATP binding|protein amino acid phosphorylation
GRMZM2G347056	-54	methylenetetrahydrofolate reductase (NADPH) activity|methionine metabolic process|oxidation reduction
GRMZM2G016084	-163	RNA binding
GRMZM2G023557	-66	DNA binding
GRMZM2G339848	-151	transcription factor activity|regulation of transcription, DNA-dependent|sequence-specific DNA binding
GRMZM2G140527	-121	copper ion binding|oxidoreductase activity|oxidation reduction
GRMZM2G122498	-180	monooxygenase activity|iron ion binding|electron carrier activity|heme binding|oxidation reduction
AC218998.2_FG001	-160	monooxygenase activity|iron ion binding|electron carrier activity|heme binding|oxidation reduction
GRMZM2G434299	-51	No Description
GRMZM2G011896	-21	protein kinase activity|protein serine/threonine kinase activity|protein binding|ATP binding|protein amino acid phosphorylation
GRMZM2G074293	-28	No Description
GRMZM2G171420	-73	No Description
AC209029.2_FG002	-173	No Description
GRMZM2G702379	-148	No Description
GRMZM2G170829	-98	manganese ion binding|nutrient reservoir activity|apoplast
AC196475.3_FG004	-175	methyltransferase activity|O-methyltransferase activity|protein dimerization activity
GRMZM2G055520	-99	No Description
GRMZM2G354618	-182	nucleus|transcription regulator activity|regulation of transcription
GRMZM2G702419	-190	No Description
GRMZM2G036217	-8	No Description
GRMZM2G425999	-181	potassium ion transport|potassium ion transmembrane transporter activity|membrane
AC205664.3_FG001	-78	No Description
AC191692.3_FG001	-138	No Description
GRMZM2G013214	-131	metabolic process|oxidoreductase activity|homoiothermy|ice binding|response to freezing|oxidation reduction

GRMZM2G168171	-124	No Description
AC205269.3_FG003	-19	No Description
GRMZM2G394945	-49	No Description
GRMZM2G122979	-138	No Description
GRMZM2G135132	-198	6-phosphofructokinase activity|adenosine kinase activity|ribokinase activity|6-phosphofructokinase complex|D-ribose metabolic process|glycolysis|purine ribonucleoside salvage
GRMZM2G129675	-66	No Description
GRMZM2G147780	-70	Rho guanyl-nucleotide exchange factor activity
GRMZM2G076631	-46	catalytic activity
GRMZM2G063276	-9	hydrolase activity
GRMZM2G081843	-163	transporter activity|transport|membrane|integral to membrane|transmembrane transport
GRMZM2G108147	-174	catalytic activity
GRMZM2G008819	-158	copper ion binding|electron carrier activity
GRMZM2G138949	-61	No Description
GRMZM2G038479	-24	nucleus|transcription regulator activity|regulation of transcription
GRMZM2G406552	-116	No Description
GRMZM2G131957	-21	nucleic acid binding|structural constituent of cell wall
GRMZM2G332505	-198	nucleosome|DNA binding|nucleus|nucleosome assembly|amino acid transport|membrane
GRMZM2G051541	-55	No Description
GRMZM2G049839	-138	aspartic-type endopeptidase activity|proteolysis
GRMZM2G336761	-30	protein binding|zinc ion binding
GRMZM2G143445	-159	No Description
GRMZM2G010152	-108	DNA binding|catalytic activity|DNA topoisomerase (ATP-hydrolyzing) activity|copper-exporting ATPase activity|copper ion binding|ATP binding|chromosome|DNA topological change|ATP biosynthetic process|copper ion transport|metabolic process|mercury ion transmembrane transporter activity|ATPase activity, coupled to transmembrane movement of ions, phosphorylative mechanism|mercury ion transport|membrane|integral to membrane|hydrolase activity, acting on acid anhydrides, catalyzing transmembrane movement of substances|metal ion transport|metal ion binding|metal ion transmembrane transporter activity
GRMZM2G088258	-11	oxidoreductase activity
GRMZM2G168888	-195	thiosulfate sulfurtransferase activity|sulfate transport
GRMZM2G119640	-8	nucleic acid binding|zinc ion binding
GRMZM5G862317	-196	lipid metabolic process|hydrolase activity, acting on ester bonds
AC190636.3_FG005	-116	oxidoreductase activity
GRMZM2G133481	-56	catalytic activity
GRMZM2G002765	-164	catalytic activity
GRMZM2G002765	-161	catalytic activity
GRMZM2G110881	-119	catalytic activity|3-beta-hydroxy-delta5-steroid dehydrogenase activity|steroid biosynthetic process|metabolic process|oxidoreductase activity|cellular metabolic process|coenzyme binding
GRMZM5G810054	-55	No Description
GRMZM2G701300	-41	No Description
GRMZM2G171953	-103	No Description
GRMZM2G077295	-28	protein binding|intracellular protein transport|ER to Golgi vesicle-mediated transport|zinc ion binding|COPII vesicle coat
GRMZM2G019958	-196	No Description
GRMZM2G160664	-20	DNA binding|ATP binding|nucleus|DNA replication|DNA-dependent DNA replication initiation
GRMZM2G102601	-131	two-component sensor activity|ATP binding|signal transduction|membrane|phosphorylation|transferase activity, transferring phosphorus-containing groups
AC194402.2_FG004	-110	No Description
GRMZM2G018943	-166	cellular metabolic process
GRMZM2G388576	-116	No Description
GRMZM2G403218	-17	No Description
GRMZM2G162544	-16	SNAP receptor activity|protein binding|intracellular protein transport|membrane|vesicle-mediated transport
GRMZM2G462081	-14	No Description
GRMZM2G701448	-177	DNA binding|DNA topoisomerase (ATP-hydrolyzing) activity|ATP binding|chromosome|DNA topological change
GRMZM2G123097	-186	No Description
GRMZM2G054745	-191	structural molecule activity|viral capsid|heat shock protein binding
GRMZM2G102447	-37	No Description
AC185261.4_FG002	-12	No Description
GRMZM2G109070	-154	No Description
GRMZM2G023798	-19	catalytic activity|intramolecular transferase activity
GRMZM2G041878	-127	No Description
GRMZM2G105331	-176	No Description
GRMZM2G138423	-53	transporter activity|binding|mitochondrial inner membrane|transport|membrane|transmembrane transport
GRMZM2G039089	-15	protein folding|heat shock protein binding|unfolded protein binding
GRMZM2G101523	-43	zinc ion binding
GRMZM2G079682	-100	beta-1,4-mannosylglycoprotein 4-beta-N-acetylglucosaminyltransferase activity|protein amino acid N-linked glycosylation|membrane
GRMZM2G128902	-190	No Description
GRMZM5G859330	-33	No Description
GRMZM2G463711	-90	No Description
GRMZM2G702126	-41	No Description
GRMZM2G056762	-40	No Description
GRMZM2G090419	-25	hydrolase activity
GRMZM2G130548	-99	No Description
GRMZM2G072300	-76	heat shock protein binding
GRMZM2G086027	-41	No Description
GRMZM2G119769	-57	homoiothermy|ice binding|response to freezing
GRMZM2G465769	-150	No Description
GRMZM2G017869	-80	lipid metabolic process|hydrolase activity, acting on ester bonds
GRMZM2G385818	-163	chromatin|DNA binding|RNA binding|structural constituent of ribosome|nucleus|regulation of transcription, DNA-dependent|translation
GRMZM2G076212	-22	protein kinase activity|protein serine/threonine kinase activity|ATP binding|protein amino acid phosphorylation
GRMZM2G081886	-182	phosphoglycerate dehydrogenase activity|L-serine biosynthetic process|potassium ion transport|metabolic process|cation transmembrane transporter activity|amino acid binding|oxidoreductase activity, acting on the CH-OH group of donors, NAD or NADP as acceptor|cofactor binding|NAD or NADH binding|oxidation reduction
GRMZM2G055844	-194	protein kinase activity|protein serine/threonine kinase activity|protein binding|ATP binding|protein amino acid phosphorylation
GRMZM2G106010	-99	No Description
GRMZM5G825909	-68	No Description
GRMZM2G039711	-20	No Description
GRMZM2G178709	-64	No Description
GRMZM2G147268	-171	cellular amino acid metabolic process|biosynthetic process|transferase activity, transferring nitrogenous groups|lyase activity|pyridoxal phosphate binding
GRMZM2G457697	-52	No Description
GRMZM2G138770	-104	nucleotide binding|ATP binding|nucleoside-triphosphatase activity
GRMZM2G441748	-118	No Description
GRMZM2G050925	-19	No Description
GRMZM2G147685	-56	nucleus|transcription regulator activity|regulation of transcription

GRMZM2G025042	-66	No Description
GRMZM2G509030	-154	No Description
GRMZM2G013357	-99	monooxygenase activity|iron ion binding|electron carrier activity|heme binding|oxidation reduction
GRMZM2G178199	-32	No Description
GRMZM2G703185	-70	No Description
GRMZM2G145666	-49	structural constituent of cell wall
GRMZM2G703193	-31	No Description
GRMZM2G180691	-188	hydrolase activity
GRMZM2G503606	-138	No Description
GRMZM2G066638	-115	motor activity|ATP binding|myosin complex
GRMZM2G067756	-39	No Description
GRMZM2G146685	-113	No Description
GRMZM2G037574	-8	intracellular|zinc ion binding
GRMZM2G002053	-27	type I hypersensitivity|homoiothermy|ice binding|response to freezing
GRMZM2G015409	-87	protein binding|zinc ion binding
GRMZM2G379563	-118	No Description
GRMZM2G158629	-163	cysteine-type endopeptidase activity|fatty acid synthase activity|fatty acid synthase complex|proteolysis|fatty acid biosynthetic process|metabolic process|oxidoreductase activity|oxidation reduction
GRMZM2G703338	-142	No Description
GRMZM2G016106	-12	nucleic acid binding
AC219035.2_FG006	-136	No Description
GRMZM2G128771	-29	GTPase activity|protein binding|GTP binding|intracellular|intracellular protein transport|nucleocytoplasmic transport|signal transduction|small GTPase mediated signal transduction|protein transport
GRMZM2G350773	-12	No Description
GRMZM2G103881	-131	No Description
GRMZM2G062019	-134	metabolic process|hydrolase activity
GRMZM2G062019	-128	metabolic process|hydrolase activity
GRMZM2G062019	-122	metabolic process|hydrolase activity
GRMZM2G062019	-116	metabolic process|hydrolase activity
GRMZM2G056329	-82	ATP binding
GRMZM2G022763	-137	nucleic acid binding|RNA binding|nucleus|mRNA processing
GRMZM2G001388	-63	protein binding
GRMZM2G398758	-68	DNA binding
GRMZM2G345270	-11	sugar:hydrogen symporter activity|transport|phosphoenolpyruvate-dependent sugar phosphotransferase system
GRMZM2G035482	-61	No Description
GRMZM2G308910	-11	DNA binding|structural constituent of ribosome|ligand-dependent nuclear receptor activity|intracellular|nucleus|ribosome|regulation of transcription, DNA-dependent|translation
GRMZM2G390236	-180	No Description
GRMZM2G336879	-21	ribonucleoside-diphosphate reductase activity|ribonucleoside-diphosphate reductase complex|DNA replication
GRMZM2G336929	-12	transferase activity, transferring glycosyl groups
GRMZM2G013471	-30	No Description
GRMZM2G170307	-31	heat shock protein binding
AC200904.3_FG003	-42	No Description
GRMZM2G013971	-135	ubiquitin ligase complex|ubiquitin-protein ligase activity|protein binding|zinc ion binding|protein ubiquitination
GRMZM2G046924	-40	transferase activity
GRMZM2G010384	-151	No Description
GRMZM2G081912	-151	ubiquitin ligase complex|ubiquitin-protein ligase activity|protein binding|zinc ion binding|protein ubiquitination
GRMZM2G701533	-191	No Description
GRMZM2G038356	-71	GTP binding|intracellular|intracellular protein transport|small GTPase mediated signal transduction
AC205833.3_FG002	-52	No Description
GRMZM2G457411	-39	protein binding|zinc ion binding
GRMZM2G053692	-61	structural molecule activity|protein binding|retrograde vesicle-mediated transport, Golgi to ER|COPI vesicle coat
GRMZM2G138268	-21	nucleus|regulation of transcription
GRMZM2G366142	-91	No Description
GRMZM2G013016	-109	No Description
GRMZM2G053284	-49	protein binding|ATP binding
GRMZM2G177518	-179	No Description
GRMZM2G004314	-184	No Description
GRMZM2G065290	-72	transcription factor activity|sequence-specific DNA binding|regulation of transcription
GRMZM2G021704	-17	dihydroorotase activity|hydrolase activity|hydrolase activity, acting on carbon-nitrogen (but not peptide) bonds, in cyclic amides|pyrimidine base biosynthetic process
GRMZM2G039246	-131	two-component signal transduction system (phosphorelay)|signal transducer activity
GRMZM2G702144	-71	No Description
GRMZM2G159724	-17	malic enzyme activity|malate metabolic process|oxidoreductase activity|oxidoreductase activity, acting on the CH-OH group of donors, NAD or NADP as acceptor|metal ion binding|NAD or NADH binding|oxidation reduction
GRMZM5G814782	-186	No Description
GRMZM5G814803	-24	No Description
AC155377.1_FG002	-55	aspartic-type endopeptidase activity|proteolysis
GRMZM2G481362	-119	hydrolase activity
GRMZM2G467147	-154	protein binding|defense response
GRMZM2G066978	-152	No Description
GRMZM2G130173	-117	structural constituent of ribosome|intracellular|ribosome|translation|homoiothermy|metal ion binding|ice binding|response to freezing
GRMZM5G844094	-138	zinc ion binding
AC203768.3_FG005	-153	metalloendopeptidase activity|proteolysis|membrane
GRMZM2G027253	-199	DNA binding|regulation of transcription, DNA-dependent
GRMZM2G119865	-9	DNA binding|transcription factor activity|structural constituent of ribosome|intracellular|ribosome|regulation of transcription, DNA-dependent|translation
GRMZM2G169333	-146	nucleic acid binding|damaged DNA binding|DNA-(apurinic or apyrimidinic site) lyase activity|DNA repair|base-excision repair|nucleotide-excision repair|zinc ion binding|oxidized purine base lesion DNA N-glycosylase activity|hydrolase activity, hydrolyzing N-glycosyl
GRMZM2G076370	-156	electron carrier activity
GRMZM2G406413	-140	DNA binding|DNA-directed RNA polymerase activity|nucleus|transcription
GRMZM2G095404	-63	peroxidase activity|response to oxidative stress|heme binding|oxidation reduction
AC187885.3_FG002	-72	No Description
GRMZM2G127080	-181	No Description
GRMZM2G061492	-30	cysteine-type endopeptidase activity|protein binding|proteolysis
GRMZM2G358491	-90	No Description
GRMZM2G378167	-60	No Description
GRMZM2G047607	-190	No Description
GRMZM2G322220	-196	transporter activity|oligopeptide transport|membrane
GRMZM2G070075	-141	No Description
GRMZM2G371058	-152	DNA binding|zinc ion binding|regulation of transcription
GRMZM5G804893	-123	DNA binding|intracellular|nucleus|regulation of transcription, DNA-dependent|sequence-specific DNA binding
GRMZM2G039173	-63	membrane

AC226373.2_FG009	-124	No Description
GRMZM2G375904	-14	protein kinase activity|protein serine/threonine kinase activity|ATP binding|protein amino acid phosphorylation
GRMZM2G440452	-73	type I hypersensitivity
GRMZM2G036837	-125	nucleic acid binding|zinc ion binding
GRMZM2G017285	-171	catalytic activity|3-beta-hydroxy-delta5-steroid dehydrogenase activity|steroid biosynthetic process|biosynthetic process|cellular metabolic process|coenzyme binding
GRMZM2G056145	-181	nucleic acid binding|zinc ion binding|homoiothermy|ice binding|response to freezing
GRMZM2G473485	-76	No Description
GRMZM2G111898	-99	No Description
GRMZM2G450228	-197	No Description
GRMZM2G107556	-193	No Description
GRMZM2G477221	-186	binding
GRMZM2G035395	-182	binding
GRMZM2G389510	-10	No Description
GRMZM2G372985	-183	No Description
GRMZM5G860590	-26	No Description
GRMZM2G154881	-82	hydrolase activity
GRMZM2G078723	-21	No Description
GRMZM2G142919	-132	tumor necrosis factor receptor binding|immune response|membrane|transmembrane transport
GRMZM2G334105	-22	No Description
GRMZM2G447005	-22	No Description
GRMZM2G700569	-18	No Description
GRMZM2G349074	-95	No Description
GRMZM2G303168	-19	No Description
GRMZM5G856391	-190	No Description
GRMZM2G139574	-152	transferase activity, transferring glycosyl groups
GRMZM2G162868	-151	No Description
GRMZM2G479869	-95	No Description
GRMZM2G375222	-171	transcription elongation regulator activity|membrane|positive regulation of RNA elongation from RNA polymerase II promoter|protein homooligomerization
GRMZM2G141873	-17	No Description
GRMZM2G456835	-189	response to stress
GRMZM2G100146	-197	ossification|intracellular|cell adhesion|zinc ion binding
GRMZM2G361066	-26	nucleotide binding|ATP-dependent peptidase activity|serine-type endopeptidase activity|ATP binding|proteolysis|membrane|ATPase activity|nucleoside-triphosphatase activity
GRMZM2G063672	-121	nucleotide binding|ATP binding|nucleoside-triphosphatase activity
GRMZM2G014066	-167	No Description
GRMZM2G307756	-75	No Description
GRMZM5G873546	-88	No Description
GRMZM2G029292	-189	transcription factor activity|sequence-specific DNA binding|regulation of transcription
GRMZM5G806622	-108	No Description
GRMZM2G173868	-198	mannosyl-oligosaccharide 1,2-alpha-mannosidase activity|calcium ion binding|membrane
GRMZM2G076450	-82	protein binding
GRMZM2G116273	-22	No Description
GRMZM2G110714	-136	catalytic activity|tricarboxylic acid cycle|phosphoenolpyruvate carboxylase activity|carbon fixation
GRMZM2G420772	-184	No Description
GRMZM2G555108	-16	No Description
GRMZM2G012031	-88	protein binding
GRMZM2G107408	-163	No Description
GRMZM2G344323	-161	No Description
GRMZM5G884407	-18	No Description
GRMZM2G410033	-189	amino acid transport|membrane
GRMZM5G874483	-165	No Description
GRMZM2G396540	-17	No Description
GRMZM2G125175	-185	No Description
GRMZM2G109299	-158	No Description
GRMZM2G355631	-102	No Description
GRMZM2G024615	-66	catalytic activity|membrane
GRMZM2G315431	-63	ubiquitin ligase complex|ubiquitin-protein ligase activity|binding|protein ubiquitination
GRMZM2G414813	-30	transporter activity|transport|membrane|transmembrane transport
GRMZM2G339214	-135	No Description
GRMZM2G146673	-17	protein folding|heat shock protein binding|unfolded protein binding
GRMZM2G307588	-131	DNA binding|nucleus
AC225790.2_FG003	-86	structural molecule activity|cell adhesion|actin cytoskeleton|homoiothermy|ice binding|response to freezing
GRMZM2G435015	-164	nucleus|transcription regulator activity|regulation of transcription
AC212732.4_FG005	-175	No Description
AC191264.3_FG003	-122	No Description
GRMZM2G150683	-17	extracellular region|iron ion transport|cellular iron ion homeostasis|ferric iron binding
GRMZM2G434696	-180	No Description
GRMZM2G702283	-41	No Description
GRMZM2G443939	-33	protein binding|ATP binding|apoptosis|defense response
GRMZM2G003625	-52	protein binding|ATP binding|apoptosis|defense response
GRMZM2G163550	-184	protein kinase activity|ATP binding|sugar binding|protein amino acid phosphorylation|recognition of pollen
GRMZM5G571844	-121	binding
AC203316.3_FG003	-108	No Description
GRMZM2G142565	-24	No Description
GRMZM2G420774	-14	nucleic acid binding
GRMZM2G123644	-141	DNA binding|RNA binding
GRMZM2G109142	-154	No Description
AC203570.4_FG005	-186	No Description
GRMZM2G481009	-28	No Description
GRMZM2G133233	-81	No Description
GRMZM2G396049	-54	RNA binding|RNA processing
GRMZM2G396049	-47	RNA binding|RNA processing
GRMZM2G315885	-53	RNA binding|RNA processing
GRMZM2G025243	-188	alcohol dehydrogenase (NAD) activity|metabolic process|2,3-dihydro-2,3-dihydroxybenzoate dehydrogenase activity|enterobactin biosynthetic process|oxidoreductase activity|oxidation reduction
GRMZM5G896983	-18	No Description

Supplementary table S6: List of the 127 genes in the maize genome containing the 5 anthocyanin-related motifs predicted by Promzea

maize ID	GO annotation
AC155377.1_FG001	GO:0016459 GO:0005524 GO:0051015 GO:0003774
AC191692.3_FG001	
AC196161.3_FG002	
AC197340.3_FG004	
AC202534.2_FG007	
AC203972.3_FG001	GO:0003735 GO:0006915 GO:0006412 GO:0005524 GO:0005840
AC206030.4_FG001	GO:0015297 GO:0016020 GO:0006855 GO:0015238
AC209810.3_FG002	GO:0006508 GO:0004197
AC211474.3_FG006	GO:0003824 GO:0005488 GO:0008152
AC217947.4_FG002	GO:0016491 GO:0005506 GO:0010181 GO:0009055
GRMZM2G004188	GO:0004518 GO:0005634 GO:0003677 GO:0006281
GRMZM2G005622	
GRMZM2G005753	GO:0031072 GO:0006457 GO:0051082
GRMZM2G011383	
GRMZM2G013016	
GRMZM2G016668	GO:0008270 GO:0005515
GRMZM2G018022	GO:0016779 GO:0008152
GRMZM2G018403	GO:0003735 GO:0005840 GO:0006412 GO:0003723
GRMZM2G018631	GO:0008270 GO:0005515
GRMZM2G021051	GO:0005506 GO:0016491
GRMZM2G021243	
GRMZM2G021277	GO:0006519 GO:0016831 GO:0019752 GO:0030170
GRMZM2G024674	
GRMZM2G025031	GO:0004853 GO:0006779
GRMZM2G026095	GO:0016787 GO:0008270 GO:0008152
GRMZM2G027241	
GRMZM2G027253	GO:0006355 GO:0003677
GRMZM2G027851	GO:0006813 GO:0006885 GO:0008076 GO:0006814 GO:0005249 GO:0015385
GRMZM2G028986	GO:0020037
GRMZM2G031996	
GRMZM2G035749	GO:0003735 GO:0016161 GO:0000272 GO:0006412 GO:0005840
GRMZM2G036837	GO:0008270 GO:0003676
GRMZM2G038279	GO:0006355 GO:0005634 GO:0000785 GO:0003677
GRMZM2G041388	
GRMZM2G043749	

GRMZM2G044348	GO:0016021 GO:0004190
GRMZM2G047214	
GRMZM2G047412	
GRMZM2G050273	
GRMZM2G055899	GO:0009081 GO:0004084
GRMZM2G058630	GO:0004607 GO:0006629
GRMZM2G062541	GO:0005634 GO:0045449 GO:0030528
GRMZM2G063961	GO:0004674 GO:0006468 GO:0004713 GO:0005524
GRMZM2G070279	GO:0009239 GO:0005488 GO:0004022 GO:0008667
GRMZM2G071745	GO:0020037
GRMZM2G074373	GO:0043565 GO:0003700 GO:0016021 GO:0006355 GO:0046983 GO:0007186 GO:0005634
GRMZM2G074462	GO:0003824 GO:0005975 GO:0030246
GRMZM2G074604	GO:0005737 GO:0009058 GO:0016841 GO:0016211 GO:0006559
GRMZM2G074824	GO:0003824
GRMZM2G075366	
GRMZM2G077696	
GRMZM2G082037	GO:0016758 GO:0008152
GRMZM2G083555	
GRMZM2G085303	
GRMZM2G085934	
GRMZM2G088140	GO:0005634 GO:0045449 GO:0006351 GO:0003677
GRMZM2G094490	GO:0004176 GO:0006508 GO:0016020 GO:0005524 GO:0004252
GRMZM2G095441	GO:0005484 GO:0006886 GO:0016020 GO:0016192
GRMZM2G099052	
GRMZM2G100568	
GRMZM2G104920	GO:0008601 GO:0007165 GO:0000159
GRMZM2G106604	
GRMZM2G106650	GO:0004497 GO:0020037 GO:0009055
GRMZM2G107299	
GRMZM2G109627	GO:0045449 GO:0003677
GRMZM2G109680	
GRMZM2G110145	GO:0016020 GO:0016760 GO:0030244
GRMZM2G113057	
GRMZM2G113319	
GRMZM2G115579	
GRMZM2G115775	GO:0016020 GO:0016192 GO:0005515
GRMZM2G125216	
GRMZM2G125448	GO:0016747
GRMZM2G129979	GO:0005634

GRMZM2G135095	GO:0003735 GO:0006412 GO:0005840
GRMZM2G142390	GO:0004674 GO:0006468 GO:0004713 GO:0005524
GRMZM2G143703	
GRMZM2G146207	
GRMZM2G146292	
GRMZM2G147245	GO:0004497 GO:0020037 GO:0009055
GRMZM2G152370	
GRMZM2G153536	GO:0009081 GO:0004084
GRMZM2G163277	
GRMZM2G163297	GO:0003723
GRMZM2G166719	GO:0004674 GO:0006468 GO:0004713 GO:0005524
GRMZM2G168675	
GRMZM2G169584	GO:0006915 GO:0005524
GRMZM2G170420	
GRMZM2G176129	
GRMZM2G176630	GO:0006486 GO:0016020 GO:0005529 GO:0008378 GO:0042309 GO:0050826 GO:0050825
GRMZM2G178278	GO:0016192
GRMZM2G312061	GO:0004869
GRMZM2G314593	
GRMZM2G315862	
GRMZM2G325008	GO:0009987 GO:0005975 GO:0004553
GRMZM2G325575	GO:0008199 GO:0006879 GO:0016491
GRMZM2G336879	GO:0005971 GO:0004748 GO:0006260
GRMZM2G347471	
GRMZM2G348039	
GRMZM2G352381	GO:0016021 GO:0007186
GRMZM2G356718	GO:0003824 GO:0003677
GRMZM2G361066	GO:0004176 GO:0006508 GO:0016020 GO:0005524 GO:0004252
GRMZM2G366434	GO:0003700 GO:0006355
GRMZM2G368827	
GRMZM2G371030	
GRMZM2G381709	GO:0019787 GO:0043687 GO:0051246
GRMZM2G394783	GO:0004497 GO:0020037 GO:0009055
GRMZM2G398758	GO:0016021 GO:0007186 GO:0003677
GRMZM2G402797	
GRMZM2G405170	GO:0006355 GO:0003677
GRMZM2G415579	GO:0055114 GO:0016491
GRMZM2G422147	
GRMZM2G428905	
GRMZM2G432566	

GRMZM2G434839	GO:0031072 GO:0006457 GO:0051082
GRMZM2G459540	GO:0008270 GO:0005622
GRMZM2G459746	GO:0003676
GRMZM2G465046	GO:0006629 GO:0016788
GRMZM2G472236	
GRMZM2G552418	
GRMZM5G820788	
GRMZM5G838435	
GRMZM5G861978	
GRMZM5G869587	
GRMZM5G871174	
GRMZM5G890241	
GRMZM5G894340	
