# Supplementary material for: Promzea: a pipeline for discovery of co-regulatory motifs in maize and other plant species and its application to the anthocyanin and phlobaphene biosynthetic pathways and the Maize Development Atlas
Source: BMC Plant Biol. 2013 Mar 15;13:42. doi: 10.1186/1471-2229-13-42 (PMC3658923; doi:10.1186/1471-2229-13-42)
Supplement: Additional file 3 — Comparison of standalone motif discovery programs. Different motif discovery programs predicted motifs embedded in 125 sets of sequences belonging to the Sandve et al. (2007) benchmark data set. The benchmark software calculated the nucleotide Correlation Coefficient scores (nCC scores), a measure of the correlation between the known nucleotide positions and the predicted nucleotide positions. The nCC scores are compared for: (A) BioProspector and MEME, (B) Weeder and MEME, and (C) Weeder and BioProspector. The Spearman correlation (r) between the sets of nCC scores is indicated. [file 1471-2229-13-42-S3.pdf]

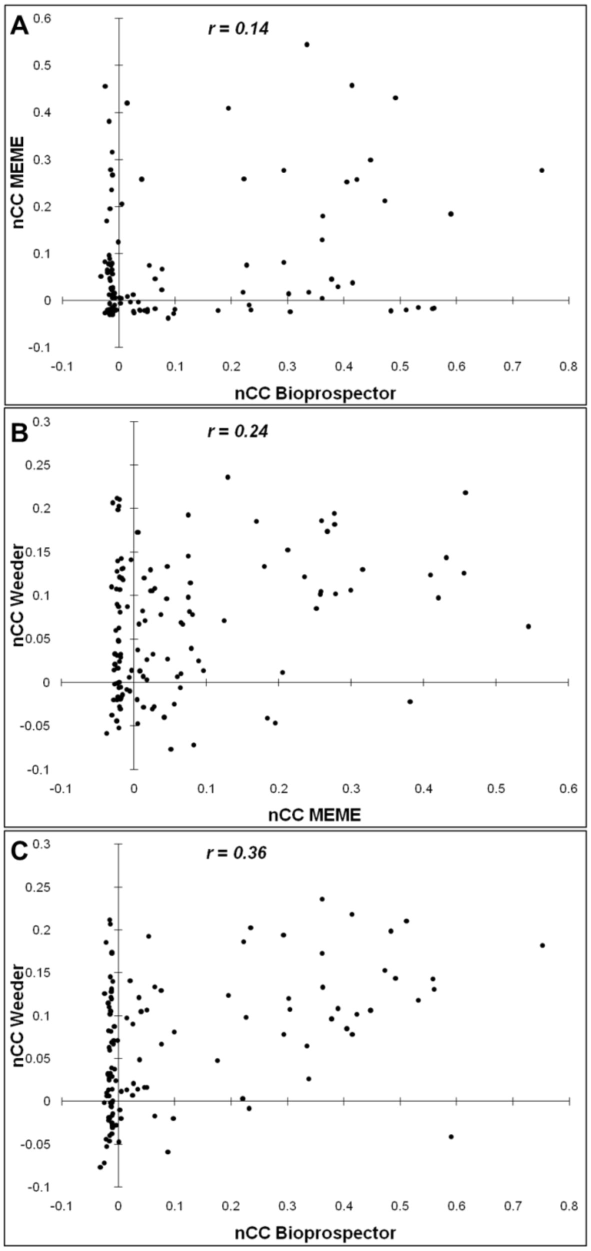

Additional File 3. Comparison of standalone motif discovery programs. Different motif discovery programs predicted motifs embedded in 125 sets of sequences belonging to the Sandve et al (2007) benchmark dataset. The benchmark software calculated the nucleotide Correlation Coefficient scores (nCC scores), a measure of the correlation between the known nucleotide positions and the predicted nucleotide positions. The nCC scores are compared for (A) BioProspector and MEME, (B) Weeder and MEME, and (C) Weeder and BioProspector. The Spearman correlation (r) between the sets of nCC scores is indicated.
