# Supplementary material for: Promzea: a pipeline for discovery of co-regulatory motifs in maize and other plant species and its application to the anthocyanin and phlobaphene biosynthetic pathways and the Maize Development Atlas
Source: BMC Plant Biol. 2013 Mar 15;13:42. doi: 10.1186/1471-2229-13-42 (PMC3658923; doi:10.1186/1471-2229-13-42)
Supplement: Additional file 4 — Effectiveness of combining different motif discovery programs. The output of each motif discovery program, applied to the Sandve et al. (2007) benchmark data set, was measured using the Nucleotide Correlation Coefficient (nCC) and the nucleotide Sensitivity (nSn). Shown are scores for the three data sets that comprise the Sandve data set: (A) synthetic (Algorithm Markov), (B) semi-synthetic (Algorithm Real), and (C) real promoters (Model Real). Shown are the scores of each standalone, unfiltered program, as well as the scores after combining the outputs of the three programs with filtering (combined). The error bars represent the 95% mean confidence interval. [file 1471-2229-13-42-S4.pdf]

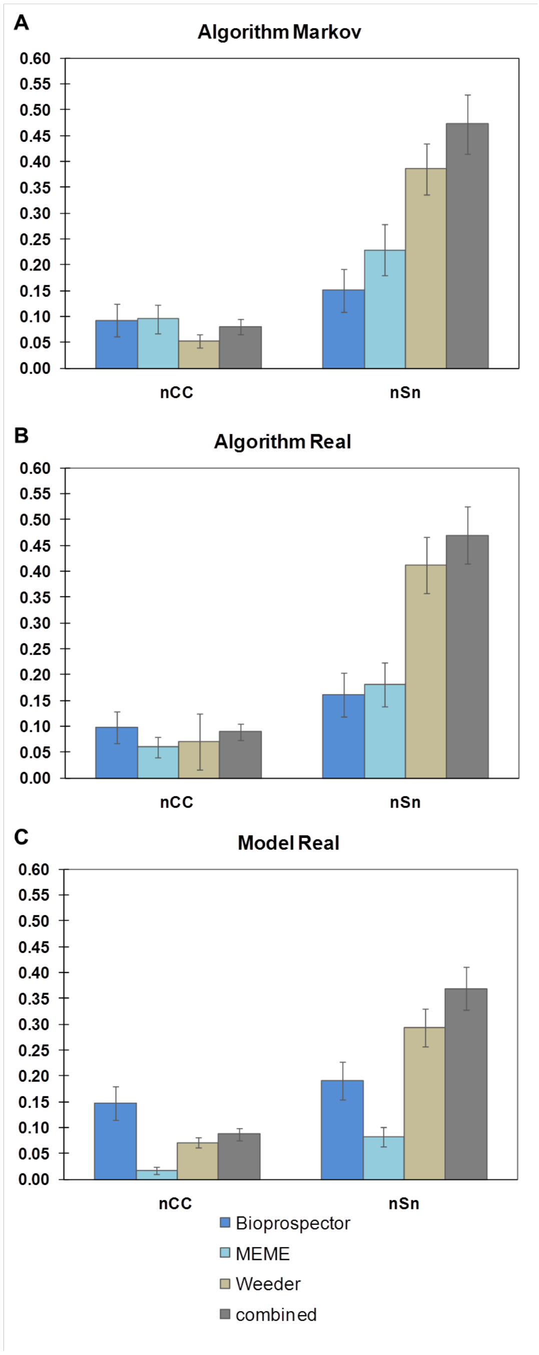

Additional File 4. Effectiveness of combining different motif discovery programs. The output of each motif discovery program, applied to the Sandve et al. (2007) benchmark data set, was measured using the Nucleotide Correlation Coefficient (nCC) and the nucleotide sensitivity score (nSn). Shown are scores for the three data sets that comprise the Sandve data set: (A) synthetic (Algorithm Markov), (B) semi-synthetic (Algorithm Real), and (C) real promoters (Model Real). Shown are the scores of each standalone unfiltered program, as well as the scores after combining the outputs of the three programs with filtering (combined). The error bars represent the 95% mean confidence interval.
