# Supplementary material for: Promzea: a pipeline for discovery of co-regulatory motifs in maize and other plant species and its application to the anthocyanin and phlobaphene biosynthetic pathways and the Maize Development Atlas
Source: BMC Plant Biol. 2013 Mar 15;13:42. doi: 10.1186/1471-2229-13-42 (PMC3658923; doi:10.1186/1471-2229-13-42)
Supplement: Additional file 5 — Anthocyanin and phlobaphene pathway gene sequences. The sequences of the cDNAs encoding the enzymes involved in the maize anthocyanin and phlobaphene biosynthetic pathways. A subset of these cDNAs is known to contain experimentally defined cis-acting elements in their promoters that permit co-expression. [file 1471-2229-13-42-S5.pdf]

>M95077.1_PAL1
GTTCCCGCTGTACCGCTTCGTGCGCGAGGAGCTCGGCTGCGTGTTCCTGACCGGCGAGAGGCTCAAGTCC
CCCGGCGAGGAGTGCAACAAGGTGTTCGTCGGCATCAGCCAGGGCAAGCTCGTGGACCCCATGCTCGAGT
GCCTCAAGGAGTGGGACGGCAAGCCGCTGCCCATCAACGTCAAGTAAAGA
>BT042976.1_C2
CCGGACTACTACTTCCGGATCACCAAGAGCGAGCACCTCACCGACCTCAAGGAGAAATTCAAGAGGATGT
GCGACAAGTCGATGATCCGGAAGCGTTACATGCACCTGACGGAGGAGTTCCTGGCGGAGAACCCGAGCAT
GTGCGCGTACATGGCGCCGTCGCTGGACGCGCGGCAGGACGTGGTGGTGGAGGTGCCGAAGCTGGGG
AAGGCGGCGGCGCAGAAGGCGATCAAGGAGTGGGGGCAGCCAAAGTCGCGGATCACGCACCTGGTGTTCT
GCACCACGTCCGGGGTGGACATGCCGGGCGCCGACTACCAGCTGACCAAGGCGCTGGGCCTGCGCCCCTC
CGTGAACCGCCTCATGATGTACCAGCAGGGGTGCTTCGCGGGCGGCACGGTGCTGCGCGTGGCCAAGGAC
CTCGCGGAGAACAACCGCGGCGCGCGGGTGCTGGTGGTGTGCTCCGAGATCACGGCCGTCACGTTCCGCG
GCCCCTCCGAGTCGCACCTCGACTCGCTCGTGGGCCAGGCGCTGTTCGGCGACGGCGCGGCGGCCGTGGT
CGTGGGCGCCGACCCGGACGACCGCGTCGAGCGCCCGCTCTTCCAGCTCGTCTCCGCCGCCCAGACCATC
CTGCCCGACTCGGAGGGCGCCATCGACGGCCACCTCCGCGGGGTGGGGCTCACCTTCCACCTGCTCAAGG
ACGTGCCTGGCCTCATCTCCAAGAACATCGGCCGCGCGCTGGACGACGCGTTCAAGCCGCTCGGCATCTC
CGACTGGAACTCCATCTTCTGGGTGGCGCACCCCGGCGGGCCCGCCATCCTCGACCAGGTGGAGGCCAAG
GTCGGGCTGGACAAGGCCAGGATGCGCGCCACCCGCCACGTCCTCTCCGAGTACGGCAACATGTCCAGCG
CCTGCGTCCTCTTCATCCTCGACGAGATGCGCAAGCGCTCCGCCGAGGACGGACAGGCCACCACGGGCGA
GGGCCTCGACTGGGGCGTCCTCTTCGGCTTCGGCCCGGGACTCACCGTCGAGACCGTCGTGCTCCACAGC
GTCCCCATCACCACCGGAGCGGCCACCGCCTGATTCATCGATTCATCAACGATCAAATTCCTCCCCTCTC
CGATCCAATTCGTCGTCGTCTCGTTCGTCATTATTTTACGTCGTCCGTCCGCAAATAATGTGCTCTCTGC
TATAATTGTCGTGTGTAGTAGTAAGTAGCTGTTACTATTTTTCCATGTACTGTCAGTCGCACAATCATCA
TATATAATTAATATCTCTATATATGCATTT
>BT061979.1_C2
CACACACAGCAGGCAGCAGCTCGATCGCTAGCTCCTCCGAACCGGAAAGCCAAGCCCGCTTTGGCGATCG
TGCTTGCTTGCTTGGTTCCTTCAATTCCTGCCGGAGCCAGCGAGAGCTAGCTGCTGCTGTCGCGTGGTAG
ACGTCGTCTTGCCAGCGAGAGCCTAGCTCGATCGGTCTCTCTGGTACAACGTAGGGAGAAAGATCGAGGG
GGAGAGGACGACGACGATGGCCGGCGCGACCGTGACCGTGGAGGAGGTGAGGAAGGCCCAGCGCGCCACC
GGCCCCGCCACCGTGCTGGCGATCGGCACCGCCACGCCCGCCAACTGCGTGTACCAGGCCGACTACCCGG
ACTACTACTTCCGGATCACCAAGAGCGAGCACCTCACCGACCTCAAGGAGAAATTCAAGAGGATGTGCGA
CAAGTCGATGATCCGGAAGCGTTACATGCACCTGACGGAGGAGTTCCTGGCGGAGAACCCGAGCATGTGC
GCGTACATGGCGCCGTCGCTGGACGCGCGGCAGGACGTGGTGGTGGAGGTGCCGAAGCTGGGGAAGG
CGGCGGCGCAGAAGGCGATCAAGGAGTGGGGGCAGCCAAAGTCGCGGATCACGCACCTGGTGTTCTGCAC
CACGTCCGGGGTGGACATGCCGGGCGCCGACTACCAGCTGACCAAGGCGCTGGGCCTGCGCCCCTCCGTG
AACCGCCTCATGATGTACCAGCAGGGGTGCTTCGCGGGCGGCACGGTGCTGCGCGTGGCCAAGGACCTCG
CGGAGAACAACCGCGGCGCGCGGGTGCTGGTGGTGTGCTCCGAGATCACGGCCGTCACGTTCCGCGGCCC
CTCCGAGTCGCACCTCGACTCGCTCGTGGGCCAGGCGCTGTTCGGCGACGGCGCGGCGGCCGTGGTCGTG
GGCGCCGACCCGGACGACCGCGTCGAGCGCCCGCTCTTCCAGCTCGTCTCCGCCGCCCAGACCATCCTGC
CCGACTCGGAGGGCGCCATCGACGGCCACCTCCGCGAGGTGGGGCTCACCTTCCACCTGCTCAAGGACGT
GCCTGGCCTCATCTCCAAGAACATCGGCCGCGCGCTGGACGACGCGTTCAAGCCGCTCGGCATCTCCGAC
TGGAACTCCATCTTCTGGGTGGCGCACCCCGGCGGGCCCGCCATCCTCGACCAGGTGGAGGCCAAGGTCG
GGCTGGACAAGGCCAGGATGCGCGCCACCCGCCACGTCCTCTCCGAGTACGGCAACATGTCCAGCGCCTG
CGTCCTCTTCATCCTCGACGAGATGCGCAAGCGCTCCGCCGAGGACGGACAGGCCACCACGGGCGAGGGC
CTCGACTGGGGCGTCCTCTTCGGCTTCGGCCCGGGACTCACCGTCGAGACCGTCGTGCTCCACAGCGTCC
CCATCACCACCGGAGCGGCCACCGCCTGATTCATCGATTCATCAACGATCAAATTCCTCCCCTCTCCGAT
CCAATTCGTCGTCGTCTCGTTCGTCATTATTTTACGTCGTCCGTCCGCAAATAATGTGCTCTCTGCTATA
ATTGTCGTGTGTAGTAGTAAGTAGCTGTTACTATTTTTCCATGTACTGTCAGTCGCACAATCATCATATA

TAATTAATATCTCTATATATGCATTTCATGCAAGACCAGGGTAGAGCTAGCTTGAGGAGAAGACCCTTAT
CGTTGTCACTTTAGCATGCATGGGGTGGTGAGGTATTGGGTATACCTGTGTAAGACAAGCTTGGCTGGTT
TAATTATATATATTTTTTTT
>NM_001156113.1_CI
AGCACGGGTCACCTCGCCGGGAATCCCACCTCGCCAGTGCCGCCTCGCCGCTCTCCCACATCGTGTCCTC
CGCCCCTTCGCACGGACGCACACGCACCTCACCTCTCACAATTCCAAGCCCACCAGTCCACCACTCTCCG
CTTCGCTTCTTGTCGCTGCGGCGCGCGGCCATGGTCTCCCTCCGCTTCCCTACCGCTGCAATCCCCCGCC
TCCCGCCATCCCAGGCCCCCAACGGCGTCGCCATCGCCGCCACGGTCGCCGCTGTAGCCGCGGCCGCAGC
TGCAGTAGCGGCCAGCTTGACCCTCACGGGCAAGTCCACCGGCCGGCCAGTCCCGCACCCTGCCCCTCTC
TGGGCCTCCCTCTCCCTGGCCGACGCGGCGGCCCCCAGCTCCGTCGAACCCCGGACCGGCGCGACATTCC
CCACCGAGGCCGCCGTTGGGCGCCGCCTACTCGGCATAGGCCTCCGGAAGACCAGCGTCCTCGGGCTCAA
GTCCATCGACGTCTACGCATTCGGCGTGTATGCGGATGACAACGATTTGAAGCAGCAGCTGAAGGAGAAG
TACTCGAAGTTCTCGGTCTCGGAACTGAAGGGAAACGGTGAGCTGATCAACGATGCGCTAGAGCGTGACA
TACCGATGACGGTCAGGCTGCAGATTGTTTATGGGAGGCTGAGTATTCGCTCGGTTCGCAGCGCATTTGA
GAAGAGTGTGGGAAGTAGGCTTCAGAAGTTCGGTGGACAAGACACCAAAGAGTTGCTTCAGAGTTTTGTT
GCGGTTTTCAAGGATGAATACAAATTACCAAAGGGGTCTGTGATTGAGCTTTCGAGGGAATCTAACCATG
TCCTTAAAATTTGCATTGAAGGCGAGGAAGTGGGGAGCATTCAGAGCAAGCTCTTGTGCAAGTCTCTCTT
AGATCTCTACATCGGAGATGACTCCTTTGACGAAAATGCGAAAGACGGCATCCATGAAAATATAGCTAGC
ATTCTTAAAAGTTAAATGGGATGCTTAAGGAAGGTTACATGGAACTGGGAGATTTTTCAGGGAAGATATT
TTTAATTCCAGCAATACCTCGACTGTGCCTAGTAAGTAGCGCACAAGTTTTTTTTCAGTAGCGCGTGAGG
AACTGAGGAAAAGGATAGATCCTTCCAAGTACAGCCTTTTTAAAAAAAAAAAAAAAA
>EU963832.1_CI
AGCACGGGTCACCTCGCCGGGAATCCCACCTCGCCAGTGCCGCCTCGCCGCTCTCCCACATCGTGTCCTC
CGCCCCTTCGCACGGACGCACACGCACCTCACCTCTCACAATTCCAAGCCCACCAGTCCACCACTCTCCG
CTTCGCTTCTTGTCGCTGCGGCGCGCGGCCATGGTCTCCCTCCGCTTCCCTACCGCTGCAATCCCCCGCC
TCCCGCCATCCCAGGCCCCCAACGGCGTCGCCATCGCCGCCACGGTCGCCGCTGTAGCCGCGGCCGCAGC
TGCAGTAGCGGCCAGCTTGACCCTCACGGGCAAGTCCACCGGCCGGCCAGTCCCGCACCCTGCCCCTCTC
TGGGCCTCCCTCTCCCTGGCCGACGCGGCGGCCCCCAGCTCCGTCGAACCCCGGACCGGCGCGACATTCC
CCACCGAGGCCGCCGTTGGGCGCCGCCTACTCGGCATAGGCCTCCGGAAGACCAGCGTCCTCGGGCTCAA
GTCCATCGACGTCTACGCATTCGGCGTGTATGCGGATGACAACGATTTGAAGCAGCAGCTGAAGGAGAAG
TACTCGAAGTTCTCGGTCTCGGAACTGAAGGGAAACGGTGAGCTGATCAACGATGCGCTAGAGCGTGACA
TACCGATGACGGTCAGGCTGCAGATTGTTTATGGGAGGCTGAGTATTCGCTCGGTTCGCAGCGCATTTGA
GAAGAGTGTGGGAAGTAGGCTTCAGAAGTTCGGTGGACAAGACACCAAAGAGTTGCTTCAGAGTTTTGTT
GCGGTTTTCAAGGATGAATACAAATTACCAAAGGGGTCTGTGATTGAGCTTTCGAGGGAATCTAACCATG
TCCTTAAAATTTGCATTGAAGGCGAGGAAGTGGGGAGCATTCAGAGCAAGCTCTTGTGCAAGTCTCTCTT
AGATCTCTACATCGGAGATGACTCCTTTGACGAAAATGCGAAAGACGGCATCCATGAAAATATAGCTAGC
ATTCTTAAAAGTTAAATGGGATGCTTAAGGAAGGTTACATGGAACTGGGAGATTTTTCAGGGAAGATATT
TTTAATTCCAGCAATACCTCGACTGTGCCTAGTAAGTAGCGCACAAGTTTTTTTTCAGTAGCGCGTGAGG
AACTGAGGAAAAGGATAGATCCTTCCAAGTACAGCCTTTTTAAAAAAAAAAAAAAAA
>NM_001112225.1_f3h
AGAAGCAGCATCACGATCCCGGCCCGGCGGCCCGTACGTCGACGAGGAGGAGGAGGACGTACGTACGCAG
AGCAGCTGCTGGCACCATGGCTCCCGTGAGCATCAGCGCTGTTCCGTTCCTCCCGACGGCGGCGGAGGGG
GAGACGAACGTGCGCGCGTCGTTCGTGCGCGAGGAGGACGAGCGCCCCAAGGTGCCGCACGACCGCTTCA
GCGACGAGGTGCCGGTGGTGTCGCTCGAGGGCATCGACGGGCCGCGCAGGCGGGCCGAGATCCGCGGCCG
CGTGGCCGCGGCCTGCGAGGACTGGGGCATCTTCCAGGTGGTGGACCACGGCGTGGACGCCGCGCTCGTG
GCCGACATGGCGCGCCTCGCCCGAGACTTCTTCGCGCTCCCGCCCGAGGACAAGCTCCGCTTCGACATGT
CCGGCGGGAAGAAGGGCGGCTTCATCGTCTCCAGCCACCTCCAGGGGGAGGCGGTGCAGGACTGGCGTGA
GATCGTGACCTACTTCTCGTACCCGGTGAAGGCCCGCGACTACTCCCGGTGGCCGGACAAGCCGGCGGCG

TGGCGGGCGGTGGTGGAGCGGTACAGCGAGCAGCTGATGGCGCTGGCGTGCAGGCTCCTGGGCGTGCTCT
CCGAGGCCATGGGCCTGGACACGGAGGCGCTGGCCAGGGCCTGCGTGGACATGGACCAGAAGGTGGTGGT
CAACTTCTACCCGAGGTGCCCGCAGCCGGACCTCACGCTGGGGCTCAAGCGCCACACCGACCCCGGCACC
ATCACGCTGCTGCTGCAGGACCTGGTCGGCGGCCTCCAGGCCACGCGCGACGGCGGCCGGACCTGGATCA
CCGTGCAGCCCGTGGAGGGCGCCTTCGTCGTCAACCTCGGCGACCACGGCCACCTCCTGAGCAACGGCAG
GTTCAAGAACGCGGACCACCAGGCGGTGGTGAACTCGGAGTGCAGCCGCCTGTCCATCGCCACGTTCCAG
AACCCGGCGCCCGACGCGACGGTGTACCCGCTGGCCGTGCGCGACGGGGAGGCGCCCATACTGGACCATC
CCATCACCTTCGCCGAGATGTACCGCCGCAAGATGGCCCGCGACATCGAGCTCGCCAGGCTCAAGAAGCA
GGCCAAGGCCGACAAGAAGCAGCAGCAGCAGAGTGCCAACAAGGAGTTCGCCGACTCCAAGCCTCTCGAC
GCCATTTTTGCCTGATATGGTAGAGGGCGCGCGACTACCGATCCGTTGTTATTGCTGCGTGTGATTAATT
TTCATCTATAAGAACATAGCTACGTGCGACCGCGCGTGTGGTGATTAATTAAGGTCTGAATAAAATAATG
GCTGCACAAAACTAGGAAGCGATCGATCACCCAGATAAATGTATGTGCGTTCCCTTTTCTTCTTTCAAGA
TGTGTAATTTTCAACGTGCGGTTCCAGATGCATGGACTGGACTGGACCGGAGTGCATTGTAATTCTTTTG
TTTCCCCCTGTTGAATATGCGAGAGGTGTGCATGTCTTTGTATGAAAAAAAAA
>BT034095.1_a1
CAAAGCAAGTGTCTGCTCACTCAGTCCTGCGCAAGAGCTCGCTCTCGGAGAAAAAAACGCGGGAGGCGAT
AATGGAGGGAGGTGCCGGTGCGAGCGAGAAAGGGAAGGTGCTGGTCACGGGGGCGTCGGGCTTCGTCGGC
TCCTGGCTCGTCATGAAGCTCCTCCAGGCCGGCTACACCGTCCGGGCGACCGTGCGCGATCCCGCGAACG
TTGGGAAGACGAAGCCATTGATGGACCTTCCCGGAGCAACGGAGCGCCTGTCCATATGGAAAGCCGACCT
GGCGGAGGAAGGCAGCTTCCACGACGCCATCAGGGGCTGCACCGGCGTCTTCCACGTCGCCACGCCCATG
GACTTCCTGTCCAAAGACCCTGAGAATGAGGTAATCAAGCCGACGGTGGAAGGGATGATAAGCATCATGC
GGGCATGCAAGGAGGCCGGCACCGTGCGGCGCATCGTCTTCACTTCCTCCGCCGGGACGGTCAACCTGGA
GGAGCGGCAGAGGCCCGTCTACGACGAGGAAAGCTGGACCGACGTCGACTTCTGCCGTCGCGTCAAGATG
ACCGGATGGATGTACTTCGTGTCTAAAACCCTGGCGGAGAAGGCGGCCCTGGCGTACGCGGCGGAGCACG
GCCTGGACCTGGTCACCATCATCCCGACGCTCGTGGTCGGCCCGTTCATCAGCGCGTCCATGCCGCCCAG
CCTCATCACCGCGCTGGCGCTCATCACGGGGAACGCGCCCCACTACTCGATCCTCAAGCAGGTGCAGCTC
ATCCACCTCGACGACCTCTGCGACGCCGAGATCTTCCTCTTCGAGAACCCGGCCGCGGCCGGGCGCTACG
TCTGCTCCTCGCACGACGTCACCATCCACGGCCTCGCCGCCATGCTCAGGGATAGGTACCCCGAGTACGA
CGTCCCGCAGAGGTTCCCCGGGATCCAGGACGACCTCCAGCCGGTGCGCTTCTCGTCCAAGAAGCTCCAG
GACCTCGGGTTCACCTTCAGGTACAAGACGCTGGAGGACATGTTCGACGCCGCCATCCGGACTTGCCAGG
AGAAGGGCCTCATCCCCCTCGCCACTGCCGCCGGAGGGGACGGCTTTGCCTCGGTGCGCGCACCCGGCGA
GACGGAGGCGACGATTGGCGCTTAAGCAACAATCACCCGGCTCTCCCCGTCGATGATGCTATCAGCTATC
TATCTCTTCGAGCTCCGCTGCCAATACGGAACTTACTTCCATGTTCAACGAATTGTTTTATCCCCTGGAT
TACGTATTGTTTTTATGTTTTATACCCAAACAACGAATGTTTCTCAGTATTTTACTACAGCTAGCCCCTA
TAGTAGAGATTCTATTATATGAATAAGCTATCTGTAGAGGCTCTGGTGCAATAT
>BT043174.1_a1
GCAAGTGTCTGCTCACTCAGTCCTGCGCAAGAGCTCGCTCTCGGAGAAAAAAACGCGGGAGGCGATAATG
GAGGGAGGTGCCGGTGCGAGCGAGAAAGGGAAGGTGCTGGTCACGGGGGCGTCGGGCTTCGTCGGCTCCT
GGCTCGTCATGAAGCTCCTCCAGGCCGGCTACACCGTCCGGGCGACCGTGCGCGATCCCGCGAACGTTGG
GAAGACGAAGCCATTGATGGACCTTCCCGGAGCAACGGAGCGCCTGTCCATATGGAAAGCCGACCTGGCG
GAGGAAGGCAGCTTCCACGACGCCATCAGGGGCTGCACCGGCGTCTTCCACGTCGCCACGCCCATGGACT
TCCTGTCCAAAGACCCTGAGAATGAGGTAATCAAGCCGACGGTGGAAGGGATGATAAGCATCATGCGGGC
ATGCAAGGAGGCCGGCACCGTGCGGCGCATCGTCTTCACTTCCTCCGCCGGGACGGTCAACCTGGAGGAG
CGGCAGAGGCCCGTCTACGACGAGGAAAGCTGGACCGACGTCGACTTCTGCCGTCGCGTCAAGATGACCG
GATGGATGTACTTCGTGTCTAAAACCCTGGCGGAGAAGGCGGCCCTGGCGTACGCGGCGGAGCACGGCCT
GGACCTGGTCACCATCATCCCGACGCTCGTGGTCGGCCCGTTCATCAGCGCGTCCATGCCGCCCAGCCTC
ATCACCGCGCTGGCGCTCATCACGGGGAACGCGCCCCACTACTCGATCCTCAAGCAGGTGCAGCTCATCC
ACCTCGACGACCTCTGCGACGCCGAGATCTTCCTCTTCGAGAACCCGGCCGCGGCCGGGCGCTACGTCTG

CTCCTCGCACGACGTCACCATCCACGGCCTCGCCGCCATGCTCAGGGATAGGTACCCCGAGTACGACGTC
CCGCAGAGGTTCCCCGGGATCCAGGACGACCTCCAGCCGGTGCGCTTCTCGTCCAAGAAGCTCCAGGACC
TCGGGTTCACCTTCAGGTACAAGACGCTGGAGGACATGTTCGACGCCGCCATCCGGACTTGCCAGGAGAA
GGGCCTCATCCCCCTCGCCACTGCCGCCGGAGGGGACGGCTTTGCCTCGGTGCGCGCACCCGGCGAGACG
GAGGCGACGATTGGCGCTTAAGCAACAATCACCCGGCTCTCCCCGTCGATGATGCTATCAGCTATCTATC
TCTTCGAGCTCCGCTGCCAATACGGAACTTACTTCCATGTTCAACGAATTGTTTTATCCCCTGGATTACG
TATTGTTTTTATGTTTTATACCCAAACAACGAATGTTTCTCAGTATTTTACTACAGCTAGCCCCTATAGT
AGAGATTCTATTATATGAATAAGCTATCTGTAGAGGCTCTGGTGCAATATATATTTCGAGTTATTTGGAT
TACTAGCATAATTCAATTTTT
>BT067630.1_a1
GTGTCTGCTCACTCAGTCCTGCGCAAGAGCTCGCTCTCGGAGAAAAAAACGCGGGAGGCGATAATGGAGG
GAGGTGCCGGTGCGAGCGAGAAAGGGAAGGTGCTGGTCACGGGGGCGTCGGGCTTCGTCGGCTCCTGGCT
CGTCATGAAGCTCCTCCAGGCCGGCTACACCGTCCGGGCGACCGTGCGCGATCCCGCGAACGTTGGGAAG
ACGAAGCCATTGATGGACCTTCCCGGAGCAACGGAGCGCCTGTCCATATGGAAAGCCGACCTGGCGGAGG
AAGGCAGCTTCCACGACGCCATCAGGGGCTGCACCGGCGTCTTCCACGTCGCCACGCCCATGGACTTCCT
GTCCAAAGACCCTGAGAATGAGGTAATCAAGCCGACGGTGGAAGGGATGATAAGCATCATGCGGGCATGC
AAGGAGGCCGGCACCGTGCGGCGCATCGTCTTCACTTCCTCCGCCGGGACGGTCAACCTGGAGGAGCGGC
AGAGGCCCGTCTACGACGAGGAAAGCTGGACCGACGTCGACTTCTGCCGTCGCGTCAAGATGACCGGATG
GATGTACTTCGTGTCTAAAACCCTGGCGGAGAAGGCGGCCCTGGCGTACGCGGCGGAGCACGGCCTGGAC
CTGGTCACCATCATCCCGACGCTCGTGGTCGGCCCGTTCATCAGCGCGTCCATGCCGCCCAGCCTCATCA
CCGCGCTGGCGCTCATCACGGGGAACGCGCCCCACTACTCGATCCTCAAGCAGGTGCAGCTCATCCACCT
CGACGACCTCTGCGACGCCGAGATCTTCCTCTTCGAGAACCCGGCCGCGGCCGGGCGCTACGTCTGCTCC
TCGCACGACGTCACCATCCACGGCCTCGCCGCCATGCTCAGGGATAGGTACCCCGAGTACGACGTCCCGC
AGAGGTTCCCCGGGATCCAGGACGACCTCCAGCCGGTGCGCTTCTCGTCCAAGAAGCTCCAGGACCTCGG
GTTCACCTTCAGGTACAAGACGCTGGAGGACATGTTCGACGCCGCCATCCGGACTTGCCAGGAGAAGGGC
CTCATCCCCCTCGCCACTGCCGCCGGAGGGGACGGCTTTGCCTCGGTGCGCGCACCCGGCGAGACGGAGG
CGACGATTGGCGCTTAAGCAACAATCACCCGGCTCTCCCCGTCGATGATGCTATCAGCTATCTATCTCTT
CGAGCTCCGCTGCCAATACGGAACTTACTTCCATGTTCAACGAATTGTTTTATCCCCTGGATTACGTATT
GTTTTTATGTTTTATACCCAAACAACGAATGTTTCTCAGTATTTTACTACAGCTAGCCCCTATAGTAGAG
ATTCTATTATATGAATAAGCTATCTGTAGAGGCTCTGGTGCAATATATATTTCGAGTTATTTGGATT
>NM_001112604.1_anthocyaninless2_a2
ATATTTAAAAATTCAATATTTGTTTGTTTATGAGTGTTTGATGAGTATATTGTACCTCTTGTAAACTGAA
GCACACGATTAATTCTTTGTTAAAGAATGCCATGATAATAGTTTGCTATAAATGAAATTAATGTTAAATC
AATTTGTTAAAAATTATGGTTTCTTCTGAATTTAGTTATTGCCGATAGAGAAAAAACATTGAAAAAATAT
TTAGATCATATACGGCTGAATTTAGTTATTGATGATCTTATTTGTTATATTTTATTTGTTTGAGGAATAC
ACGCTTATTTTACTTCACCCTGTTTGTTACTTTAGTCACTAGATTAAACTTTAGTTTAATCGCTAAAGTA
CCTCGTTGGTTTCAGTGACTAAACCAGACTAAAGAGCATTAATTGATGTAAATAATGACTATGTTGTCCA
TATTAATTAGTGGATATTTGCTGCAAAAAGAAAGTGAAGAGGGCAAATAAGGTAAAAATCCTACTTTAAT
CCCTTTTAGCCATCCCTTGGTGACTAAAGAATTAAAGTTTAGTCACAACACTTTAGTCACCATATTTAAT
TCTTTAGGGACTAAAAGTGACTAAACTTTAGTCACCCCAAACTAAACGGTACCTTAGATCTATTTATTTG
AGGAATGCAAGTGTTTGAATAACTATTCTATTTAAATAAGATCTACCTAACGACGTACGGACATCTAACT
AGTATAGAATTAACGTACACATGTATTGATTTGAGTGACCCATGGCTGATTCCAGTGATCAACAATAATC
CTGCCAAGCATCAAGCTAATCCCAAGGTGCCACGGAATGGTCTCGCCCGCCGCCGCTGGCCACATATACC
AGCAGGTGACCGTCGGATTGGGTGGCGCTTGGCACGGAAAAAAATTTGGTGCAGCACTGCTGCAAACCAG
AGTGGTTTGCAGCCCCTCACAAAAAGGAGGATAGACCCCACCTGCAGGTCCAGCAGATAACGTTTTAGTT
GTATTATTGTTGCTGCGGGTGGGGTCTATCCTCTTTTTTGTGAGGGGCTGCAAACACTCTGGTTTGCAGC
AGTGCTGCACCAAATTTTTTTCCCTTGGCACGTGTTCGGGTGGTAGCTGGCTCCTCCATGCAATGCCTAT
GCCTGTCGTCGCGATCGCAACCACCAGTCAAGACGAATGGCAGGCAGCTAAGTAGCTAACAACAACAGGC

TTGTATTGTATGTACAGCAATATATATACATCTCGAGAAGCTCCTAGCAGAGATGCATCCATCTCGACGA
CGATCCATCCATCTTAAAGCAAGCAGAGACATGGAGTCGTCGCCGCTGCTGCAGCTGCCGGCGGCACGCG
TGGAGGCTCTGAGCCTCAGCGGCCTCTCCGCCATCCCGCCCGAGTACGTCCGCCCCGCCGACGAGCGAGC
CGGCCTCGGGGATGCCTTCGACCTGGCGCGCACCCATGCCAACGACCACACCGCGCCGAGGATCCCCGTC
GTCGACATCTCCCCGTTCCTCGACAGCAGCAGCCAGCAGCAGCAGCGGGACGAATGCGTGGAGGCCGTGC
GTGCCGCCGCCGCCGACTGGGGCGTCATGCACATCGCCGGCCACGGCATCCCCGCCGAGCTCATGGACCG
CCTGCGCGCCGCAGGAACCGCCTTCTTCGCCCTCCCCGTCCAGGACAAGGAGGCCTACGCCAACGACCCC
GCCGCCGGCCGCCTGCAGGGCTACGGCAGCCGCCTCGCCACCAACACCTGCGGGCAGCGCGAGTGGGAGG
ACTACCTCTTCCACCTTGTGCACCCCGACGGGCTCGCCGACCACGCGCTCTGGCCTGCGTACCCGCCCGA
CTACATCGCCGCCACCCGCGACTTCGGCCGCCGAACGCGGGACCTGGCCTCCACGCTGCTCGCCATCCTC
TCCATGGGCCTCCTTGGCACAGACCGTGGCGACGCGCTAGAGAAGGCGCTCACCACCACCACCACCAGGA
CAGCAGCTGACGACGACCTCCTCCTGCAGCTCAAGATCAACTACTACCCGAGGTGCCCGCAGCCGGAGCT
GGCCGTCGGCGTGGAGGCCCACACGGACGTCAGCGCCCTCTCCTTCATCCTCCACAACGGCGTGCCGGGC
CTGCAGGTGCTCCACGGCGCCCGCTGGGTGACGGCGCGCCACGAGCCGGGCACCATCATCGTCCACGTGG
GGGACGCCCTGGAGATCCTCAGCAACGGCCGCTACACCAGCGTCCTCCACCGCGGCCTCGTCAACCGGGA
GGCCGTGCGCATCTCCTGGGTCGTCTTCTGCGAGCCGCCACCAGACTCCGTGCTGCTGCACCCGCTGCCG
GAGCTTGTCACGGAAGGCCACCCCGCAAGGTTCACGCCGCGCACATTCAAGCAGCACCTGGATCGCAAGC
TTTTCAAGAAGAAACAGCAGCACAAAGCAAAAGCAGAGGAAGAGGATGGCGGCAATGGTGACCACCACCG
CCACGAGCCGCCGCCGCAGACCAACTGATGGGCTGCACATGTCTTTCCATCCGCCCACGCATATCTTCTC
TCGCGAAATTAATAAGGATCCATCAGCATTTTCCATATATTTATATTAGTTTCATGCTCCTACGTTACTA
CGAGAAAAAAAATAGTATCTATGATATATAGAATCCATGTGTTAAAATATCACTGTAACACTAATATTAT
ATATGTTGTTACGAAATCAATATAATAAAATAAATTGATATACCAAAACACTAATTATTATTCTATAGTT
TTGTTAGAGTAACTAAAAGTTAAGATTTATATACTTTAAAAGATATATAAGATTGTACCATCACTAGTAA
AAAATAAACAATACCAATATTTTATAAATATGAGTAT
>DQ335126.1_Bz1
ATCGCATTCGCATCGCATCGCAGGTCGCATCCGACGCTAGCGGCTAGCCTAGCCGAACAGCCTGAGCGCG
CGAAGATGGCGCCCGCCGACGGCGAGTCCTCCCCGCCGCCGCACGTGGCCGTGGTCGCCTTCCCGTTCAG
CTCCCACGCGGCGGTGCTGCTCTCCATCGCGCGCGCCCTGGCTGCCGCCGCGGCGCCGTCCGGGGCCACG
CTCTCGTTCCTCTCCACCGCGTCCTCCCTCGCGCAGCTCCGCAAGGCCAGCAGCGCCTCCGCCGGGCACG
GGCTCCCGGGGAACCTGCGCTTCGTCGAGGTACCGGACGGCGCGCCCGCGGCCGAGGAGACCGTGCCGGT
GCCGCGGCAGATGCAGCTGTTCATGGAGGCCGCGGAGGCCGGCGGGGTGAAGGCCTGGCTGGAGGCGGCC
CGCGCCGCGGCGGGCGGCGCCAGGGTGACCTGCGTGGTGGGCGACGCGTTCGTGTGGCCGGCGGCGGACG
CGGCCGCCTCCGCGGGGGCGCCGTGGGTGCCGGTGTGGACGGCCGCGTCGTGCGCGCTCCTGGCGCACAT
CCGCACCGACGCGCTCCGGGAGGACGTTGGCGACCAGGCAGCAAACAGGGTGGACGGGCTACTGATCTCC
CACCCGGGCCTCGCCAGCTACCGCGTCCGTGACCTCCCAGACGGCGTCGTCTCCGGCGACTTCAACTACG
TCATCAACCTCCTCGTCCACCGCATGGGGCAGTGCCTCCCGCGCTCTGCCGCCGCCGTGGCACTCAACAC
GTTCCCAGGCCTGGACCCGCCCGACGTCACCGCGGCGCTCGCGGAGATCCTGCCCAACTGCGTCCCGTTC
GGCCCCTACCACCTCCTCCTCGCCGAGGACGACGCCGACACCGCCGCACCAGCCGACCCGCACGGCTGCC
TCGCCTGGCTGGGCCGCCAACCCGCGCGCGGCGTCGCGTACGTCAGCTTCGGCACGGTGGCGTGCCCGCG
GCCCGACGAGCTCCGCGAGCTGGCGGCCGGGCTGGAGGACTCGGGCGCGCCGTTCCTGTGGTCGCTGCGC
GAGGACTCGTGGCCGCACCTCCCGCCGGGTTTCCTGGACCGCGCCGCGGGCACCGGGTCCGGGCTCGTGG
TGCCCTGGGCGCCGCAGGTGGCCGTGCTGCGCCACCCTTCCGTGGGCGCGTTCGTGACGCACGCCGGGTG
GGCGTCGGTGCTGGAGGGCTTGTCCAGCGGGGTGCCCATGGCGTGCCGCCCCTTCTTCGGCGACCAGCGG
ATGAACGCGCGGTCCGTGGCGCACGTGTGGGGGTTCGGCGCCGCGTTCGAGGGCGCTATGACGAGCGCCG
GAGTGGCCACGGCCGTGGAGGAGCTGCTGCGCGGGGAGGAAGGGGCGCGGATGAGGGCAAGGGCCAAGGA
GCTGCAGGCCTTGGTGGCCGAGGCGTTCGGGCCAGGCGGTGAGTGCAGGAAGAACTTCGACAGGTTCGTC
GAGATAGTCTGTCGCGCGTGAAAGGTCGTCTTGCTGTTCAGAGGTTTTACCAACAGAAGAACATAATGAA
TTGGATGGCATGCTACGTCGTATTCTCTTTTTTTGTTGATCCCTGAGTTGATACATTTTGTACTTGATAC

ATGAGTTGCAGCAGCAGCAGCAACAGCCTTCTGTACCTTGGCTTTGGATCTGTATTCTTGTCACCAGTTA
TCTGAAAGCATCAATAACCTTCTGTCTTCTAAAAAAAAAAAAAAAA
>AF377543.1_B73z2
ACAGGCTCCTCGCTGCCAACCCCGTCTACGGGAAGATCCCCGTGCTGCTCCTCCCCGACGGCCGCGCCAT
ATGCGAGTCCGCAGTCATCATCCAGTGCATCGAGGACGTGGCGCGTGGAAGCGGCGGCGCCGAGGCTAGC
AGCCTGCTGCTGCCGGACGACCCCTACGAGCGCGCCATGCACCGCTTCTGGACCGCCTTCATCGACGACA
AGGTGAGCACCGAGCAGAGCACGGCAGCAAGTGTCTTTTCTTCAAGGTAACGTGCAACAAACTGCCGTGC
CTTTCTGCAGTTTTGGCCGGCGCTGGATGCCGTCTCCCTGGCGCCGACCCCGGGAGCACGCGCGCAGGCC
GTGGAAGACACCCGCGCCGCGCTGAGCCTCCTGGAGGTGGCGTTCAAGGACCGCAGCAACGGCAGGGCTT
TCTTCTCCGGTGGCGACGCCGCGCCAGGCCTCCTGGACCTGGCCCTCGGATGCTTCCTGCCGGCGCTCAG
GGCCTGCGAACGGCTCCACGGCCTCTCACTCATCGACGCGTCCGCGACGCCGCTGCTGGACGGGTGGAGC
CAGCGCTTCGCCGCGCACCCTGCAGCCAAGCGCGTCTGCCGGACA
