# Supplementary material for: Promzea: a pipeline for discovery of co-regulatory motifs in maize and other plant species and its application to the anthocyanin and phlobaphene biosynthetic pathways and the Maize Development Atlas
Source: BMC Plant Biol. 2013 Mar 15;13:42. doi: 10.1186/1471-2229-13-42 (PMC3658923; doi:10.1186/1471-2229-13-42)
Supplement: Additional file 6 — Promzea output for searches of the maize genome with the anthocyanin/phlobaphene-related motifs predicted by Promzea. Shown is the user output from the Promzea website or command line. [file 1471-2229-13-42-S6.pdf]

Home

Results Summary

./input/anthocyanin_pathway.fas

Promzea - 00000496

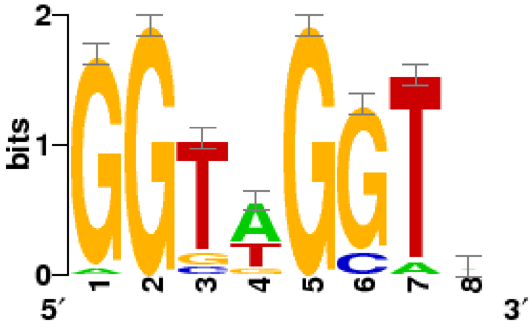

Motif1
(from Weeder)
score: 1.8

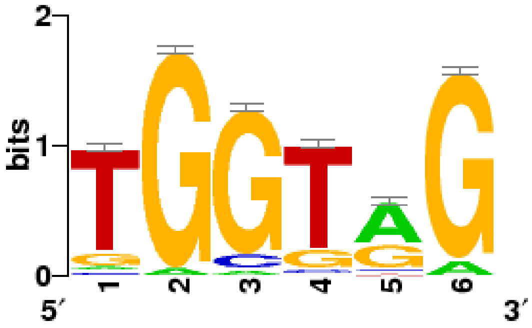

Motif2
(from Weeder)
score: 1.71

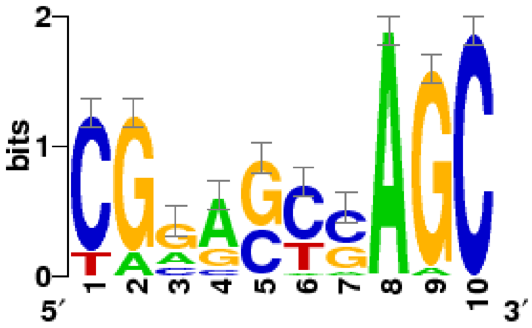

Motif3
(from BioP)
score: 1.42

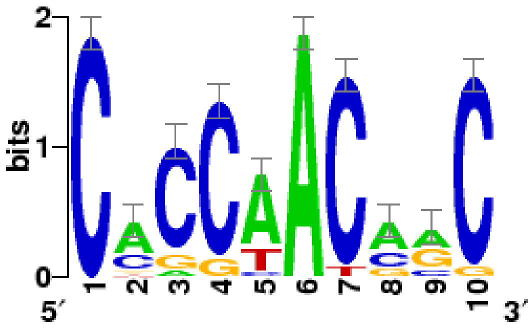

Motif4
(from MEME)
score: 1.27

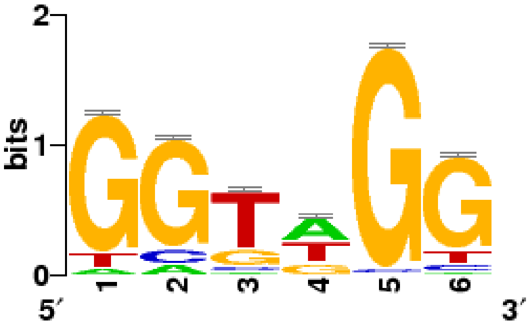

Motif5
(from Weeder)
score: 1.26

Compare your motifs to known promoter motif databases using STAMP website motif file to copy in STAMP website

Open the above link, copy content of the newly open file and paste in STAMP program link below In STAMP, under "Similarity Matching", we suggest selecting the plant motif databases: Athamap, AGRIS, PLACE, TRANSFAC; then submit

STAMP website

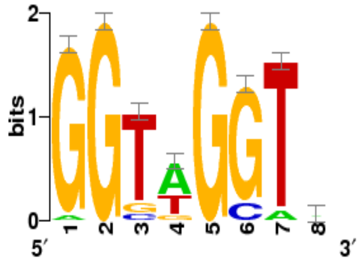

GGTAGGTA

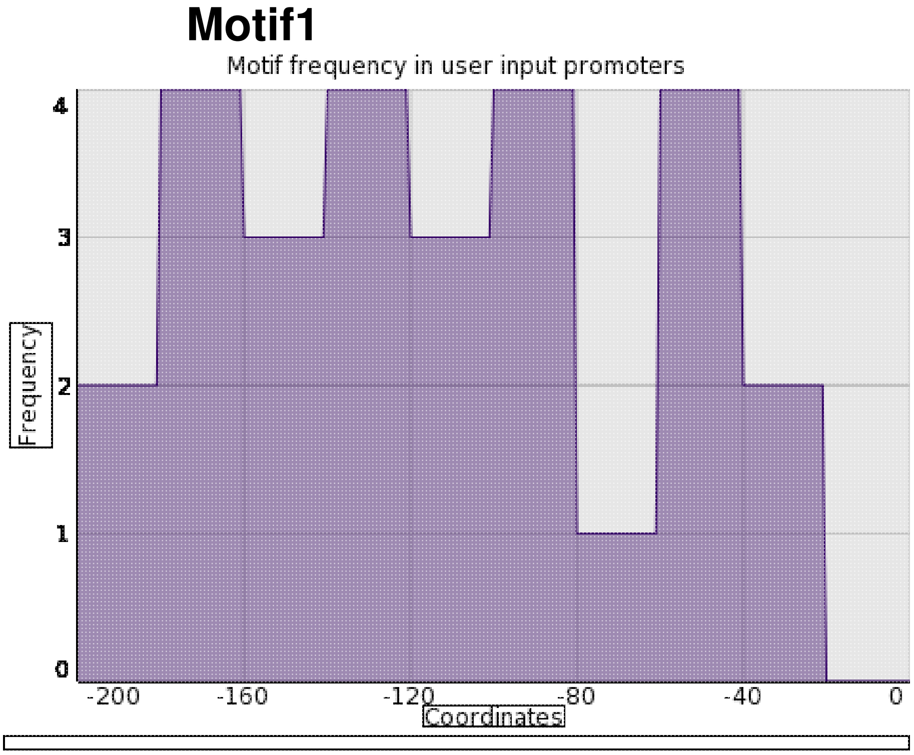

Motif1 annotation in the genome

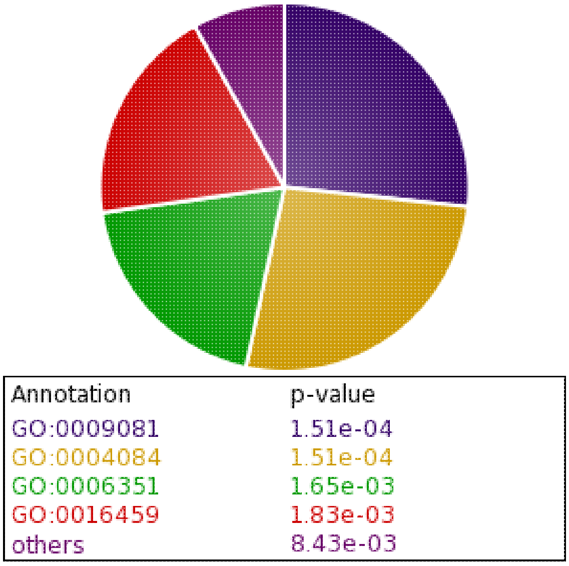

Annotation complete description

GO:0009081 => branched chain family amino acid metabolic process GO:0004084 => branched-chain-amino-acid transaminase activity GO:0006351 => transcription, DNA-dependent GO:0016459 => myosin complex GO:0003774 => motor activity GO:0016655 => oxidoreductase activity, acting on NADH or NADPH, quinone or similar compound as acceptor GO:0005971 => ribonucleoside-diphosphate reductase complex

Genome-wide Motif1 search results

Motif1 gene list of over-represented annotation(s)

Motif2

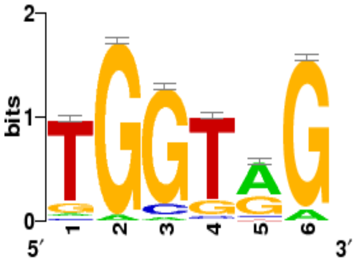

TGGTAG

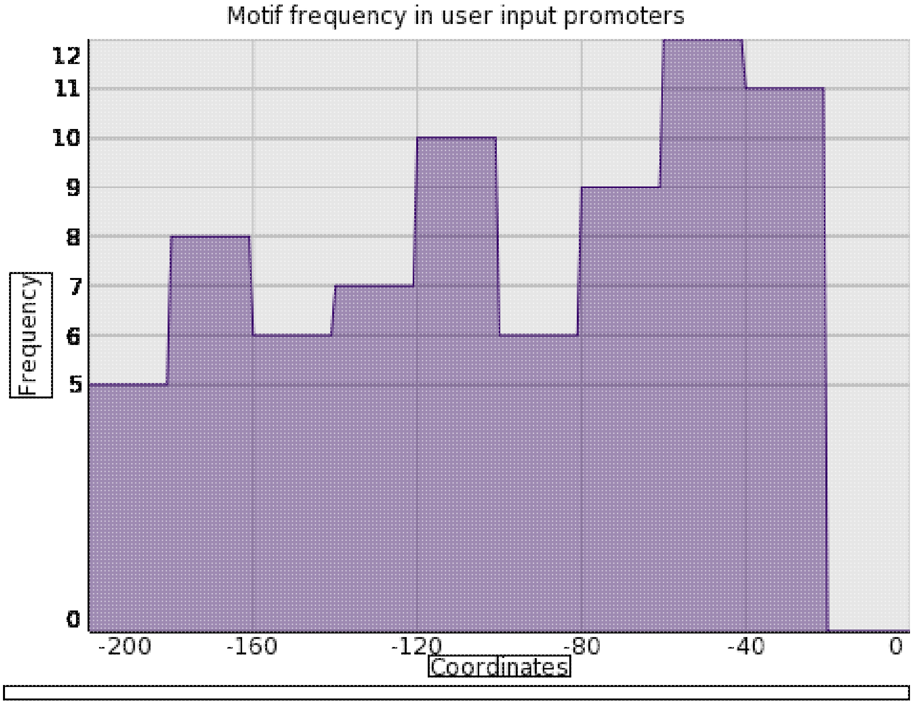

Motif2 annotation in the genome

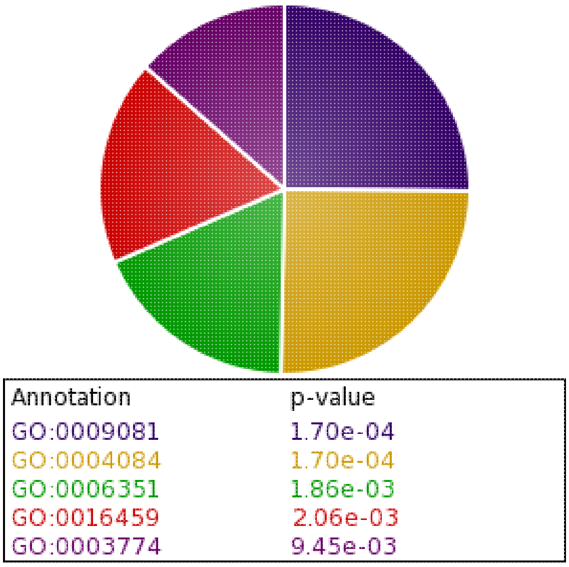

Annotation complete description

GO:0009081 => branched chain family amino acid metabolic process GO:0004084 => branched-chain-amino-acid transaminase activity GO:0006351 => transcription, DNA-dependent GO:0016459 => myosin complex GO:0003774 => motor activity

Genome-wide Motif2 search results

Motif2 gene list of over-represented annotation(s)

Motif3

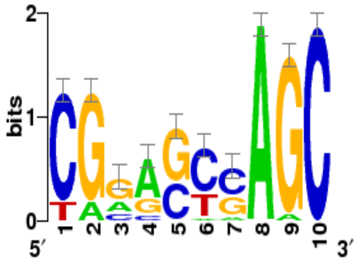

CGGAGCCAGC

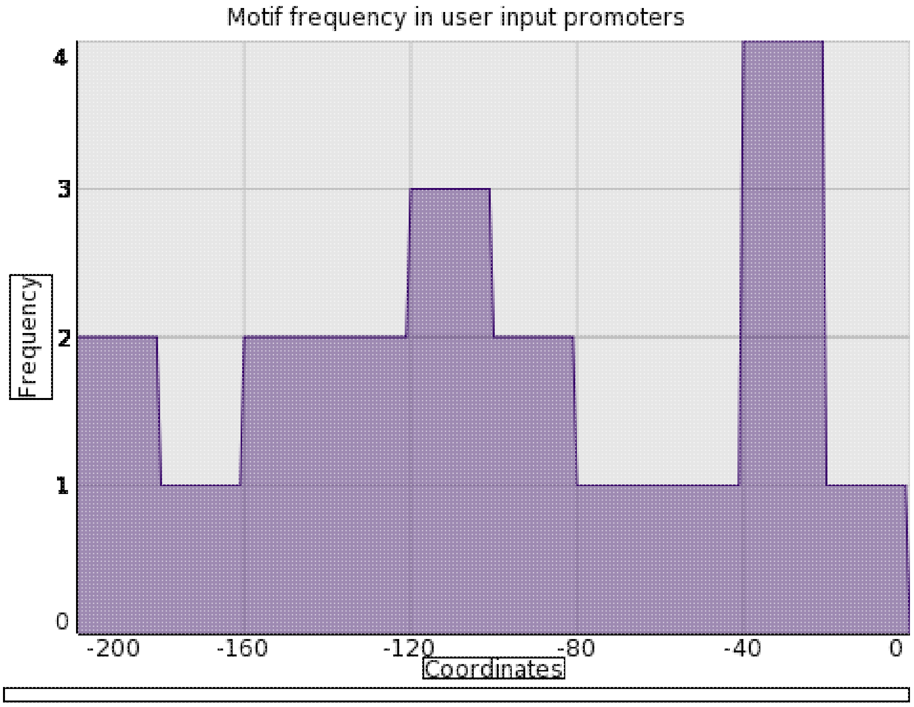

Motif3 annotation in the genome

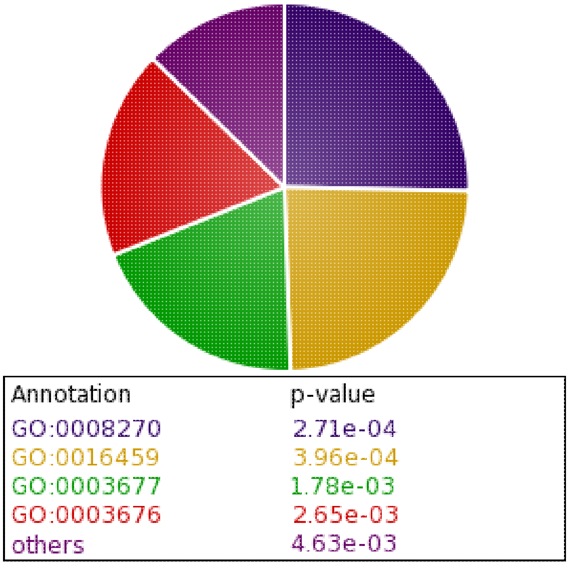

Annotation complete description

GO:0008270 => zinc ion binding GO:0016459 => myosin complex GO:0003677 => DNA binding GO:0003676 => nucleic acid binding GO:0009081 => branched chain family amino acid metabolic process GO:0004084 => branched-chain-amino-acid transaminase activity GO:0016987 => sigma factor activity

Genome-wide Motif3 search results

Motif3 gene list of over-represented annotation(s)

Motif4

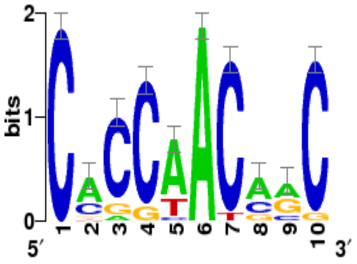

CACCAACAAC

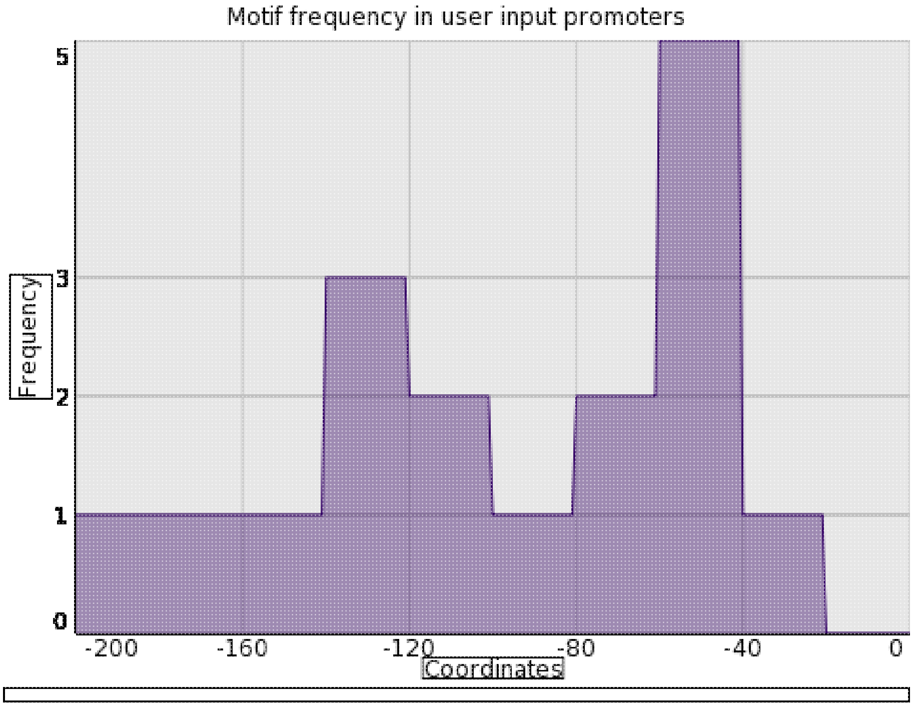

Motif4 annotation in the genome

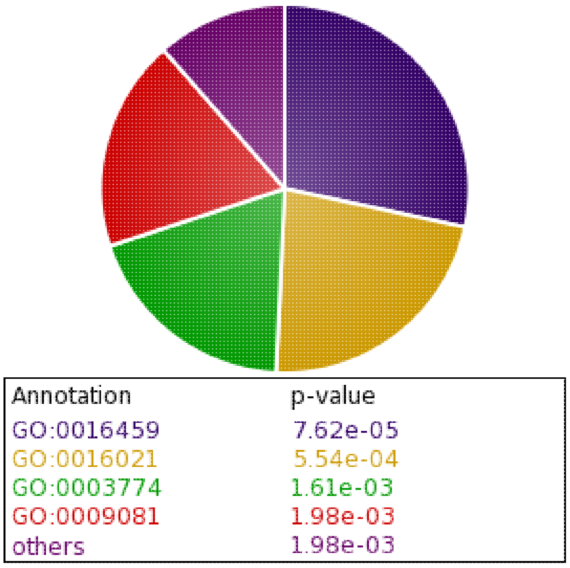

Annotation complete description

GO:0016459 => myosin complex GO:0016021 => integral to membrane GO:0003774 => motor activity GO:0009081 => branched chain family amino acid metabolic process GO:0004084 => branched-chain-amino-acid transaminase activity GO:0008199 => ferric iron binding GO:0031072 => heat shock protein binding

Genome-wide Motif4 search results

Motif4 gene list of over-represented annotation(s)

Motif5

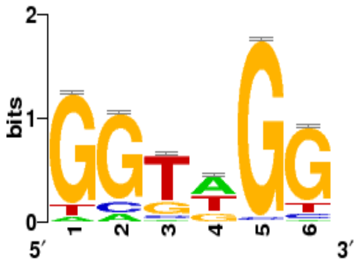

GGTAGG

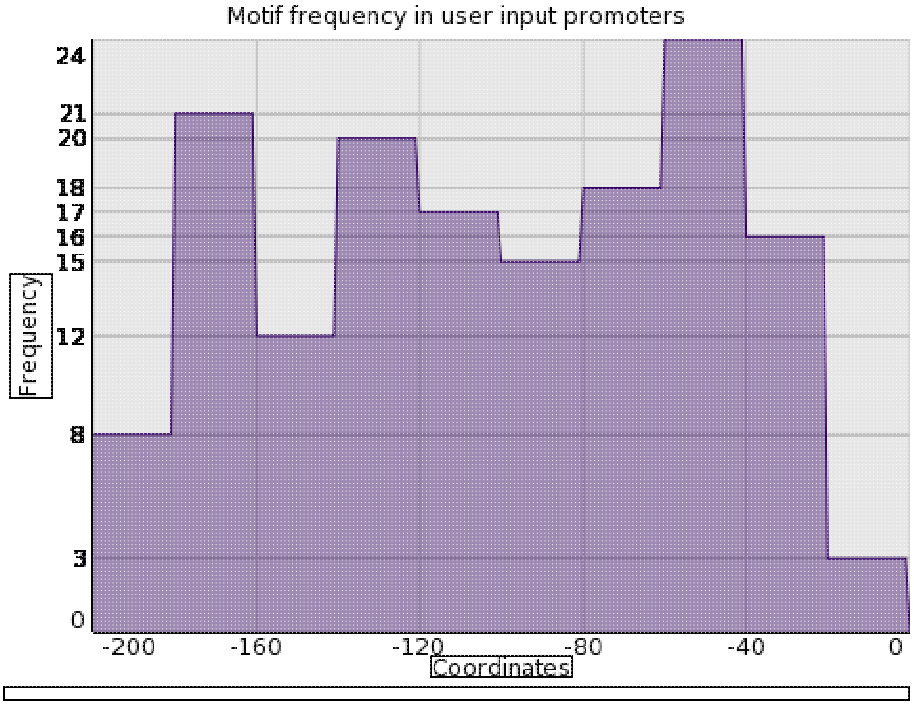

Motif5 annotation in the genome

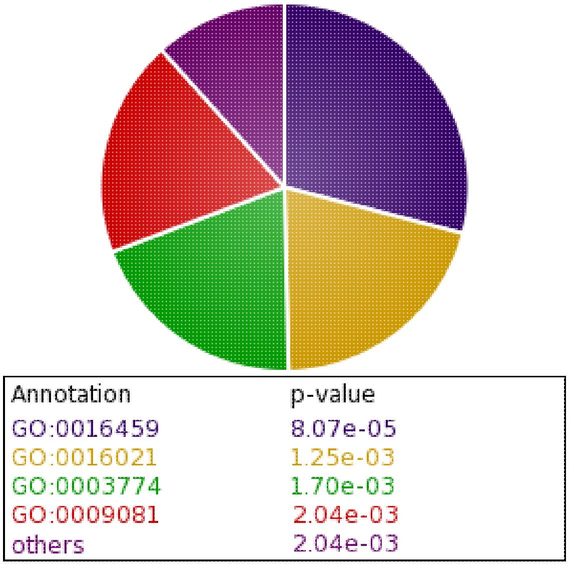

Annotation complete description

GO:0016459 => myosin complex GO:0016021 => integral to membrane GO:0003774 => motor activity GO:0009081 => branched chain family amino acid metabolic process GO:0004084 => branched-chain-amino-acid transaminase activity GO:0008199 => ferric iron binding GO:0031072 => heat shock protein binding

Genome-wide Motif5 search results

Motif5 gene list of over-represented annotation(s)

Sequence logo generated by weblogo
Graphic generated with Chart::Clicker,Perl module
Promzea program from the Raizada lab
