# Supplementary material for: Promzea: a pipeline for discovery of co-regulatory motifs in maize and other plant species and its application to the anthocyanin and phlobaphene biosynthetic pathways and the Maize Development Atlas
Source: BMC Plant Biol. 2013 Mar 15;13:42. doi: 10.1186/1471-2229-13-42 (PMC3658923; doi:10.1186/1471-2229-13-42)
Supplement: Additional file 7 — Supplemental files for testing Promzea with data sets from the Maize Development Atlas. The zip folder contains 3 folders. The first contains the promoter input for Promzea for each maize tissue; the second folder has all the outputs from Promzea; the third folder contains the STAMP website outputs for comparisons of the predicted motifs with experimentally defined motifs. [file 1471-2229-13-42-S7.zip › Supplemental files 3 -Case study 3/2-Promzea results/embryo.pdf]

Home

Results Summary

/vbox_shared/1-case_study_3/casestudy3_embryo.txt

Promzea - 00000443

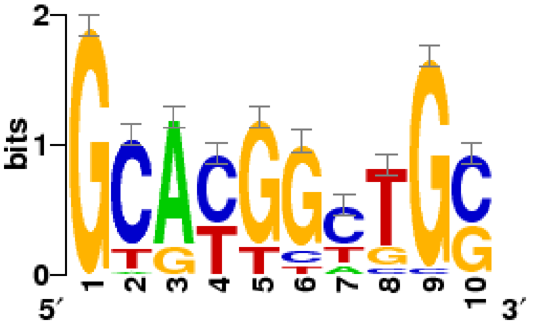

Motif1
(from MEME)
score: 1.76

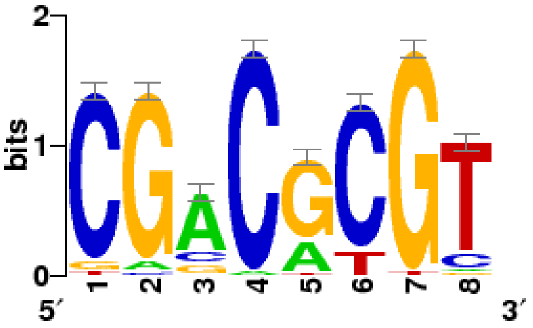

Motif2
(from Weeder)
score: 1.72

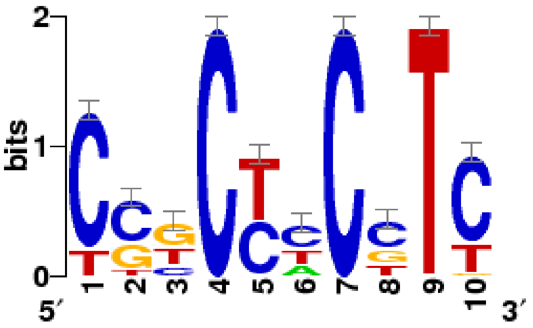

Motif3
(from BioP)
score: 1.52

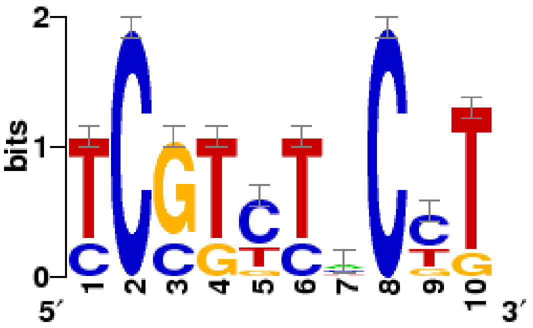

Motif4
(from BioP)
score: 1.43

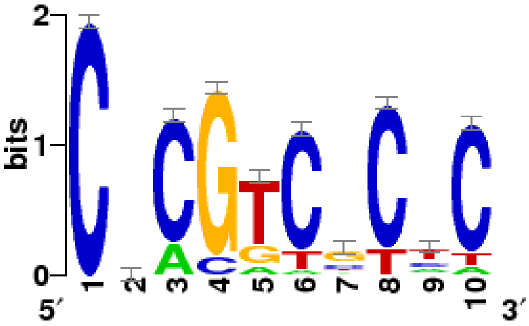

Motif5
(from MEME)
score: 1.19

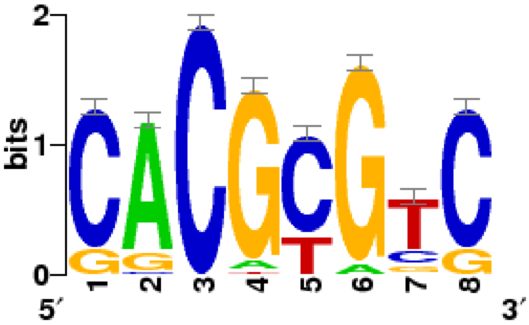

Motif6
(from Weeder)
score: 1.09

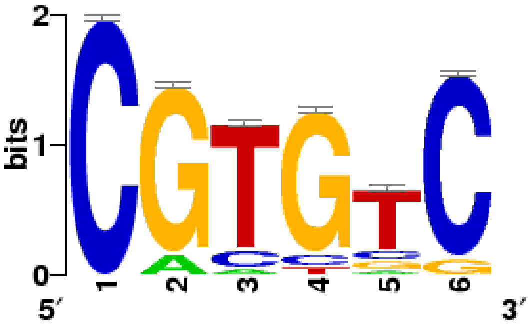

Motif7
(from Weeder)
score: 1.09

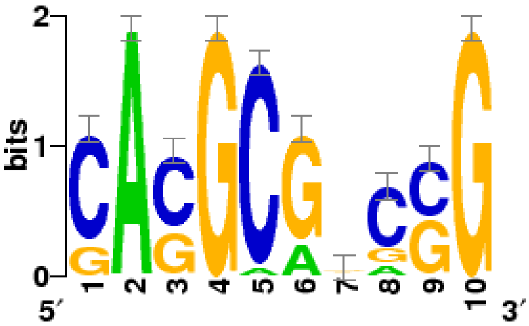

Motif8
(from MEME)
score: 1.07

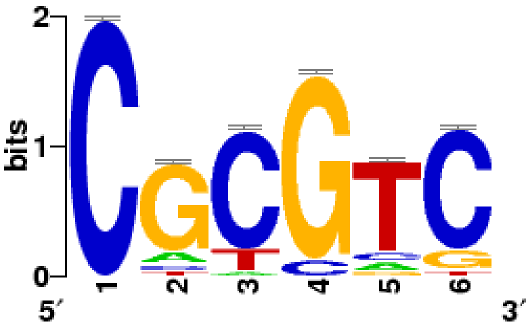

Motif9
(from Weeder)
score: 1.07

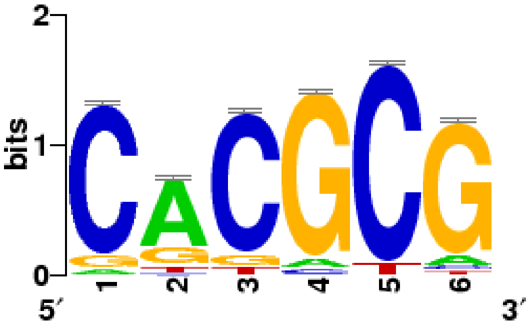

Motif10
(from Weeder)
score: 1.07

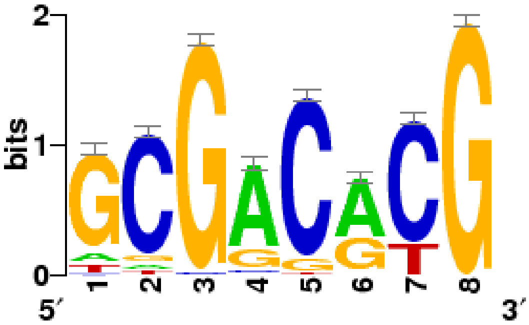

Motif11
(from Weeder)
score: 1.06

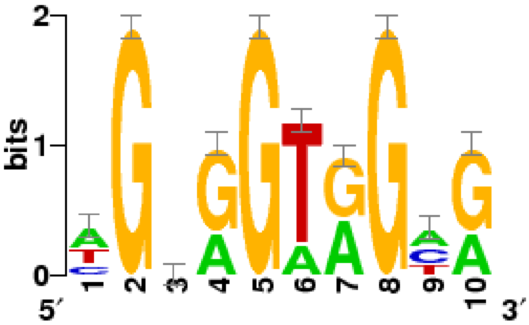

Motif12
(from BioP)
score: 1.06

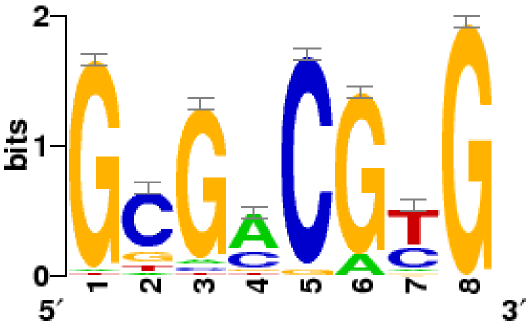

Motif13
(from Weeder)
score: 1.05

Compare your motifs to known promoter motif databases using STAMP website motif file to copy in STAMP website

Open the above link, copy content of the newly open file and paste in STAMP program link below In STAMP, under "Similarity Matching", we suggest selecting the plant motif databases: Athamap, AGRIS, PLACE, TRANSFAC; then submit

STAMP website

Motif1

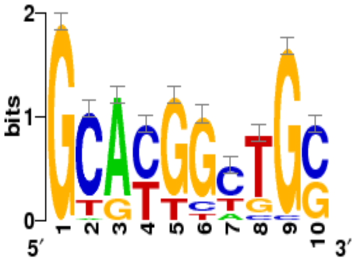

GCACGGCTGC

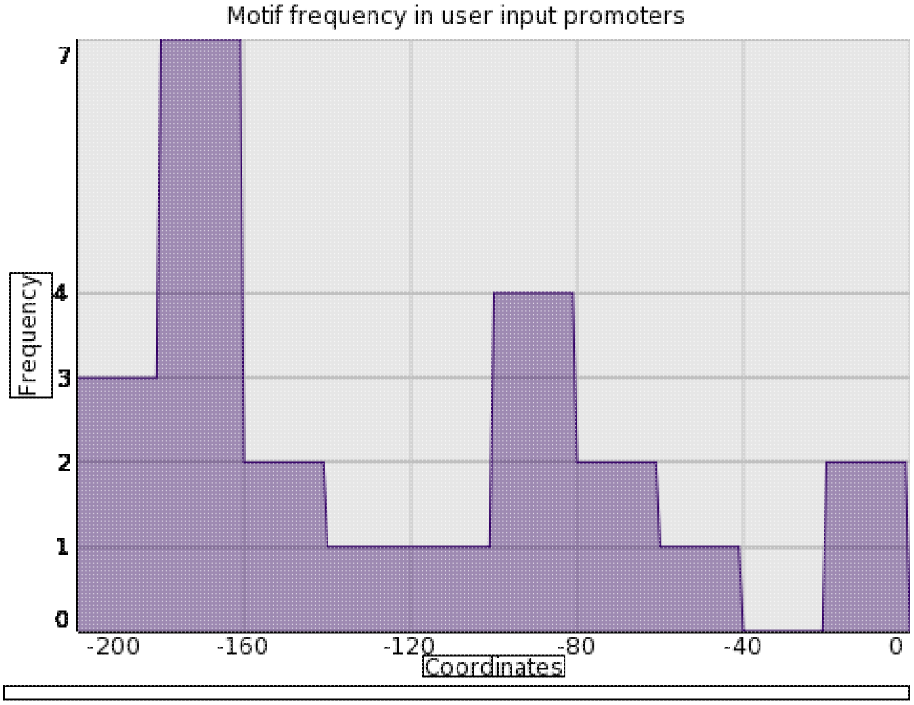

Motif1 annotation in the genome

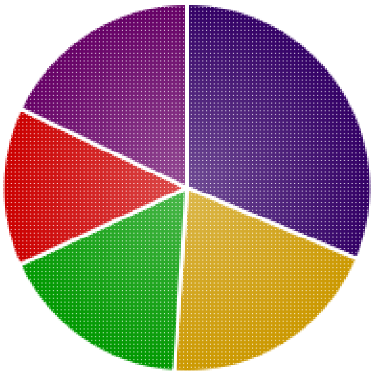

Annotation	p-value
GO:0003677	2.91e-10
GO:0046983	9.15e-07
GO:0003676	6.45e-06
GO:0008270	5.77e-05
others	1.07e-04

Annotation complete description

GO:0003677 => DNA binding GO:0046983 => protein dimerization activity GO:0003676 => nucleic acid binding GO:0008270 => zinc ion binding GO:0045735 => nutrient reservoir activity GO:0005840 => ribosome GO:0048046 => apoplast

Genome-wide Motif1 search results
Motif1 gene list of over-represented annotation(s)

Motif2

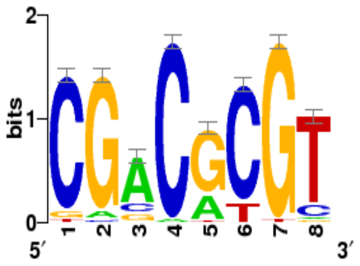

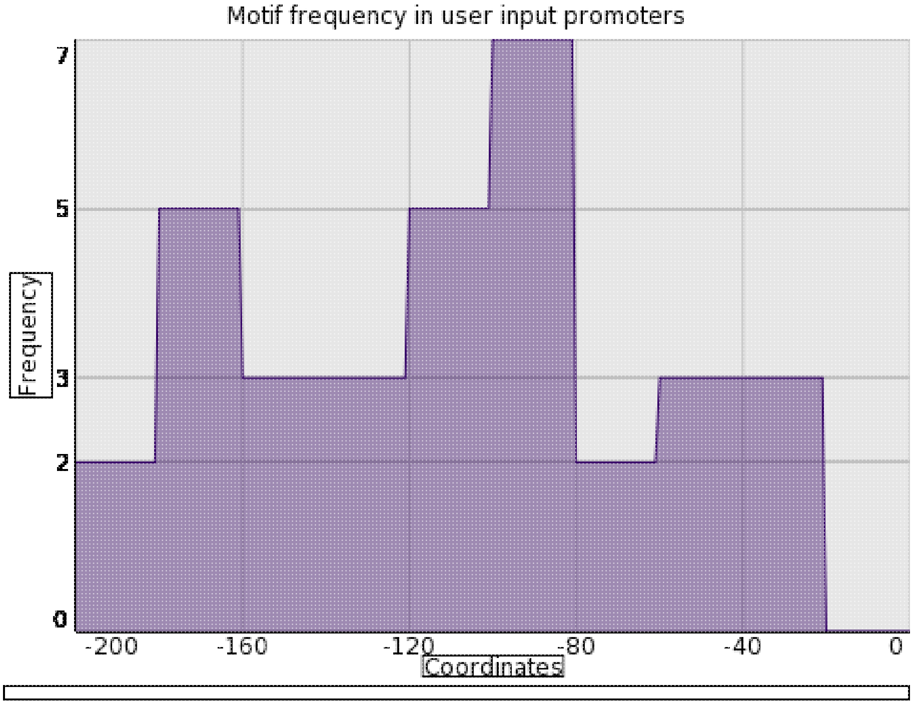

CGACGCGT

Motif2 annotation in the genome

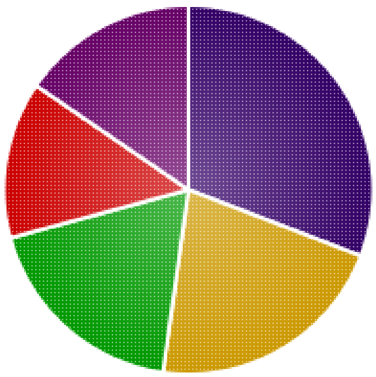

Annotation	p-value
GO:0003677	1.32e-11
GO:0046983	2.88e-08
GO:0003676	2.77e-07
GO:0008270	1.41e-05
others	2.75e-04

Annotation complete description

GO:0003677 => DNA binding GO:0046983 => protein dimerization activity GO:0003676 => nucleic acid binding GO:0008270 => zinc ion binding GO:0045735 => nutrient reservoir activity GO:0004707 => MAP kinase activity GO:0048046 => apoplast

Genome-wide Motif2 search results

Motif2 gene list of over-represented annotation(s)

Motif3

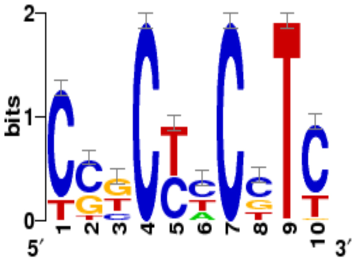

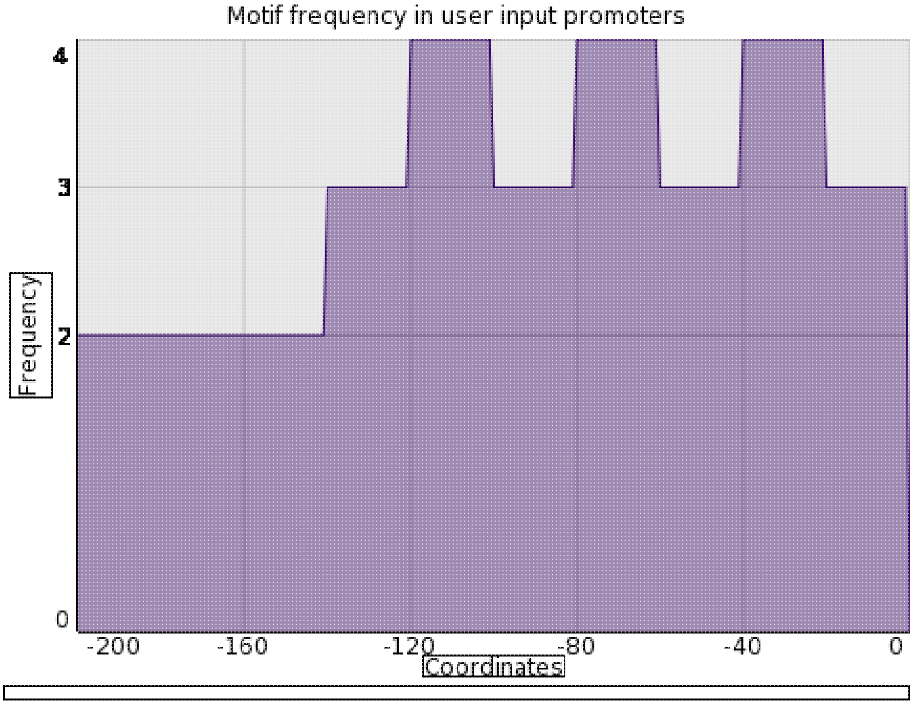

CCGCTCCCTC

Motif3 annotation in the genome

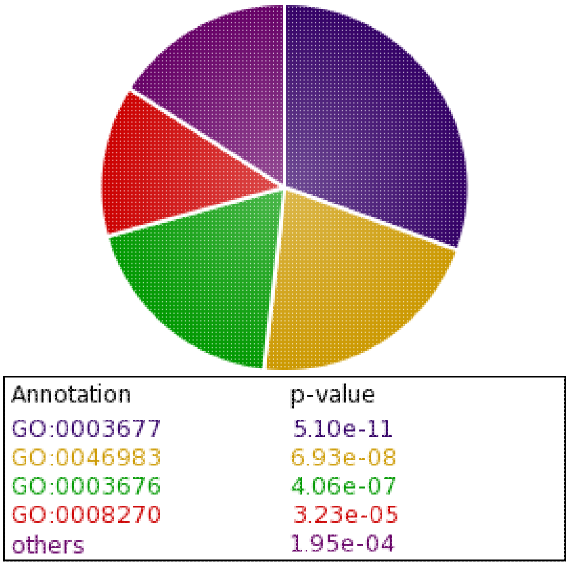

Annotation complete description

GO:0003677 => DNA binding GO:0046983 => protein dimerization activity GO:0003676 => nucleic acid binding GO:0008270 => zinc ion binding GO:0045735 => nutrient reservoir activity GO:0004707 => MAP kinase activity GO:0048046 => apoplast

Genome-wide Motif3 search results

Motif3 gene list of over-represented annotation(s)

Motif4

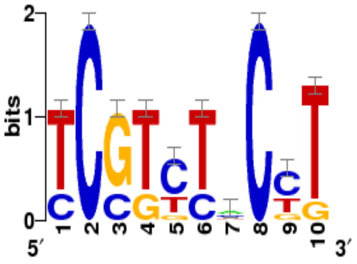

TCGTCTACCT

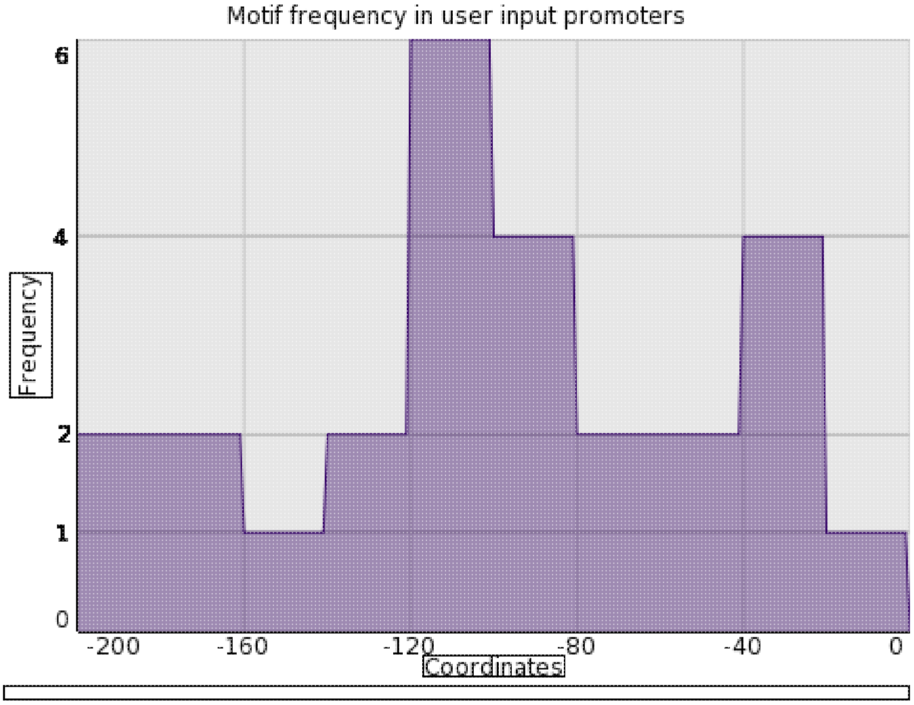

Motif4 annotation in the genome

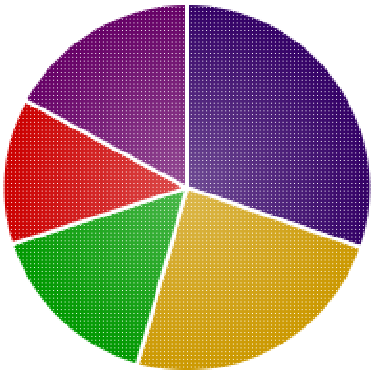

Annotation	p-value
GO:0003677	1.06e-08
GO:0046983	4.51e-07
GO:0008270	7.43e-05
GO:0015995	4.09e-04
others	6.86e-04

Annotation complete description

GO:0003677 => DNA binding GO:0046983 => protein dimerization activity GO:0008270 => zinc ion binding GO:0015995 => chlorophyll biosynthetic process GO:0048046 => apoplast GO:0006511 => ubiquitin-dependent protein catabolic process GO:0003676 => nucleic acid binding

Genome-wide Motif4 search results

Motif4 gene list of over-represented annotation(s)

Motif5

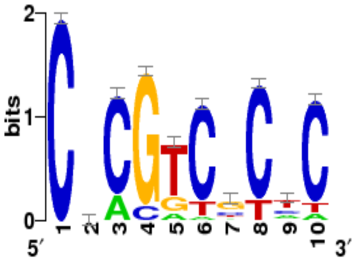

CACGTCGCTC

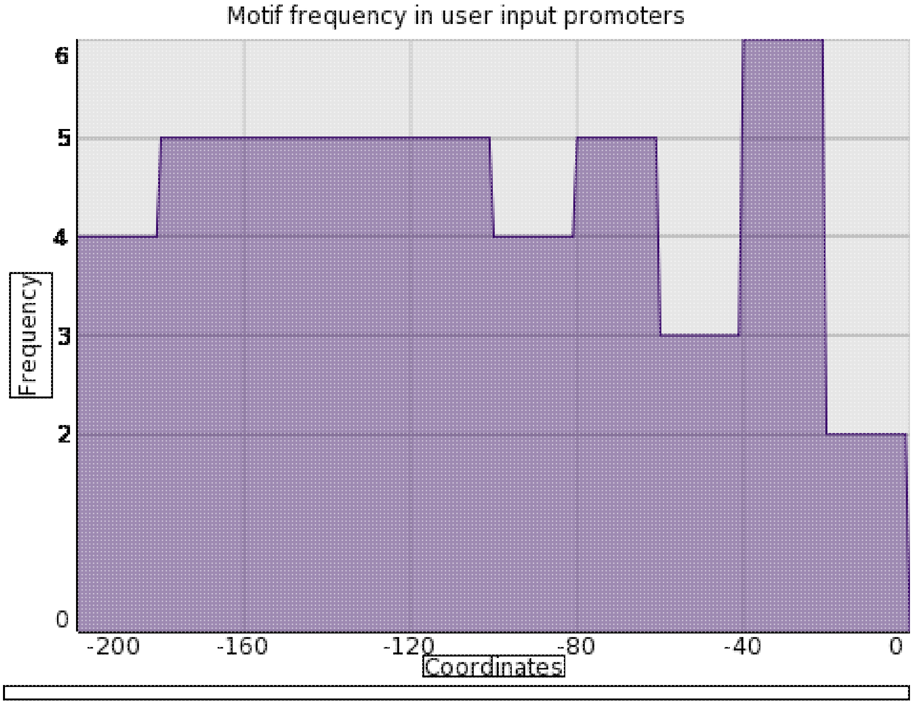

Motif5 annotation in the genome

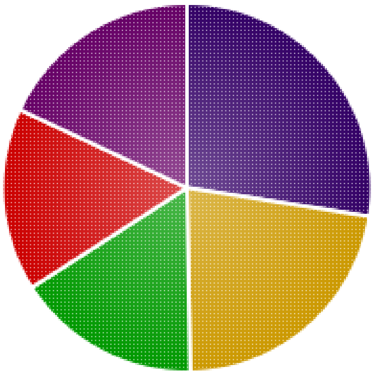

Annotation	p-value
GO:0003677	2.44e-07
GO:0046983	4.43e-06
GO:0048046	1.27e-04
GO:0008270	1.37e-04
others	1.86e-04

Annotation complete description

GO:0003677 => DNA binding GO:0046983 => protein dimerization activity GO:0048046 => apoplast GO:0008270 => zinc ion binding GO:0045735 => nutrient reservoir activity GO:0004707 => MAP kinase activity GO:0003676 => nucleic acid binding

Genome-wide Motif5 search results

Motif5 gene list of over-represented annotation(s)

Motif6

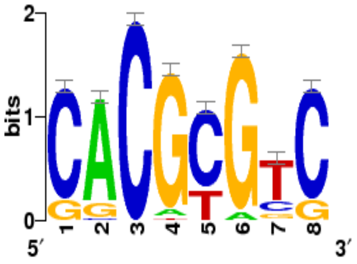

CACGCGTC

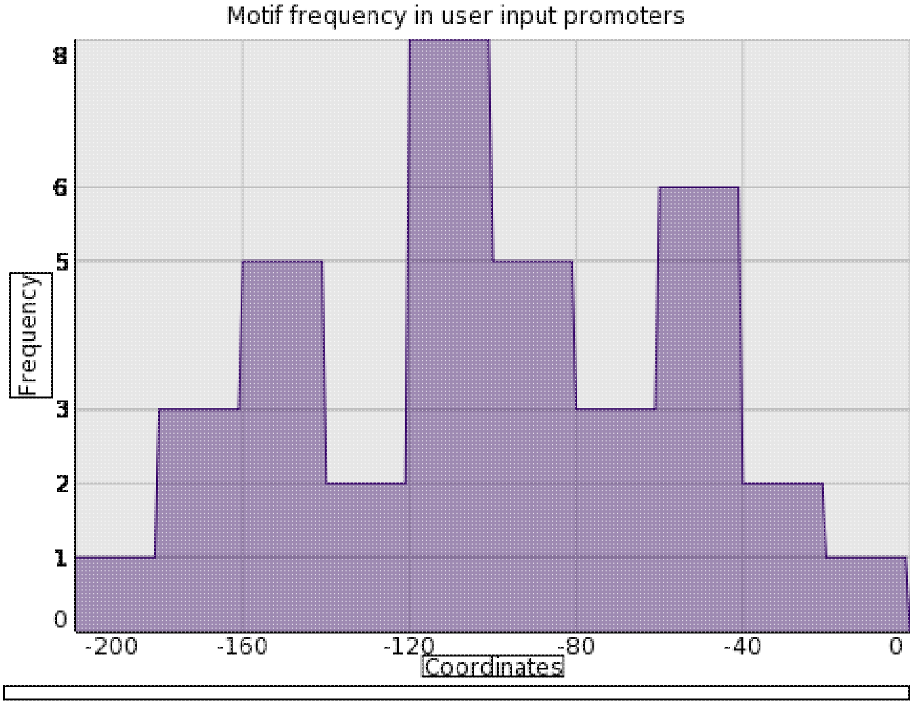

Motif6 annotation in the genome

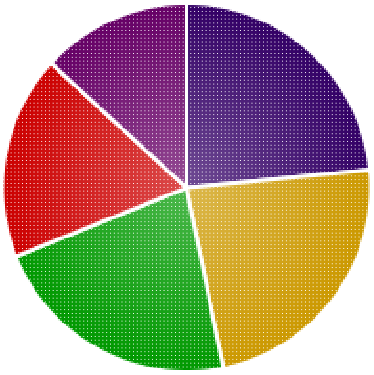

Annotation	p-value
GO:0048046	9.90e-06
GO:0045735	1.05e-05
GO:0030145	1.91e-05
GO:0003677	1.51e-04
others	5.15e-04

Annotation complete description

GO:0048046 => apoplast GO:0045735 => nutrient reservoir activity GO:0030145 => manganese ion binding GO:0003677 => DNA binding GO:0007205 => activation of protein kinase C activity by G-protein coupled receptor protein signaling pathway GO:0004143 => diacylglycerol kinase activity GO:0008270 => zinc ion binding

Genome-wide Motif6 search results

Motif6 gene list of over-represented annotation(s)

Motif7

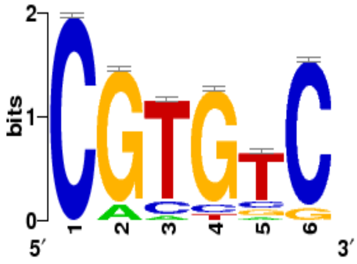

CGTGTC

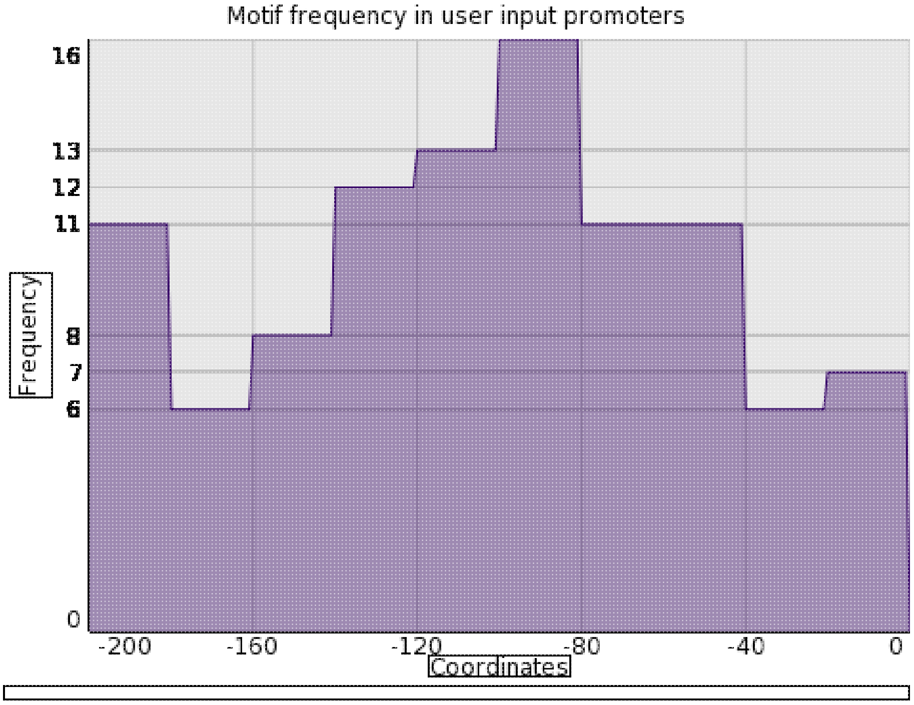

Motif7 annotation in the genome

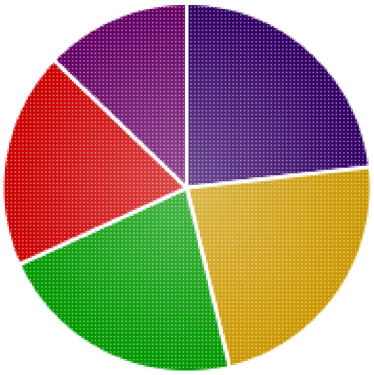

Annotation	p-value
GO:0048046	1.17e-05
GO:0045735	1.25e-05
GO:0030145	2.19e-05
GO:0003677	9.15e-05
others	2.39e-04

Annotation complete description

GO:0048046 => apoplast GO:0045735 => nutrient reservoir activity GO:0030145 => manganese ion binding GO:0003677 => DNA binding GO:0004707 => MAP kinase activity GO:0007205 => activation of protein kinase C activity by G-protein coupled receptor protein signaling pathway GO:0004143 => diacylglycerol kinase activity

Genome-wide Motif7 search results

Motif7 gene list of over-represented annotation(s)

Motif8

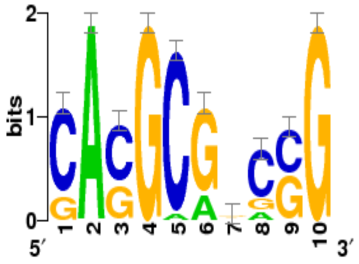

CACGCGGCGG

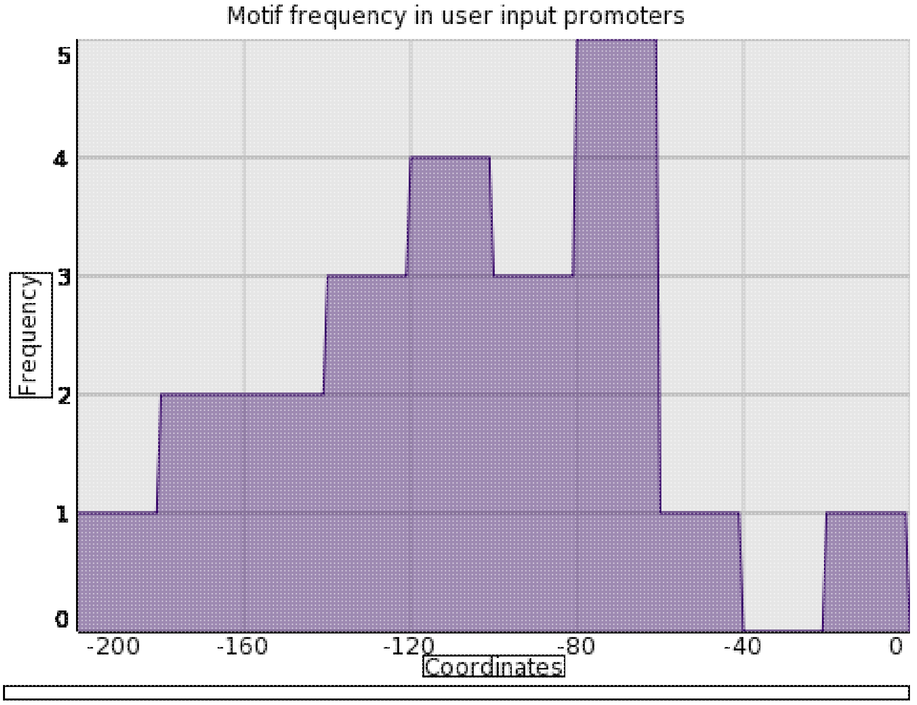

Motif8 annotation in the genome

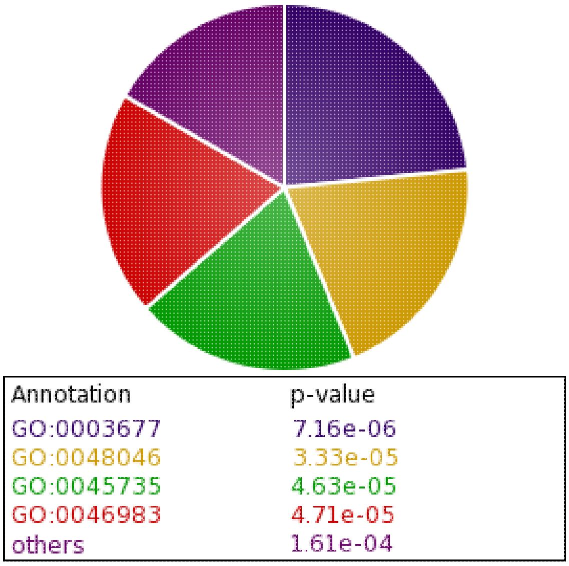

Annotation complete description

GO:0003677 => DNA binding GO:0048046 => apoplast GO:0045735 => nutrient reservoir activity GO:0046983 => protein dimerization activity GO:0004707 => MAP kinase activity GO:0030145 => manganese ion binding GO:0008270 => zinc ion binding

Genome-wide Motif8 search results

Motif8 gene list of over-represented annotation(s)

Motif9

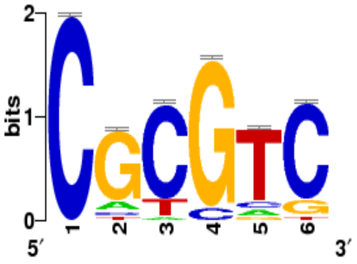

CGCGTC

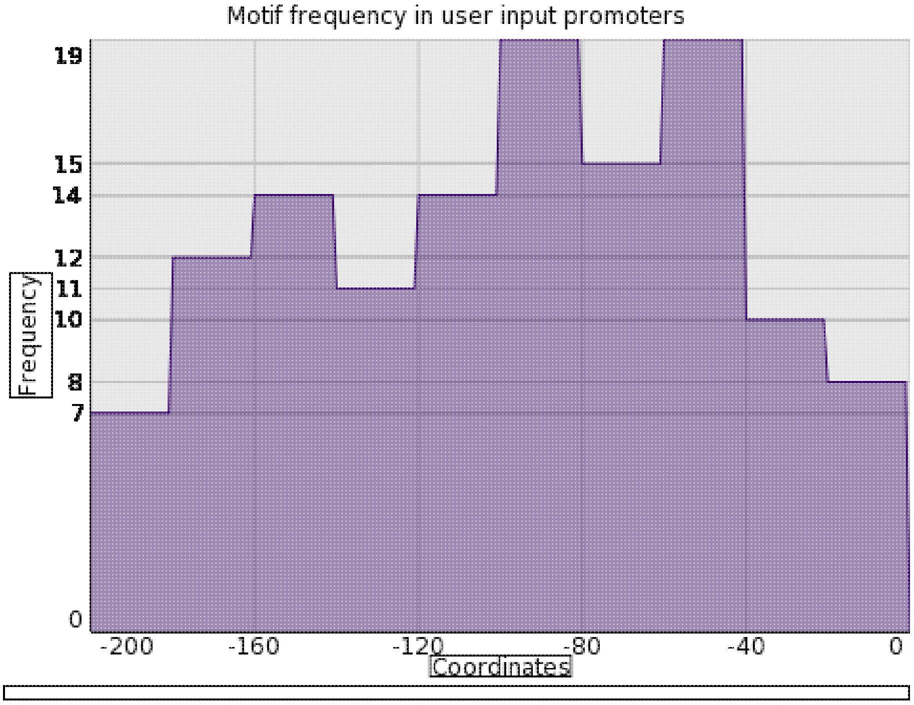

Motif9 annotation in the genome

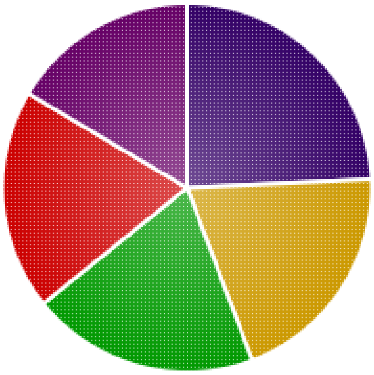

Annotation	p-value
GO:0003677	4.26e-06
GO:0048046	3.77e-05
GO:0046983	3.87e-05
GO:0045735	5.27e-05
others	1.74e-04

Annotation complete description

GO:0003677 => DNA binding GO:0048046 => apoplast GO:0046983 => protein dimerization activity GO:0045735 => nutrient reservoir activity GO:0004707 => MAP kinase activity GO:0030145 => manganese ion binding GO:0003676 => nucleic acid binding

Genome-wide Motif9 search results

Motif9 gene list of over-represented annotation(s)

Motif10

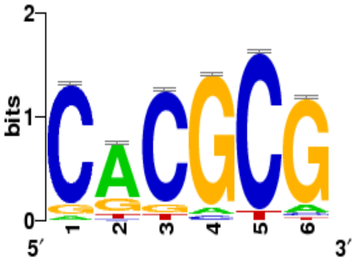

CACGCG

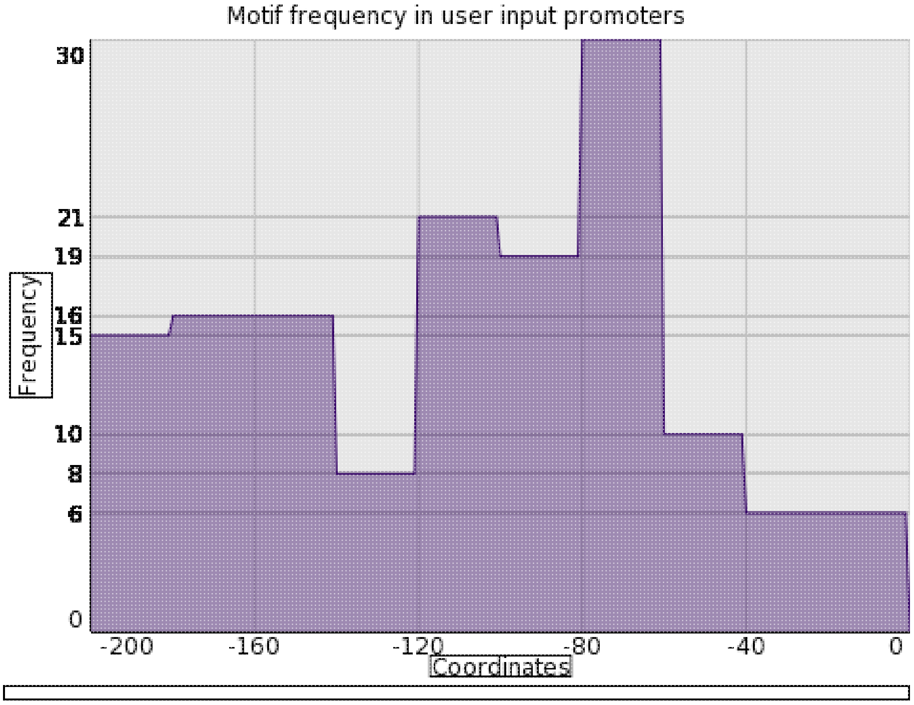

Motif10 annotation in the genome

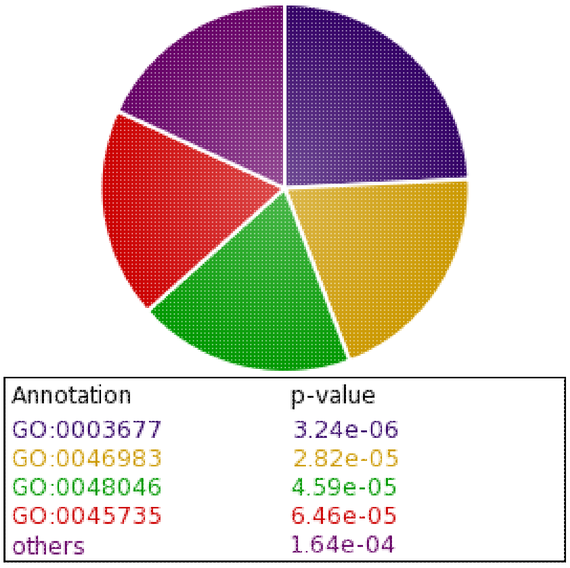

Annotation complete description

GO:0003677 => DNA binding GO:0046983 => protein dimerization activity GO:0048046 => apoplast GO:0045735 => nutrient reservoir activity GO:0008270 => zinc ion binding GO:0003676 => nucleic acid binding GO:0004707 => MAP kinase activity

Genome-wide Motif10 search results

Motif10 gene list of over-represented annotation(s)

Motif11

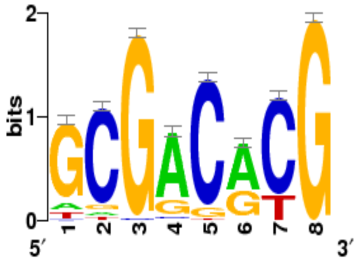

GCGACACG

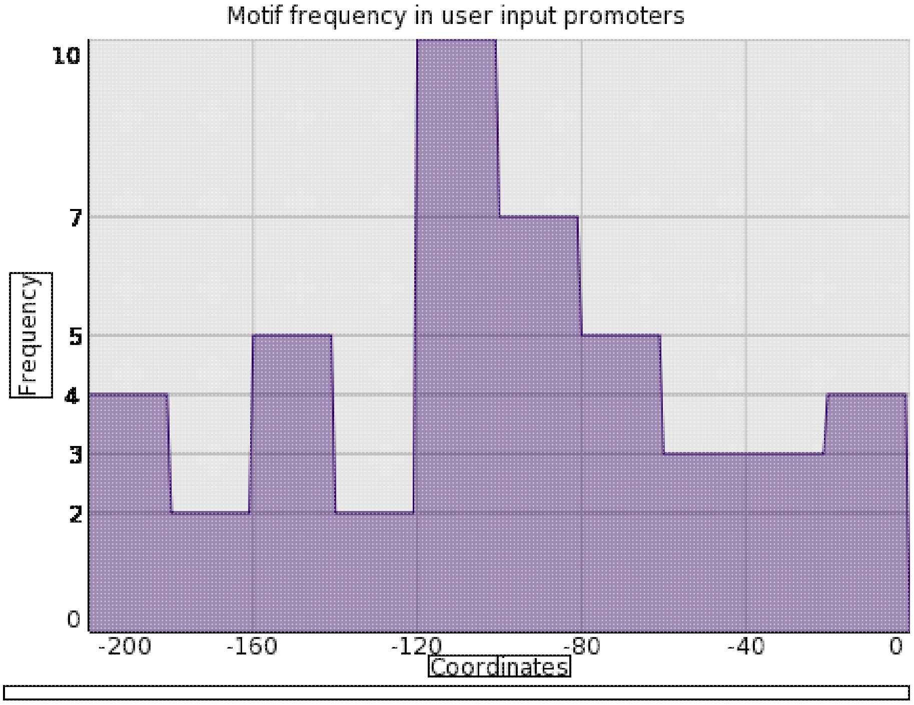

Motif11 annotation in the genome

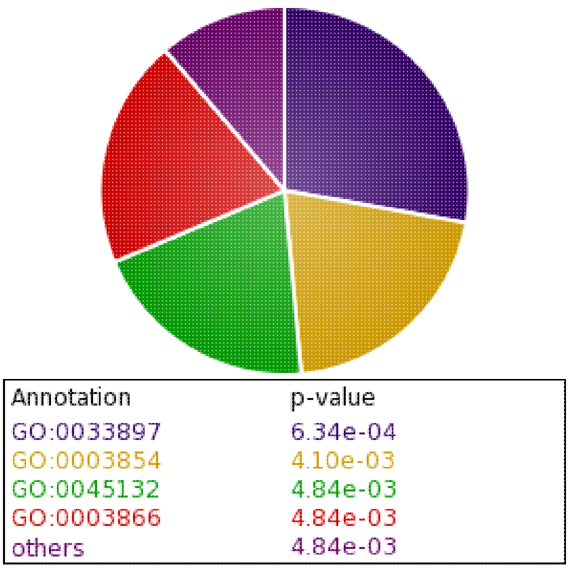

Annotation complete description

GO:0033897 => ribonuclease T2 activity GO:0003854 => 3-beta-hydroxy-delta5-steroid dehydrogenase activity GO:0045132 => meiotic chromosome segregation GO:0003866 => 3-phosphoshikimate 1-carboxyvinyltransferase activity GO:0050515 => 4-(cytidine 5'-diphospho)-2-C-methyl-D-erythritol kinase activity GO:0071266 => 'de novo' L-methionine biosynthetic process GO:0004121 => cystathionine beta-lyase activity

Genome-wide Motif11 search results

Motif11 gene list of over-represented annotation(s)

Motif12

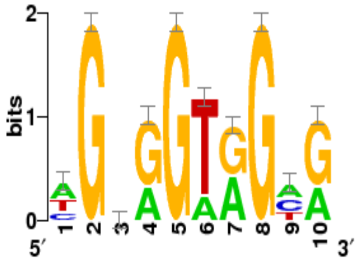

AGAGGTGGAG

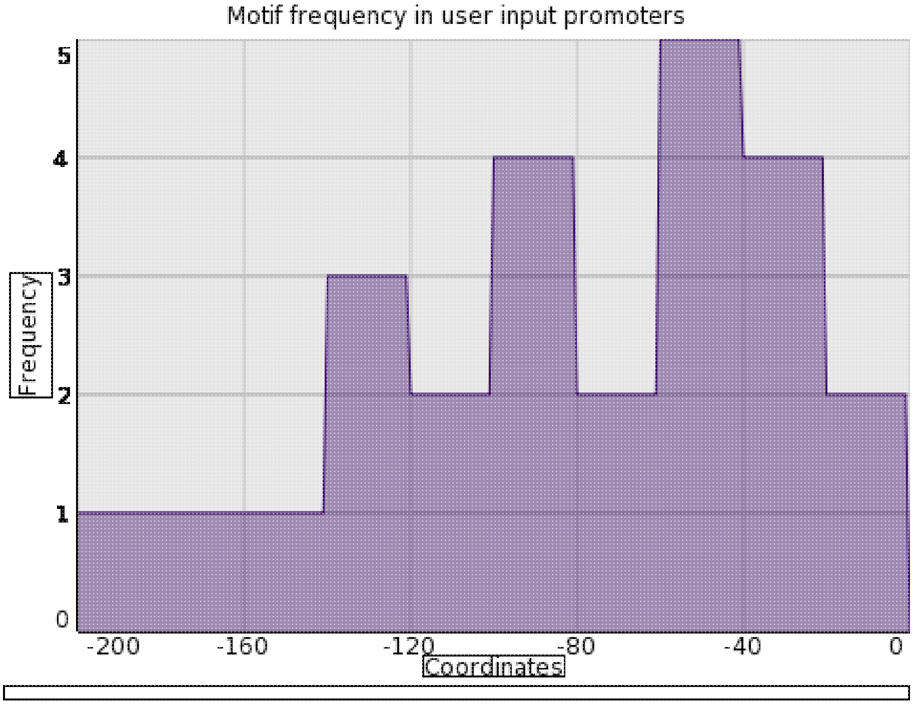

Motif12 annotation in the genome

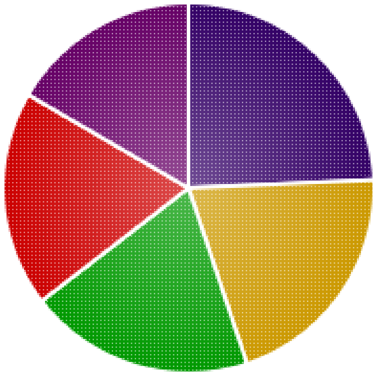

Annotation	p-value
GO:0008270	7.86e-04
GO:0016114	2.33e-03
GO:0033897	3.08e-03
GO:0006879	3.91e-03
GO:0006418	7.57e-03

Annotation complete description

GO:0008270 => zinc ion binding GO:0016114 => terpenoid biosynthetic process GO:0033897 => ribonuclease T2 activity GO:0006879 => cellular iron ion homeostasis GO:0006418 => tRNA aminoacylation for protein translation

Genome-wide Motif12 search results

Motif12 gene list of over-represented annotation(s)

Motif13

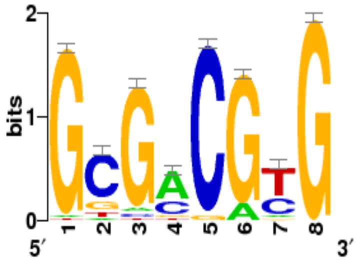

GCGACGTG

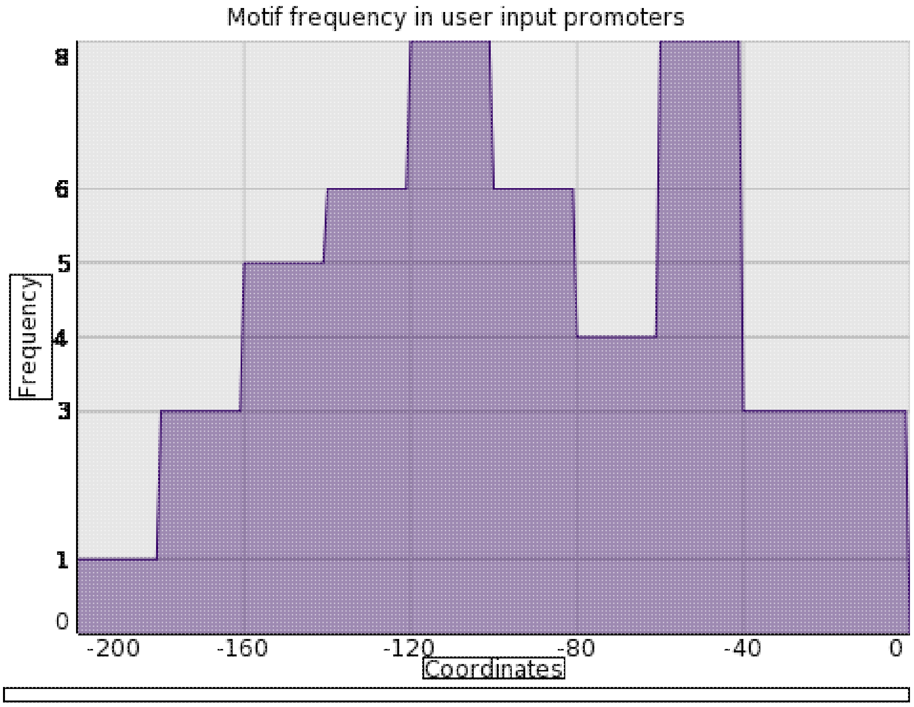

Motif13 annotation in the genome

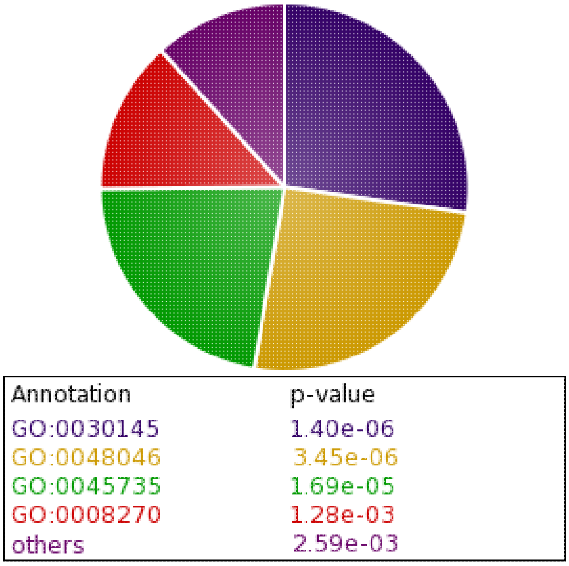

Annotation complete description

GO:0030145 => manganese ion binding GO:0048046 => apoplast GO:0045735 => nutrient reservoir activity GO:0008270 => zinc ion binding GO:0003677 => DNA binding GO:0003676 => nucleic acid binding GO:0004707 => MAP kinase activity

Genome-wide Motif13 search results

Motif13 gene list of over-represented annotation(s)

results - 00000443

Sequence logo generated by weblogo
Graphic generated with Chart::Clicker,Perl module
Promzea program from the Raizada lab
