# Supplementary material for: Promzea: a pipeline for discovery of co-regulatory motifs in maize and other plant species and its application to the anthocyanin and phlobaphene biosynthetic pathways and the Maize Development Atlas
Source: BMC Plant Biol. 2013 Mar 15;13:42. doi: 10.1186/1471-2229-13-42 (PMC3658923; doi:10.1186/1471-2229-13-42)
Supplement: Additional file 7 — Supplemental files for testing Promzea with data sets from the Maize Development Atlas. The zip folder contains 3 folders. The first contains the promoter input for Promzea for each maize tissue; the second folder has all the outputs from Promzea; the third folder contains the STAMP website outputs for comparisons of the predicted motifs with experimentally defined motifs. [file 1471-2229-13-42-S7.zip › Supplemental files 3 -Case study 3/2-Promzea results/endosperm.pdf]

Results Summary

/vbox_shared/1-case_study_3/casestudy3_endosperm.txt

Promzea - 00000446

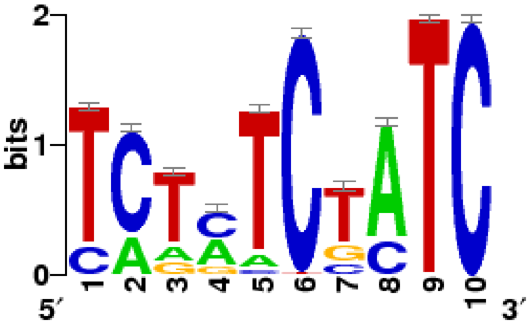

Motif1
(from BioP)
score: 1.45

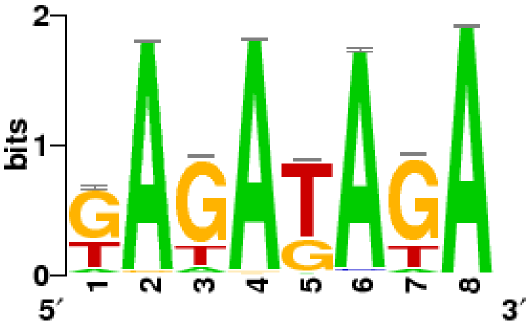

Motif2
(from Weeder)
score: 1.43

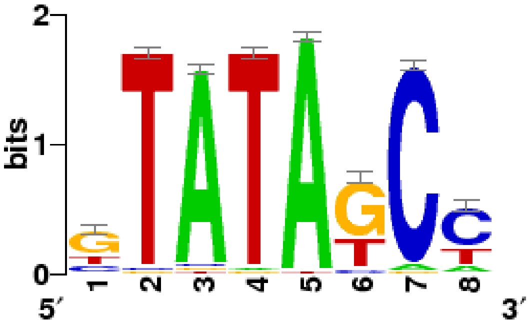

Motif3
(from Weeder)
score: 1.42

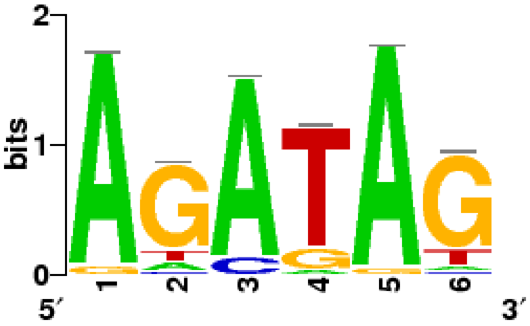

Motif4
(from Weeder)
score: 1.4

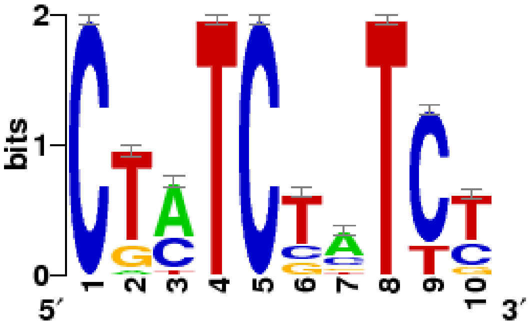

Motif5
(from BioP)
score: 1.38

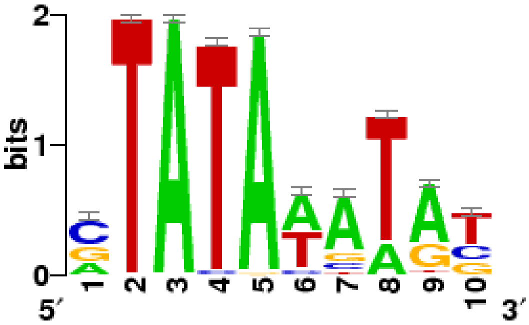

Motif6
(from MEME)
score: 1.38

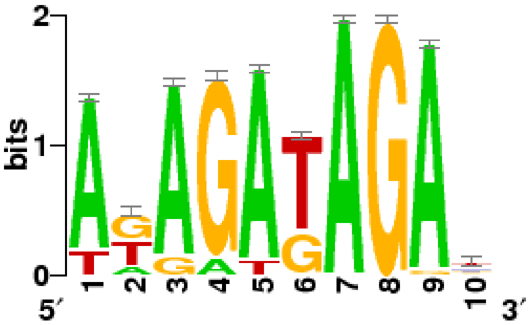

Motif7
(from BioP)
score: 1.37

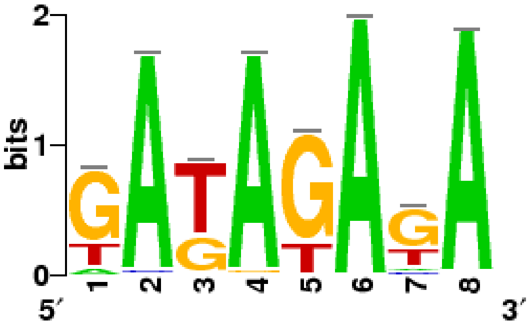

Motif8
(from Weeder)
score: 1.36

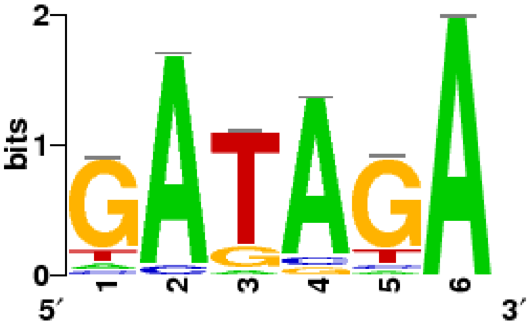

Motif9
(from Weeder)
score: 1.34

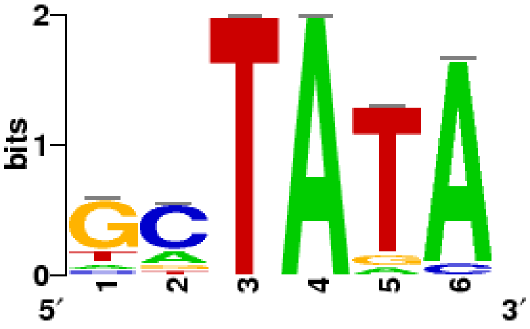

Motif10
(from Weeder)
score: 1.34

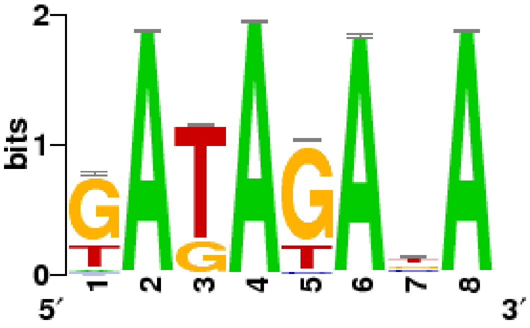

Motif11
(from Weeder)
score: 1.32

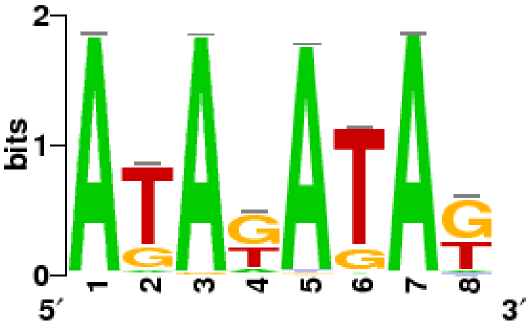

Motif12
(from Weeder)
score: 1.07

Compare your motifs to known promoter motif databases using STAMP website [motif file to copy in STAMP website]

Open the above link, copy content of the newly open file and paste in STAMP program link below In STAMP, under "Similarity Matching", we suggest selecting the plant motif databases: Athamap, AGRIS, PLACE, TRANSFAC; then submit

[STAMP website]

Motif1

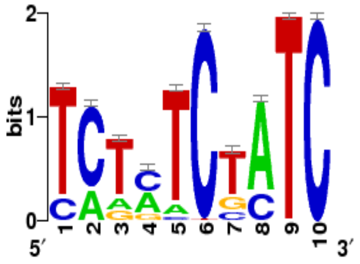

TCTCTCTATC

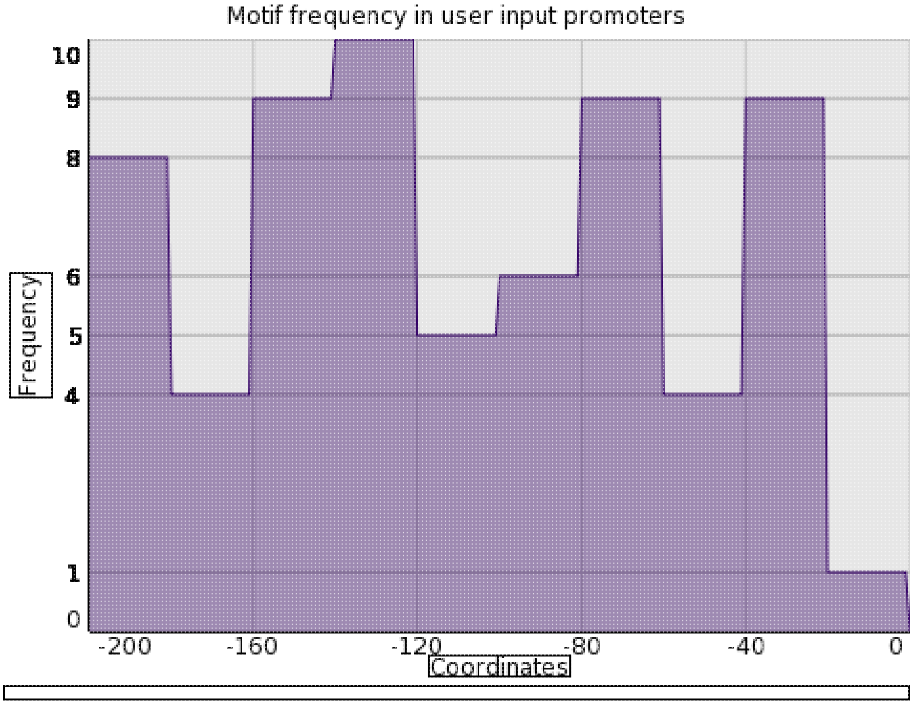

Motif1 annotation in the genome

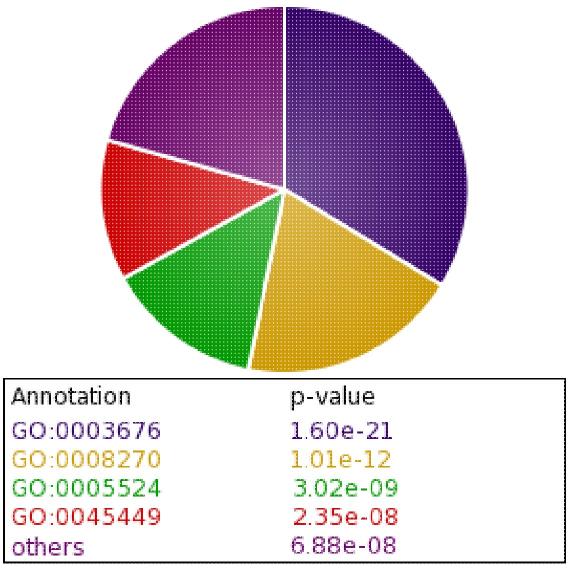

Annotation complete description

GO:0003676 => nucleic acid binding GO:0008270 => zinc ion binding GO:0005524 => ATP binding GO:0045449 => regulation of transcription GO:0046983 => protein dimerization activity GO:0006886 => intracellular protein transport GO:0005515 => protein binding

Genome-wide Motif1 search results

Motif1 gene list of over-represented annotation(s)

Motif2

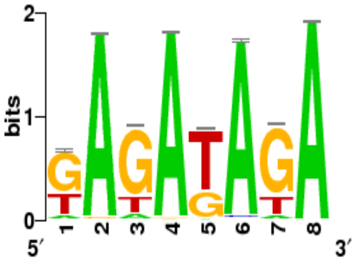

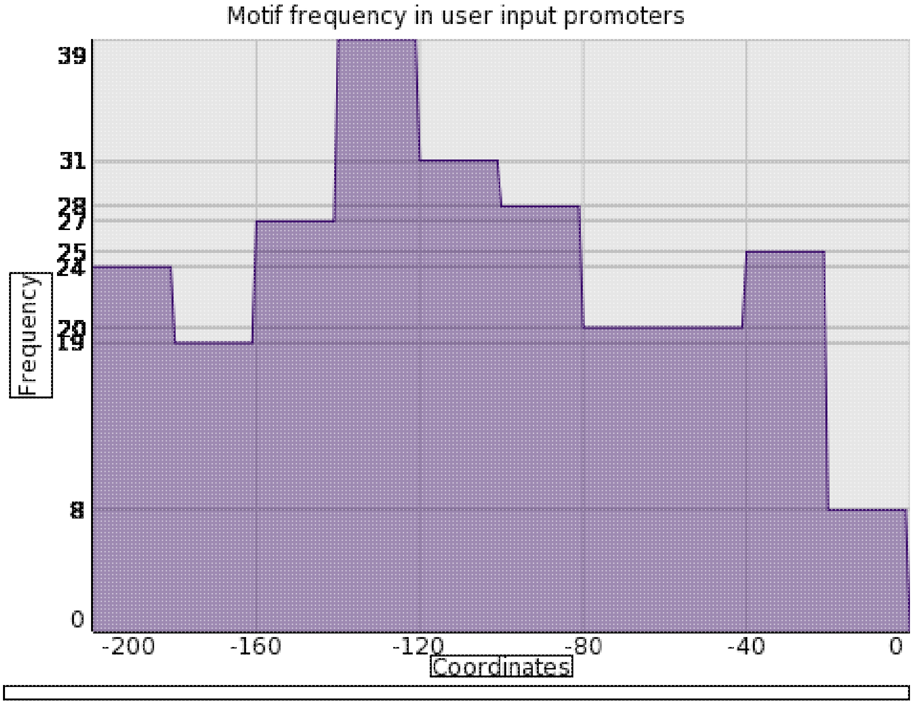

GAGATAGA

Motif2 annotation in the genome

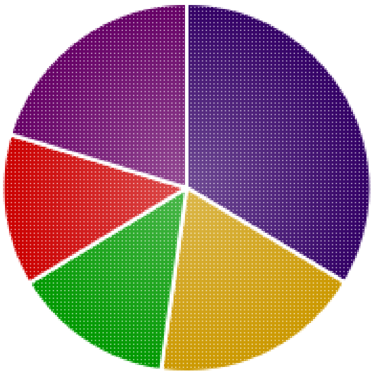

Annotation	p-value
GO:0003676	8.56e-22
GO:0008270	1.98e-12
GO:0005524	1.39e-09
GO:0045449	4.41e-09
others	7.53e-08

Annotation complete description

GO:0003676 => nucleic acid binding GO:0008270 => zinc ion binding GO:0005524 => ATP binding GO:0045449 => regulation of transcription GO:0046983 => protein dimerization activity GO:0006886 => intracellular protein transport GO:0006508 => proteolysis

Genome-wide Motif2 search results

Motif2 gene list of over-represented annotation(s)

Motif3

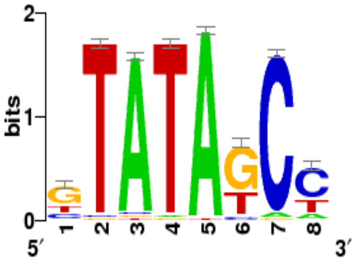

GTATAGCC

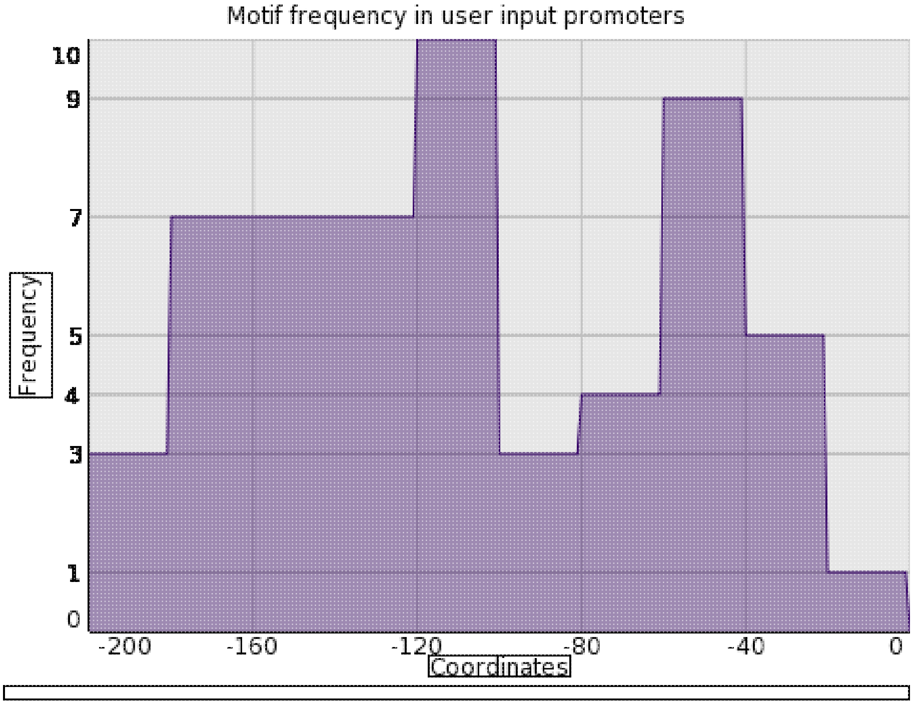

Motif3 annotation in the genome

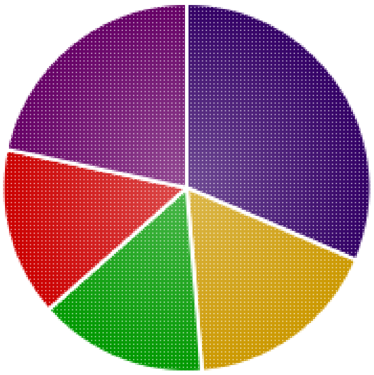

Annotation	p-value
GO:0003676	1.53e-09
GO:0008270	1.43e-05
GO:0045449	6.87e-05
GO:0016068	6.90e-05
others	1.22e-04

Annotation complete description

GO:0003676 => nucleic acid binding GO:0008270 => zinc ion binding GO:0045449 => regulation of transcription GO:0016068 => type I hypersensitivity GO:0005524 => ATP binding GO:0051082 => unfolded protein binding GO:0005488 => binding

Genome-wide Motif3 search results

Motif3 gene list of over-represented annotation(s)

Motif4

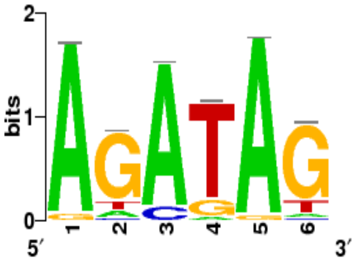

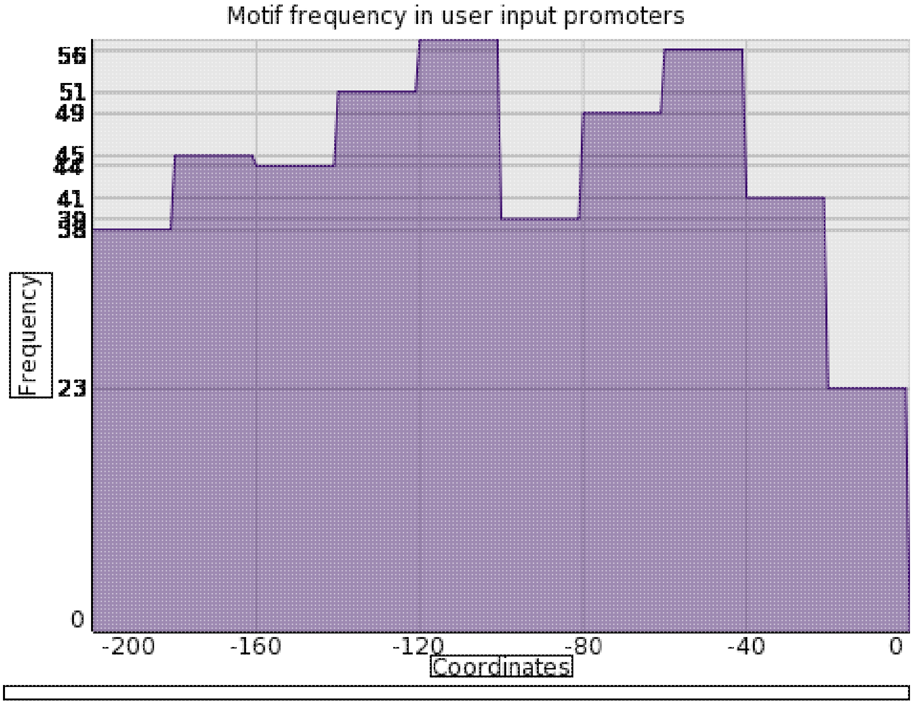

AGATAG

Motif4 annotation in the genome

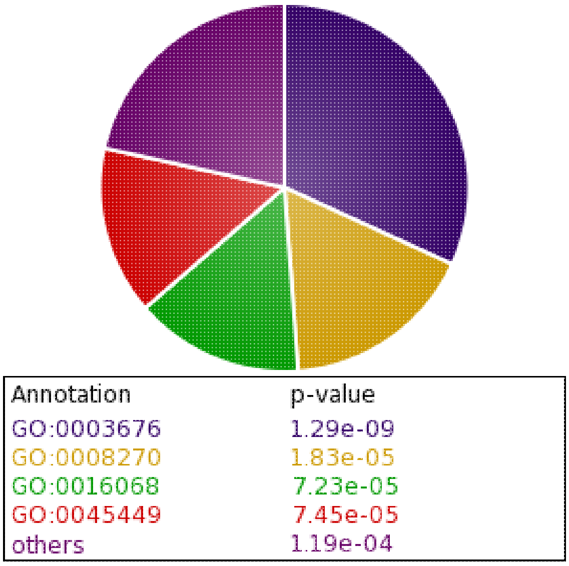

Annotation complete description

GO:0003676 => nucleic acid binding GO:0008270 => zinc ion binding GO:0016068 => type I hypersensitivity GO:0045449 => regulation of transcription GO:0051082 => unfolded protein binding GO:0005524 => ATP binding GO:0005488 => binding

Genome-wide Motif4 search results

Motif4 gene list of over-represented annotation(s)

Motif5

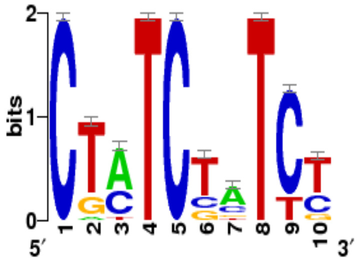

CTATCTATCT

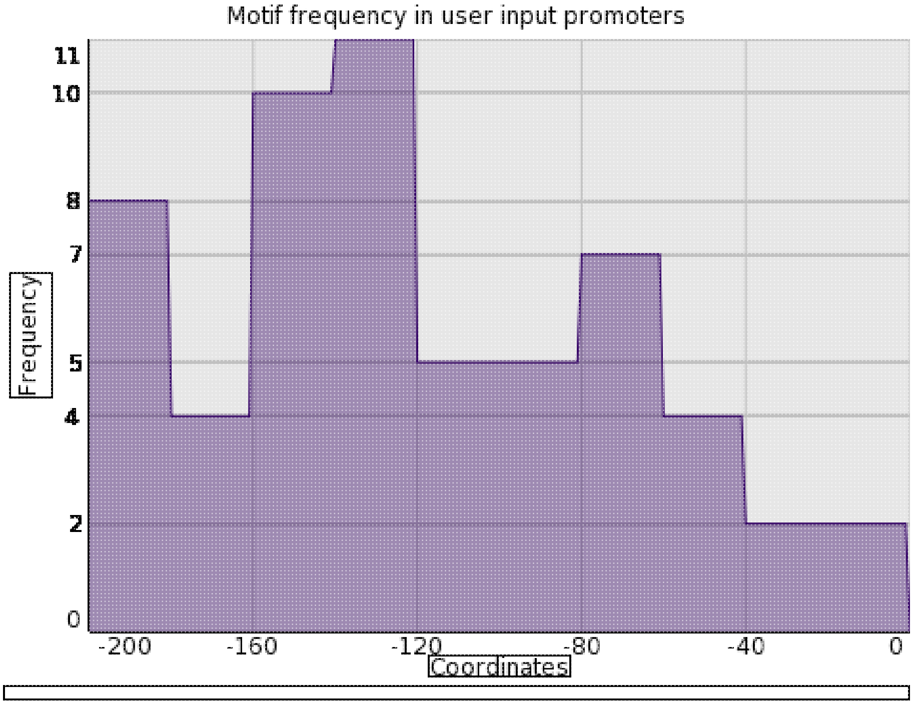

Motif5 annotation in the genome

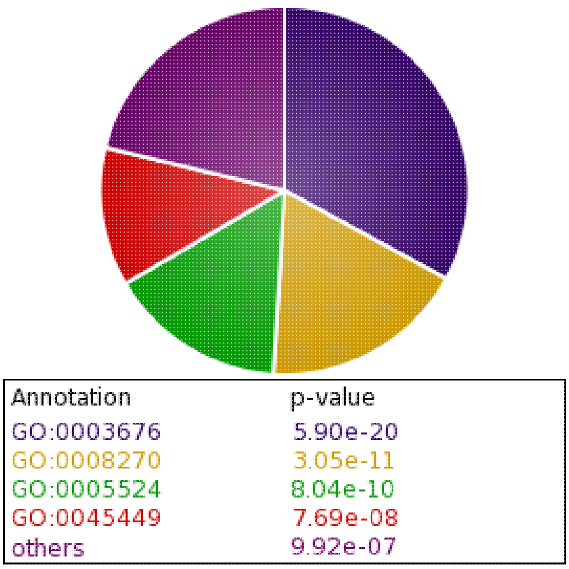

Annotation complete description

GO:0003676 => nucleic acid binding GO:0008270 => zinc ion binding GO:0005524 => ATP binding GO:0045449 => regulation of transcription GO:0046983 => protein dimerization activity GO:0003723 => RNA binding GO:0006508 => proteolysis

Genome-wide Motif5 search results

Motif5 gene list of over-represented annotation(s)

Motif6

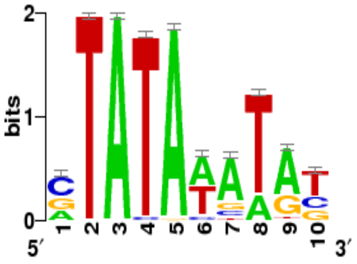

CTATAAATAT

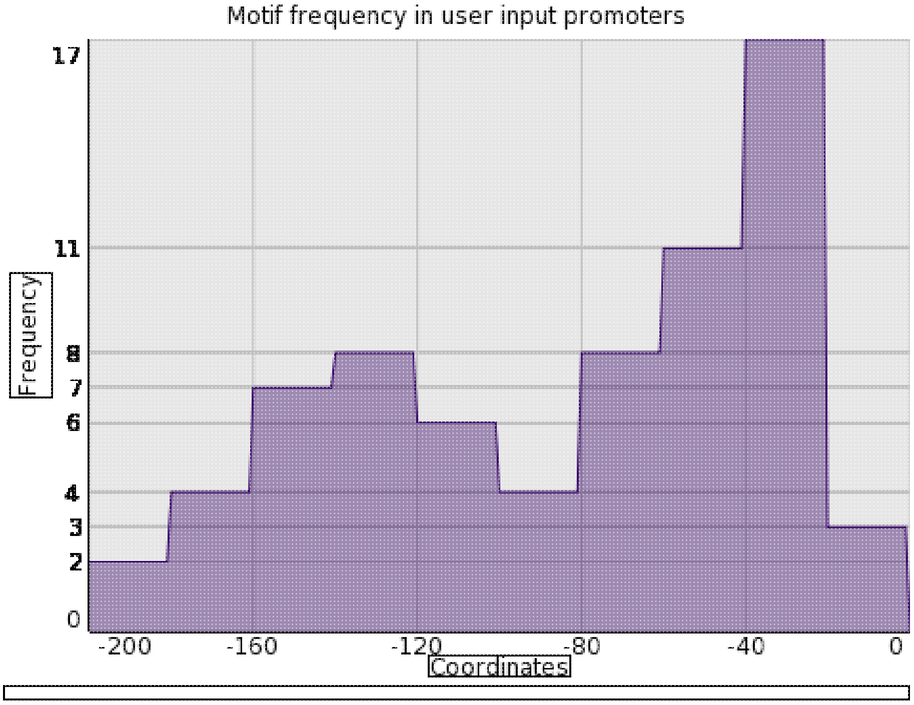

Motif6 annotation in the genome

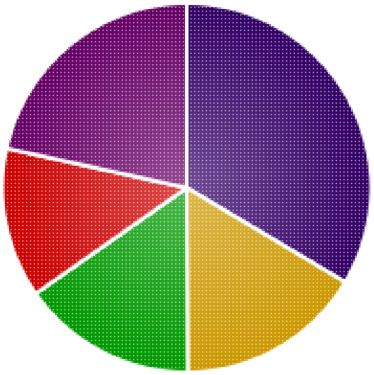

Annotation	p-value
GO:0003676	1.44e-19
GO:0008270	6.40e-10
GO:0005524	2.36e-09
GO:0045449	4.15e-08
others	1.69e-06

Annotation complete description

GO:0003676 => nucleic acid binding GO:0008270 => zinc ion binding GO:0005524 => ATP binding GO:0045449 => regulation of transcription GO:0003723 => RNA binding GO:0005488 => binding GO:0046983 => protein dimerization activity

Genome-wide Motif6 search results

Motif6 gene list of over-represented annotation(s)

Motif7

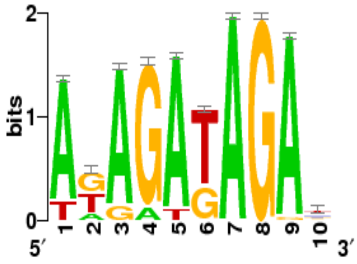

AGAGATAGAT

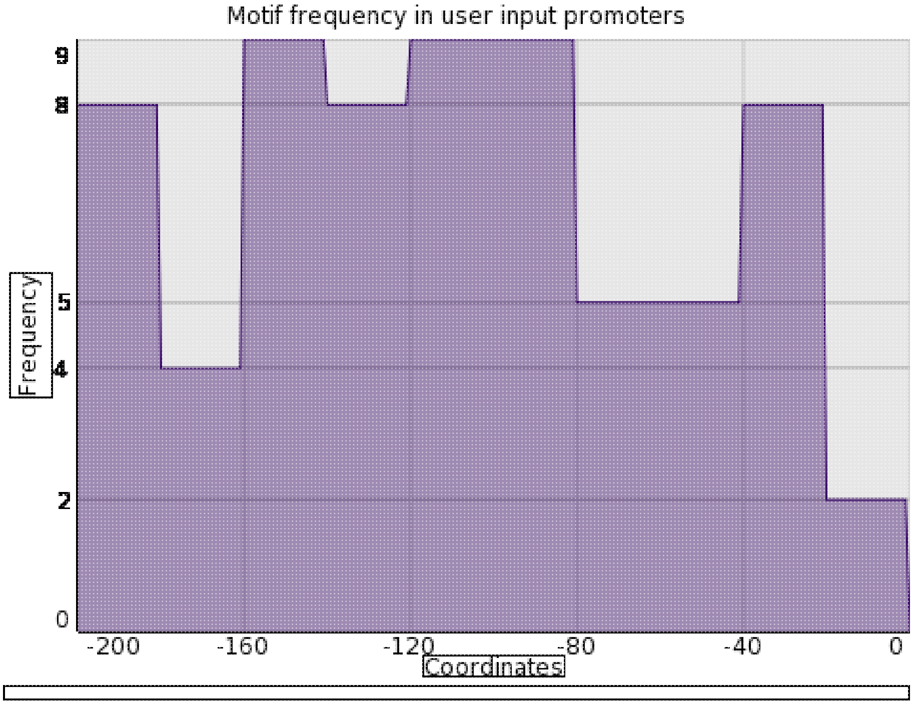

Motif7 annotation in the genome

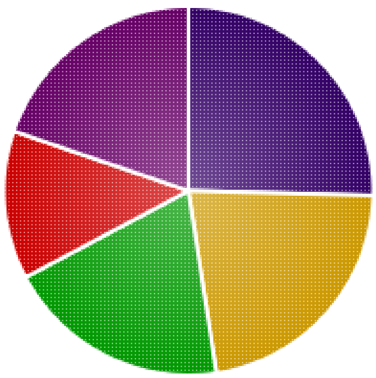

Annotation	p-value
GO:0003676	3.49e-07
GO:0016068	2.31e-06
GO:0045449	1.02e-05
GO:0003999	4.95e-04
others	4.95e-04

Annotation complete description

GO:0003676 => nucleic acid binding GO:0016068 => type I hypersensitivity GO:0045449 => regulation of transcription GO:0003999 => adenine phosphoribosyltransferase activity GO:0006168 => adenine salvage GO:0008270 => zinc ion binding GO:0046983 => protein dimerization activity

Genome-wide Motif7 search results

Motif7 gene list of over-represented annotation(s)

Motif8

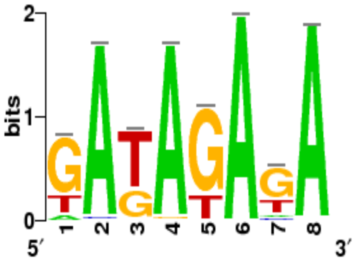

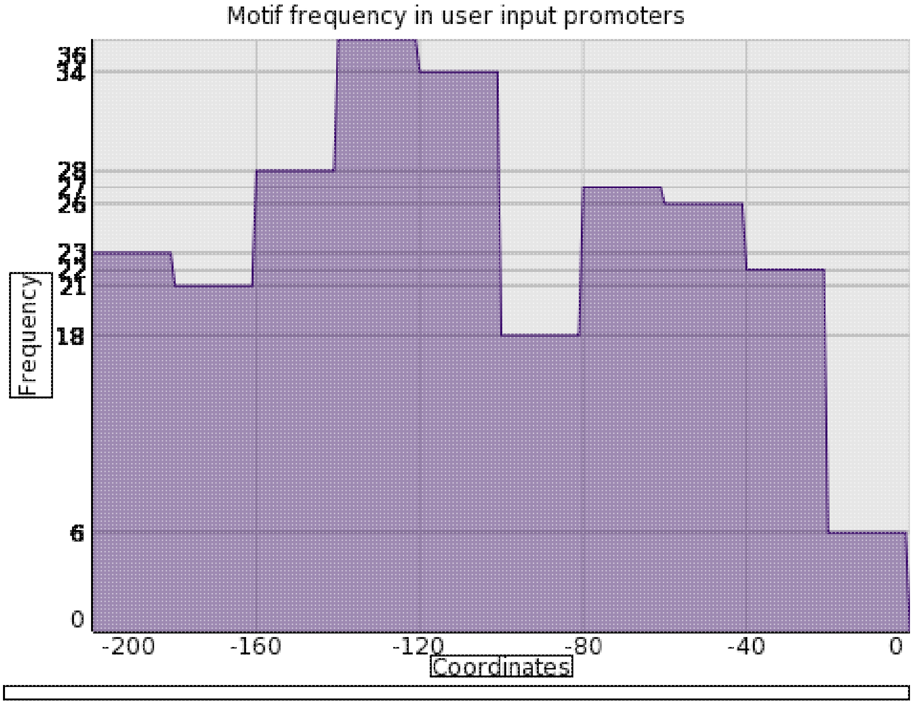

GATAGAGA

Motif8 annotation in the genome

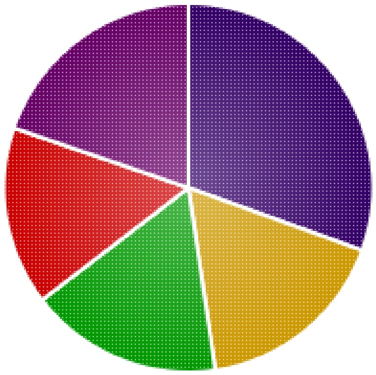

Annotation	p-value
GO:0003676	2.08e-08
GO:0016068	4.77e-05
GO:0008270	5.50e-05
GO:0045449	1.14e-04
others	1.28e-04

Annotation complete description

GO:0003676 => nucleic acid binding GO:0016068 => type I hypersensitivity GO:0008270 => zinc ion binding GO:0045449 => regulation of transcription GO:0046983 => protein dimerization activity GO:0051082 => unfolded protein binding GO:0005488 => binding

Genome-wide Motif8 search results

Motif8 gene list of over-represented annotation(s)

Motif9

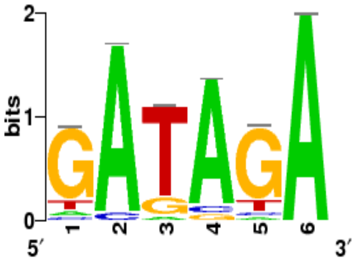

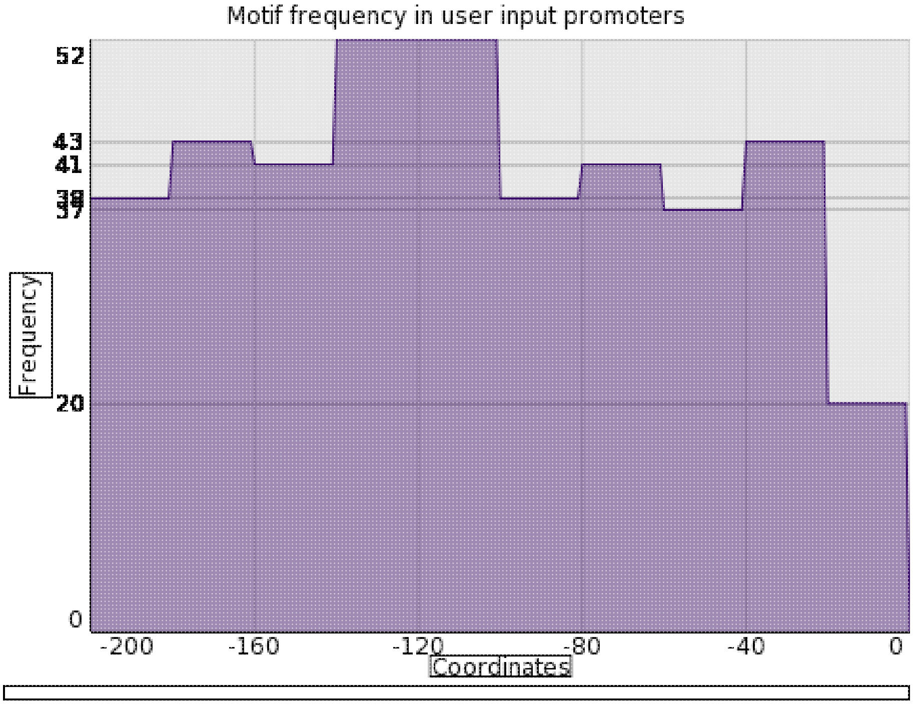

GATAGA

Motif9 annotation in the genome

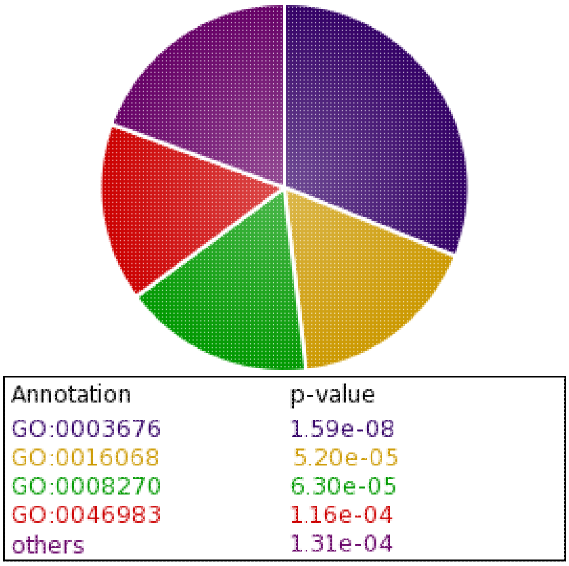

Annotation complete description

GO:0003676 => nucleic acid binding GO:0016068 => type I hypersensitivity GO:0008270 => zinc ion binding GO:0046983 => protein dimerization activity GO:0045449 => regulation of transcription GO:0051082 => unfolded protein binding GO:0005524 => ATP binding

Genome-wide Motif9 search results

Motif9 gene list of over-represented annotation(s)

Motif10

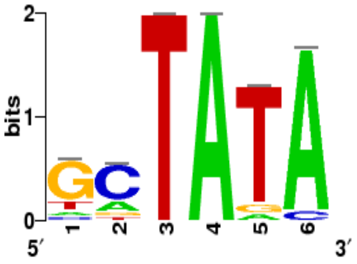

GCTATA

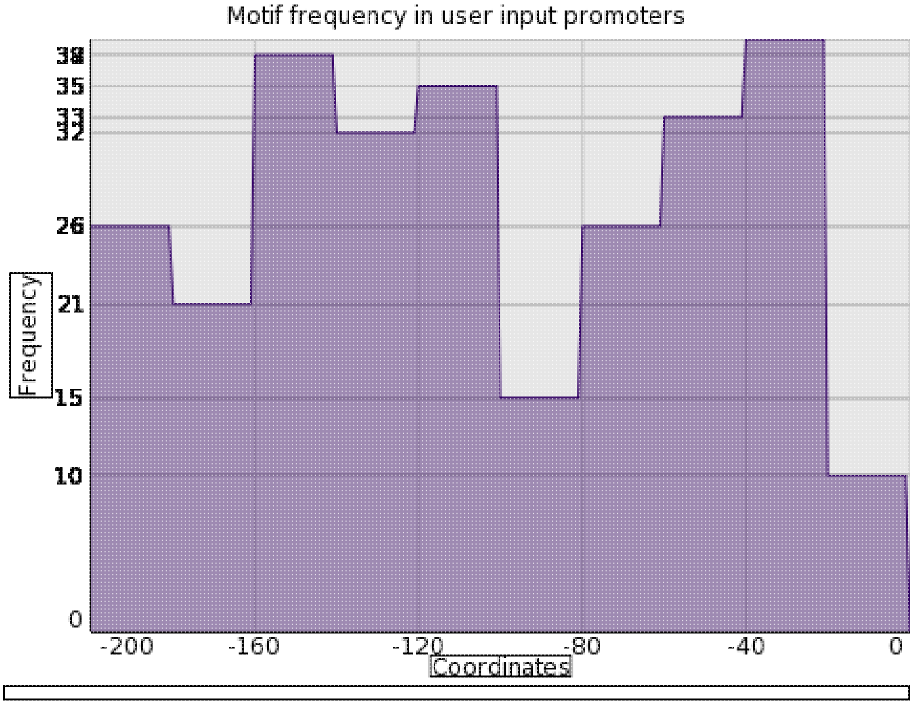

Motif10 annotation in the genome

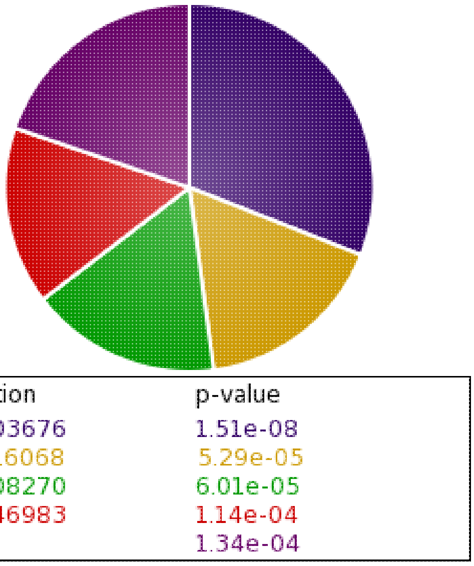

Annotation complete description

GO:0003676 => nucleic acid binding GO:0016068 => type I hypersensitivity GO:0008270 => zinc ion binding GO:0046983 => protein dimerization activity GO:0045449 => regulation of transcription GO:0051082 => unfolded protein binding GO:0005524 => ATP binding

Genome-wide Motif10 search results

Motif10 gene list of over-represented annotation(s)

Motif11

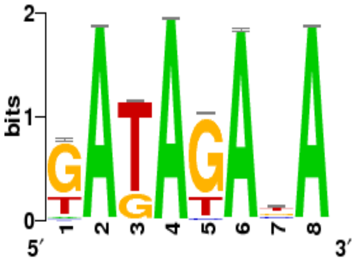

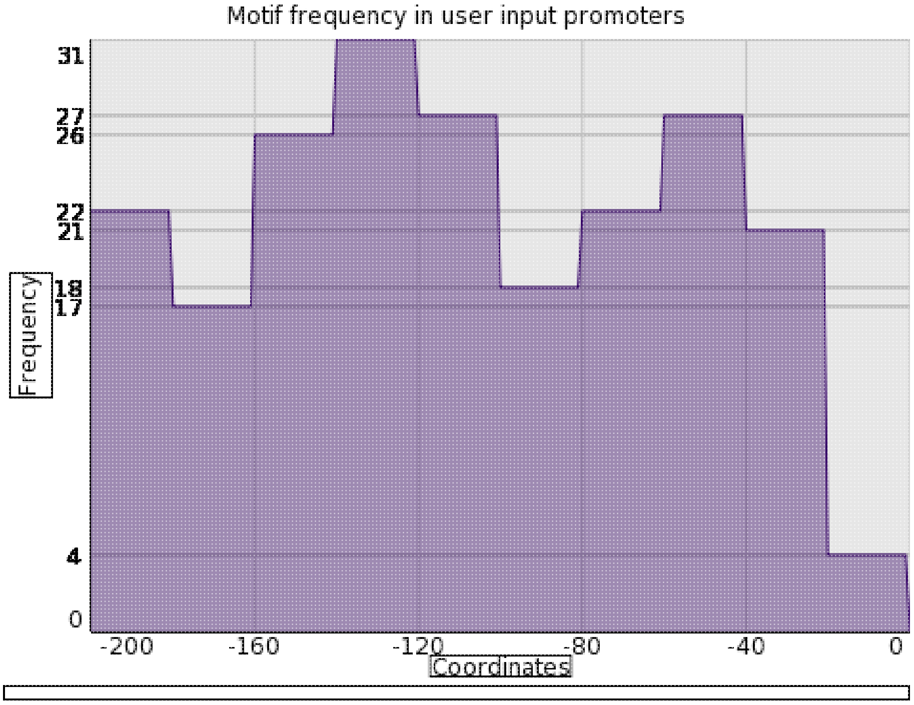

GATAGATA

Motif11 annotation in the genome

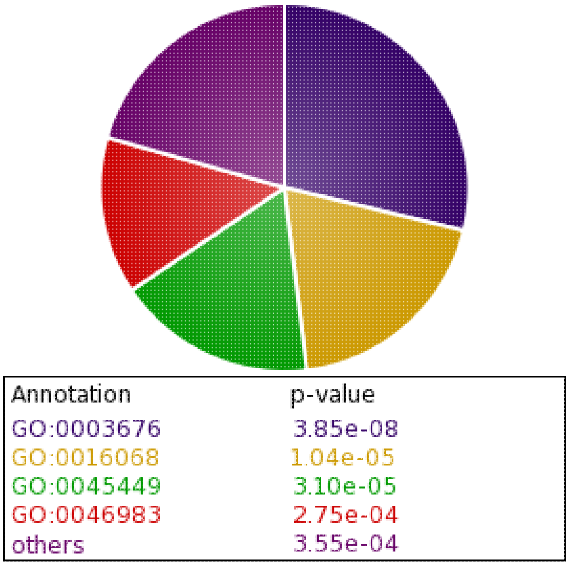

Annotation complete description

GO:0003676 => nucleic acid binding GO:0016068 => type I hypersensitivity GO:0045449 => regulation of transcription GO:0046983 => protein dimerization activity GO:0008270 => zinc ion binding GO:0003999 => adenine phosphoribosyltransferase activity GO:0006168 => adenine salvage

Genome-wide Motif11 search results

Motif11 gene list of over-represented annotation(s)

Motif12

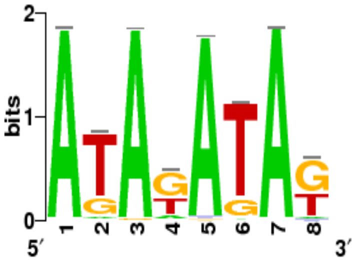

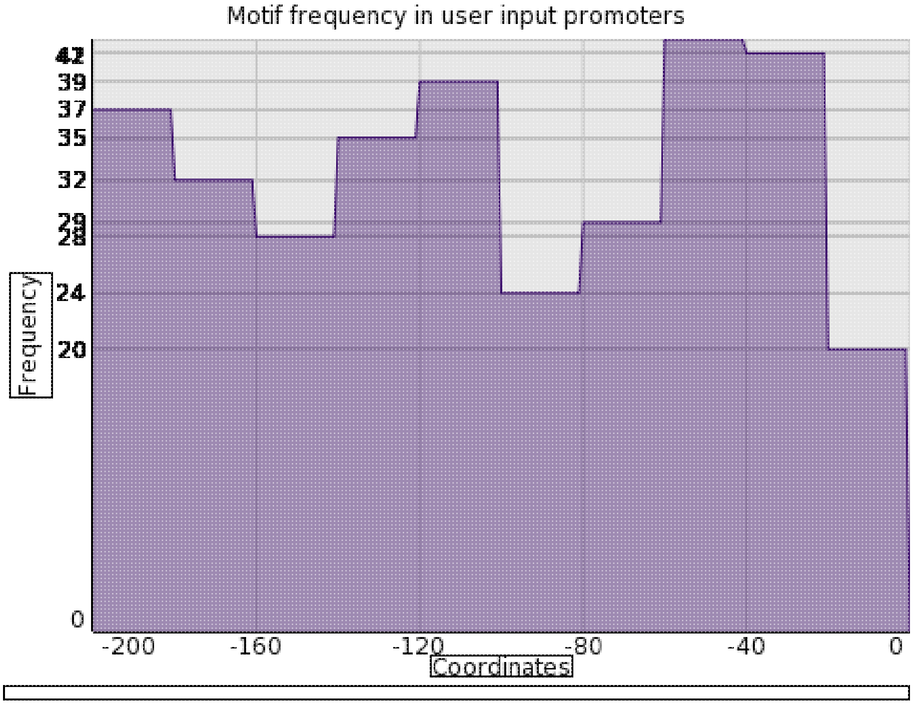

ATAGATAG

Motif12 annotation in the genome

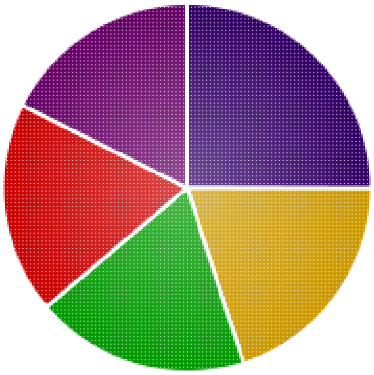

Annotation	p-value
GO:0016702	1.20e-04
GO:0004097	7.37e-04
GO:0005524	1.16e-03
GO:0045449	1.23e-03
others	1.27e-03

Annotation complete description

GO:0016702 => oxidoreductase activity, acting on single donors with incorporation of molecular oxygen, incorporation of two atoms of oxygen GO:0004097 => catechol oxidase activity GO:0005524 => ATP binding GO:0045449 => regulation of transcription GO:0003676 => nucleic acid binding GO:0016165 => lipoxygenase activity GO:0016068 => type I hypersensitivity

Genome-wide Motif12 search results

Motif12 gene list of over-represented annotation(s)

Sequence logo generated by weblogo
Graphic generated with Chart::Clicker,Perl module
Promzea program from the Raizada lab
