# Supplementary material for: Promzea: a pipeline for discovery of co-regulatory motifs in maize and other plant species and its application to the anthocyanin and phlobaphene biosynthetic pathways and the Maize Development Atlas
Source: BMC Plant Biol. 2013 Mar 15;13:42. doi: 10.1186/1471-2229-13-42 (PMC3658923; doi:10.1186/1471-2229-13-42)
Supplement: Additional file 7 — Supplemental files for testing Promzea with data sets from the Maize Development Atlas. The zip folder contains 3 folders. The first contains the promoter input for Promzea for each maize tissue; the second folder has all the outputs from Promzea; the third folder contains the STAMP website outputs for comparisons of the predicted motifs with experimentally defined motifs. [file 1471-2229-13-42-S7.zip › Supplemental files 3 -Case study 3/2-Promzea results/internode.pdf]

Home

# Results Summary

# /vbox_shared/1-case_study_3/casestudy3_internode.txt

Promzea - 00000447

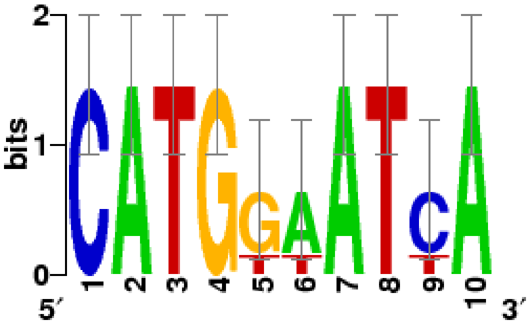

**Motif1**
(from MEME)
**score: 36.89**

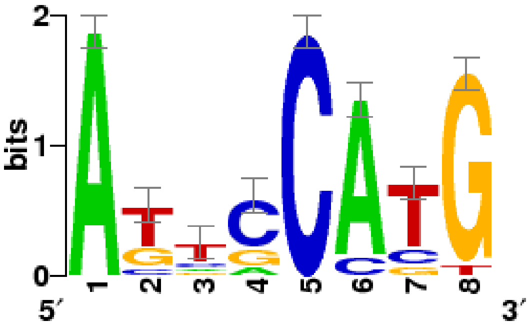

**Motif2**
(from Weeder)
**score: 35.74**

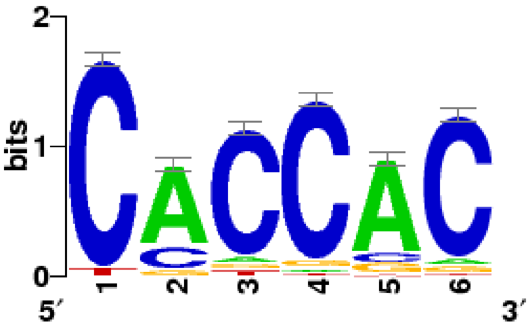

**Motif3**
(from Weeder)
**score: 34.76**

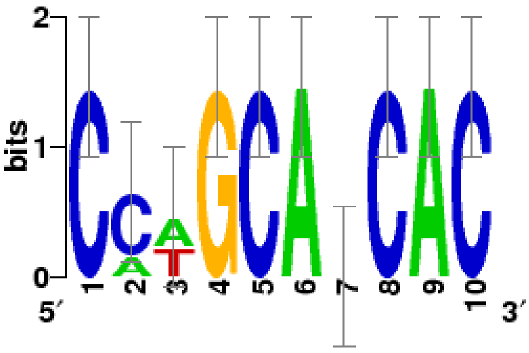

**Motif4**
(from MEME)
**score: 25.01**

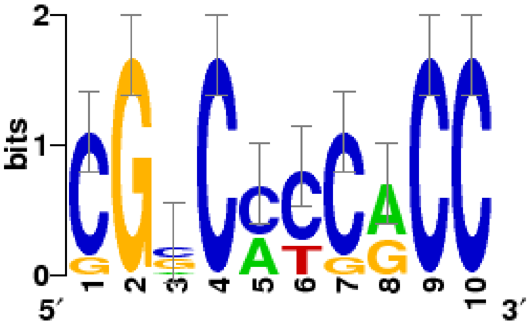


**Motif5**
(from MEME)
**score: 22.78**

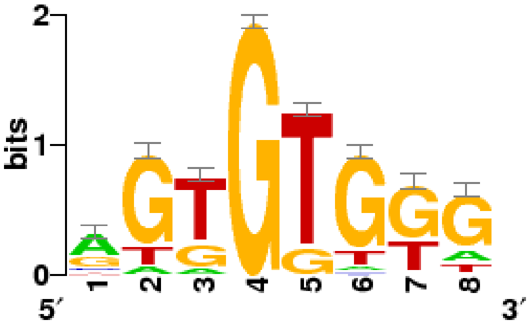


**Motif6**
(from Weeder)
**score: 13.96**

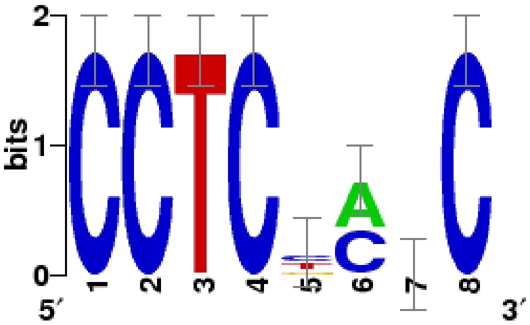


**Motif7**
(from MEME)
**score: 13.06**

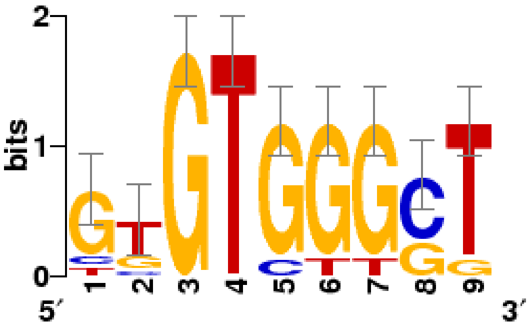


**Motif8**
(from MEME)
**score: 11.61**

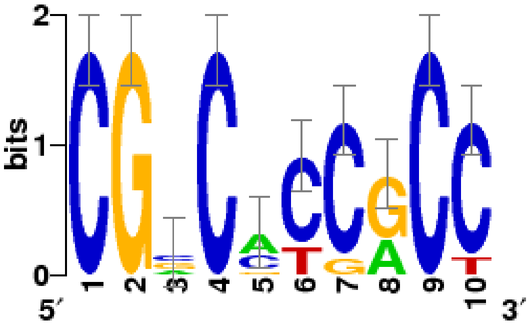


**Motif9**
(from BioP)
**score: 9.56**

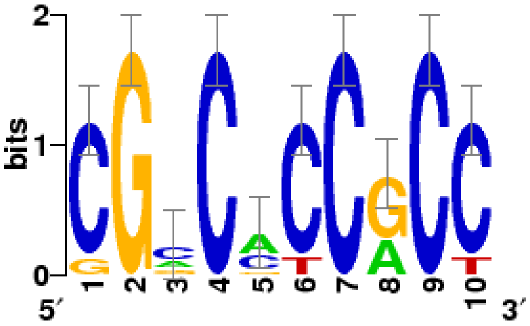

**Motif10**
(from BioP)
**score: 3.67**

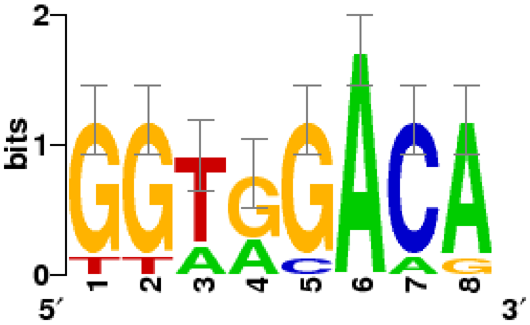

**Motif11**
(from MEME)
**score: 3.24**

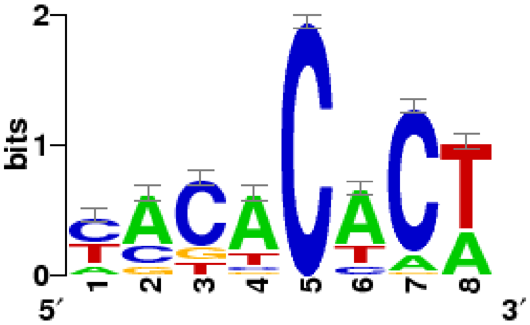

**Motif12**
(from Weeder)
**score: 3.1**

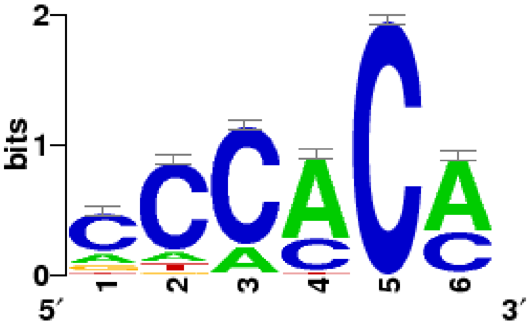

**Motif13**
(from Weeder)
**score: 3.04**

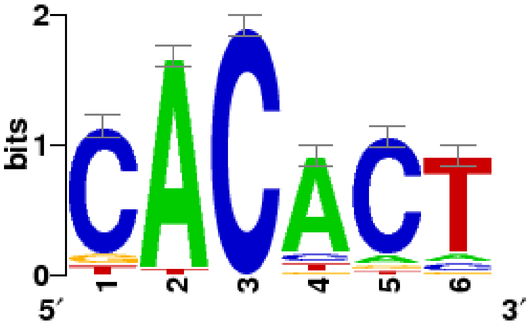

**Motif14**
(from Weeder)
**score: 3.02**

Compare your motifs to known promoter motif databases using STAMP website motif file to copy in STAMP website

Open the above link, copy content of the newly open file and paste in STAMP program link below In STAMP, under "Similarity Matching", we suggest selecting the plant motif databases: Athamap, AGRIS, PLACE, TRANSFAC; then submit

STAMP website

---

---

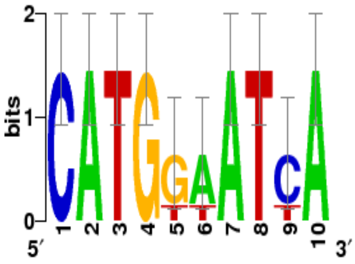


**CATGGAATCA**

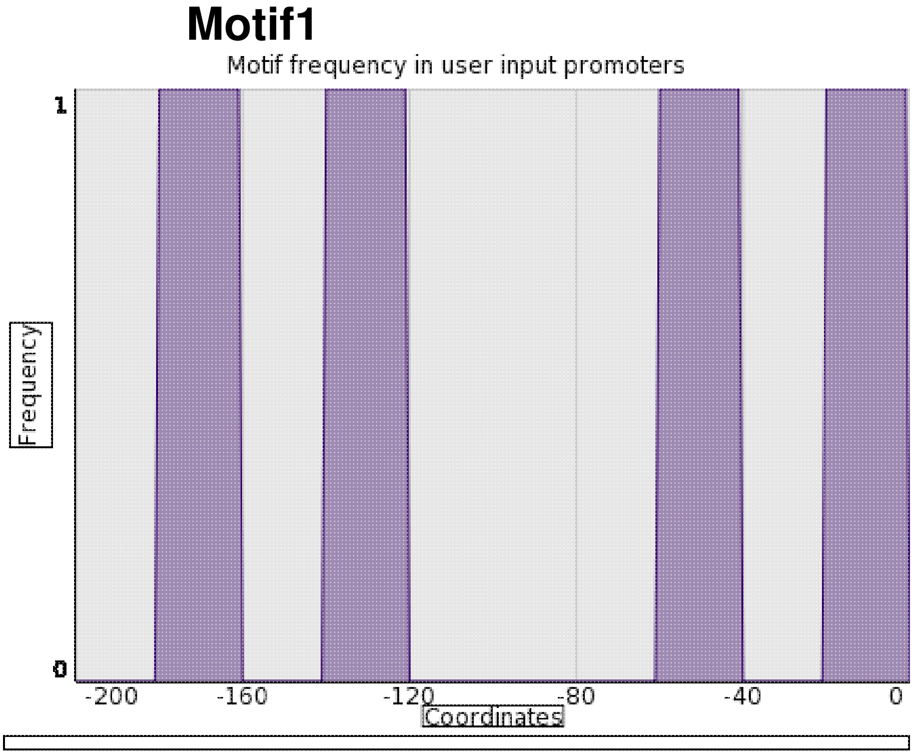


---

Motif1 annotation in the genome

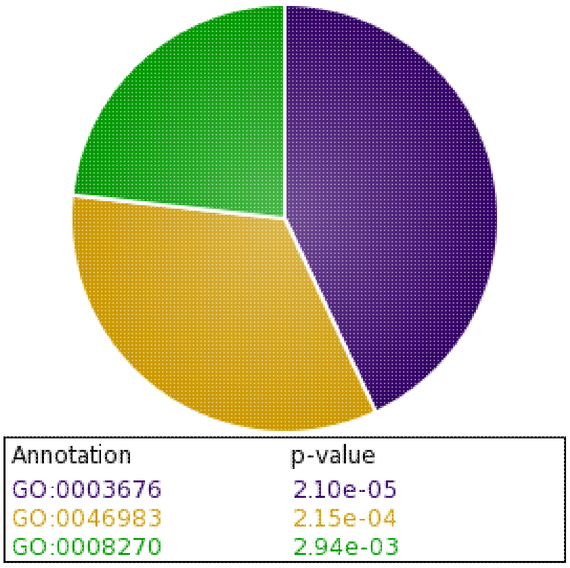


**Annotation complete description**

GO:0003676 => nucleic acid binding GO:0046983 => protein dimerization activity GO:0008270 => zinc ion binding

Genome-wide Motif1 search results

Motif1 gene list of over-represented annotation(s)

---

---

# Motif2

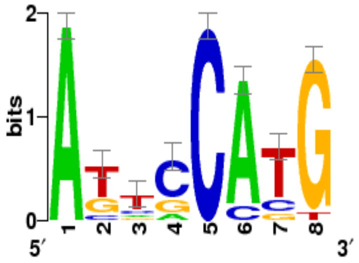


**ATTCCATG**

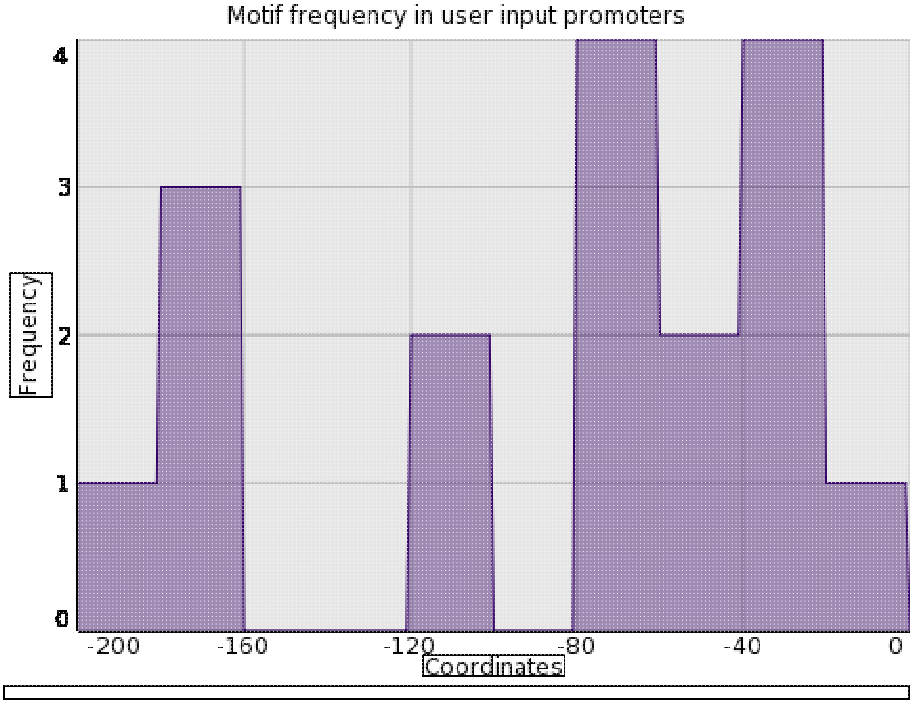


---

Motif2 annotation in the genome

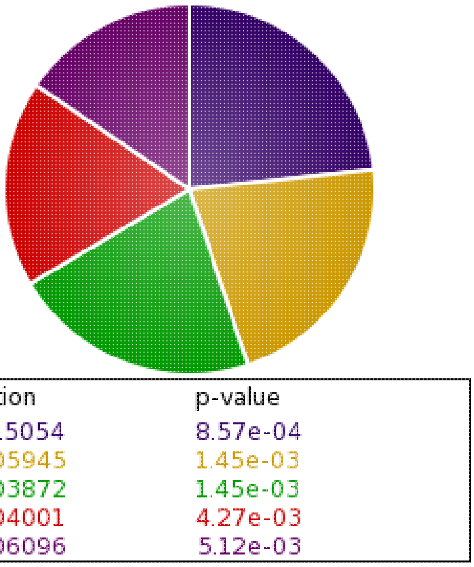


**Annotation complete description**

GO:0015054 => gastrin receptor activity GO:0005945 => 6-phosphofructokinase complex GO:0003872 => 6-phosphofructokinase activity GO:0004001 => adenosine kinase activity GO:0006166 => purine ribonucleoside salvage GO:0006096 => glycolysis

Genome-wide Motif2 search results

Motif2 gene list of over-represented annotation(s)

---

# Motif3

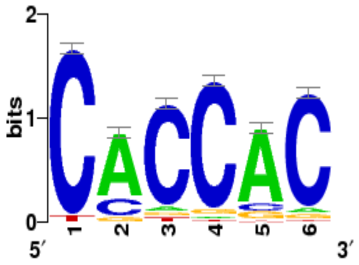


**CACCAC**

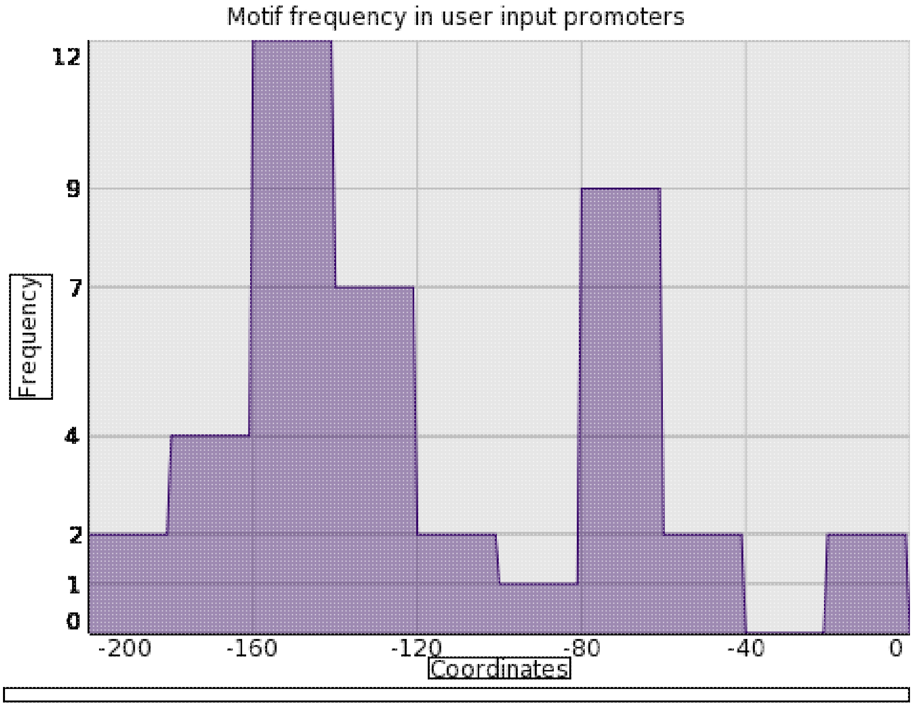


---

Motif3 annotation in the genome

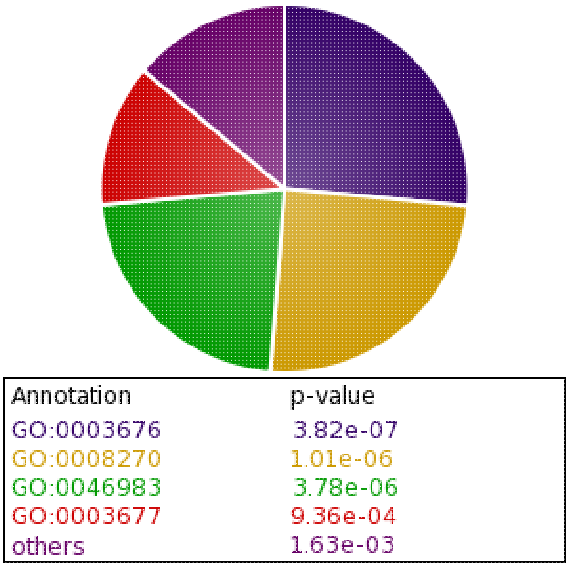


**Annotation complete description**

GO:0003676 => nucleic acid binding GO:0008270 => zinc ion binding GO:0046983 => protein dimerization activity GO:0003677 => DNA binding GO:0000151 => ubiquitin ligase complex GO:0016567 => protein ubiquitination GO:0004842 => ubiquitin-protein ligase activity

Genome-wide Motif3 search results

Motif3 gene list of over-represented annotation(s)

---

---

# Motif4

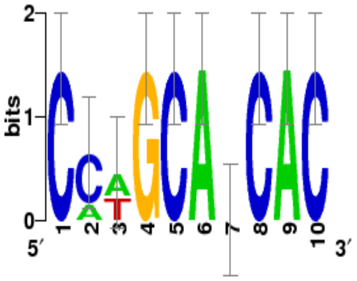


**CCWGCACCAC**

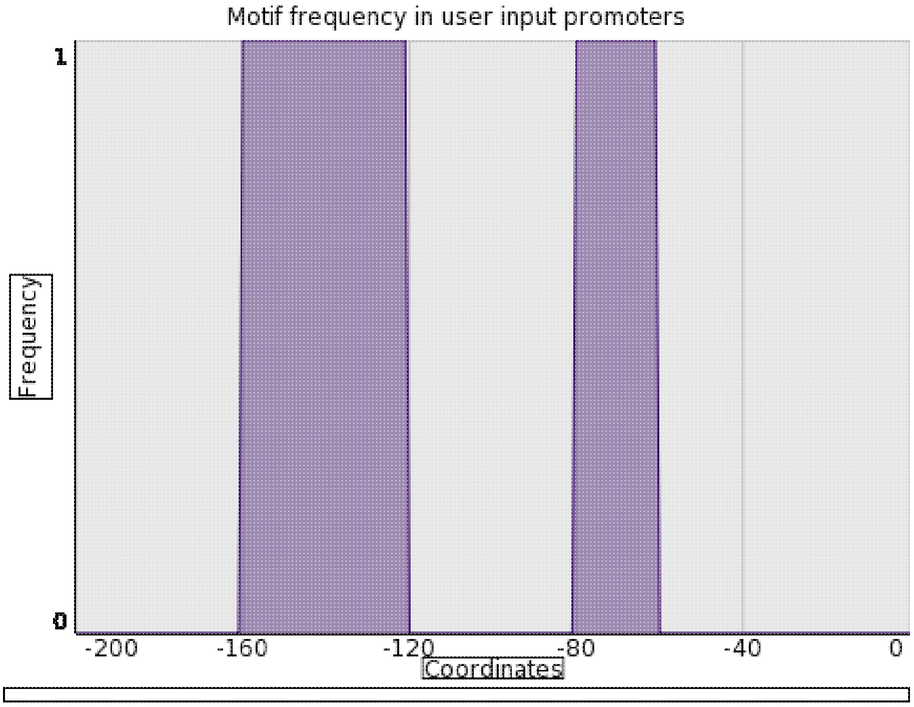


---

Motif4 annotation in the genome

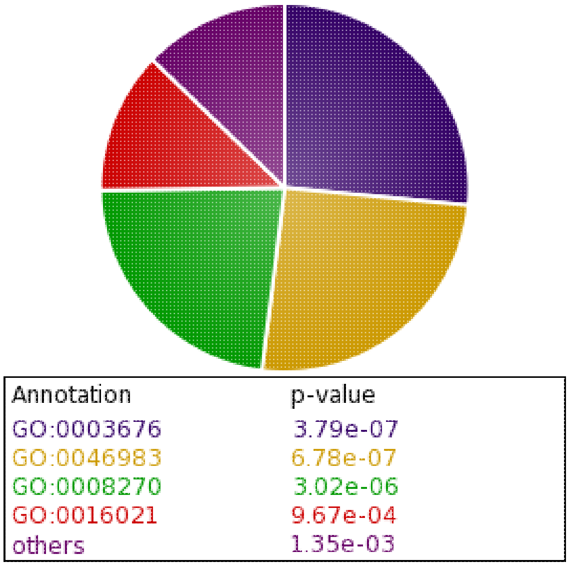


**Annotation complete description**

GO:0003676 => nucleic acid binding GO:0046983 => protein dimerization activity GO:0008270 => zinc ion binding GO:0016021 => integral to membrane GO:0003677 => DNA binding GO:0004379 => glycylpeptide N-tetradecanoyltransferase activity GO:0004722 => protein serine/threonine phosphatase activity

Genome-wide Motif4 search results

Motif4 gene list of over-represented annotation(s)

---

---

# Motif5

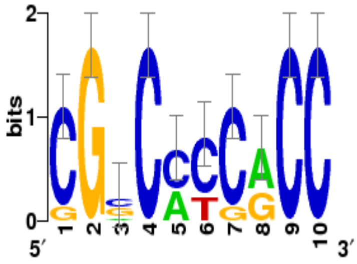


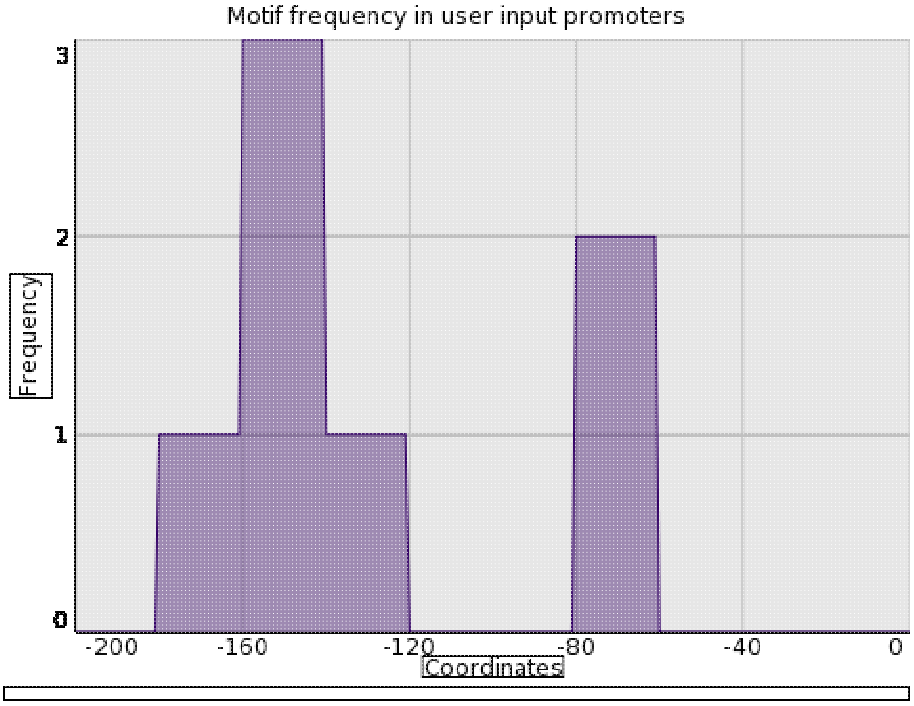


**CGSCCCCACC**

---

Motif5 annotation in the genome

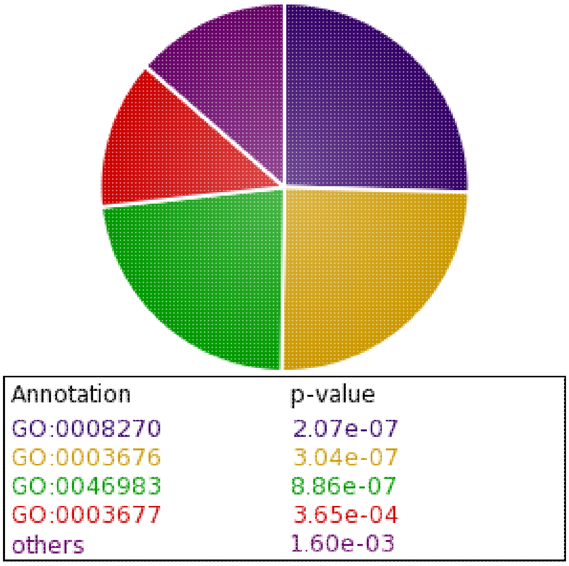


**Annotation complete description**

GO:0008270 => zinc ion binding GO:0003676 => nucleic acid binding GO:0046983 => protein dimerization activity GO:0003677 => DNA binding GO:0016021 => integral to membrane GO:0005524 => ATP binding GO:0004379 => glycylpeptide N-tetradecanoyltransferase activity

Genome-wide Motif5 search results

Motif5 gene list of over-represented annotation(s)

# Motif6

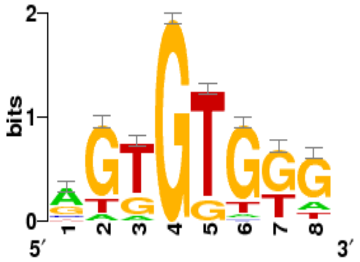


**AGTGTGGG**

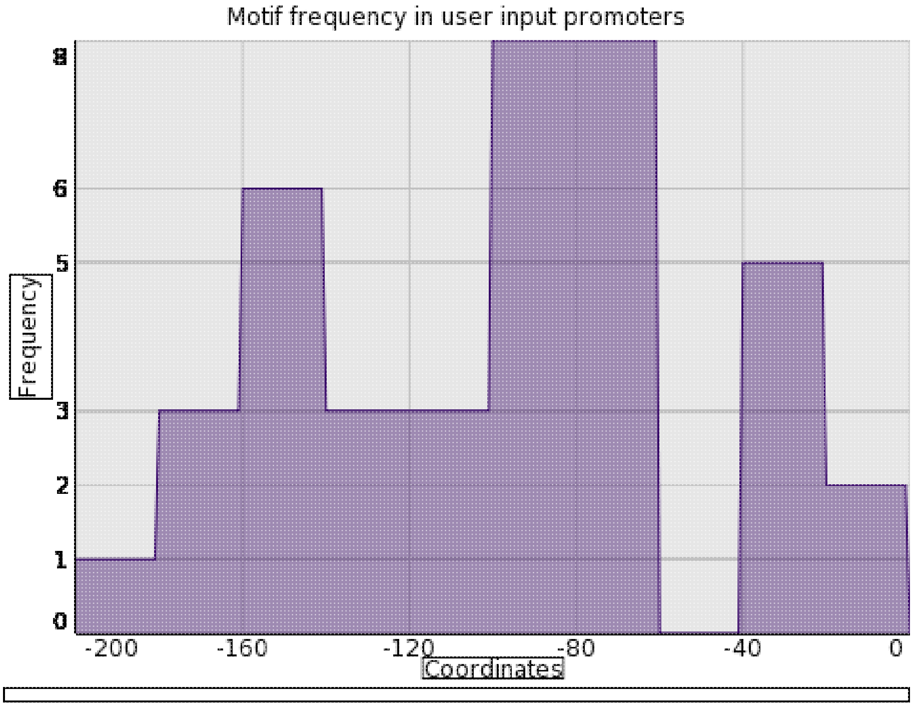


Motif6 annotation in the genome

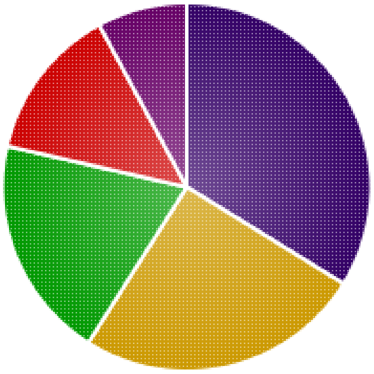

| Annotation | p-value |
|---|---|
| GO:0003676 | 4.53e-06 |
| GO:0046983 | 1.05e-04 |
| GO:0008270 | 7.97e-04 |
| GO:0005524 | 7.22e-03 |
| others | 8.08e-03 |

**Annotation complete description**

GO:0003676 => nucleic acid binding GO:0046983 => protein dimerization activity GO:0008270 => zinc ion binding GO:0005524 => ATP binding GO:0003677 => DNA binding GO:0005525 => GTP binding GO:0005622 => intracellular

Genome-wide Motif6 search results

Motif6 gene list of over-represented annotation(s)

---

# Motif7

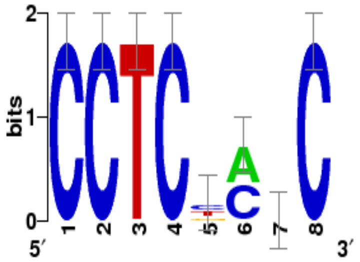


**CCTCYMAC**

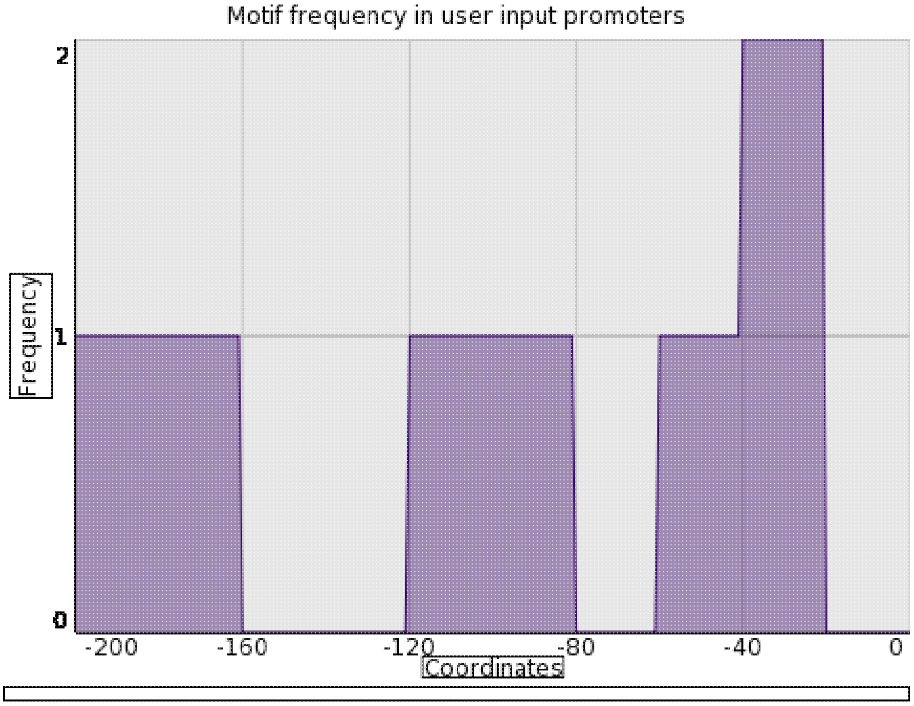


---

Motif7 annotation in the genome

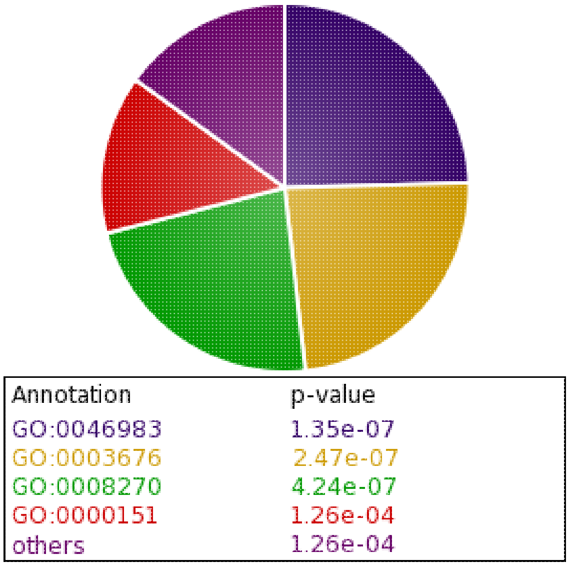


**Annotation complete description**

GO:0046983 => protein dimerization activity GO:0003676 => nucleic acid binding GO:0008270 => zinc ion binding GO:0000151 => ubiquitin ligase complex GO:0016567 => protein ubiquitination GO:0004842 => ubiquitin-protein ligase activity GO:0003677 => DNA binding

Genome-wide Motif7 search results

Motif7 gene list of over-represented annotation(s)

---

---

# Motif8

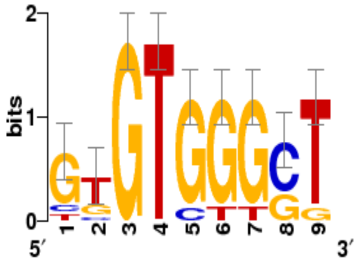


**GTGTGGGCT**

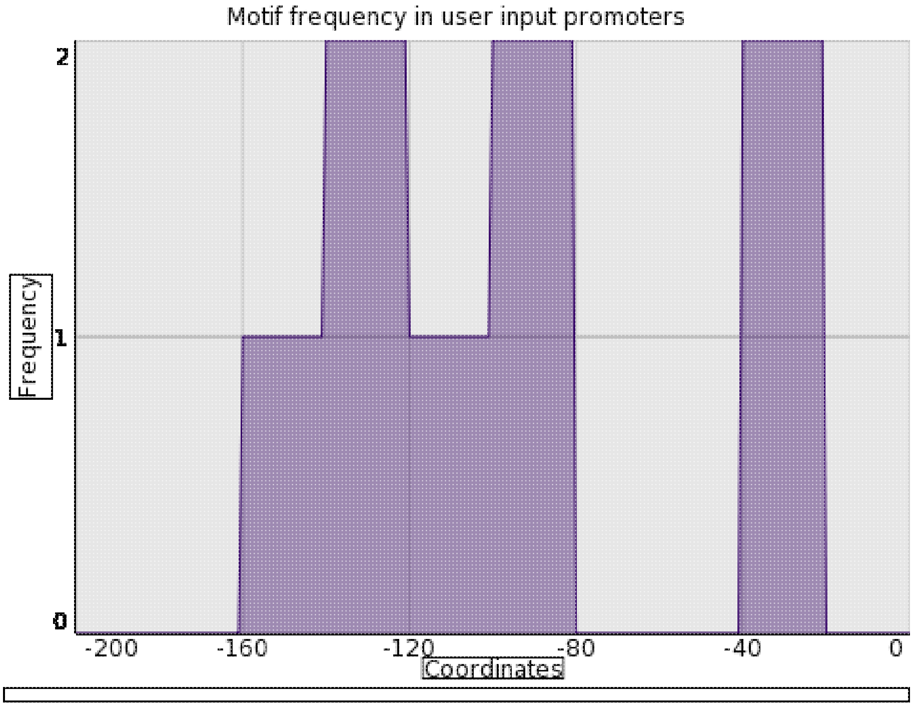


---

Motif8 annotation in the genome

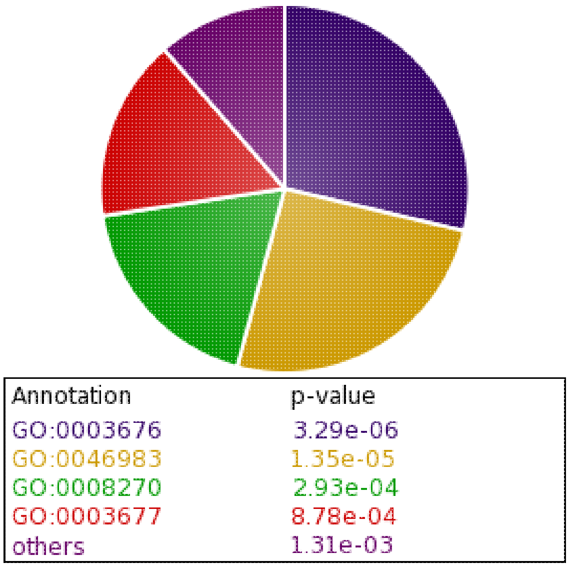


**Annotation complete description**

GO:0003676 => nucleic acid binding GO:0046983 => protein dimerization activity GO:0008270 => zinc ion binding GO:0003677 => DNA binding GO:0004379 => glycylpeptide N-tetradecanoyltransferase activity GO:0005525 => GTP binding GO:0045900 => negative regulation of translational elongation

Genome-wide Motif8 search results

Motif8 gene list of over-represented annotation(s)

---

# Motif9

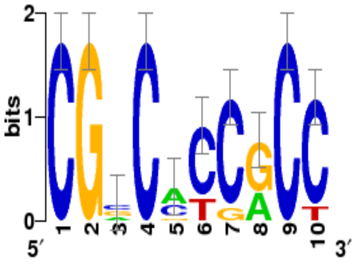


**CGSCACCGCC**

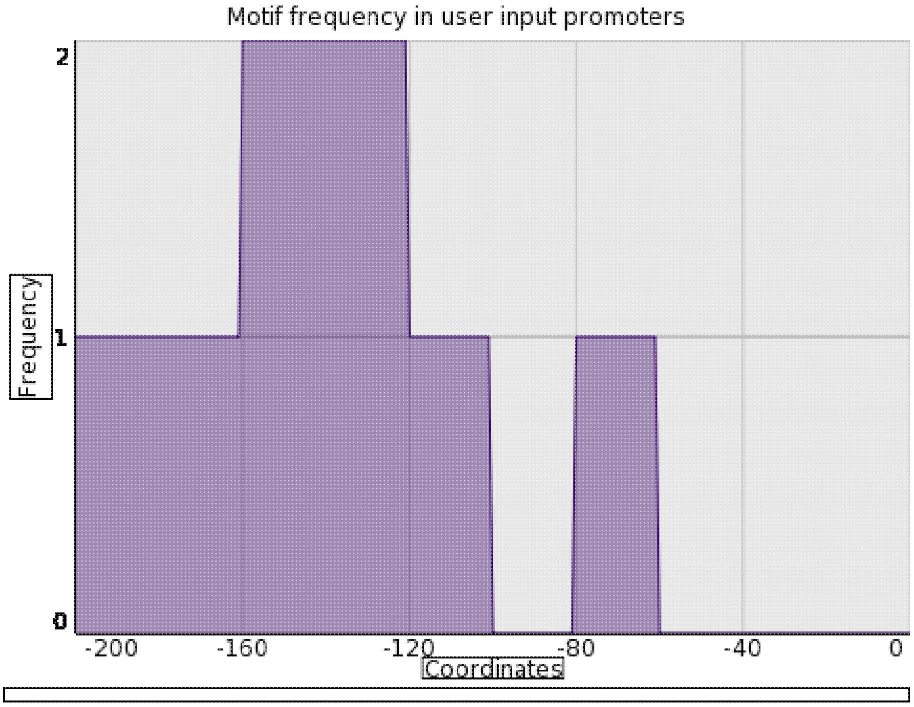


Motif9 annotation in the genome

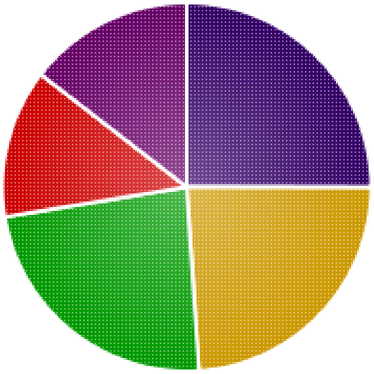

| Annotation | p-value |
|---|---|
| GO:0008270 | 3.03e-07 |
| GO:0046983 | 5.98e-07 |
| GO:0003676 | 7.77e-07 |
| GO:0003677 | 4.01e-04 |
| others | 1.73e-03 |

**Annotation complete description**

GO:0008270 => zinc ion binding GO:0046983 => protein dimerization activity GO:0003676 => nucleic acid binding GO:0003677 => DNA binding GO:0016021 => integral to membrane GO:0005524 => ATP binding GO:0005622 => intracellular

Genome-wide Motif9 search results

Motif9 gene list of over-represented annotation(s)

# Motif10

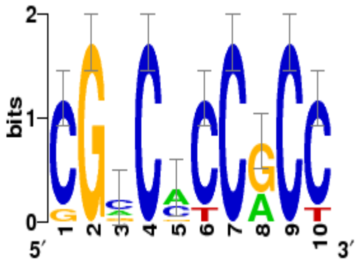


**CGCCACCGCC**

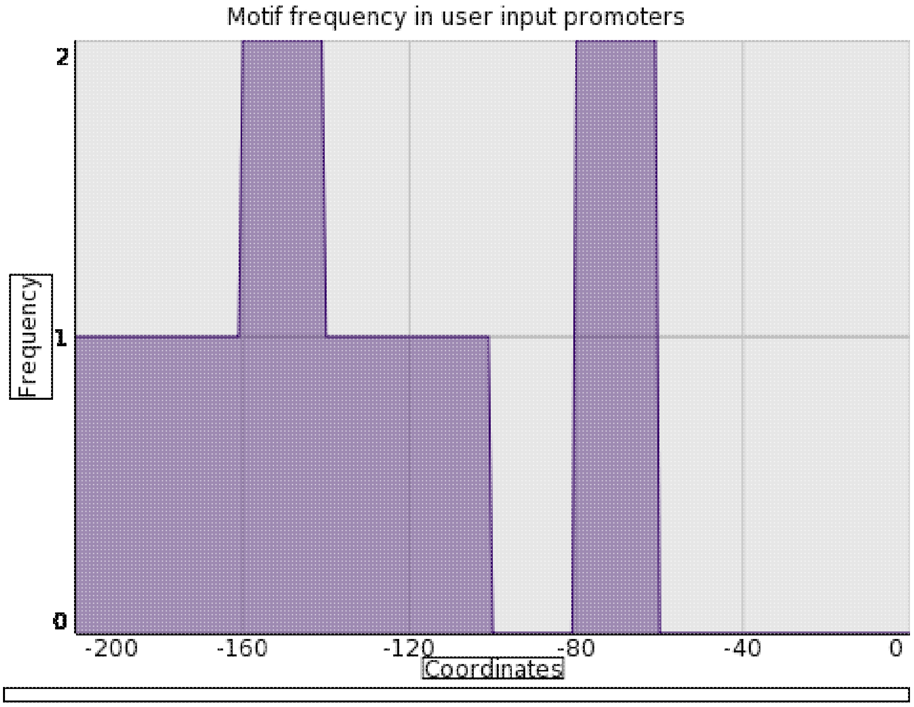


Motif10 annotation in the genome

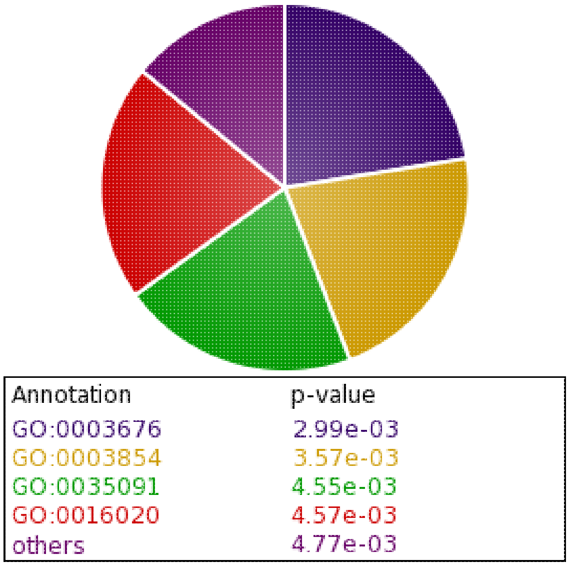


**Annotation complete description**

GO:0003676 => nucleic acid binding GO:0003854 => 3-beta-hydroxy-delta5-steroid dehydrogenase activity GO:0035091 => phosphoinositide binding GO:0016020 => membrane GO:0006694 => steroid biosynthetic process GO:0007154 => cell communication GO:0044237 => cellular metabolic process

Genome-wide Motif10 search results

Motif10 gene list of over-represented annotation(s)

---

# Motif11

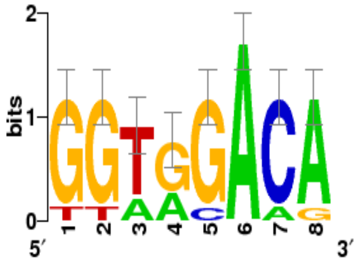


**GGTGGACA**

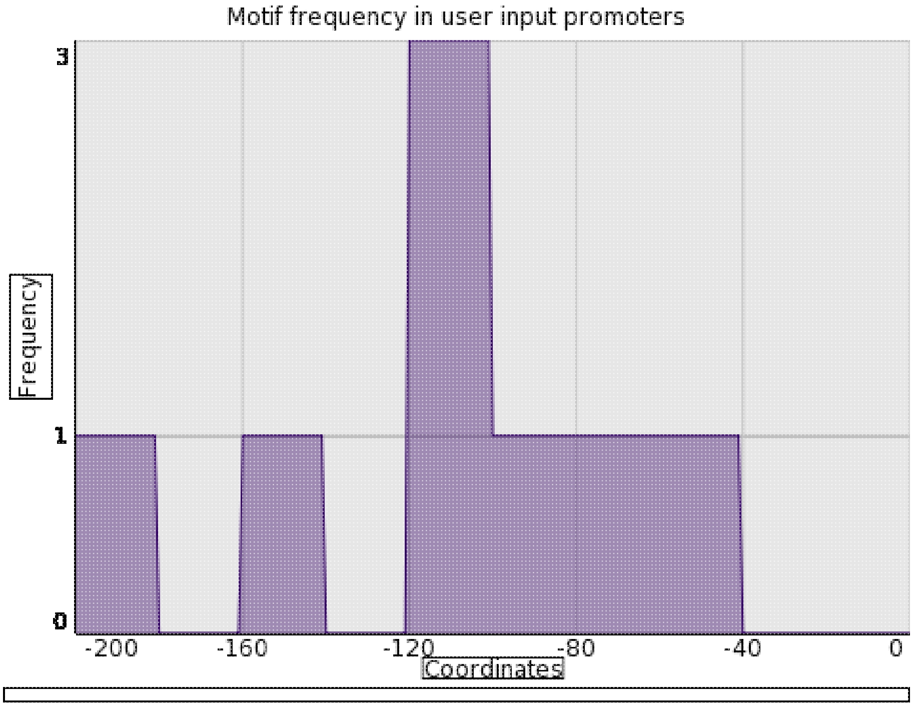


---

Motif11 annotation in the genome

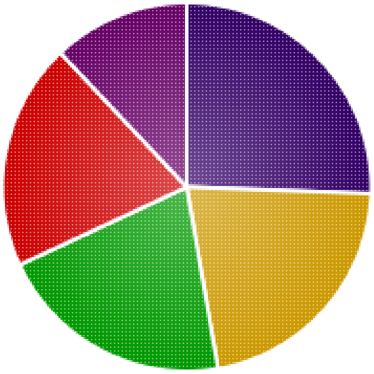

| Annotation | p-value |
|---|---|
| GO:0003676 | 1.08e-03 |
| GO:0006412 | 3.03e-03 |
| GO:0008270 | 3.89e-03 |
| GO:0016020 | 4.90e-03 |
| others | 4.90e-03 |

**Annotation complete description**

GO:0003676 => nucleic acid binding GO:0006412 => translation GO:0008270 => zinc ion binding GO:0016020 => membrane GO:0003735 => structural constituent of ribosome GO:0005840 => ribosome GO:0016021 => integral to membrane

Genome-wide Motif11 search results

Motif11 gene list of over-represented annotation(s)

---

---

# Motif12

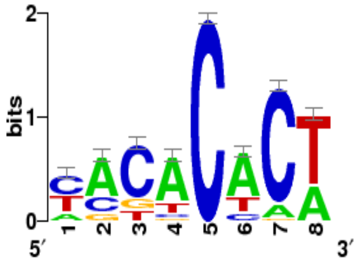


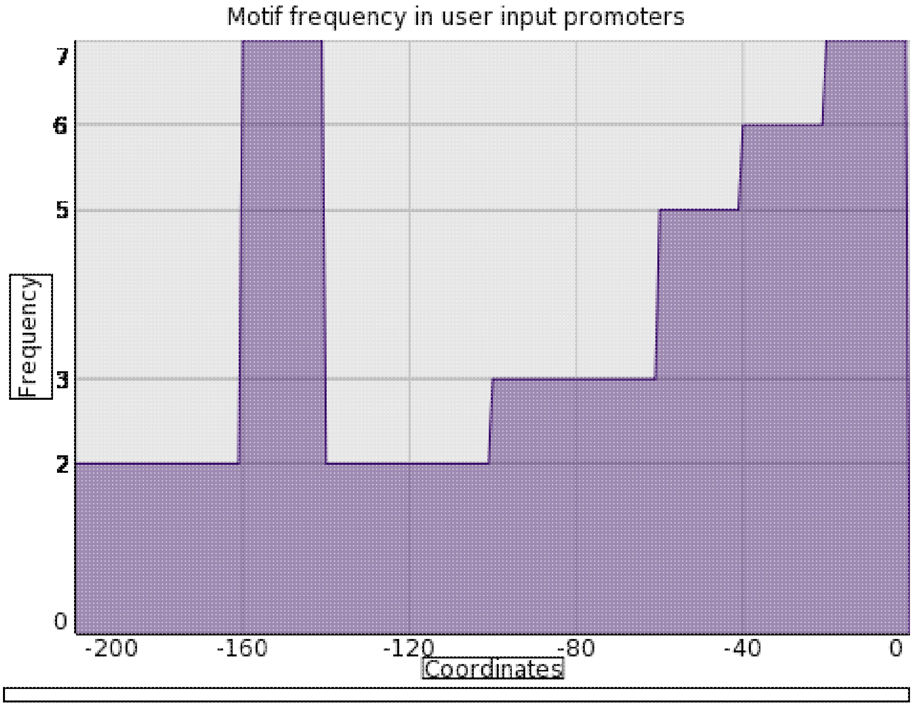


**CACACACT**

---

Motif12 annotation in the genome

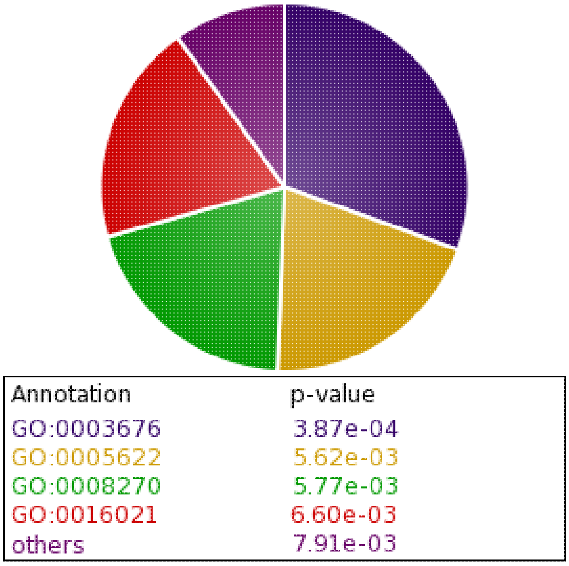


**Annotation complete description**

GO:0003676 => nucleic acid binding GO:0005622 => intracellular GO:0008270 => zinc ion binding GO:0016021 => integral to membrane GO:0035091 => phosphoinositide binding GO:0003735 => structural constituent of ribosome GO:0005840 => ribosome

Genome-wide Motif12 search results

Motif12 gene list of over-represented annotation(s)

---

# Motif13

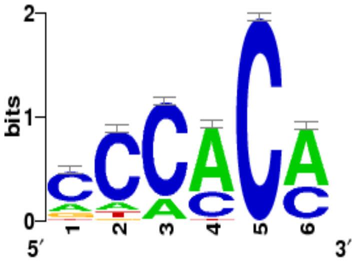


**CCCACA**

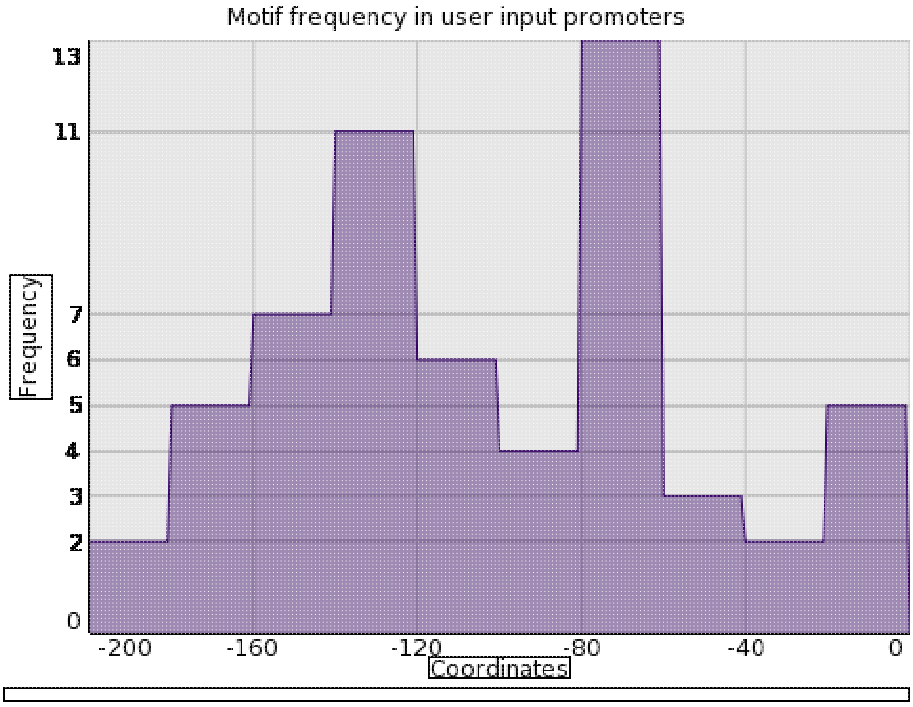


Motif13 annotation in the genome

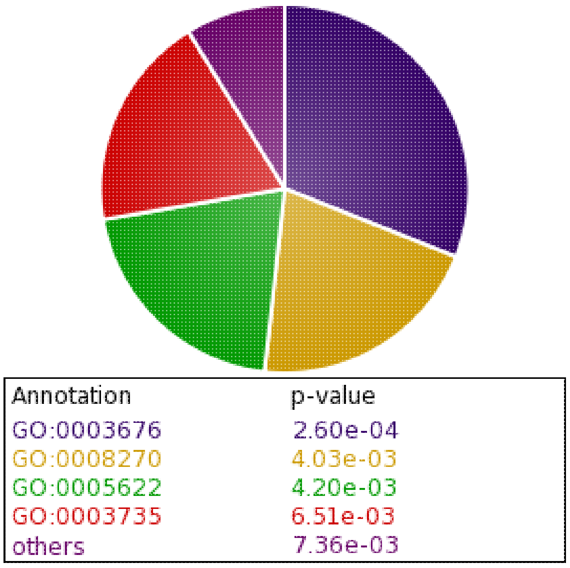


**Annotation complete description**

GO:0003676 => nucleic acid binding GO:0008270 => zinc ion binding GO:0005622 => intracellular GO:0003735 => structural constituent of ribosome GO:0005840 => ribosome GO:0006412 => translation GO:0035091 => phosphoinositide binding

Genome-wide Motif13 search results

Motif13 gene list of over-represented annotation(s)

---

---

# Motif14

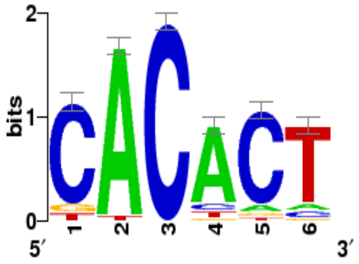


**CACACT**

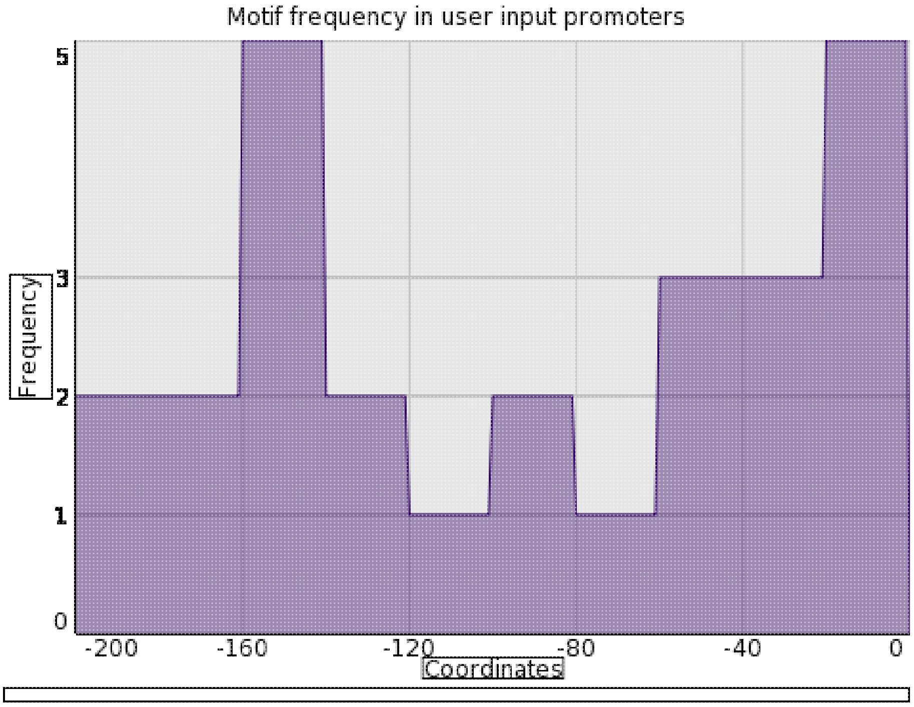


---

Motif14 annotation in the genome

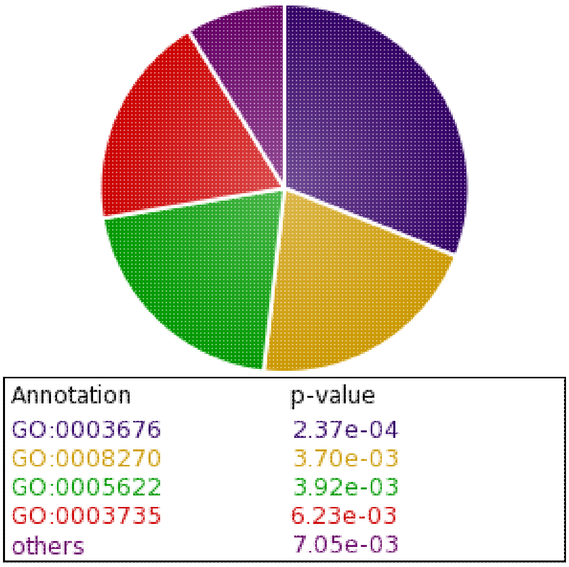


**Annotation complete description**

GO:0003676 => nucleic acid binding GO:0008270 => zinc ion binding GO:0005622 => intracellular GO:0003735 => structural constituent of ribosome GO:0005840 => ribosome GO:0006412 => translation GO:0035091 => phosphoinositide binding

Genome-wide Motif14 search results

Motif14 gene list of over-represented annotation(s)

Sequence logo generated by weblogo
Graphic generated with Chart::Clicker,Perl module
Promzea program from the Raizada lab
