# Supplementary material for: Promzea: a pipeline for discovery of co-regulatory motifs in maize and other plant species and its application to the anthocyanin and phlobaphene biosynthetic pathways and the Maize Development Atlas
Source: BMC Plant Biol. 2013 Mar 15;13:42. doi: 10.1186/1471-2229-13-42 (PMC3658923; doi:10.1186/1471-2229-13-42)
Supplement: Additional file 7 — Supplemental files for testing Promzea with data sets from the Maize Development Atlas. The zip folder contains 3 folders. The first contains the promoter input for Promzea for each maize tissue; the second folder has all the outputs from Promzea; the third folder contains the STAMP website outputs for comparisons of the predicted motifs with experimentally defined motifs. [file 1471-2229-13-42-S7.zip › Supplemental files 3 -Case study 3/2-Promzea results/leaf.pdf]

Home

Results Summary

/vbox_shared/1-case_study_3/casestudy3_leaf.txt

Promzea - 00000444

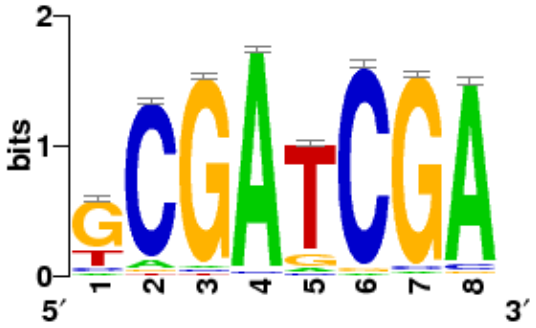

Motif1
(from Weeder)
score: 1.11

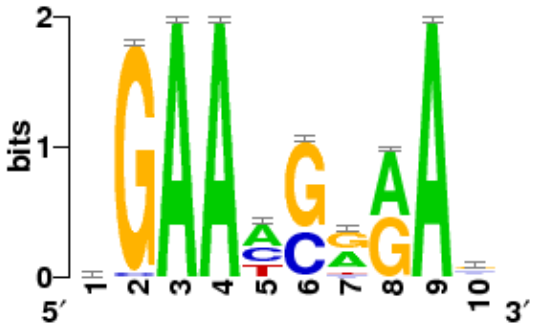

Motif2
(from BioP)
score: 1.1

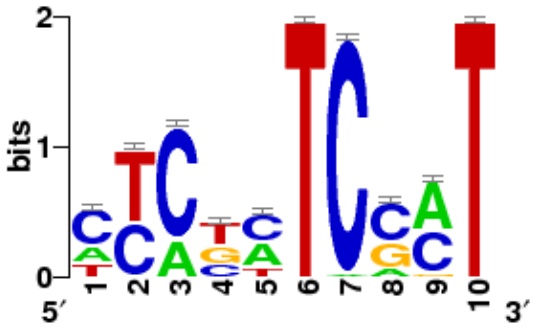

Motif3
(from BioP)
score: 1.08

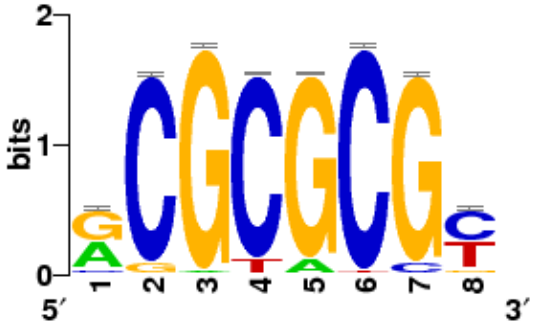

Motif4
(from Weeder)
score: 1.08

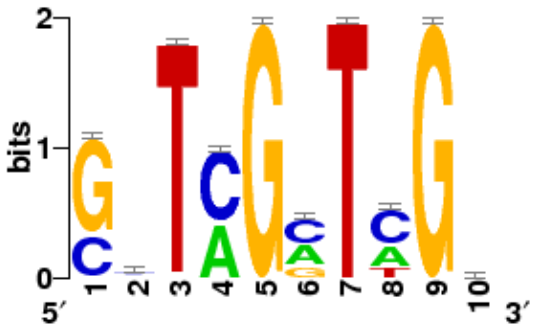

Motif5
(from BioP)
score: 1.06

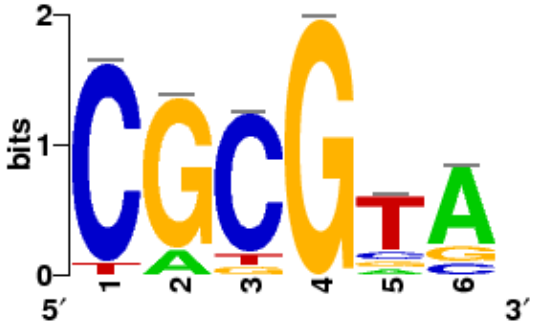

Motif6
(from Weeder)
score: 1.01

Compare your motifs to known promoter motif databases using STAMP website motif file to copy in STAMP website

Open the above link, copy content of the newly open file and paste in STAMP program link below In STAMP, under "Similarity Matching", we suggest selecting the plant motif databases: Athamap, AGRIS, PLACE, TRANSFAC; then submit

STAMP website

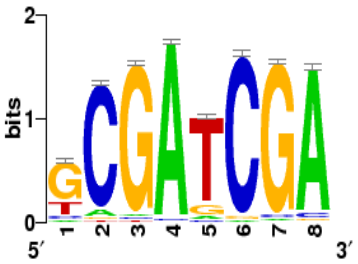

GCGATCGA

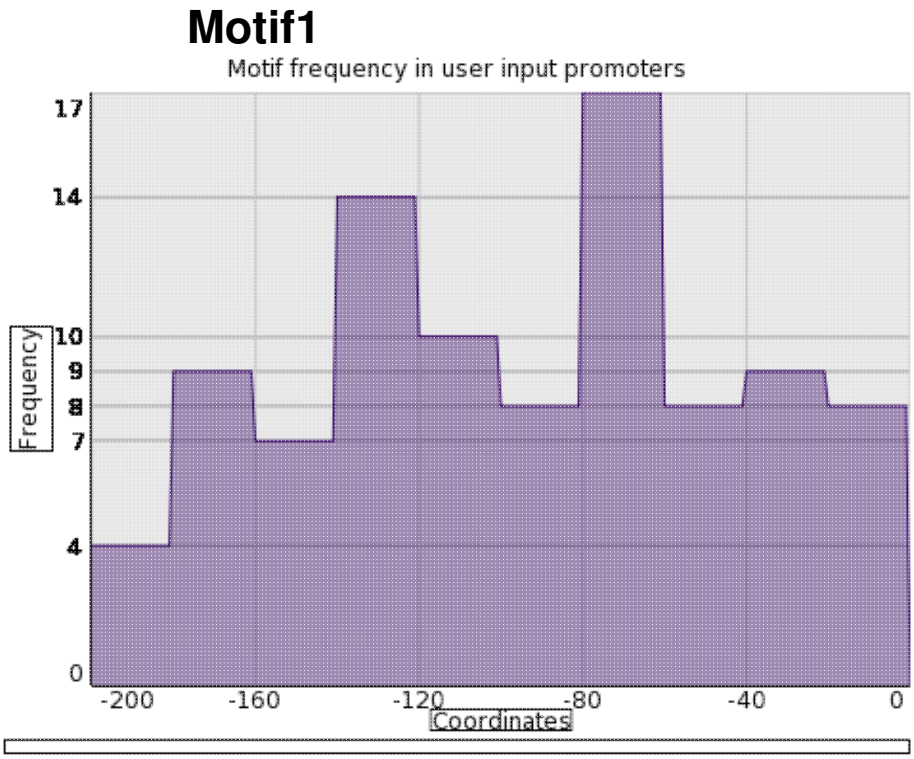

Motif1 annotation in the genome

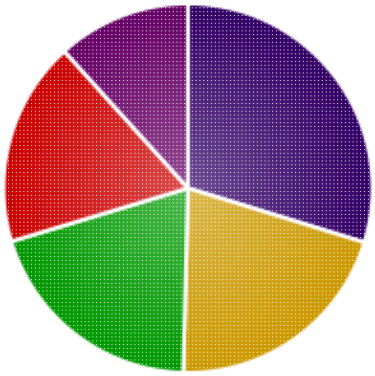

Annotation	p-value
GO:0003676	2.28e-05
GO:0005515	5.96e-04
GO:0008270	7.77e-04
GO:0004452	1.57e-03
others	1.82e-03

Annotation complete description

GO:0003676 => nucleic acid binding GO:0005515 => protein binding GO:0008270 => zinc ion binding GO:0004452 => isopentenyl-diphosphate delta-isomerase activity GO:0046983 => protein dimerization activity GO:0003677 => DNA binding GO:0005576 => extracellular region

Genome-wide Motif1 search results

Motif1 gene list of over-represented annotation(s)

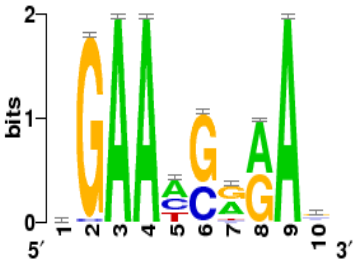

TGAAAGGAAG

Motif2

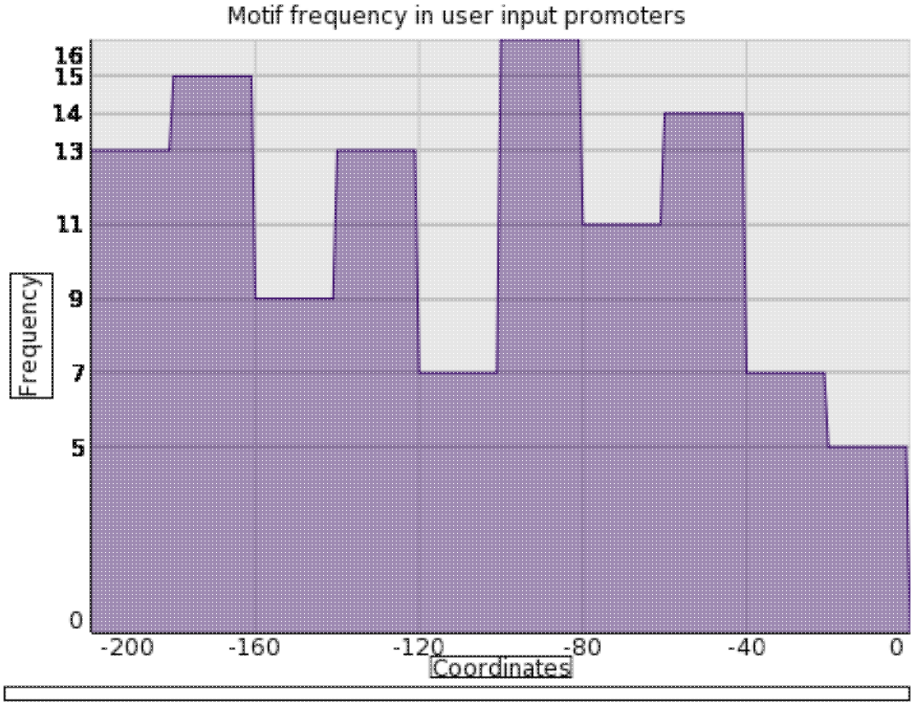

Motif2 annotation in the genome

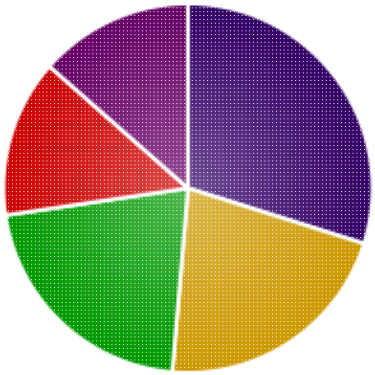

Annotation	p-value
GO:0003676	6.10e-08
GO:0008270	6.40e-06
GO:0003677	7.12e-06
GO:0005515	4.56e-04
others	9.86e-04

Annotation complete description

GO:0003676 => nucleic acid binding GO:0008270 => zinc ion binding GO:0003677 => DNA binding GO:0005515 => protein binding GO:0030528 => transcription regulator activity GO:0016787 => hydrolase activity GO:0006259 => DNA metabolic process

Genome-wide Motif2 search results

Motif2 gene list of over-represented annotation(s)

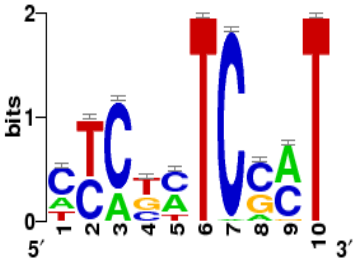

CTCTCTCCAT

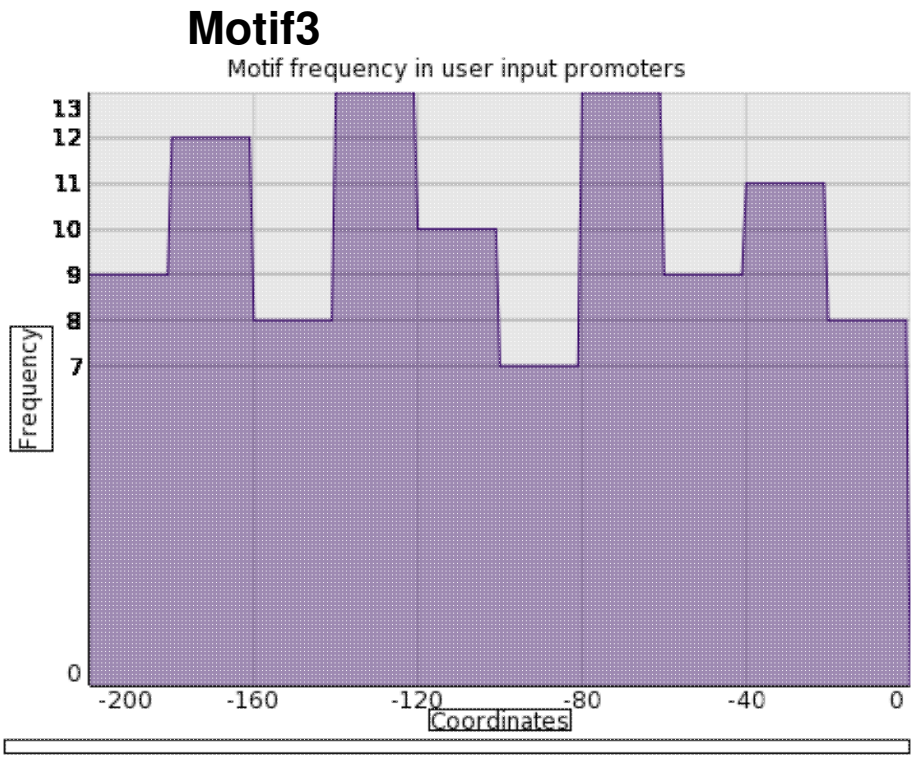

Motif3 annotation in the genome

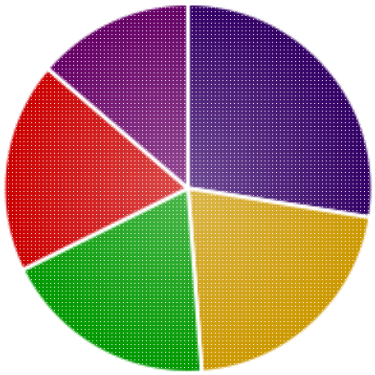

Annotation	p-value
GO:0003676	9.37e-07
GO:0008270	2.19e-05
GO:0003677	7.30e-05
GO:0005515	8.75e-05
others	1.60e-03

Annotation complete description

GO:0003676 => nucleic acid binding GO:0008270 => zinc ion binding GO:0003677 => DNA binding GO:0005515 => protein binding GO:0004008 => copper-exporting ATPase activity GO:0046983 => protein dimerization activity GO:0016820 => hydrolase activity, acting on acid anhydrides, catalyzing transmembrane movement of substances

Genome-wide Motif3 search results

Motif3 gene list of over-represented annotation(s)

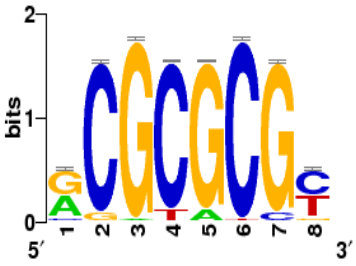

GCGCGCGC

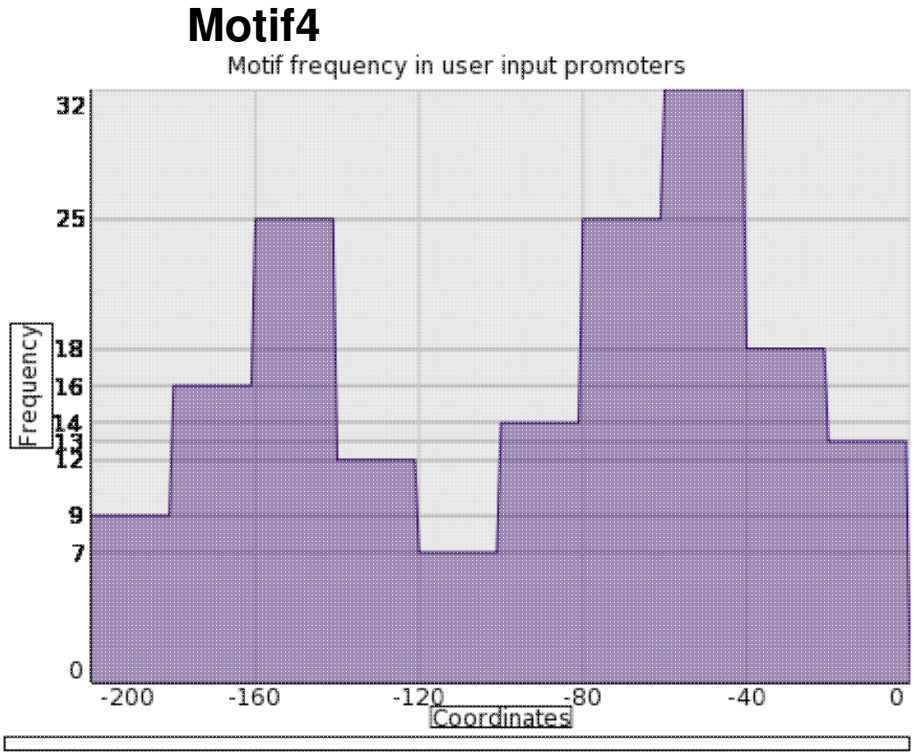

Motif4 annotation in the genome

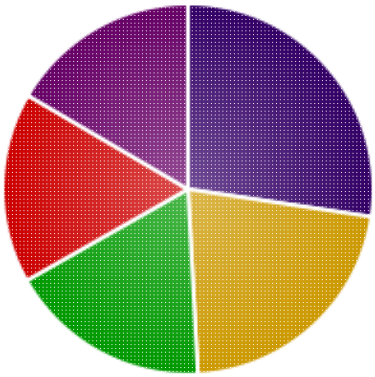

Annotation	p-value
GO:0003677	1.99e-04
GO:0006879	1.14e-03
GO:0019898	4.11e-03
GO:0009654	5.64e-03
GO:0019211	5.75e-03

Annotation complete description

GO:0003677 => DNA binding GO:0006879 => cellular iron ion homeostasis GO:0019898 => extrinsic to membrane GO:0009654 => oxygen evolving complex GO:0019211 => phosphatase activator activity

Genome-wide Motif4 search results

Motif4 gene list of over-represented annotation(s)

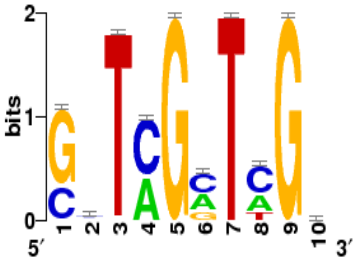

GCTCGCTCGC

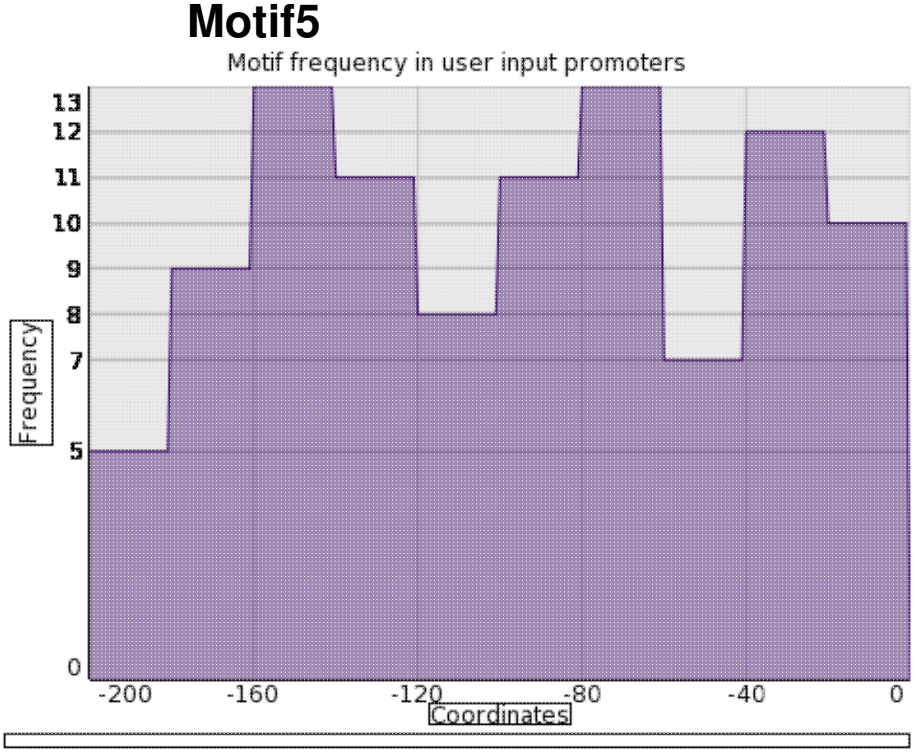

Motif5 annotation in the genome

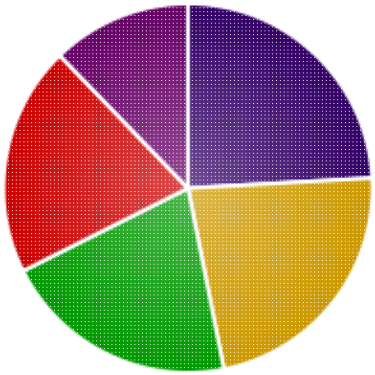

Annotation	p-value
GO:0004452	5.02e-04
GO:0003677	7.73e-04
GO:0008270	1.47e-03
GO:0005515	1.74e-03
others	2.41e-03

Annotation complete description

GO:0004452 => isopentenyl-diphosphate delta-isomerase activity GO:0003677 => DNA binding GO:0008270 => zinc ion binding GO:0005515 => protein binding GO:0009966 => regulation of signal transduction GO:0055085 => transmembrane transport GO:0003676 => nucleic acid binding

Genome-wide Motif5 search results

Motif5 gene list of over-represented annotation(s)

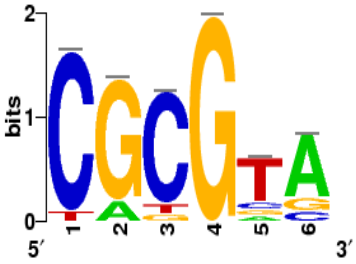

CGCGTA

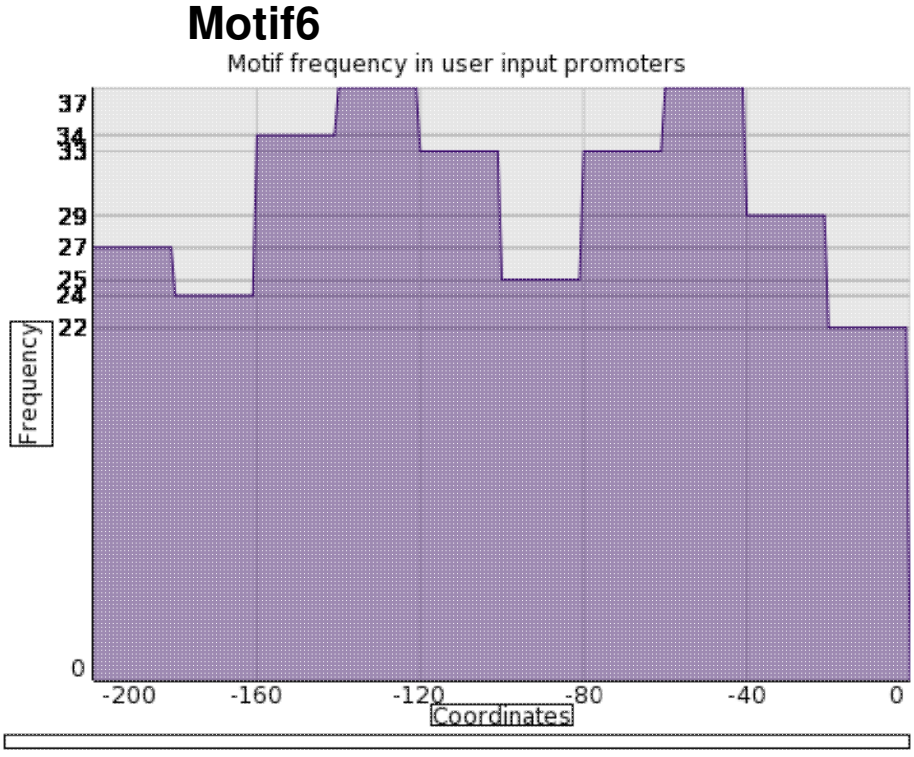

Motif6 annotation in the genome

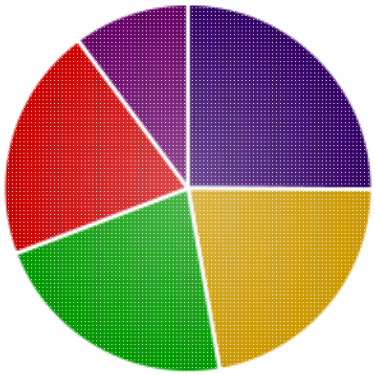

Annotation	p-value
GO:0047800	1.39e-03
GO:0006839	3.02e-03
GO:0009055	3.12e-03
GO:0004175	4.52e-03
others	4.90e-03

Annotation complete description

GO:0047800 => cysteamine dioxygenase activity GO:0006839 => mitochondrial transport GO:0009055 => electron carrier activity GO:0004175 => endopeptidase activity GO:0004298 => threonine-type endopeptidase activity GO:0005839 => proteasome core complex GO:0051603 => proteolysis involved in cellular protein catabolic process

Genome-wide Motif6 search results

Motif6 gene list of over-represented annotation(s)

Sequence logo generated by weblogo
Graphic generated with Chart::Clicker,Perl module
Promzea program from the Raizada lab
