# Supplementary material for: Promzea: a pipeline for discovery of co-regulatory motifs in maize and other plant species and its application to the anthocyanin and phlobaphene biosynthetic pathways and the Maize Development Atlas
Source: BMC Plant Biol. 2013 Mar 15;13:42. doi: 10.1186/1471-2229-13-42 (PMC3658923; doi:10.1186/1471-2229-13-42)
Supplement: Additional file 7 — Supplemental files for testing Promzea with data sets from the Maize Development Atlas. The zip folder contains 3 folders. The first contains the promoter input for Promzea for each maize tissue; the second folder has all the outputs from Promzea; the third folder contains the STAMP website outputs for comparisons of the predicted motifs with experimentally defined motifs. [file 1471-2229-13-42-S7.zip › Supplemental files 3 -Case study 3/2-Promzea results/root.pdf]

Home

Results Summary

/vbox_shared/1-case_study_3/casestudy3_root.txt

Promzea - 00000445

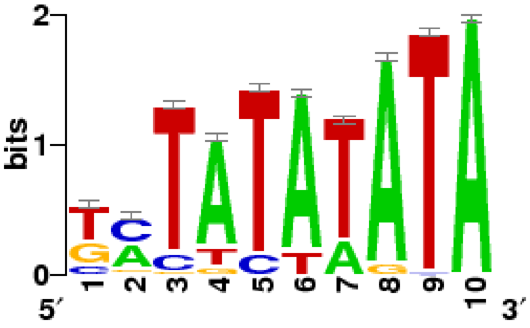

Motif1
(from MEME)
score: 2.05

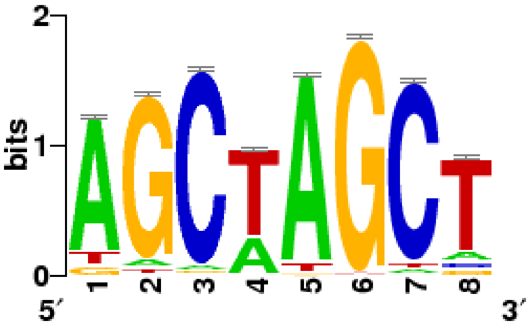

Motif2
(from Weeder)
score: 1.88

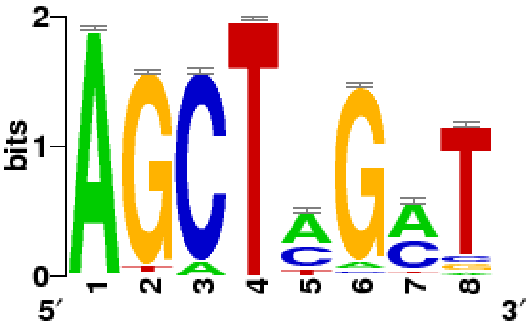

Motif3
(from Weeder)
score: 1.87

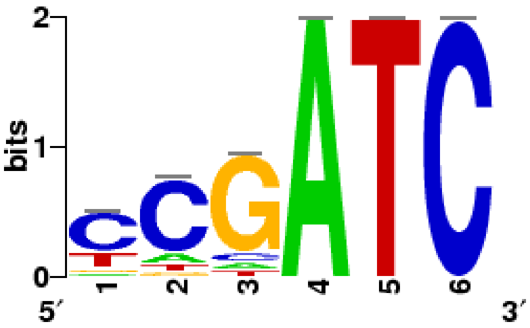

Motif4
(from Weeder)
score: 1.78

results - 00000445

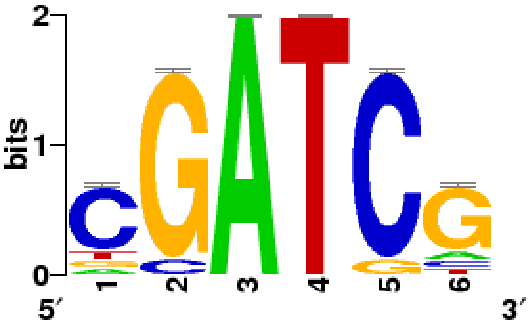

Motif5
(from Weeder)
score: 1.77

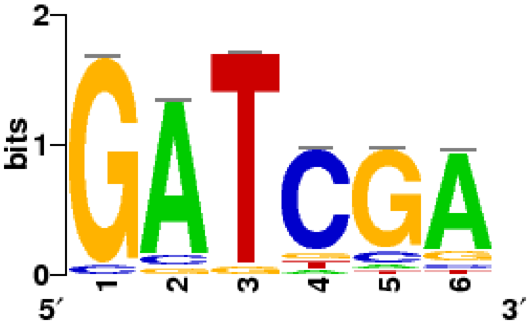

Motif6
(from Weeder)
score: 1.77

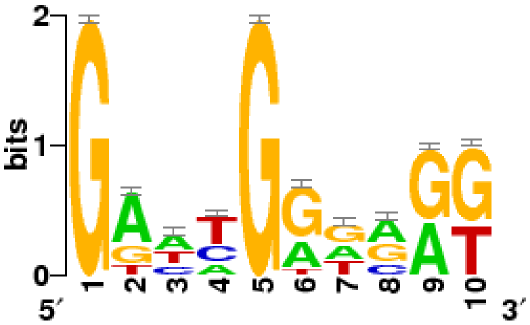

Motif7
(from BioP)
score: 1.76

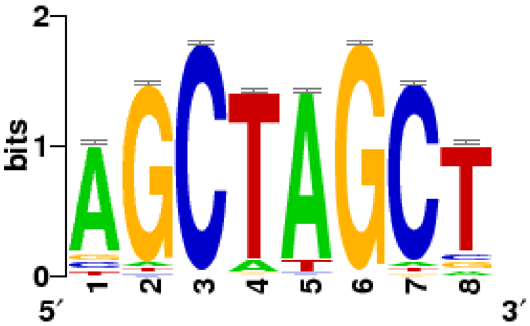

Motif8
(from Weeder)
score: 1.7

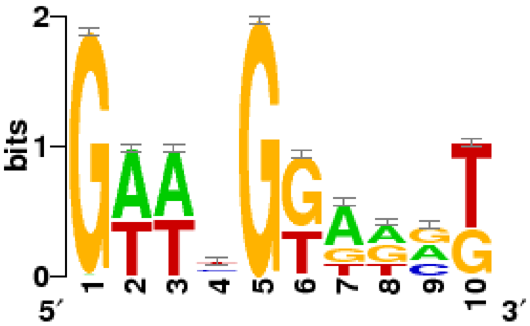

Motif9
(from BioP)
score: 1.57

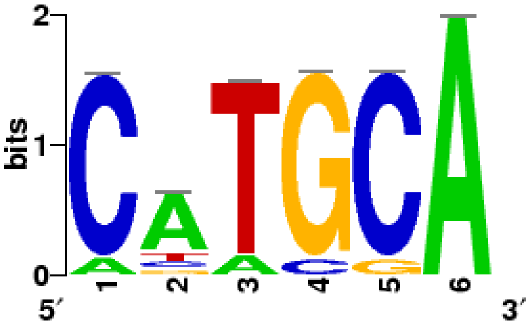

Motif10
(from Weeder)
score: 1.57

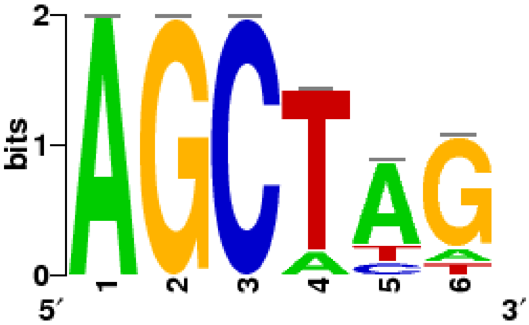

Motif11
(from Weeder)
score: 1.56

Compare your motifs to known promoter motif databases using STAMP website motif file to copy in STAMP website

Open the above link, copy content of the newly open file and paste in STAMP program link below In STAMP, under "Similarity Matching", we suggest selecting the plant motif databases: Athamap, AGRIS, PLACE, TRANSFAC; then submit

STAMP website

Motif1

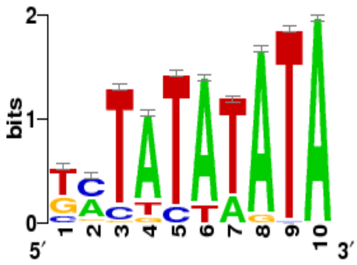

TCTATATATA

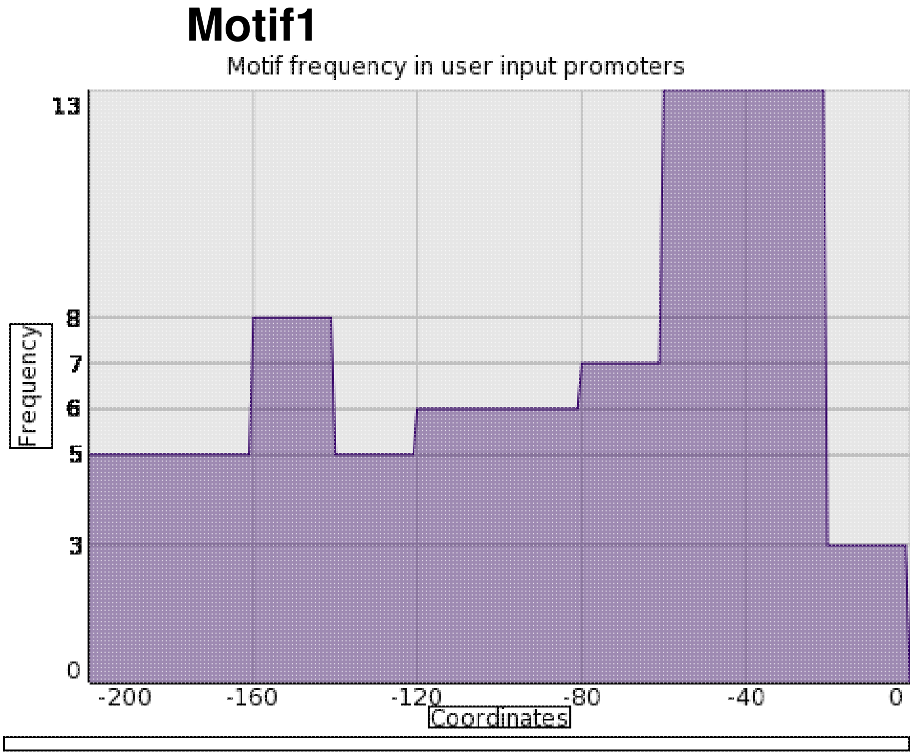

Motif1 annotation in the genome

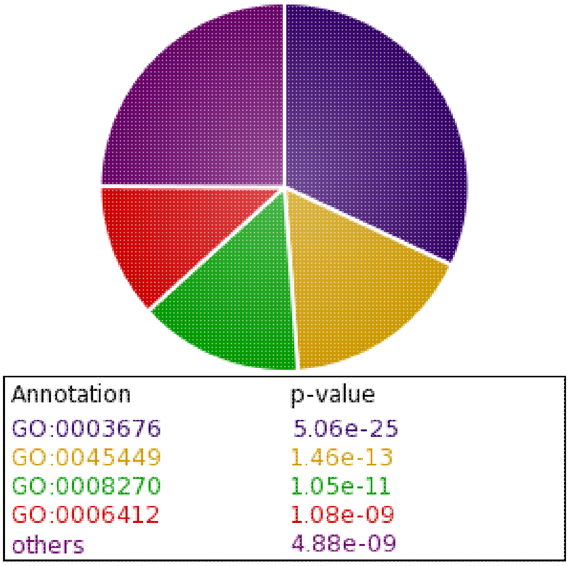

Annotation complete description

GO:0003676 => nucleic acid binding GO:0045449 => regulation of transcription GO:0008270 => zinc ion binding GO:0006412 => translation GO:0005524 => ATP binding GO:0046983 => protein dimerization activity GO:0048046 => apoplast

Genome-wide Motif1 search results

Motif1 gene list of over-represented annotation(s)

Motif2

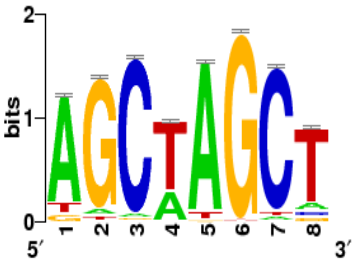

AGCTAGCT

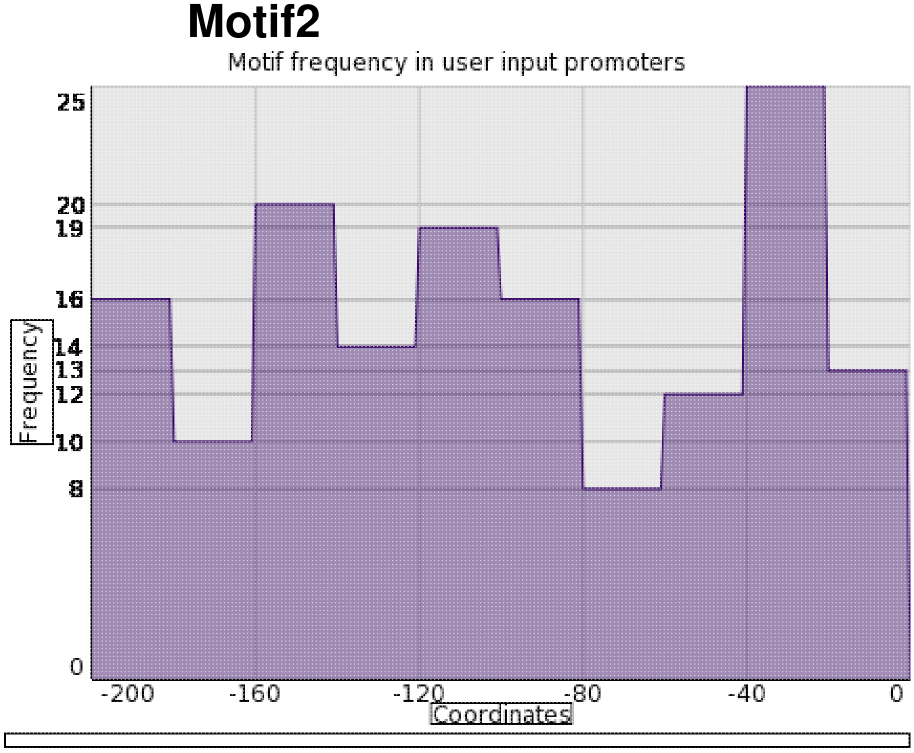

Motif2 annotation in the genome

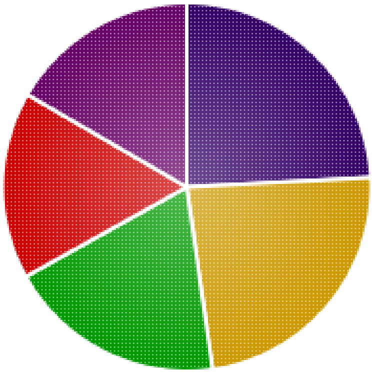

Annotation	p-value
GO:0045449	3.63e-10
GO:0003676	6.19e-10
GO:0003700	3.32e-08
GO:0043565	3.76e-07
others	1.82e-05

Annotation complete description

GO:0045449 => regulation of transcription GO:0003676 => nucleic acid binding GO:0003700 => transcription factor activity GO:0043565 => sequence-specific DNA binding GO:0006412 => translation GO:0006073 => cellular glucan metabolic process GO:0016762 => xyloglucan:xyloglucosyl transferase activity

Genome-wide Motif2 search results

Motif2 gene list of over-represented annotation(s)

Motif3

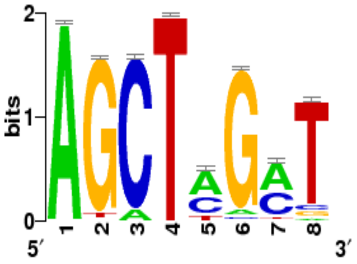

AGCTAGAT

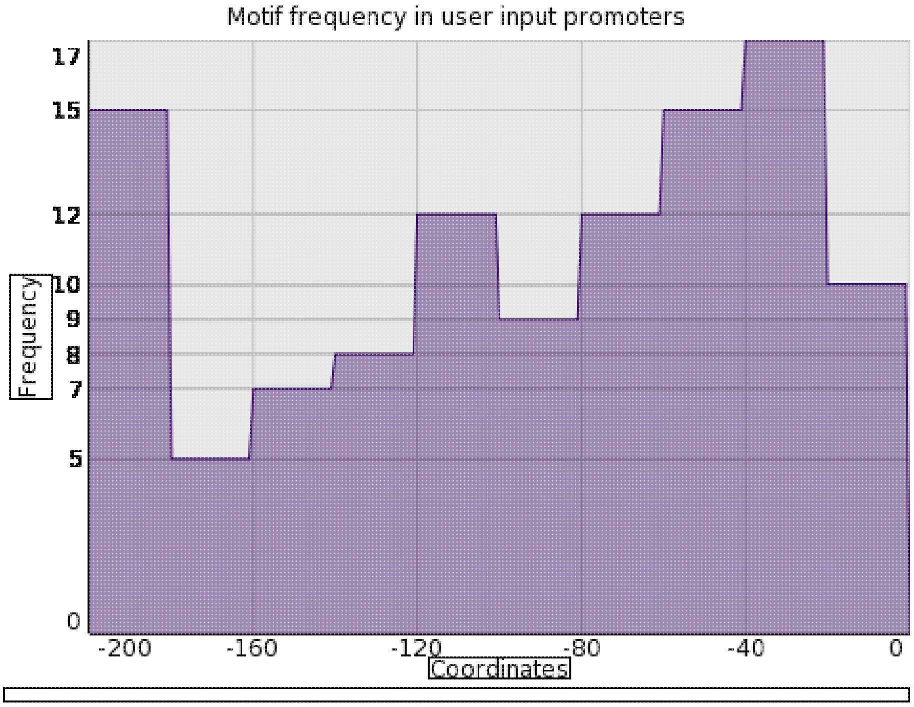

Motif3 annotation in the genome

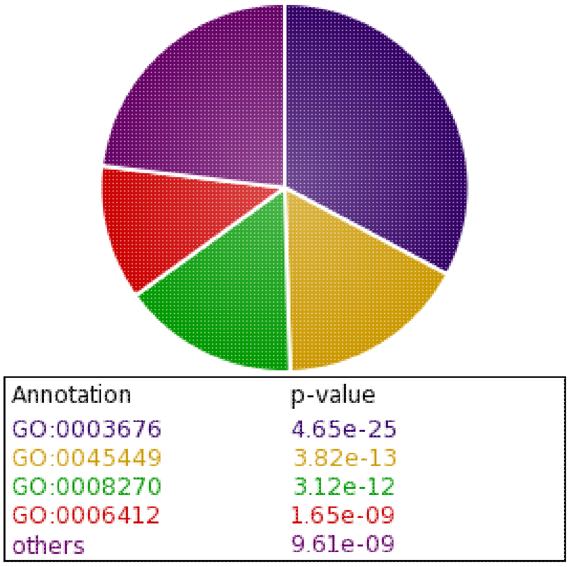

Annotation complete description

GO:0003676 => nucleic acid binding GO:0045449 => regulation of transcription GO:0008270 => zinc ion binding GO:0006412 => translation GO:0046983 => protein dimerization activity GO:0005524 => ATP binding GO:0048046 => apoplast

Genome-wide Motif3 search results

Motif3 gene list of over-represented annotation(s)

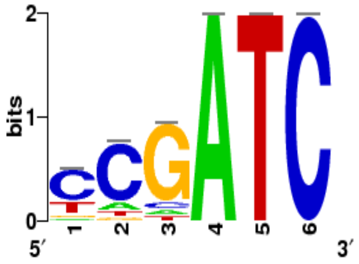

CCGATC

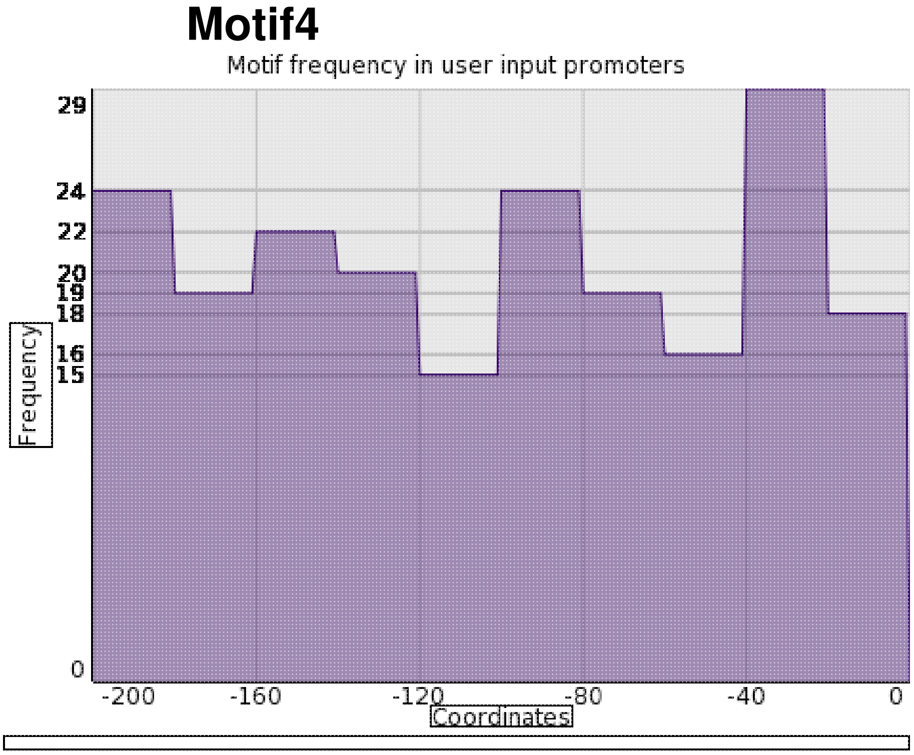

Motif4 annotation in the genome

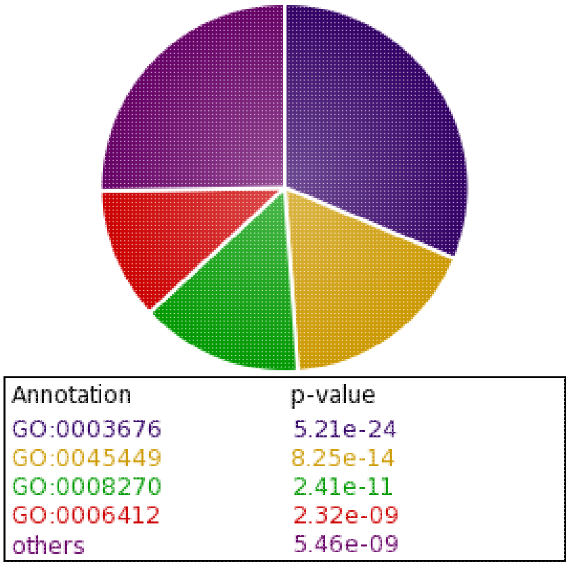

Annotation complete description

GO:0003676 => nucleic acid binding GO:0045449 => regulation of transcription GO:0008270 => zinc ion binding GO:0006412 => translation GO:0005524 => ATP binding GO:0046983 => protein dimerization activity GO:0048046 => apoplast

Genome-wide Motif4 search results

Motif4 gene list of over-represented annotation(s)

Motif5

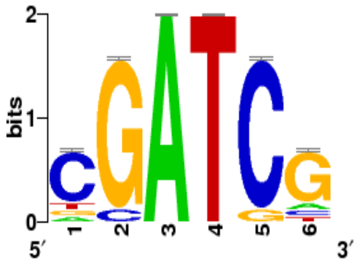

CGATCG

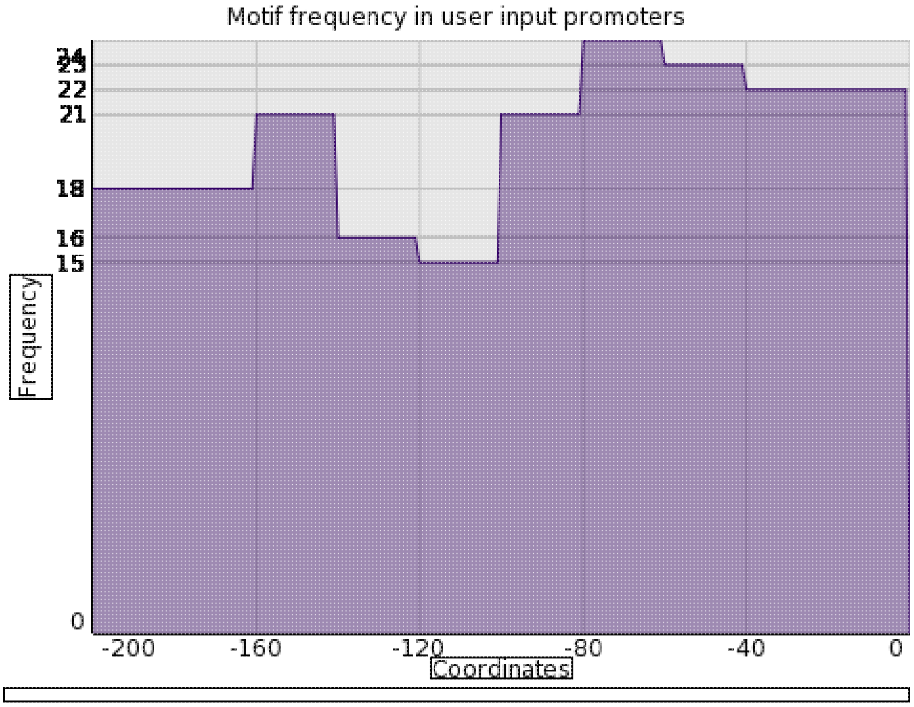

Motif5 annotation in the genome

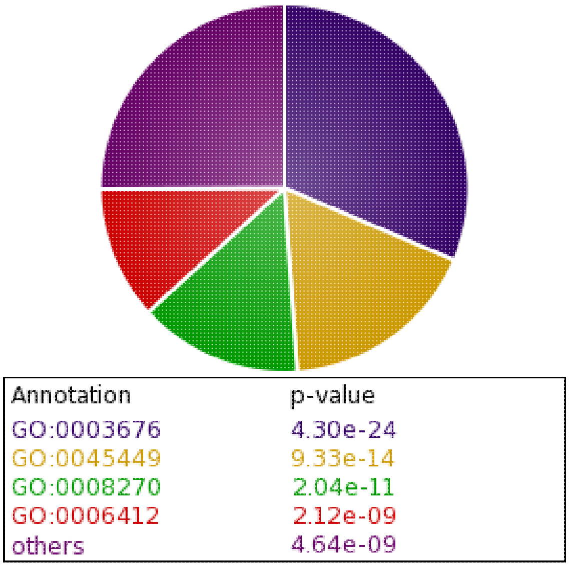

Annotation complete description

GO:0003676 => nucleic acid binding GO:0045449 => regulation of transcription GO:0008270 => zinc ion binding GO:0006412 => translation GO:0005524 => ATP binding GO:0046983 => protein dimerization activity GO:0048046 => apoplast

Genome-wide Motif5 search results

Motif5 gene list of over-represented annotation(s)

Motif6

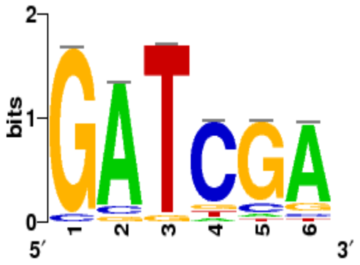

GATCGA

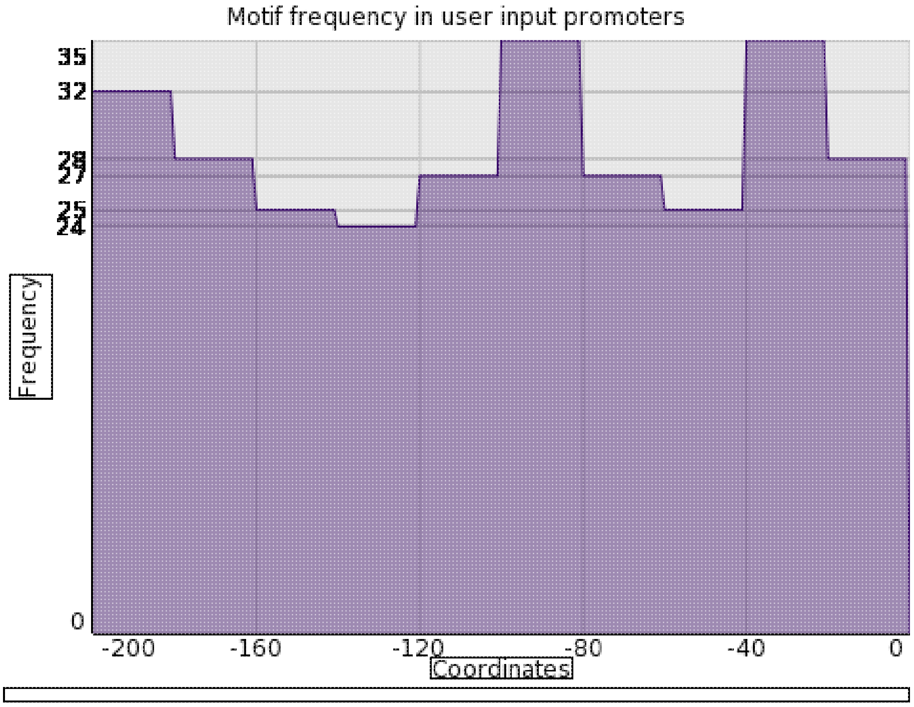

Motif6 annotation in the genome

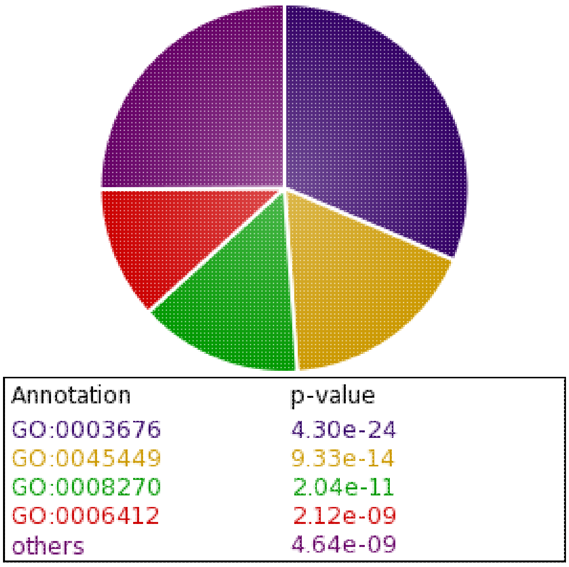

Annotation complete description

GO:0003676 => nucleic acid binding GO:0045449 => regulation of transcription GO:0008270 => zinc ion binding GO:0006412 => translation GO:0005524 => ATP binding GO:0046983 => protein dimerization activity GO:0048046 => apoplast

Genome-wide Motif6 search results

Motif6 gene list of over-represented annotation(s)

Motif7

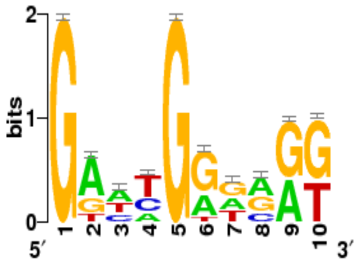

GAATGGGAGG

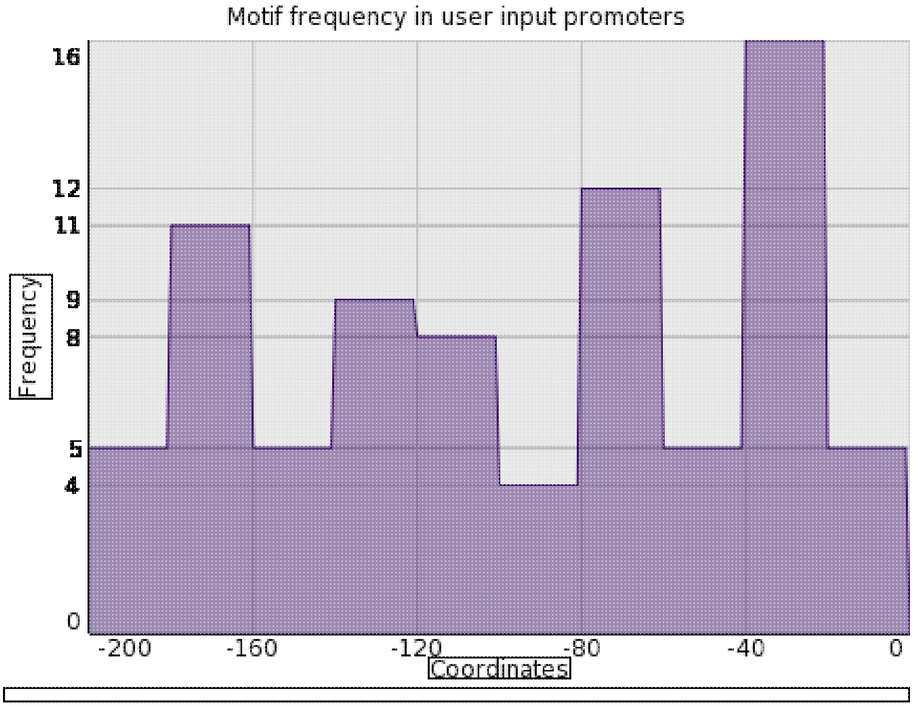

Motif7 annotation in the genome

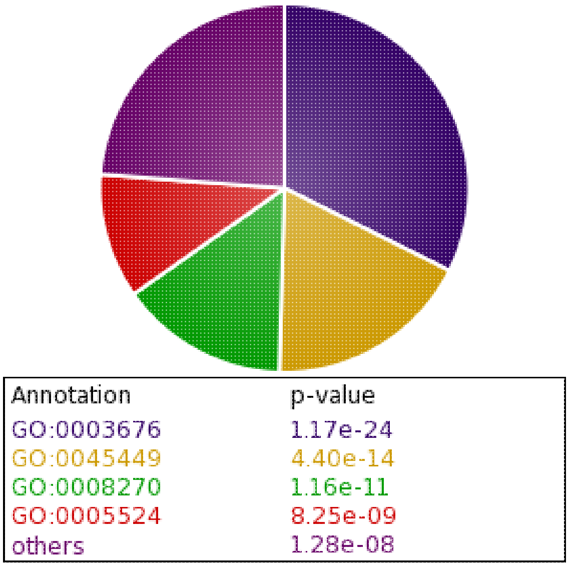

Annotation complete description

GO:0003676 => nucleic acid binding GO:0045449 => regulation of transcription GO:0008270 => zinc ion binding GO:0005524 => ATP binding GO:0006412 => translation GO:0046983 => protein dimerization activity GO:0048046 => apoplast

Genome-wide Motif7 search results

Motif7 gene list of over-represented annotation(s)

Motif8

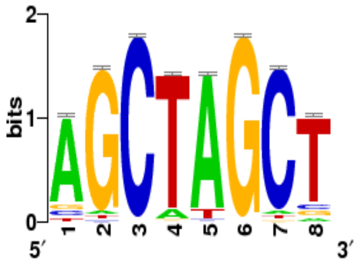

AGCTAGCT

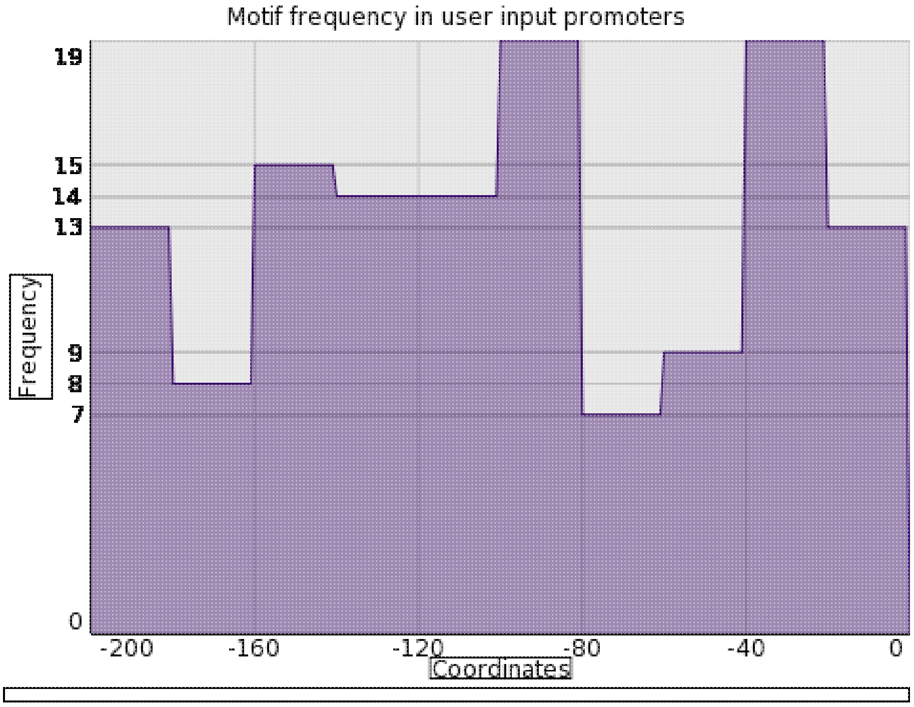

Motif8 annotation in the genome

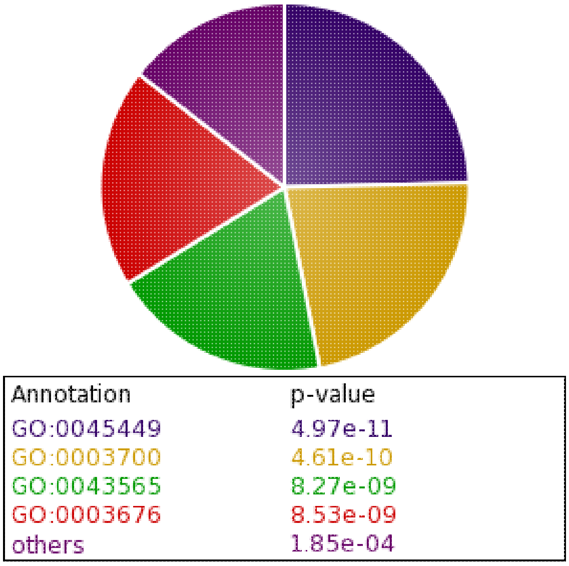

Annotation complete description

GO:0045449 => regulation of transcription GO:0003700 => transcription factor activity GO:0043565 => sequence-specific DNA binding GO:0003676 => nucleic acid binding GO:0048046 => apoplast GO:0003677 => DNA binding GO:0006073 => cellular glucan metabolic process

Genome-wide Motif8 search results

Motif8 gene list of over-represented annotation(s)

Motif9

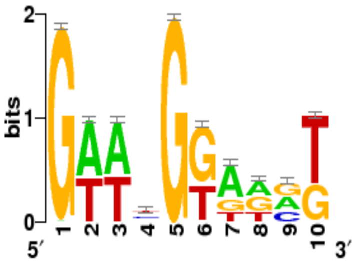

GAATGGARGT

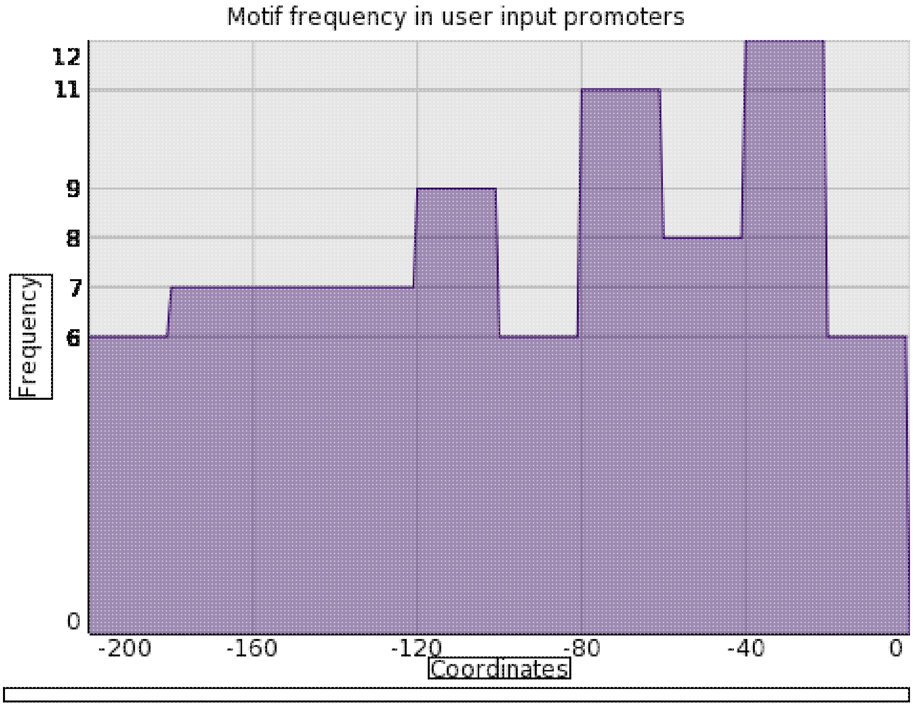

Motif9 annotation in the genome

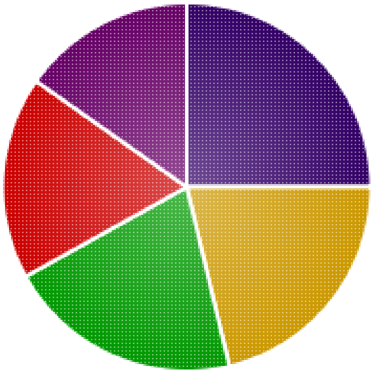

Annotation	p-value
GO:0045449	1.31e-10
GO:0003700	3.81e-09
GO:0003676	6.86e-09
GO:0043565	8.90e-08
others	1.51e-04

Annotation complete description

GO:0045449 => regulation of transcription GO:0003700 => transcription factor activity GO:0003676 => nucleic acid binding GO:0043565 => sequence-specific DNA binding GO:0006412 => translation GO:0003677 => DNA binding GO:0048046 => apoplast

Genome-wide Motif9 search results

Motif9 gene list of over-represented annotation(s)

Motif10

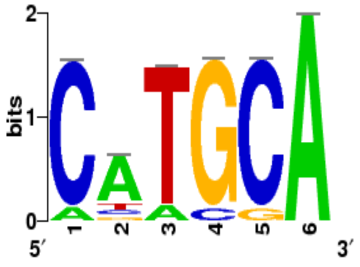

CATGCA

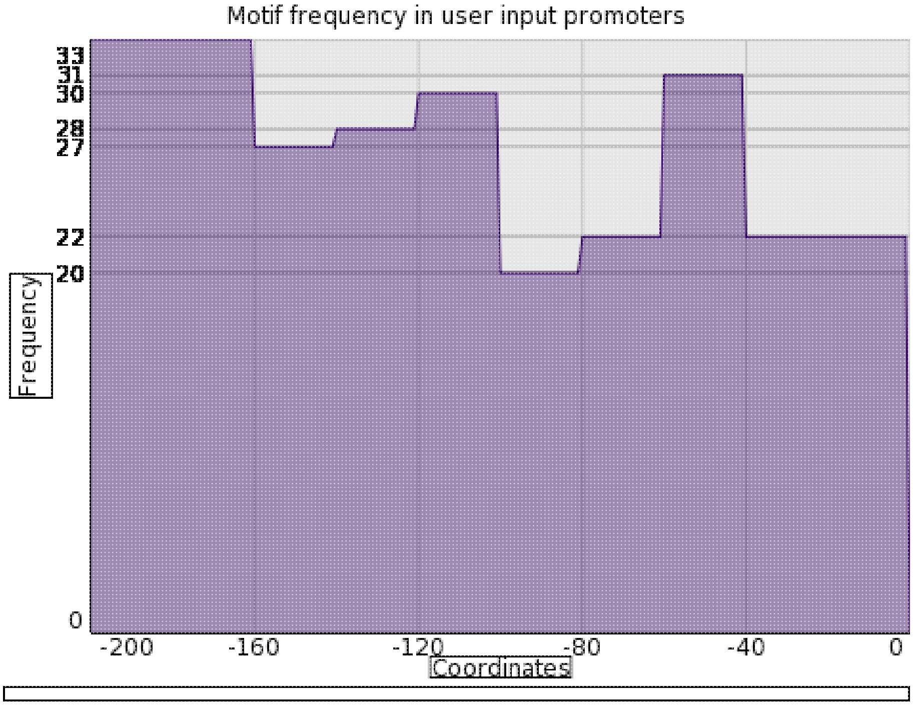

Motif10 annotation in the genome

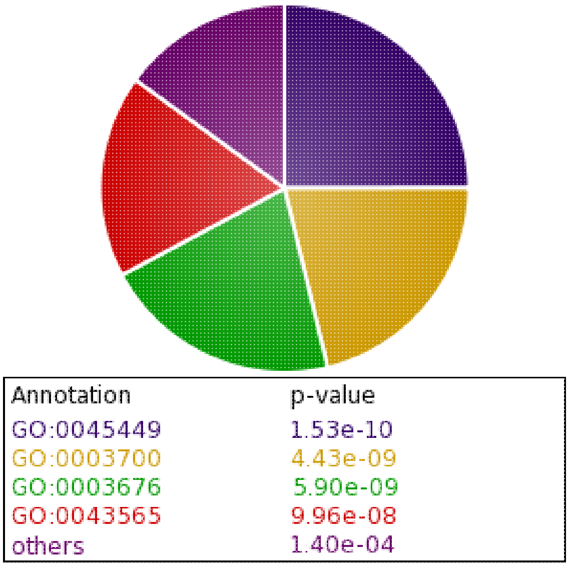

Annotation complete description

GO:0045449 => regulation of transcription GO:0003700 => transcription factor activity GO:0003676 => nucleic acid binding GO:0043565 => sequence-specific DNA binding GO:0006412 => translation GO:0003677 => DNA binding GO:0048046 => apoplast

Genome-wide Motif10 search results

Motif10 gene list of over-represented annotation(s)

Motif11

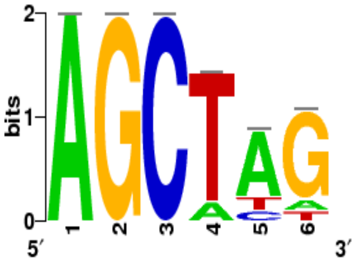

AGCTAG

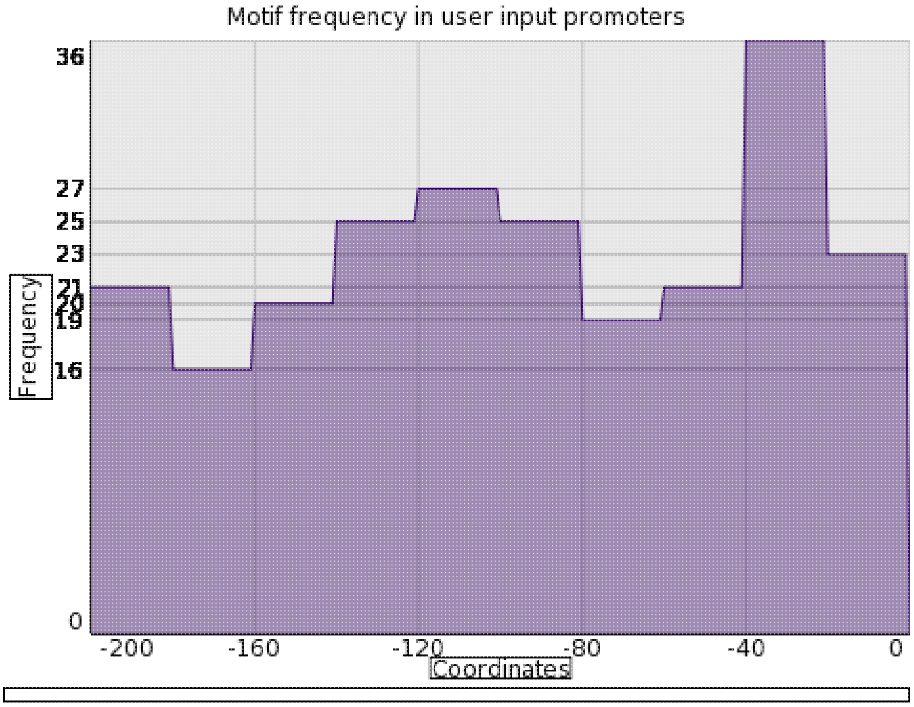

Motif11 annotation in the genome

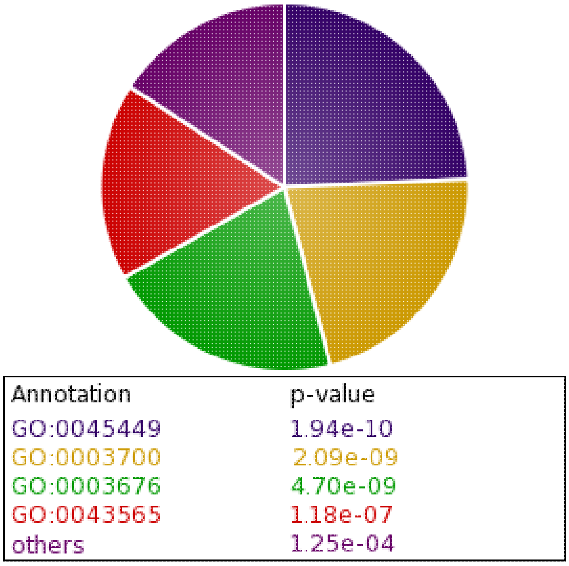

Annotation complete description

GO:0045449 => regulation of transcription GO:0003700 => transcription factor activity GO:0003676 => nucleic acid binding GO:0043565 => sequence-specific DNA binding GO:0006412 => translation GO:0003677 => DNA binding GO:0048046 => apoplast

Genome-wide Motif11 search results

Motif11 gene list of over-represented annotation(s)

Sequence logo generated by weblogo
Graphic generated with Chart::Clicker,Perl module
Promzea program from the Raizada lab
