# Supplementary material for: Promzea: a pipeline for discovery of co-regulatory motifs in maize and other plant species and its application to the anthocyanin and phlobaphene biosynthetic pathways and the Maize Development Atlas
Source: BMC Plant Biol. 2013 Mar 15;13:42. doi: 10.1186/1471-2229-13-42 (PMC3658923; doi:10.1186/1471-2229-13-42)
Supplement: Additional file 7 — Supplemental files for testing Promzea with data sets from the Maize Development Atlas. The zip folder contains 3 folders. The first contains the promoter input for Promzea for each maize tissue; the second folder has all the outputs from Promzea; the third folder contains the STAMP website outputs for comparisons of the predicted motifs with experimentally defined motifs. [file 1471-2229-13-42-S7.zip › Supplemental files 3 -Case study 3/2-Promzea results/silk.pdf]

Home

Results Summary

/vbox_shared/1-case_study_3/casestudy3_silk.txt

Promzea - 00000448

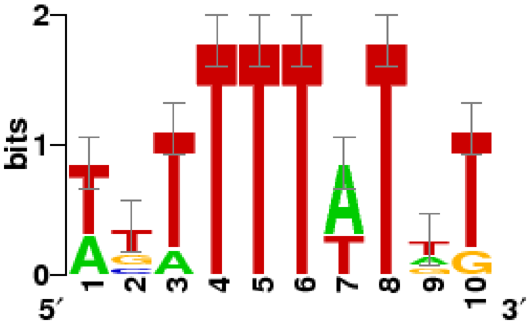

Motif1
(from BioP)
score: 6.76

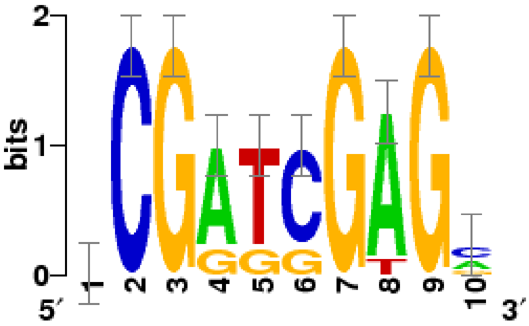

Motif2
(from BioP)
score: 5.6

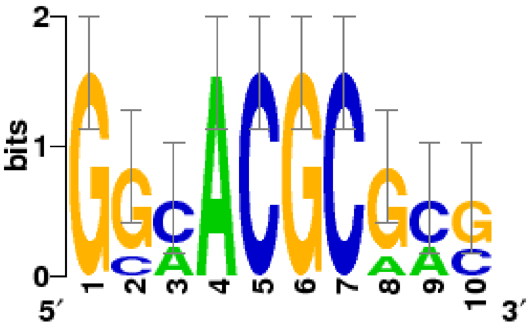

Motif3
(from MEME)
score: 5.26

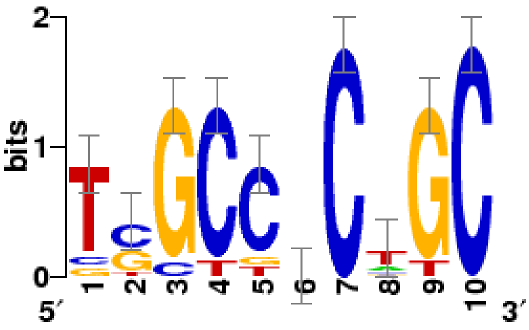

Motif4
(from MEME)
score: 3.9

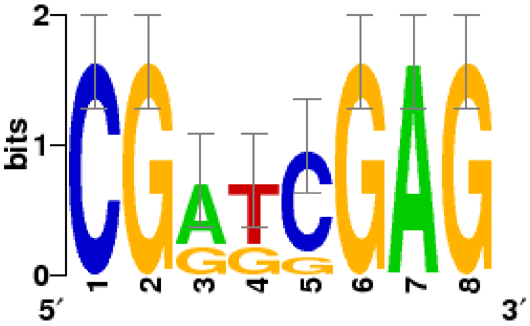

Motif5
(from MEME)
score: 3.36

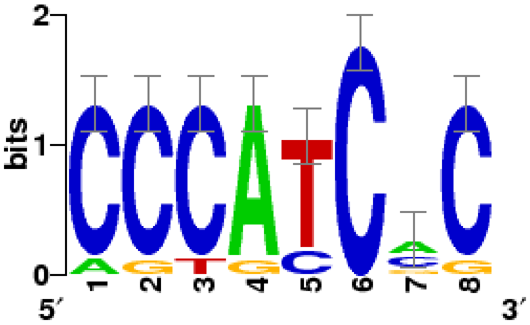

Motif6
(from MEME)
score: 2.78

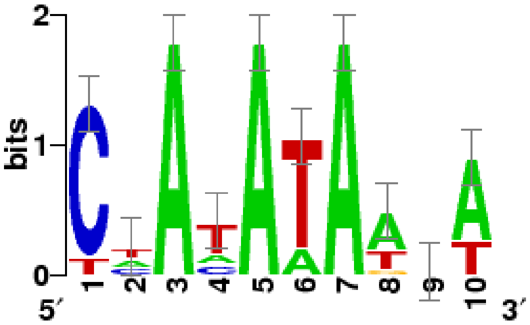

Motif7
(from BioP)
score: 2.72

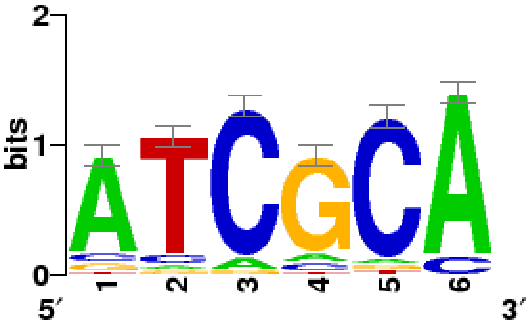

Motif8
(from Weeder)
score: 2.35

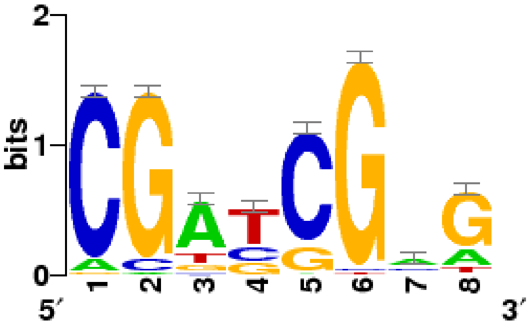

Motif9
(from Weeder)
score: 2.34

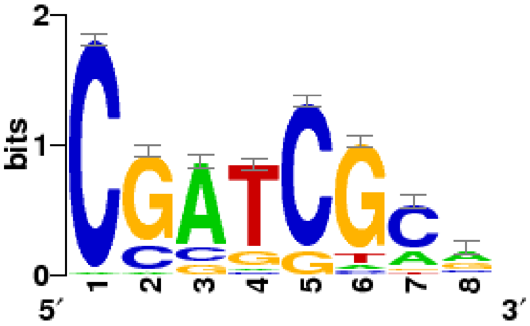

Motif10
(from Weeder)
score: 1.3

Compare your motifs to known promoter motif databases using STAMP website [motif file to copy in STAMP website]

Open the above link, copy content of the newly open file and paste in STAMP program link below In STAMP, under "Similarity Matching", we suggest selecting the plant motif databases: Athamap, AGRIS, PLACE, TRANSFAC; then submit

STAMP website

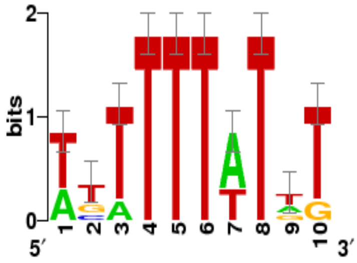

TTTTTTATTT

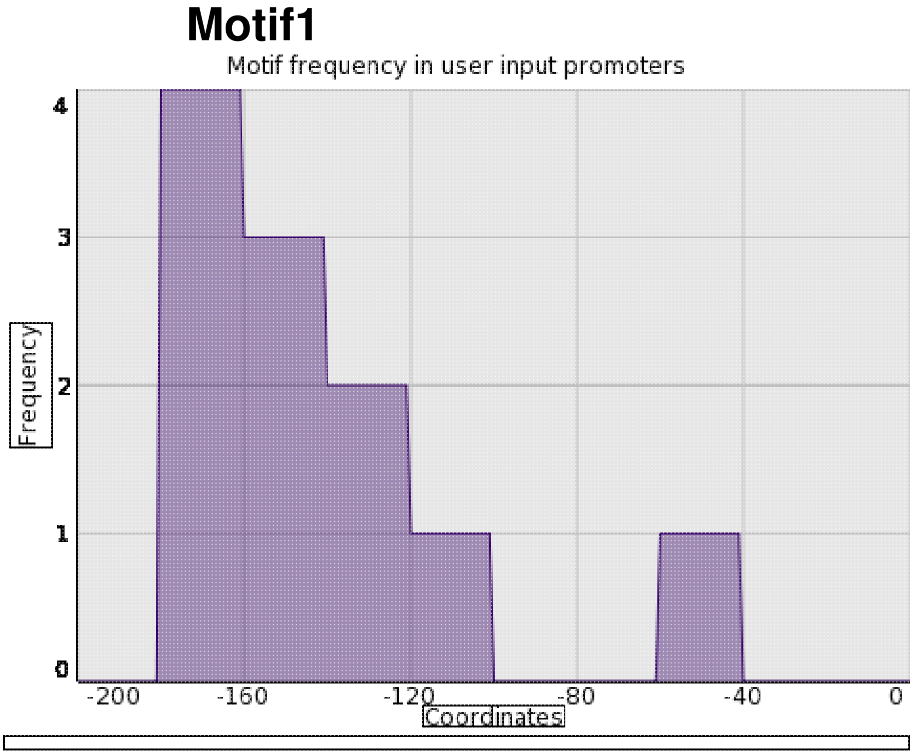

Motif1 annotation in the genome

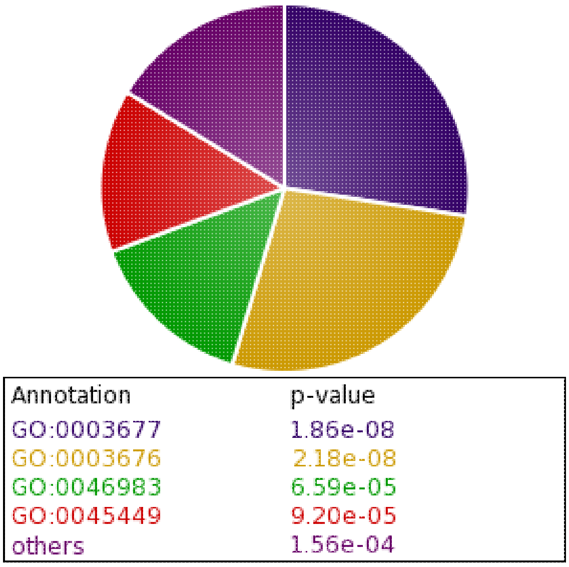

Annotation complete description

GO:0003677 => DNA binding GO:0003676 => nucleic acid binding GO:0046983 => protein dimerization activity GO:0045449 => regulation of transcription GO:0008270 => zinc ion binding GO:0005840 => ribosome GO:0003735 => structural constituent of ribosome

Genome-wide Motif1 search results

Motif1 gene list of over-represented annotation(s)

Motif2

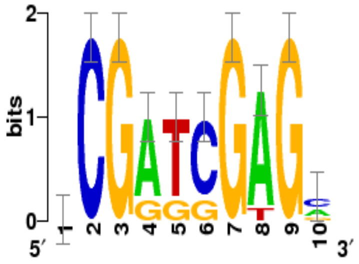

ACGATCGAGC

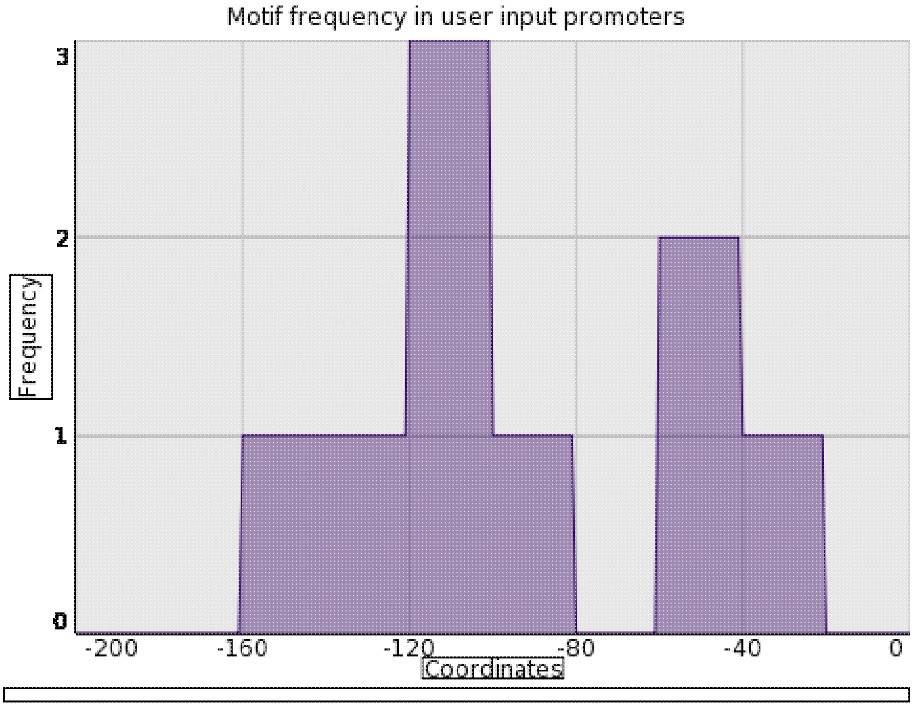

Motif2 annotation in the genome

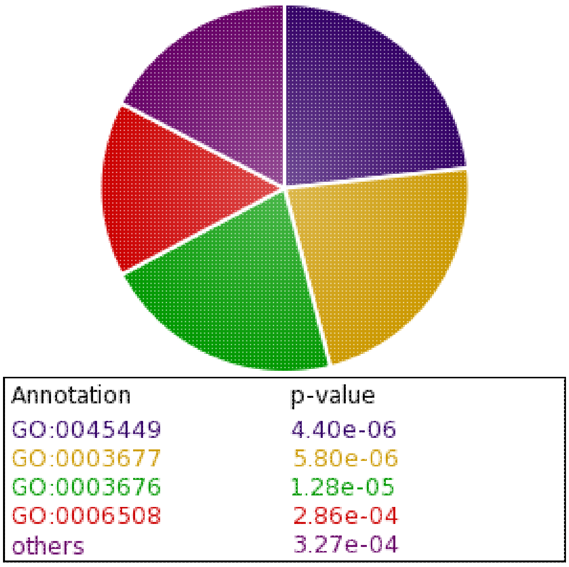

Annotation complete description

GO:0045449 => regulation of transcription GO:0003677 => DNA binding GO:0003676 => nucleic acid binding GO:0006508 => proteolysis GO:0005524 => ATP binding GO:0005985 => sucrose metabolic process GO:0050832 => defense response to fungus

Genome-wide Motif2 search results

Motif2 gene list of over-represented annotation(s)

Motif3

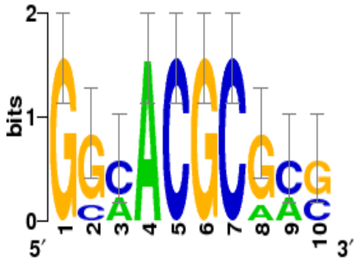

GGCACGCGCG

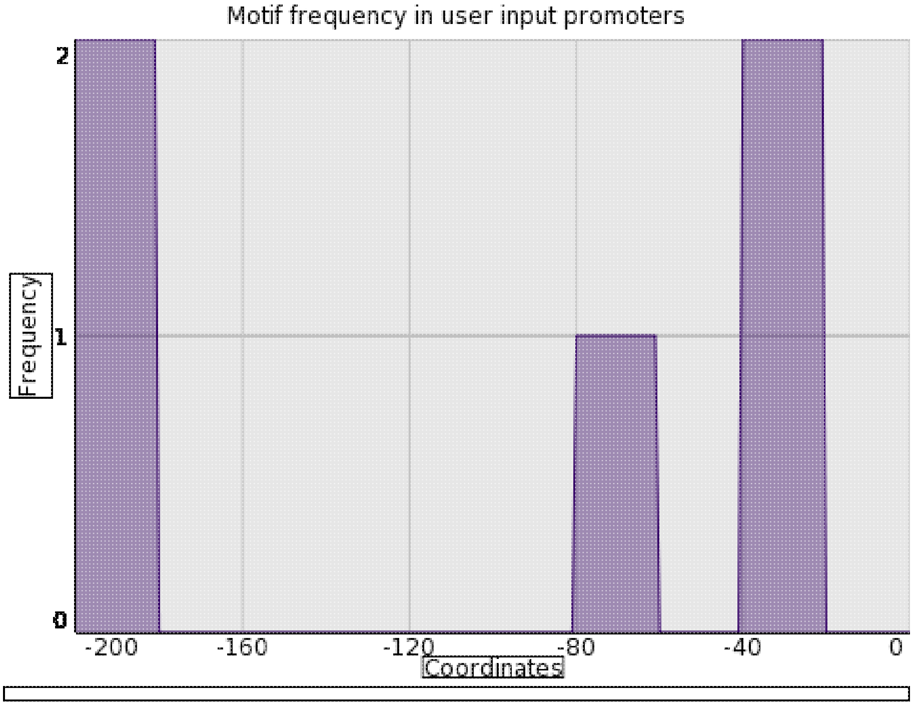

Motif3 annotation in the genome

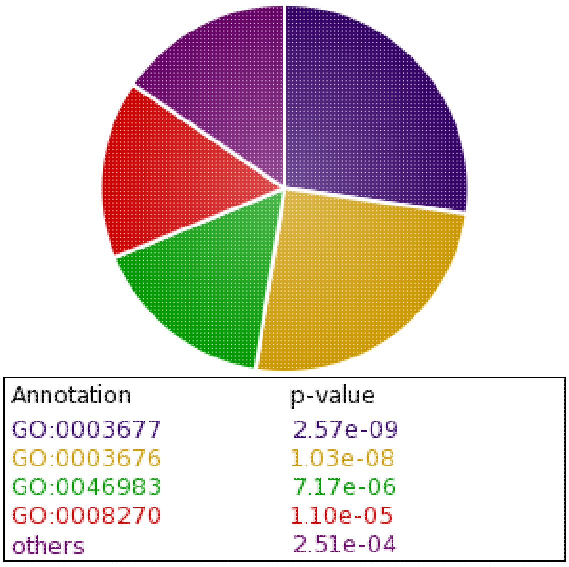

Annotation complete description

GO:0003677 => DNA binding GO:0003676 => nucleic acid binding GO:0046983 => protein dimerization activity GO:0008270 => zinc ion binding GO:0005840 => ribosome GO:0003735 => structural constituent of ribosome GO:0045449 => regulation of transcription

Genome-wide Motif3 search results

Motif3 gene list of over-represented annotation(s)

Motif4

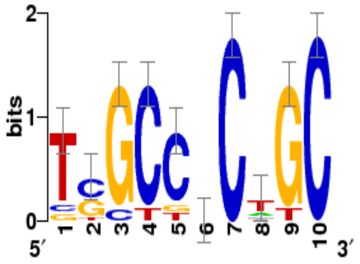

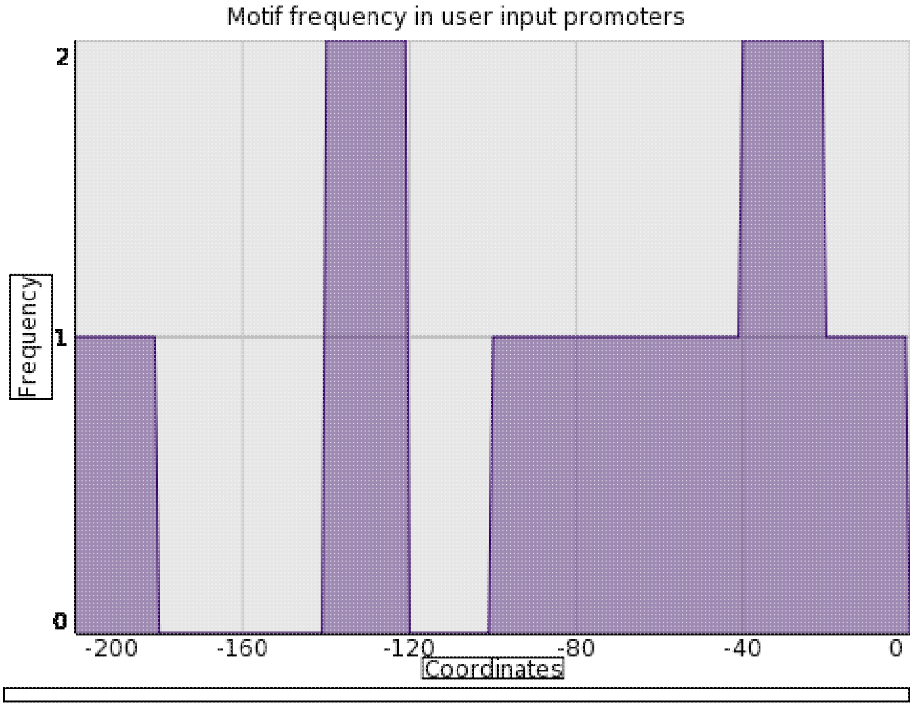

TCGCCMCTGC

Motif4 annotation in the genome

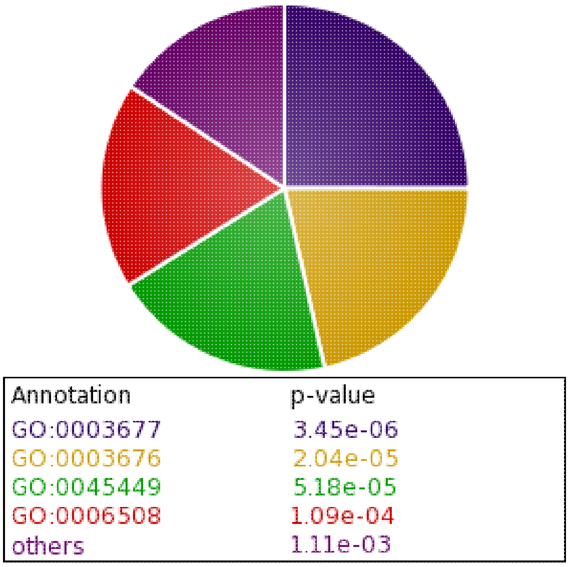

Annotation complete description

GO:0003677 => DNA binding GO:0003676 => nucleic acid binding GO:0045449 => regulation of transcription GO:0006508 => proteolysis GO:0005985 => sucrose metabolic process GO:0050832 => defense response to fungus GO:0046524 => sucrose-phosphate synthase activity

Genome-wide Motif4 search results

Motif4 gene list of over-represented annotation(s)

Motif5

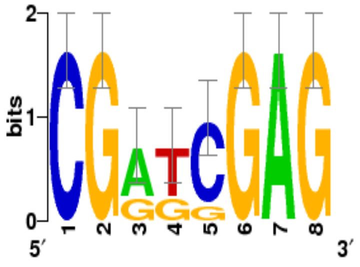

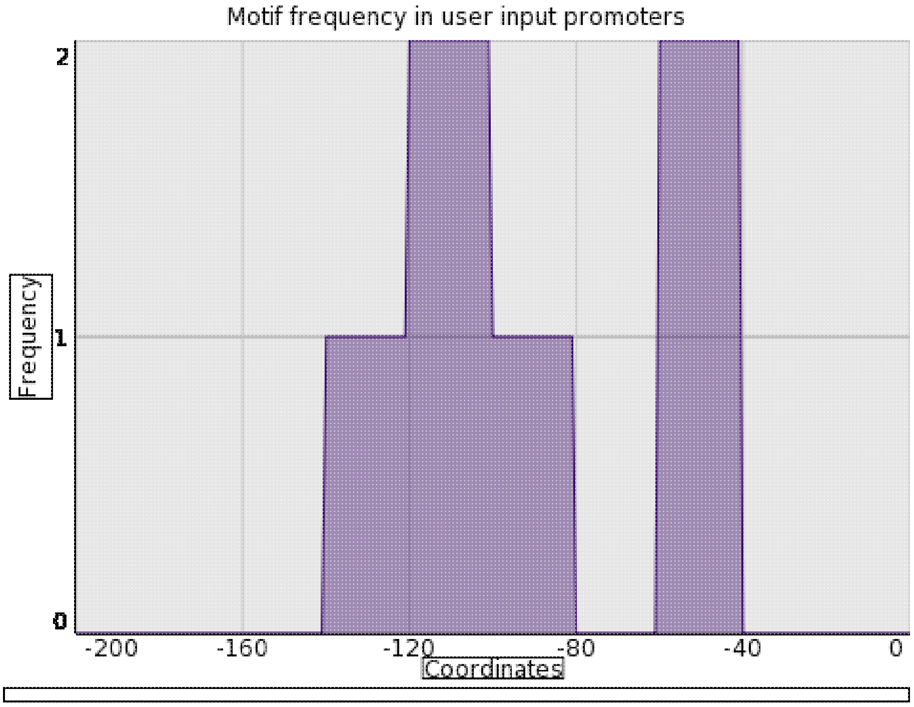

CGATCGAG

Motif5 annotation in the genome

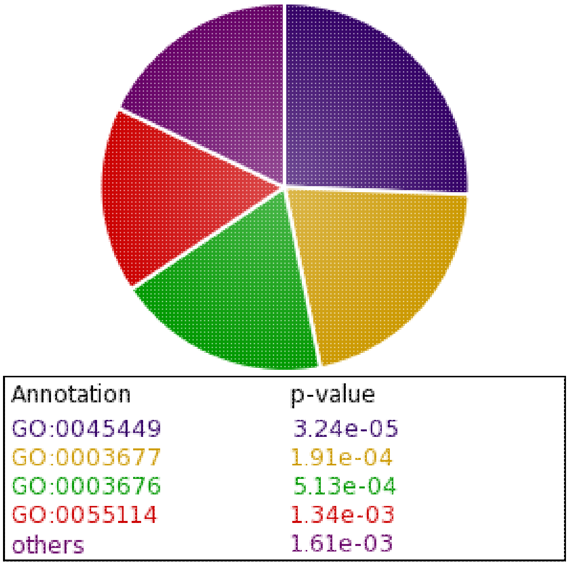

Annotation complete description

GO:0045449 => regulation of transcription GO:0003677 => DNA binding GO:0003676 => nucleic acid binding GO:0055114 => oxidation reduction GO:0005506 => iron ion binding GO:0005524 => ATP binding GO:0020037 => heme binding

Genome-wide Motif5 search results

Motif5 gene list of over-represented annotation(s)

Motif6

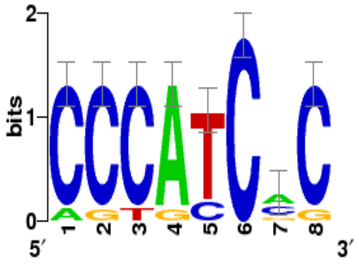

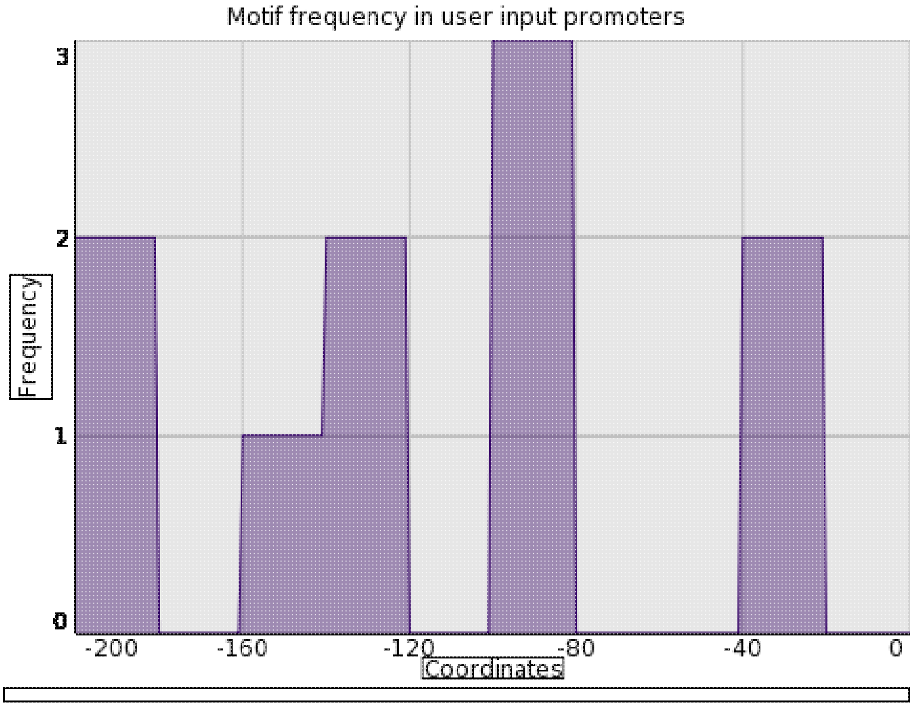

CCCATCMC

Motif6 annotation in the genome

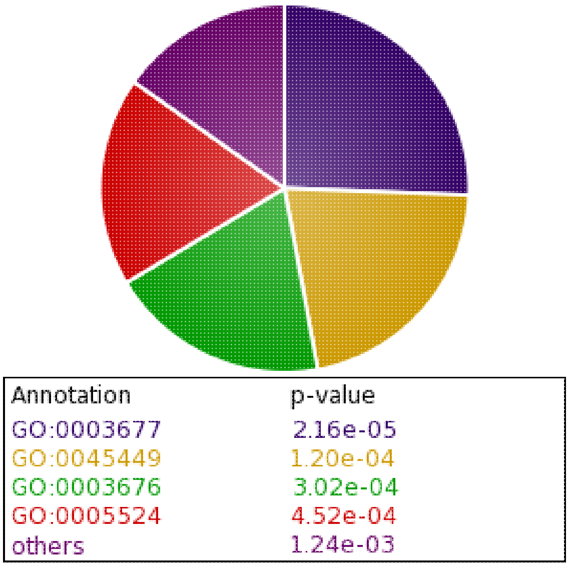

Annotation complete description

GO:0003677 => DNA binding GO:0045449 => regulation of transcription GO:0003676 => nucleic acid binding GO:0005524 => ATP binding GO:0005506 => iron ion binding GO:0055114 => oxidation reduction GO:0008270 => zinc ion binding

Genome-wide Motif6 search results

Motif6 gene list of over-represented annotation(s)

Motif7

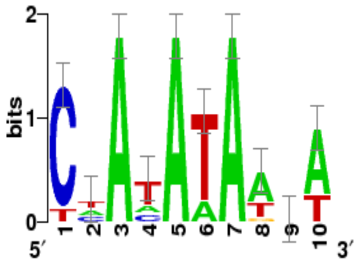

CTATATAAAA

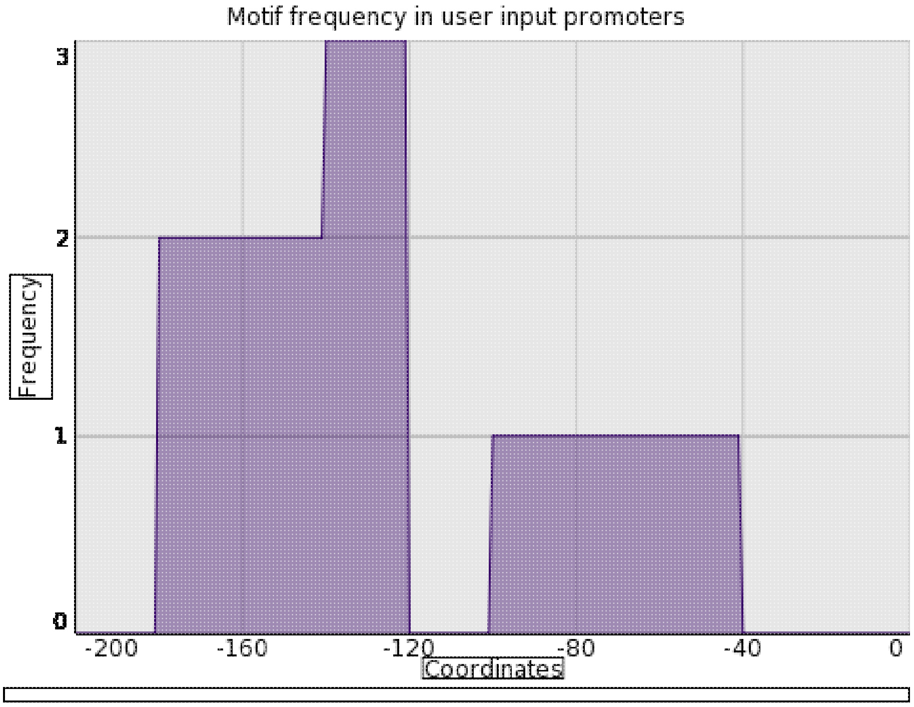

Motif7 annotation in the genome

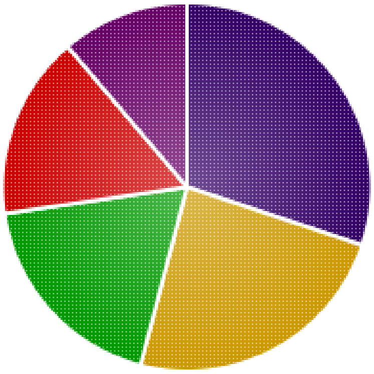

Annotation	p-value
GO:0045449	1.58e-05
GO:0005506	1.52e-04
GO:0009055	1.07e-03
GO:0005488	2.70e-03
others	3.01e-03

Annotation complete description

GO:0045449 => regulation of transcription GO:0005506 => iron ion binding GO:0009055 => electron carrier activity GO:0005488 => binding GO:0046524 => sucrose-phosphate synthase activity GO:0020037 => heme binding GO:0006508 => proteolysis

Genome-wide Motif7 search results

Motif7 gene list of over-represented annotation(s)

Motif8

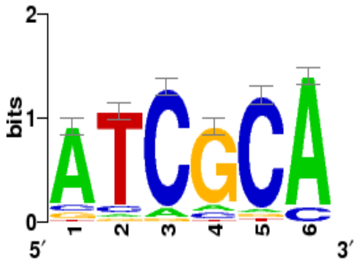

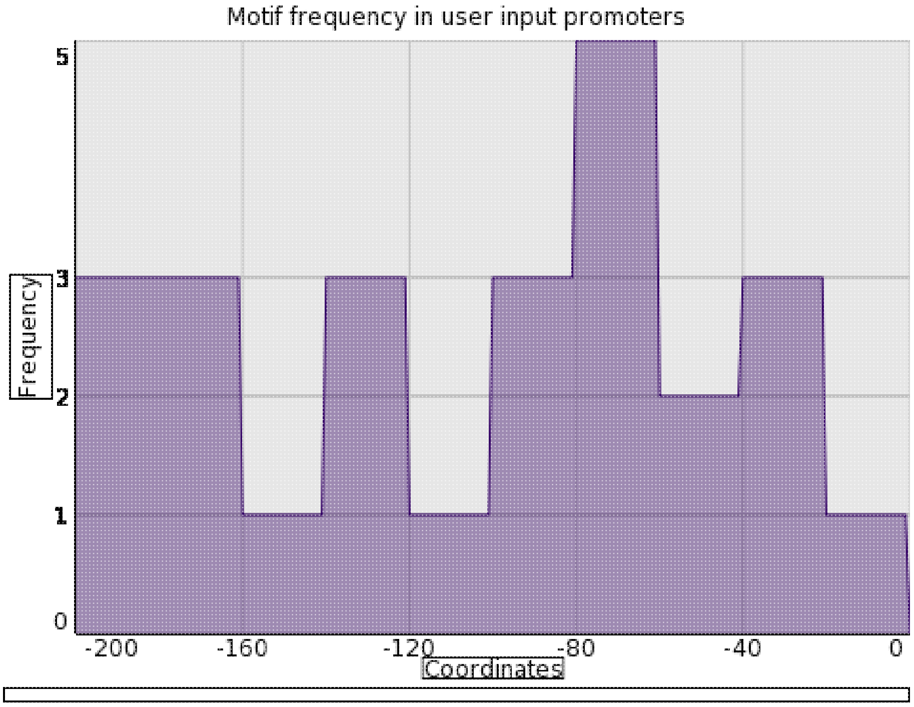

ATCGCA

Motif8 annotation in the genome

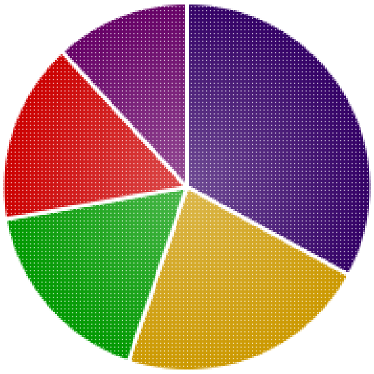

Annotation	p-value
GO:0045449	8.42e-06
GO:0005506	3.79e-04
GO:0009055	2.36e-03
GO:0046524	3.50e-03
others	5.05e-03

Annotation complete description

GO:0045449 => regulation of transcription GO:0005506 => iron ion binding GO:0009055 => electron carrier activity GO:0046524 => sucrose-phosphate synthase activity GO:0005488 => binding GO:0007050 => cell cycle arrest GO:0004861 => cyclin-dependent protein kinase inhibitor activity

Genome-wide Motif8 search results

Motif8 gene list of over-represented annotation(s)

Motif9

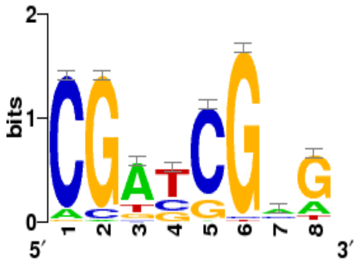

CGATCGAG

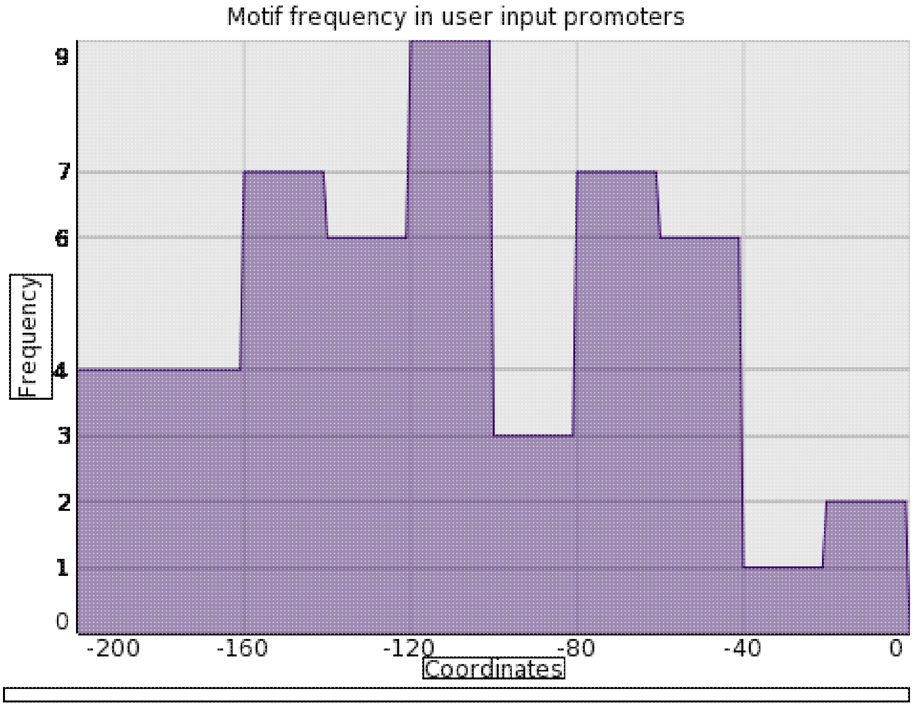

Motif9 annotation in the genome

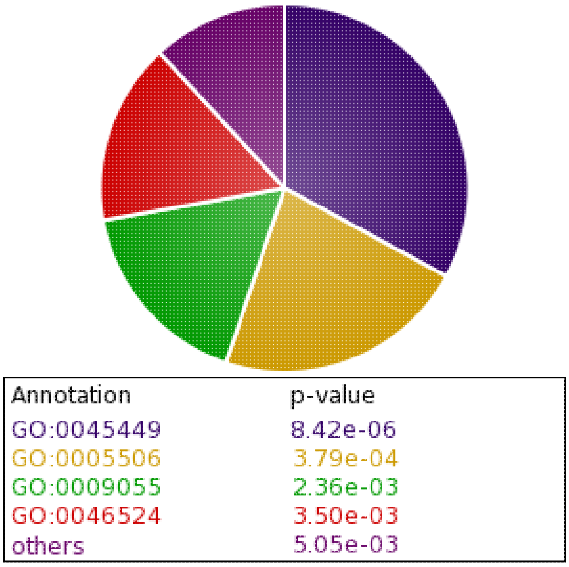

Annotation complete description

GO:0045449 => regulation of transcription GO:0005506 => iron ion binding GO:0009055 => electron carrier activity GO:0046524 => sucrose-phosphate synthase activity GO:0005488 => binding GO:0007050 => cell cycle arrest GO:0004861 => cyclin-dependent protein kinase inhibitor activity

Genome-wide Motif9 search results

Motif9 gene list of over-represented annotation(s)

Motif10

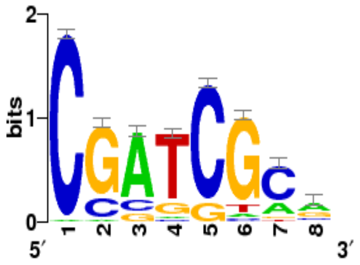

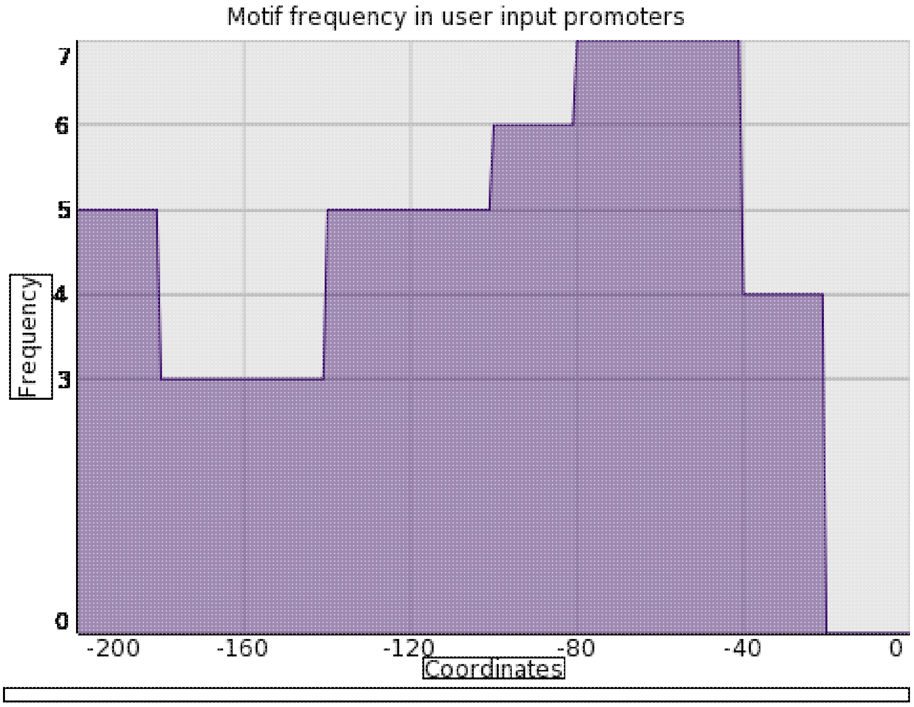

CGATCGCA

Motif10 annotation in the genome

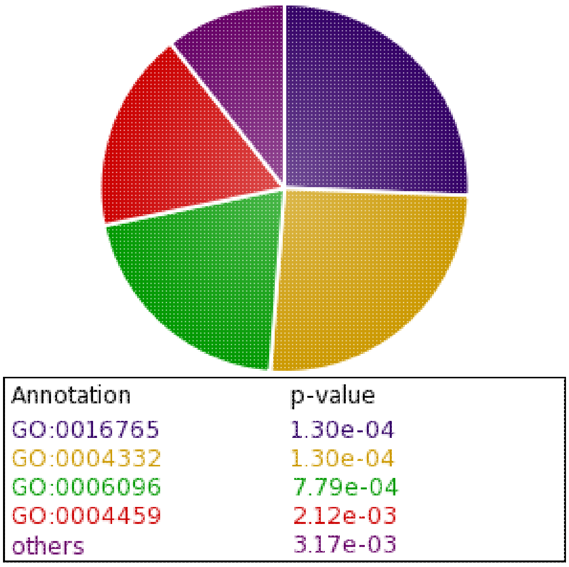

Annotation complete description

GO:0016765 => transferase activity, transferring alkyl or aryl (other than methyl) groups GO:0004332 => fructose-bisphosphate aldolase activity GO:0006096 => glycolysis GO:0004459 => L-lactate dehydrogenase activity GO:0004097 => catechol oxidase activity GO:0006302 => double-strand break repair GO:0030259 => lipid glycosylation

Genome-wide Motif10 search results

Motif10 gene list of over-represented annotation(s)

Sequence logo generated by weblogo
Graphic generated with Chart::Clicker,Perl module
Promzea program from the Raizada lab
