# Supplementary material for: Promzea: a pipeline for discovery of co-regulatory motifs in maize and other plant species and its application to the anthocyanin and phlobaphene biosynthetic pathways and the Maize Development Atlas
Source: BMC Plant Biol. 2013 Mar 15;13:42. doi: 10.1186/1471-2229-13-42 (PMC3658923; doi:10.1186/1471-2229-13-42)
Supplement: Additional file 7 — Supplemental files for testing Promzea with data sets from the Maize Development Atlas. The zip folder contains 3 folders. The first contains the promoter input for Promzea for each maize tissue; the second folder has all the outputs from Promzea; the third folder contains the STAMP website outputs for comparisons of the predicted motifs with experimentally defined motifs. [file 1471-2229-13-42-S7.zip › Supplemental files 3 -Case study 3/2-Promzea results/tassel.pdf]

Results Summary

/vbox_shared/1-case_study_3/casestudy3_tassel.txt

Promzea - 00000449

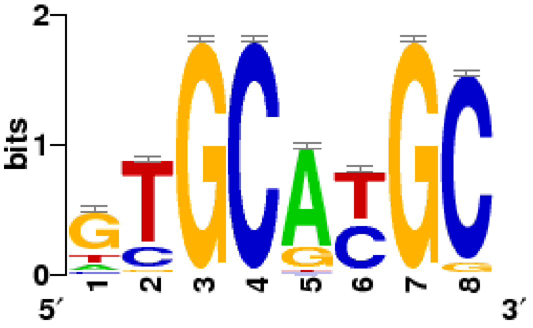

Motif1
(from Weeder)
score: 5.98

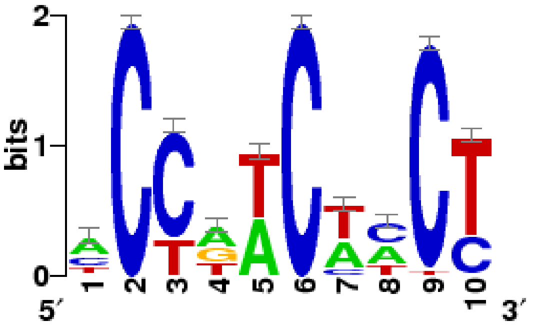

Motif2
(from BioP)
score: 5.44

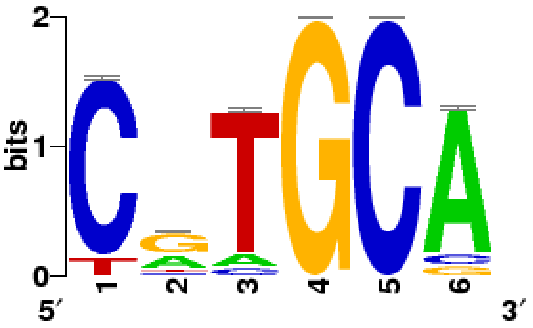

Motif3
(from Weeder)
score: 5.39

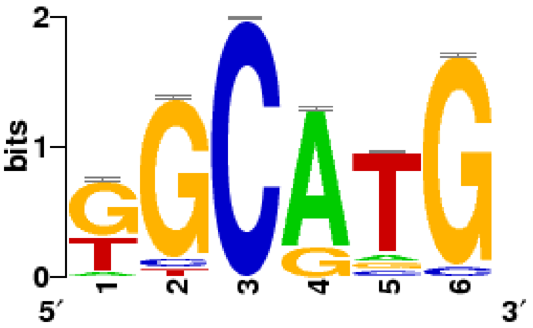

Motif4
(from Weeder)
score: 5.39

results - 00000449

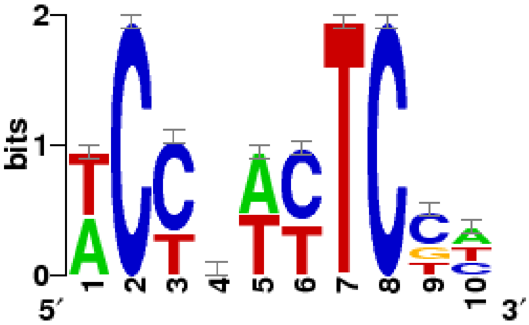

Motif5
(from BioP)
score: 5.32

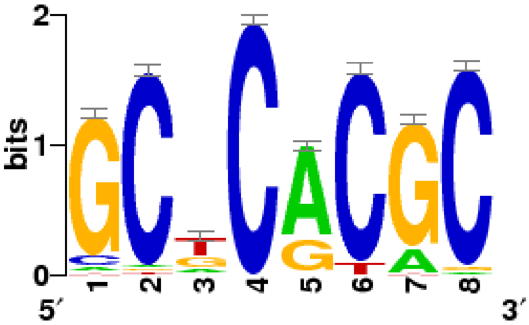

Motif6
(from Weeder)
score: 4.98

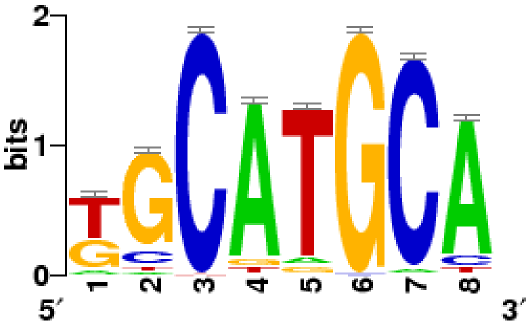

Motif7
(from Weeder)
score: 3.98

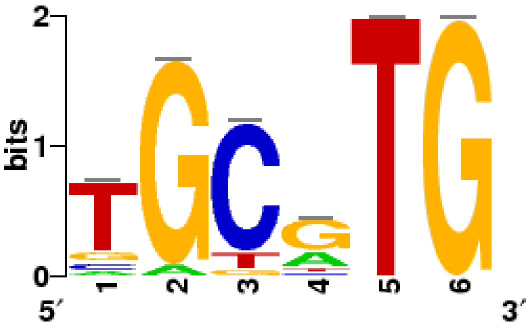

Motif8
(from Weeder)
score: 3.44

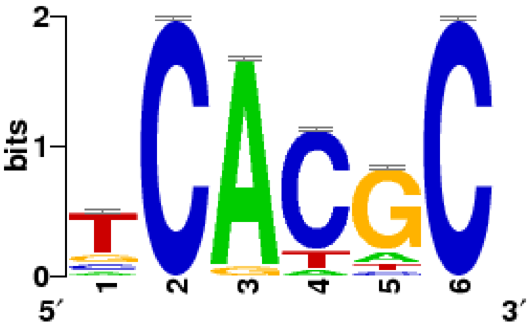

Motif9
(from Weeder)
score: 3.44

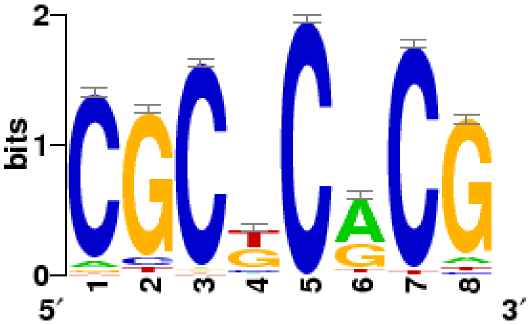

Motif10
(from Weeder)
score: 1.74

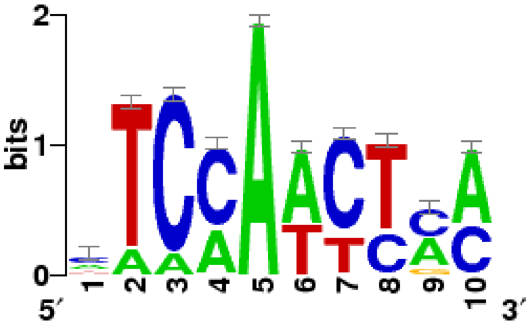

Motif11
(from BioP)
score: 1.68

Compare your motifs to known promoter motif databases using STAMP website motif file to copy in STAMP website

Open the above link, copy content of the newly open file and paste in STAMP program link below In STAMP, under "Similarity Matching", we suggest selecting the plant motif databases: Athamap, AGRIS, PLACE, TRANSFAC; then submit

STAMP website

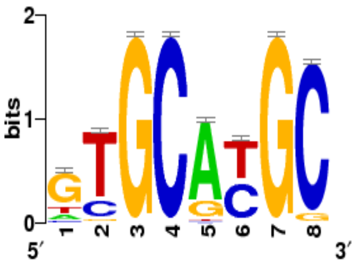

GTGCATGC

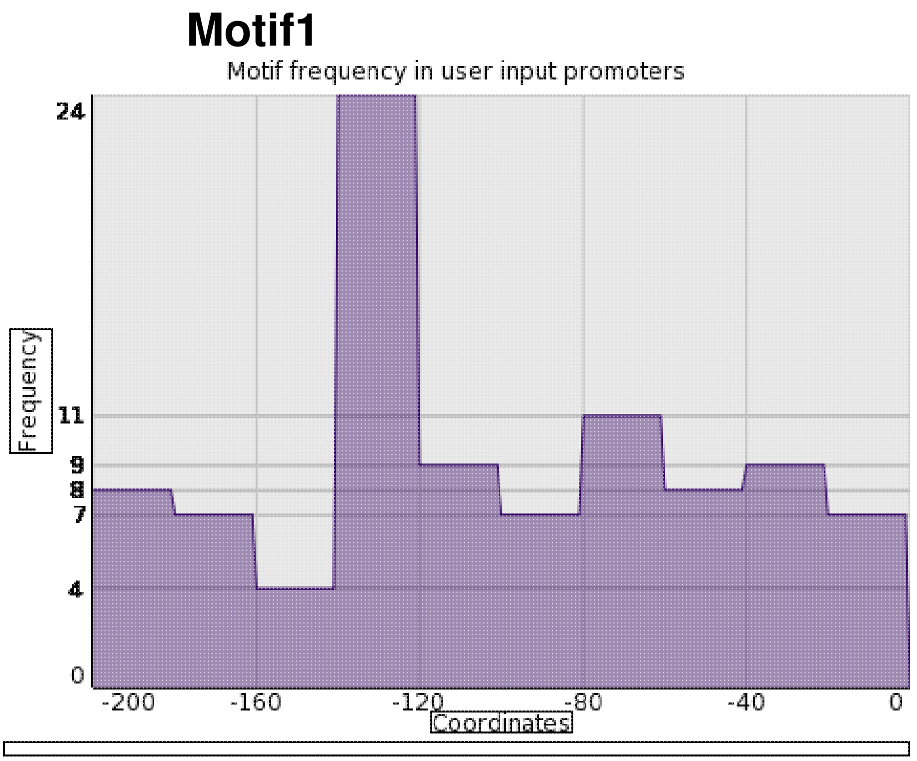

Motif1 annotation in the genome

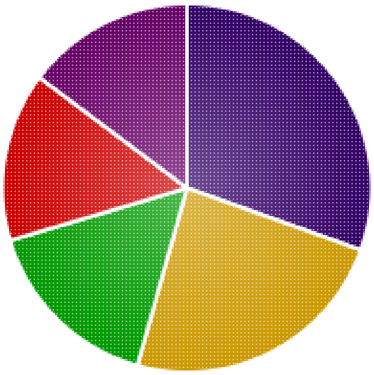

Annotation	p-value
GO:0003676	5.41e-13
GO:0019953	2.42e-10
GO:0003677	3.37e-07
GO:0004857	1.06e-06
others	5.71e-06

Annotation complete description

GO:0003676 => nucleic acid binding GO:0019953 => sexual reproduction GO:0003677 => DNA binding GO:0004857 => enzyme inhibitor activity GO:0008270 => zinc ion binding GO:0005576 => extracellular region GO:0005488 => binding

Genome-wide Motif1 search results

Motif1 gene list of over-represented annotation(s)

Motif2

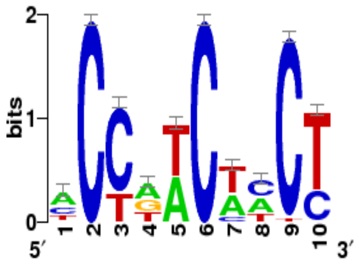

ACCATCTCCT

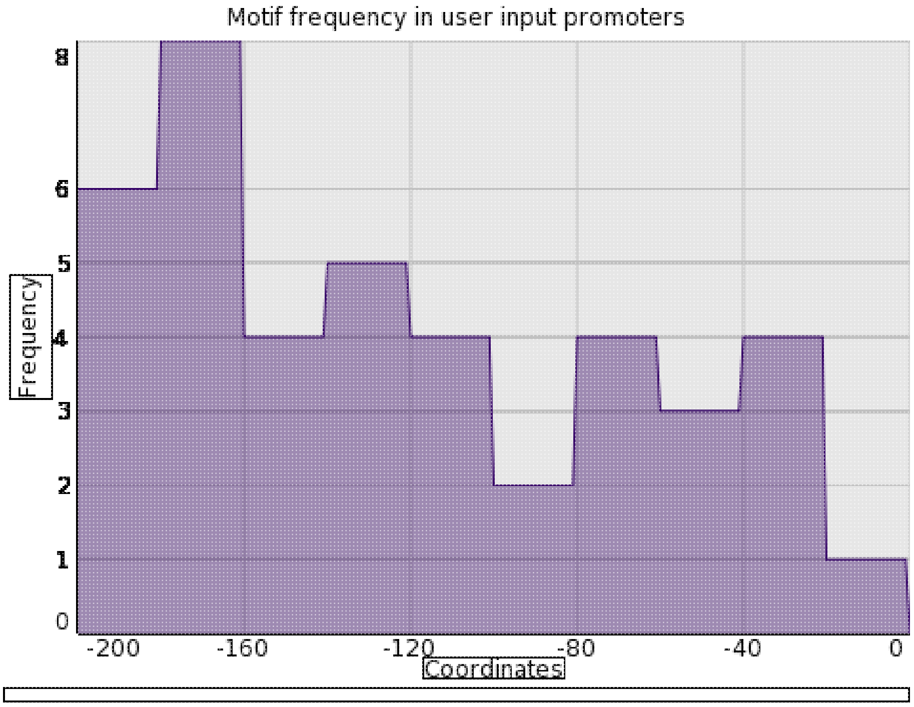

Motif2 annotation in the genome

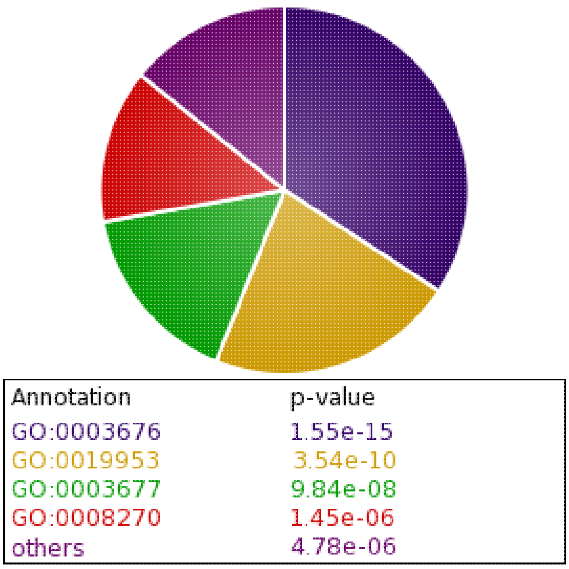

Annotation complete description

GO:0003676 => nucleic acid binding GO:0019953 => sexual reproduction GO:0003677 => DNA binding GO:0008270 => zinc ion binding GO:0004857 => enzyme inhibitor activity GO:0046983 => protein dimerization activity GO:0005576 => extracellular region

Genome-wide Motif2 search results

Motif2 gene list of over-represented annotation(s)

Motif3

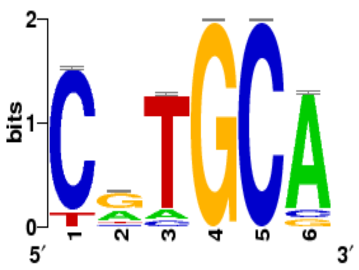

CGTGCA

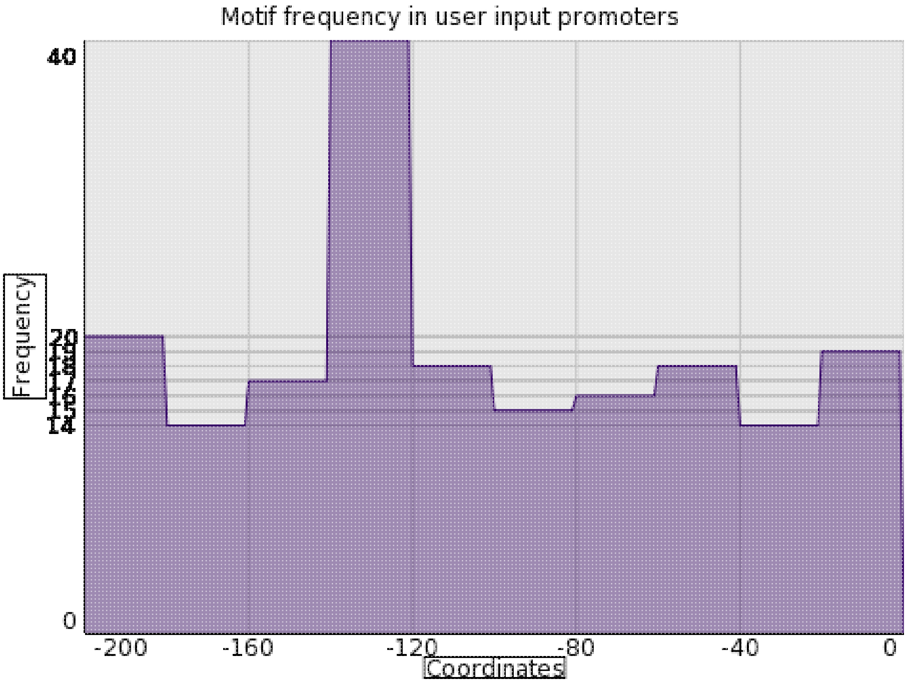

Motif3 annotation in the genome

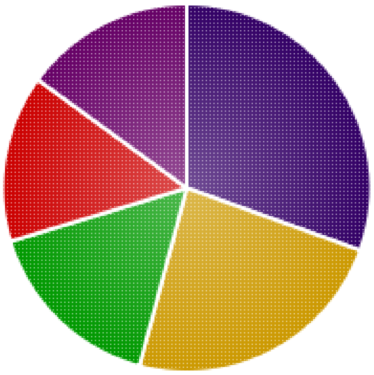

Annotation	p-value
GO:0003676	4.38e-13
GO:0019953	2.57e-10
GO:0003677	2.76e-07
GO:0004857	1.11e-06
others	4.82e-06

Annotation complete description

GO:0003676 => nucleic acid binding GO:0019953 => sexual reproduction GO:0003677 => DNA binding GO:0004857 => enzyme inhibitor activity GO:0008270 => zinc ion binding GO:0005576 => extracellular region GO:0005488 => binding

Genome-wide Motif3 search results

Motif3 gene list of over-represented annotation(s)

Motif4

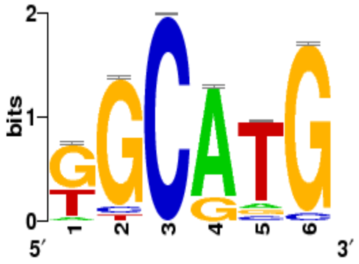

GGCATG

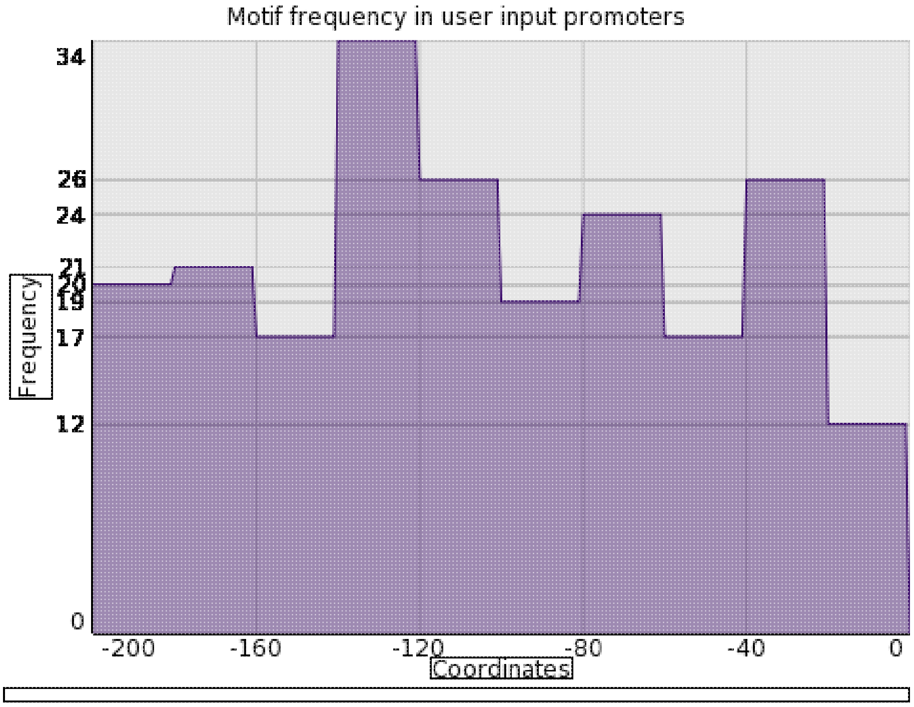

Motif4 annotation in the genome

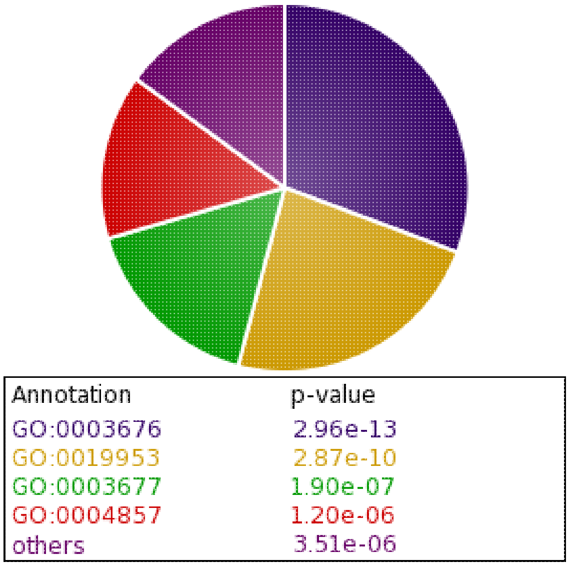

Annotation complete description

GO:0003676 => nucleic acid binding GO:0019953 => sexual reproduction GO:0003677 => DNA binding GO:0004857 => enzyme inhibitor activity GO:0008270 => zinc ion binding GO:0005488 => binding GO:0005576 => extracellular region

Genome-wide Motif4 search results

Motif4 gene list of over-represented annotation(s)

Motif5

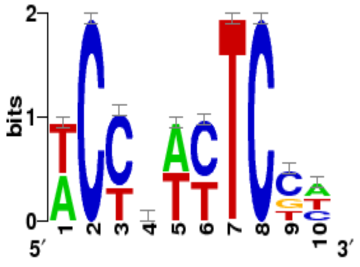

TCCAWCTCCA

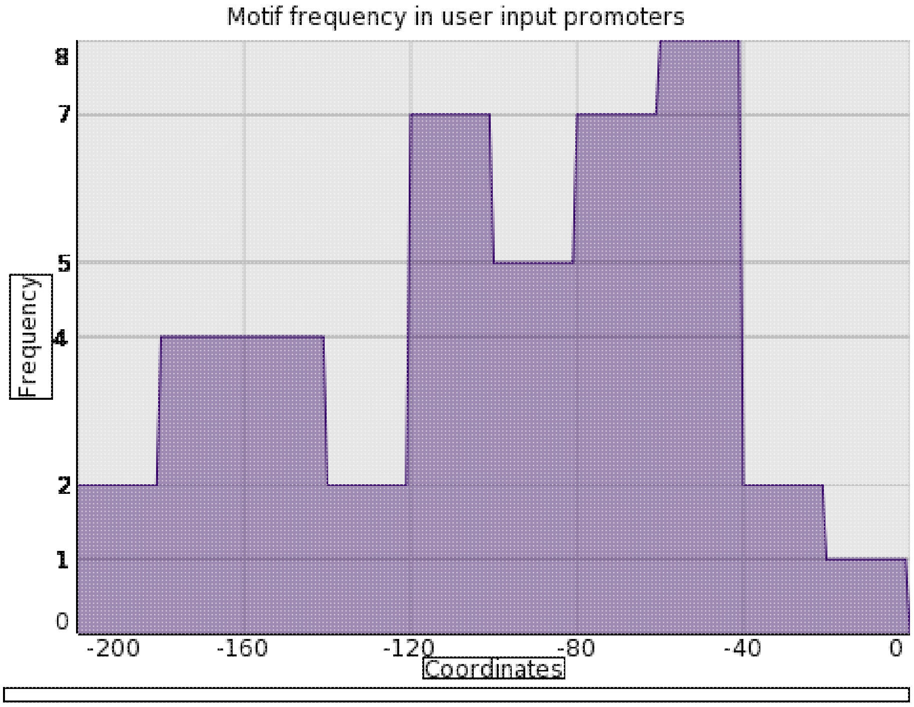

Motif5 annotation in the genome

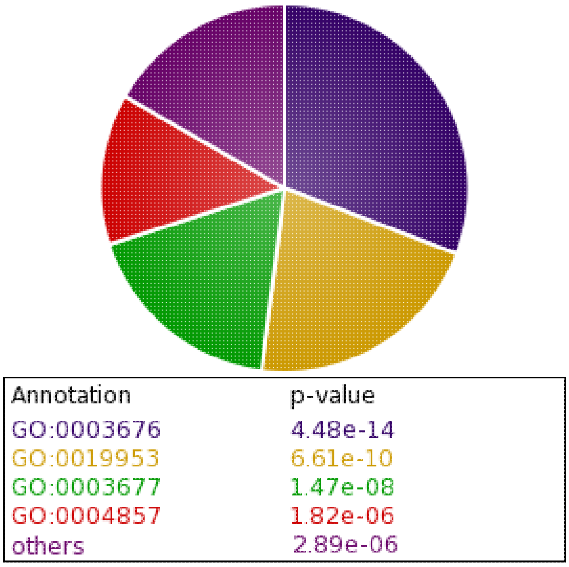

Annotation complete description

GO:0003676 => nucleic acid binding GO:0019953 => sexual reproduction GO:0003677 => DNA binding GO:0004857 => enzyme inhibitor activity GO:0005488 => binding GO:0008270 => zinc ion binding GO:0046983 => protein dimerization activity

Genome-wide Motif5 search results

Motif5 gene list of over-represented annotation(s)

Motif6

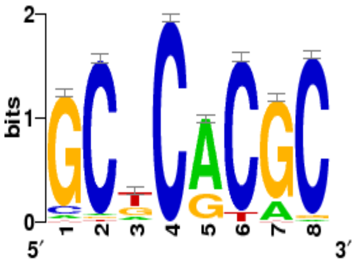

GCTCACGC

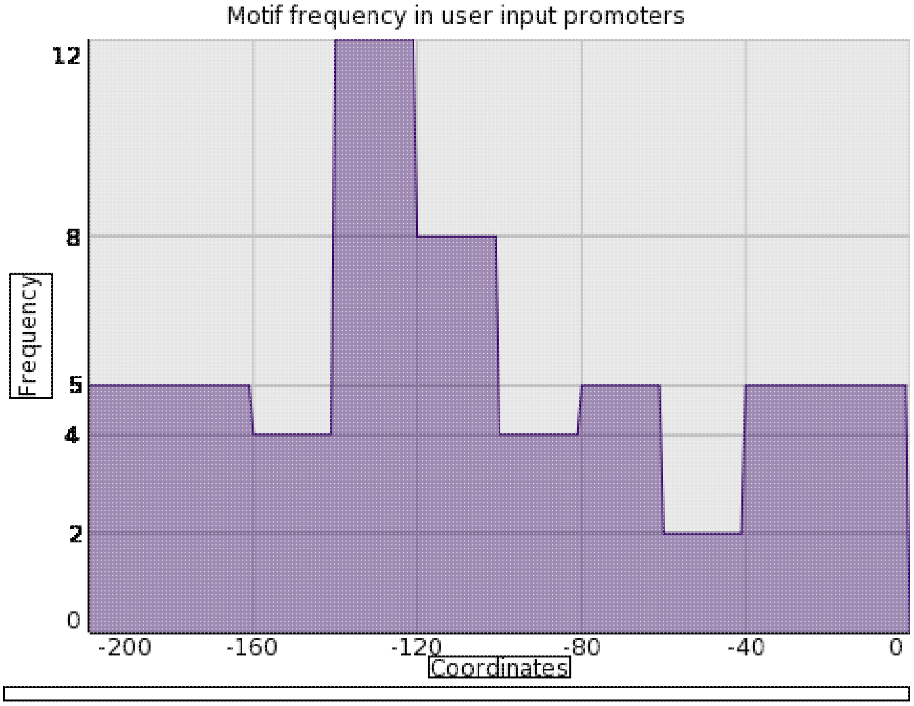

Motif6 annotation in the genome

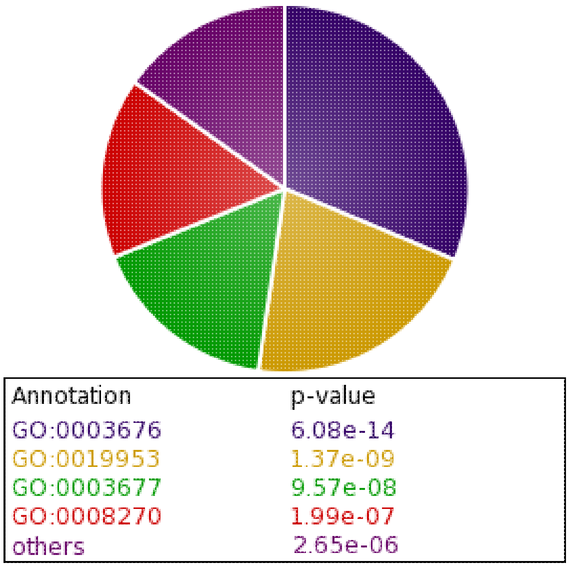

Annotation complete description

GO:0003676 => nucleic acid binding GO:0019953 => sexual reproduction GO:0003677 => DNA binding GO:0008270 => zinc ion binding GO:0004857 => enzyme inhibitor activity GO:0046983 => protein dimerization activity GO:0005576 => extracellular region

Genome-wide Motif6 search results

Motif6 gene list of over-represented annotation(s)

Motif7

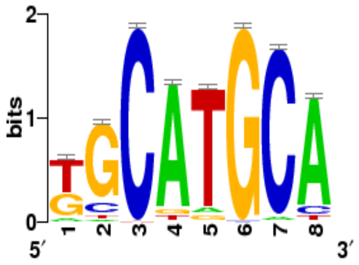

TGCATGCA

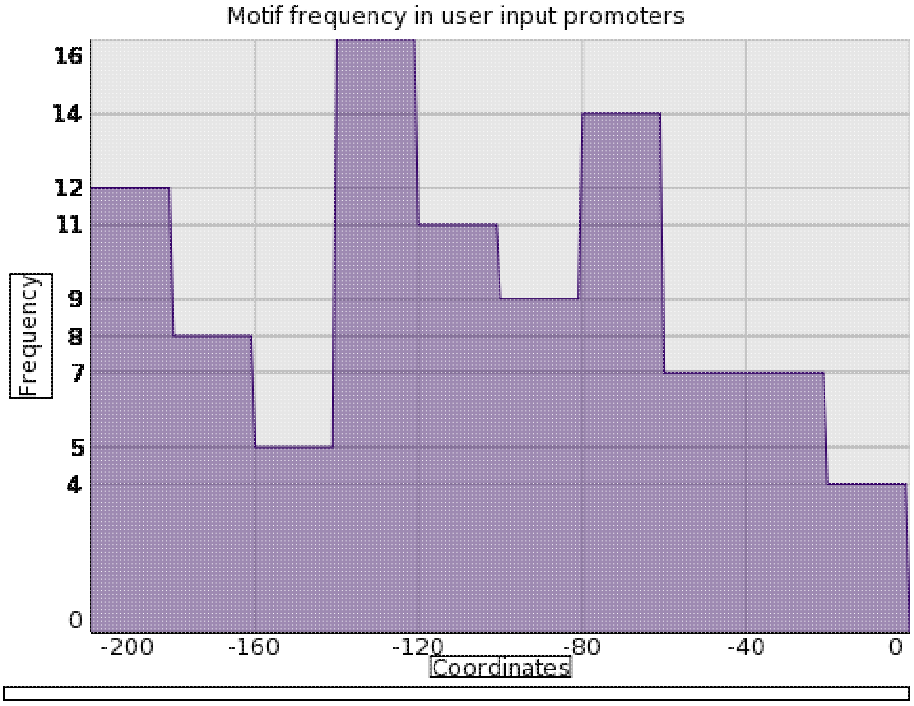

Motif7 annotation in the genome

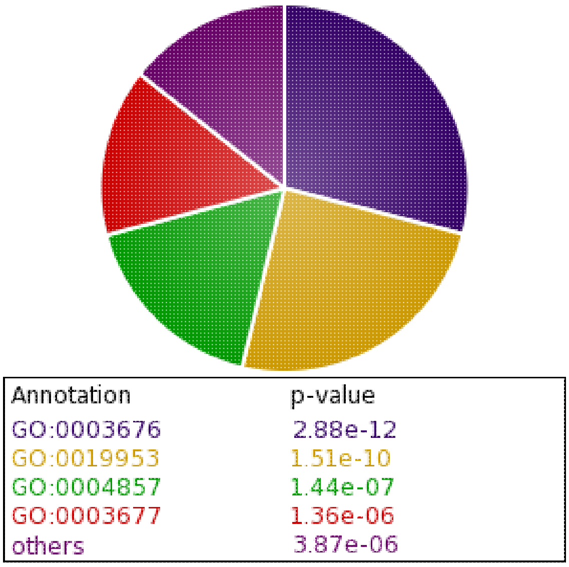

Annotation complete description

GO:0003676 => nucleic acid binding GO:0019953 => sexual reproduction GO:0004857 => enzyme inhibitor activity GO:0003677 => DNA binding GO:0005488 => binding GO:0005576 => extracellular region GO:0008270 => zinc ion binding

Genome-wide Motif7 search results

Motif7 gene list of over-represented annotation(s)

Motif8

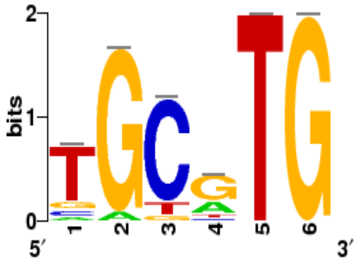

TGCGTG

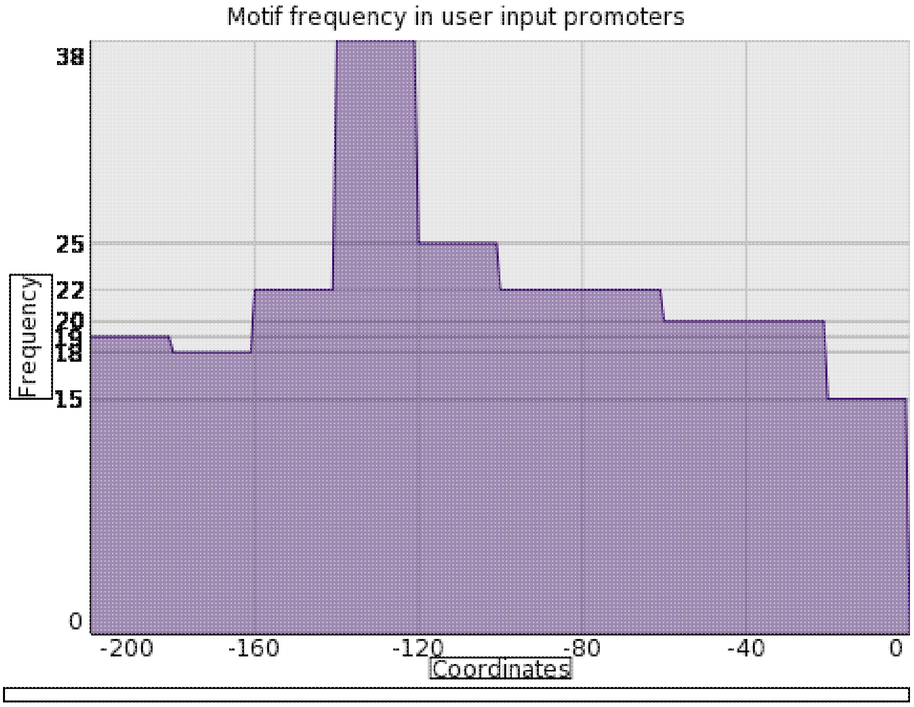

Motif8 annotation in the genome

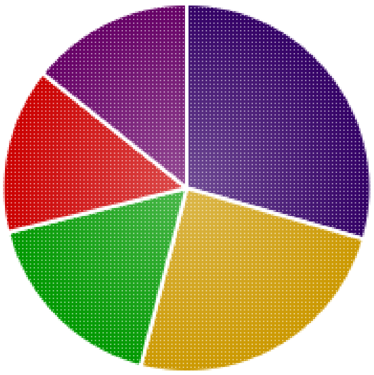

Annotation	p-value
GO:0003676	1.92e-12
GO:0019953	1.71e-10
GO:0004857	1.59e-07
GO:0003677	1.61e-06
others	8.00e-06

Annotation complete description

GO:0003676 => nucleic acid binding GO:0019953 => sexual reproduction GO:0004857 => enzyme inhibitor activity GO:0003677 => DNA binding GO:0005576 => extracellular region GO:0008270 => zinc ion binding GO:0005488 => binding

Genome-wide Motif8 search results

Motif8 gene list of over-represented annotation(s)

Motif9

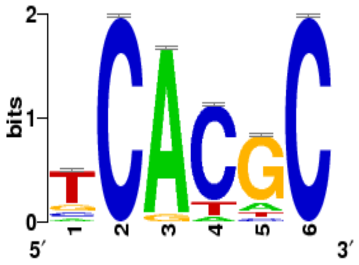

TCACGC

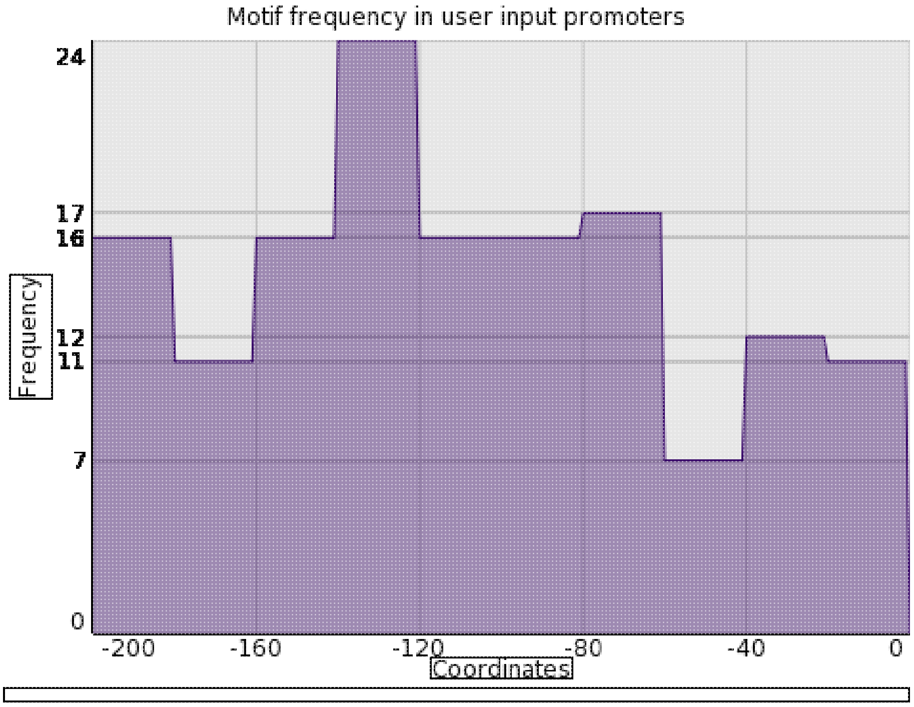

Motif9 annotation in the genome

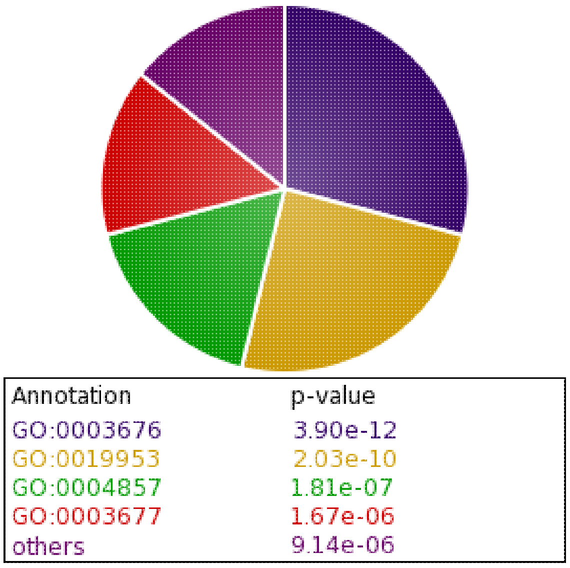

Annotation complete description

GO:0003676 => nucleic acid binding GO:0019953 => sexual reproduction GO:0004857 => enzyme inhibitor activity GO:0003677 => DNA binding GO:0005488 => binding GO:0005576 => extracellular region GO:0008270 => zinc ion binding

Genome-wide Motif9 search results

Motif9 gene list of over-represented annotation(s)

Motif10

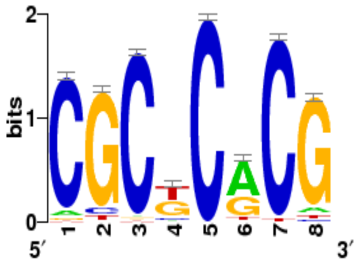

CGCTCACG

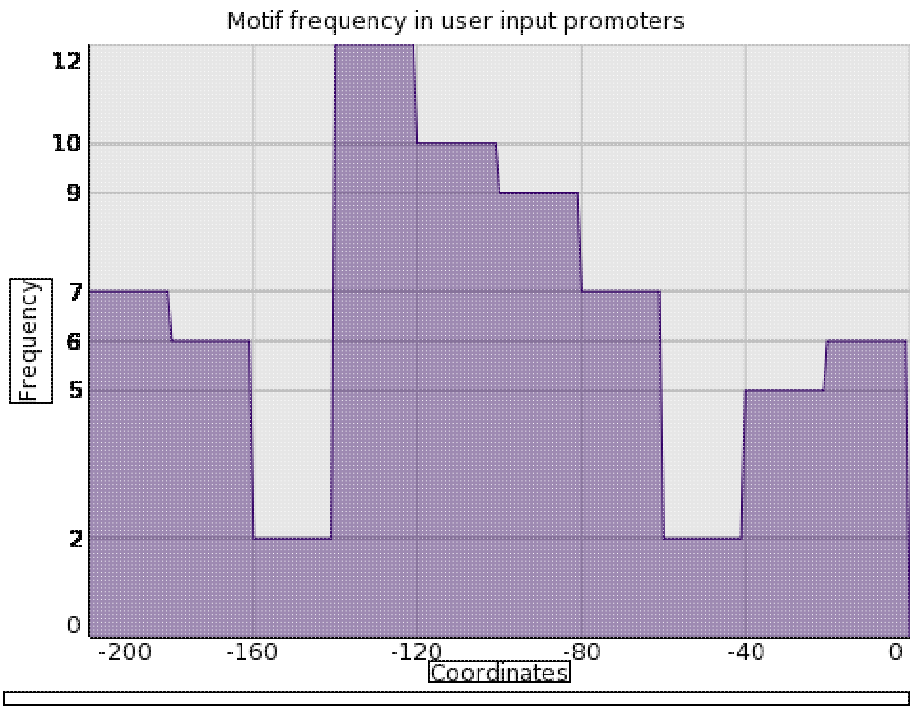

Motif10 annotation in the genome

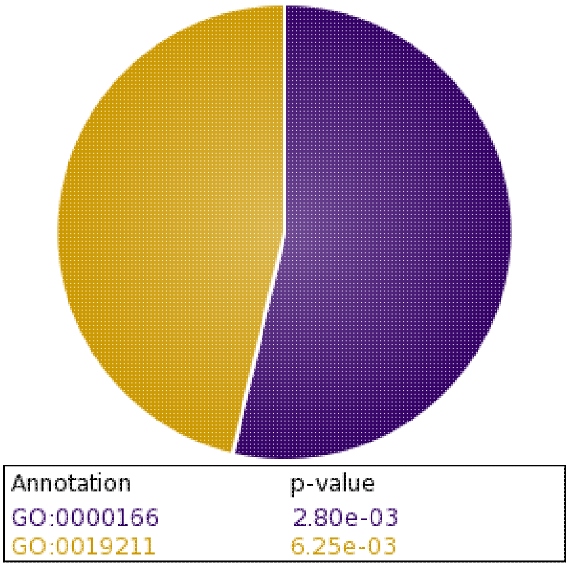

Annotation complete description

GO:0000166 => nucleotide binding GO:0019211 => phosphatase activator activity

Genome-wide Motif10 search results

Motif10 gene list of over-represented annotation(s)

Motif11

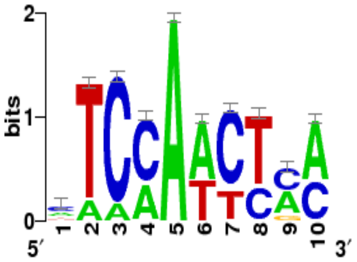

CTCCAACTMA

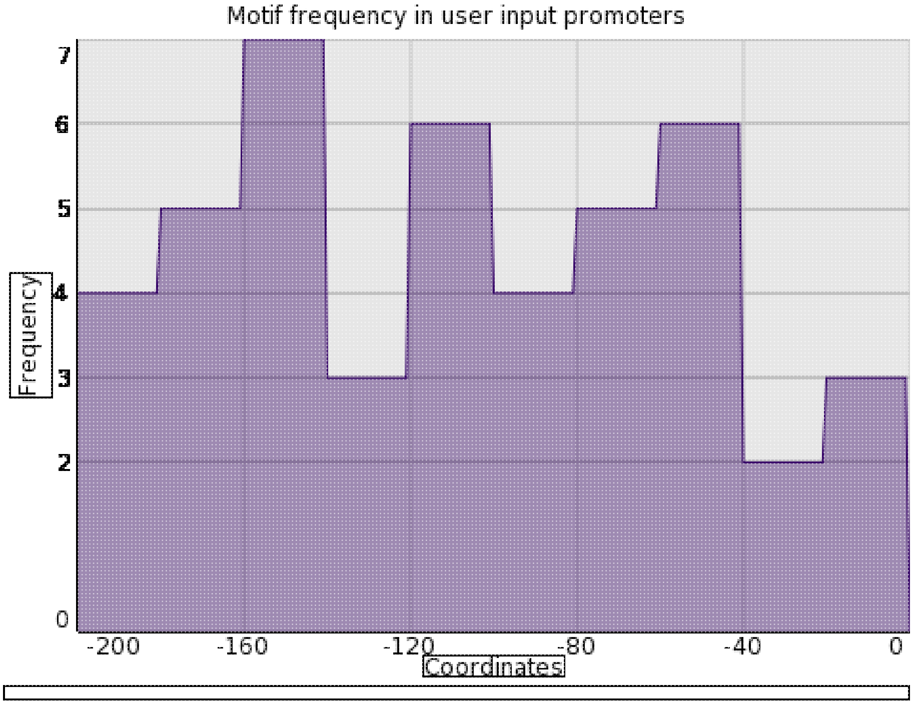

Motif11 annotation in the genome

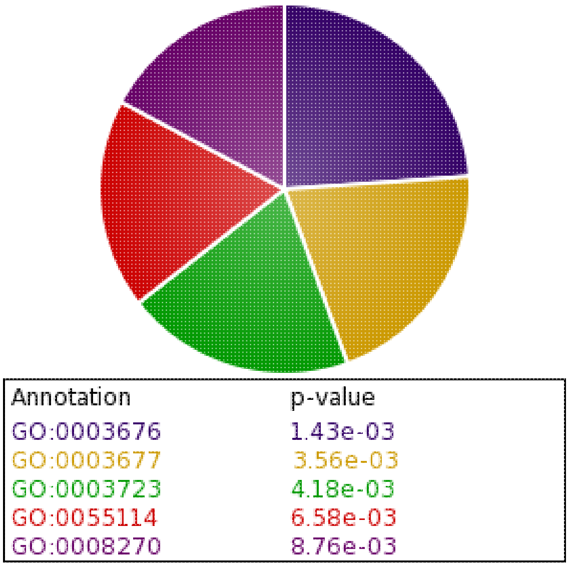

Annotation complete description

GO:0003676 => nucleic acid binding GO:0003677 => DNA binding GO:0003723 => RNA binding GO:0055114 => oxidation reduction GO:0004713 => protein tyrosine kinase activity GO:0008270 => zinc ion binding

Genome-wide Motif11 search results

Motif11 gene list of over-represented annotation(s)

Sequence logo generated by weblogo
Graphic generated with Chart::Clicker,Perl module
Promzea program from the Raizada lab
