# Supplementary material for: Promzea: a pipeline for discovery of co-regulatory motifs in maize and other plant species and its application to the anthocyanin and phlobaphene biosynthetic pathways and the Maize Development Atlas
Source: BMC Plant Biol. 2013 Mar 15;13:42. doi: 10.1186/1471-2229-13-42 (PMC3658923; doi:10.1186/1471-2229-13-42)
Supplement: Additional file 7 — Supplemental files for testing Promzea with data sets from the Maize Development Atlas. The zip folder contains 3 folders. The first contains the promoter input for Promzea for each maize tissue; the second folder has all the outputs from Promzea; the third folder contains the STAMP website outputs for comparisons of the predicted motifs with experimentally defined motifs. [file 1471-2229-13-42-S7.zip › Supplemental files 3 -Case study 3/3-Promzea similarity STAMP/STAMP-embryo.pdf]

Jump to: Multiple Alignment Motif Tree Motif Matching
Input file: 13 motifs loaded
Settings: Metric=PCC, Alignment=SWU, Gap-open=1000, Gap-extend=1000, -nooverlapalign
Multiple Alignment=IR, Tree=UPGMA, Matching against: Place
Note: All results files are removed nightly at midnight EST. Please save your results by saving "Webpage, complete".
Download results as a PDF
Click here to run STAMP again.

Multiple Alignment

(Consensus sequence representations shown, but multiple alignment was carried out on the matrices)

Motif1:	----GCAYGGYTGS-
Motif2:	--CGACGCGT-----
Motif3:	-----GANGNRGNSG
Motif4:	ARGNAGACGA-----
Motif5:	-----CNCGTCNCNC
Motif6:	-----CACGCGTC--
Motif7:	---GACACG------
Motif8:	-----CACGCGNCSG
Motif9:	---GACGCG------
Motif10:	-----CACGCG----
Motif11:	-GCGACACG------
Motif12:	--CNCYACCNCN---
Motif13:	-----CRCGKCGC--

Familial Profile:
(click for matrix)

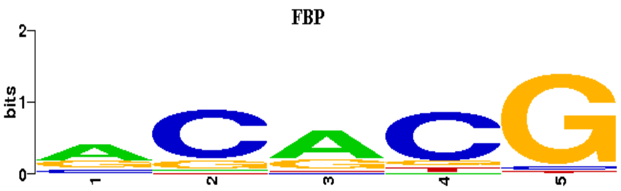

Motif Tree

Tree (drawn by Phylip)
Click here for Newick-format tree (viewable with MEGA)

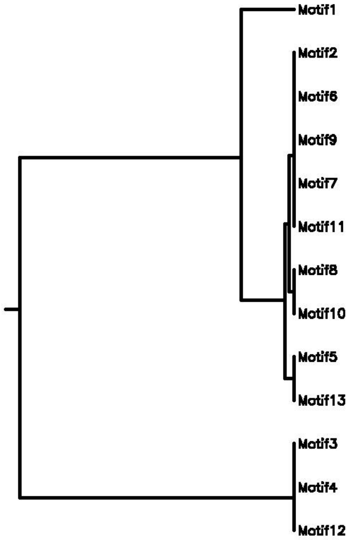

Input Motif	Best match in Place
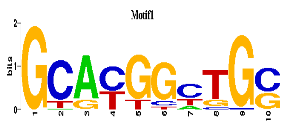 Motif1	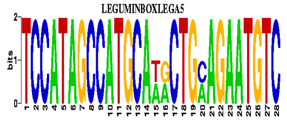 LEGUMINBOXLEGA5 (E val: 6.7609e-05)
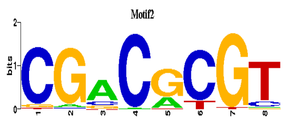 Motif2	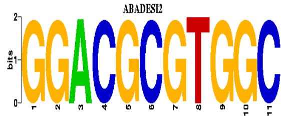 ABADESI2 (E val: 5.0644e-08)
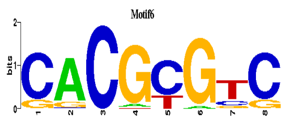 Motif6	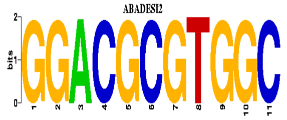 ABADESI2 (E val: 1.1021e-11)
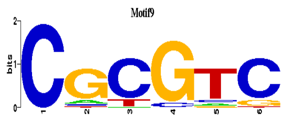 Motif9	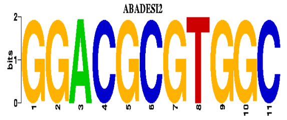 ABADESI2 (E val: 6.6292e-08)
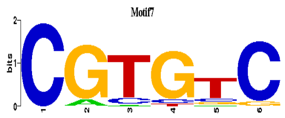 Motif7	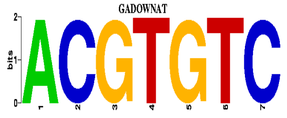 GADOWNAT (E val: 1.1183e-09)
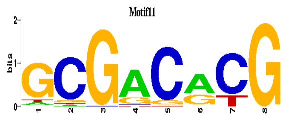 Motif11	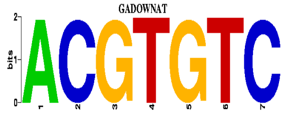 GADOWNAT (E val: 8.9106e-07)
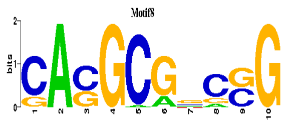 Motif8	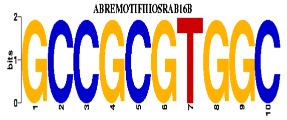 ABREMOTIFIIIOSRAB16B (E val: 4.0996e-09)

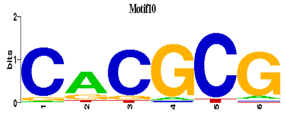 Motif10	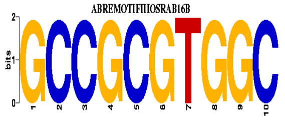 ABREMOTIFIIIOSRAB16B (E val: 3.5108e-08)
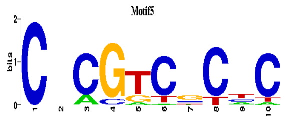 Motif5	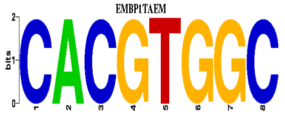 EMBP1TAEM (E val: 1.3216e-06)
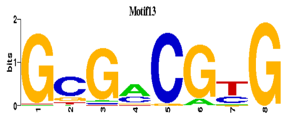 Motif13	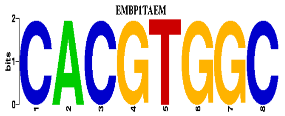 EMBP1TAEM (E val: 4.0159e-08)
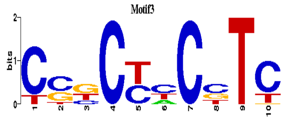 Motif3	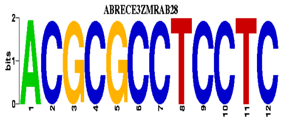 ABRECE3ZMRAB28 (E val: 4.9678e-06)
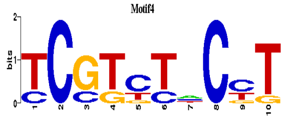 Motif4	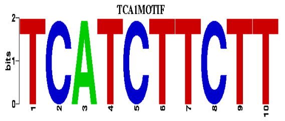 TCA1MOTIF (E val: 4.6937e-05)
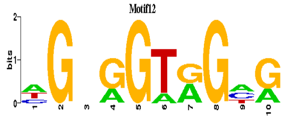 Motif12	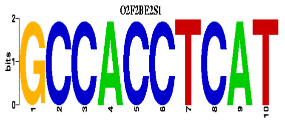 O2F2BE2S1 (E val: 2.6096e-07)

Motif Similarity Matches

Motif1

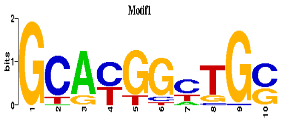

forward

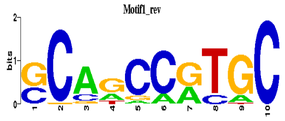

reverse compliment

Name *E value* *Alignment* *Motif*

LEGUMINBOXLEGA5	6.7609e-05	---------------GCAYGGYTGS---<br>GACATTCTKCAGYWTGCATGGCTATGGA	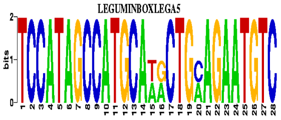
RYREPEAT4	6.5586e-04	-SCARCCRTGC--<br>TCCATGCATGCAC	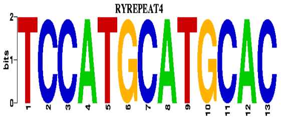
SORLREP5AT	7.4356e-04	SCARCCRTGC--<br>--AGTCATGCAA	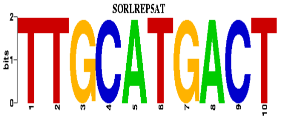
PE3ASPHYA3	7.6958e-04	----GCAYGGYTGS-----------------<br>ACCGGCGCGGATGGGAGAGCCATGGGAGCTG	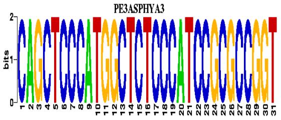
ANAERO2CONSENSUS	8.1115e-04	GCAYGGYTGS-<br>-----GCTGCT	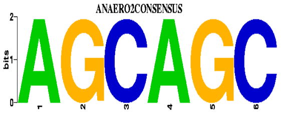

Motif2

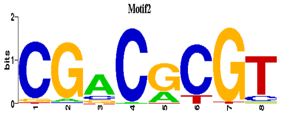

forward

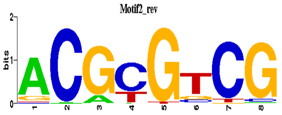

reverse compliment

Name	*E value*	*Alignment*	*Motif*
ABADESI2	5.0644e-08	---ACGCGTCG<br>GCCACGCGTCC	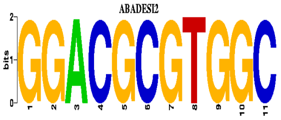
GADOWNAT	1.3606e-07	CGACGCGT<br>-GACACGT	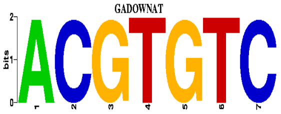
ABREMOTIFAOSOSEM	1.0916e-06	CGACGCGT-<br>-GACACGTA	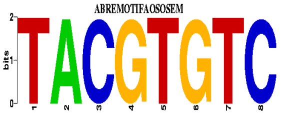
ACGTABREMOTIFAOSOSEM	1.0916e-06	CGACGCGT-<br>-GACACGTA	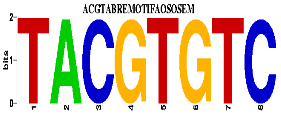

ACGTABREMOTIFA2OSEM	1.6705e-06	CGACGCGT<br>-GMCACGT	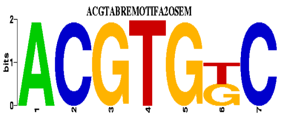

Motif6

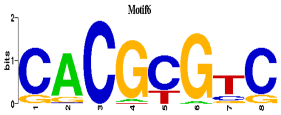

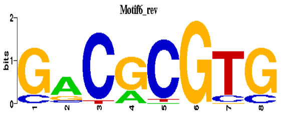

forward *reverse compliment*

Name	*E value*	*Alignment*	*Motif*
ABADESI2	1.1021e-11	-GACGCGTG--<br>GGACGCGTGGC	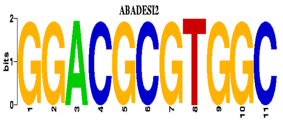
ABRE2HVA22	9.8036e-09	GACGCGTG--<br>GACACGTGCG	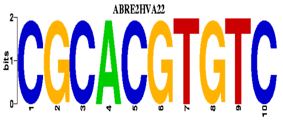
SGBFGMGMAUX28	9.8036e-09	GACGCGTG--<br>GACACGTGGA	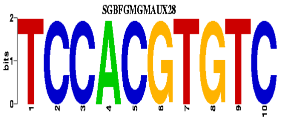
CE3OSOSEM	2.4446e-08	GACGCGTG--<br>AACGCGTGTC	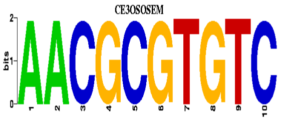
ABRETAEM	2.5709e-08	-GACGCGTG--<br>GGACACGTGGC	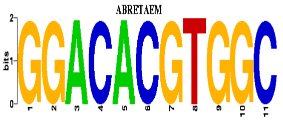

Motif9

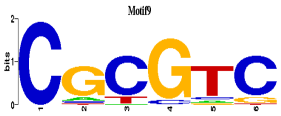

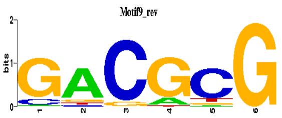

forward *reverse compliment*

Name	*E value*	*Alignment*	*Motif*

ABADESI2	6.6292e-08	----CGCGTC-<br>GCCACGCGTCC	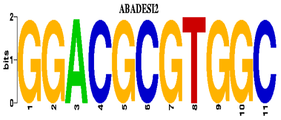
GADOWNAT	2.4902e-05	-CGCGTC<br>ACGTGTC	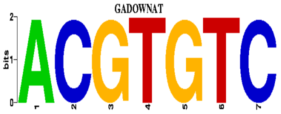
ABREMOTIFAOSOSEM	6.4488e-05	--CGCGTC<br>TACGTGTC	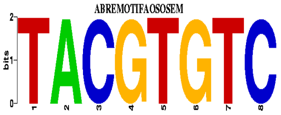
ACGTABREMOTIFAOSOSEM	6.4488e-05	--CGCGTC<br>TACGTGTC	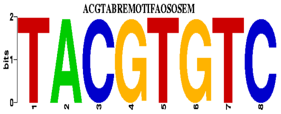
ABREMOTIFIIIOSRAB16B	7.7817e-05	--GACGCG--<br>GCCACGCGGC	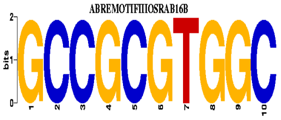

Motif7

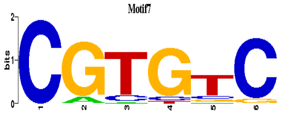

forward

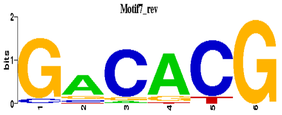

reverse compliment

Name	*E value*	*Alignment*	*Motif*
GADOWNAT	1.1183e-09	-CGTGTC<br>ACGTGTC	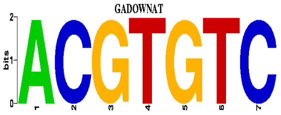
ABREMOTIFAOSOSEM	4.8571e-09	--CGTGTC<br>TACGTGTC	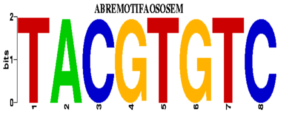
ACGTABREMOTIFAOSOSEM	4.8571e-09	--CGTGTC<br>TACGTGTC	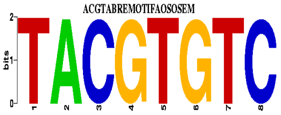
ABRE2HVA22	3.2845e-08	----CGTGTC<br>CGCACGTGTC	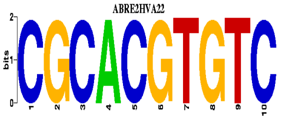

ABRE3HVA1	3.2845e-08	----CGTGTC<br>GCAACGTGTC	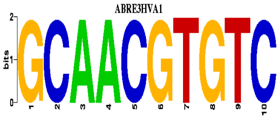

Motif11

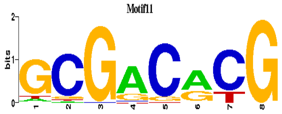

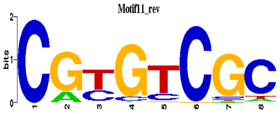

forward *reverse compliment*

Name	*E value*	*Alignment*	*Motif*
GADOWNAT	8.9106e-07	-CGTGTCGC<br>ACGTGTC--	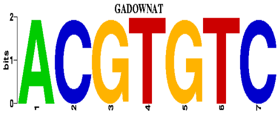
CGTGTSPHZMC1	1.3168e-06	--------GCGACACG<br>ATGCATGGACGACACG	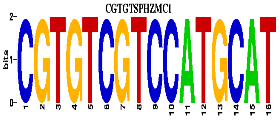
CCTCGTGTCTCGMGH3	1.5866e-06	GCGACACG---<br>GAGACACGAGG	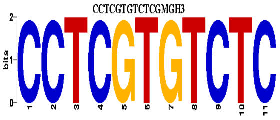
ABRE3OSRAB16	2.0755e-06	GCGACACG---<br>GCGCCACGTAC	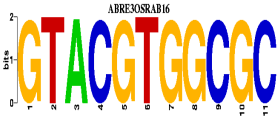
ABREMOTIFAOSOSEM	5.8681e-06	--CGTGTCGC<br>TACGTGTC--	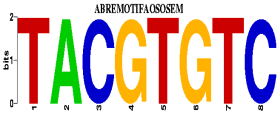

Motif8

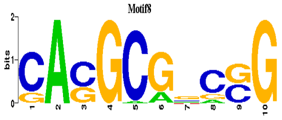

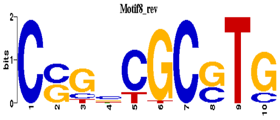

forward *reverse compliment*

Name	*E value*	*Alignment*	*Motif*

ABREMOTIFIIIOSRAB16B	4.0996e-09	CSGNCGCGTG--<br>--GCCGCGTGGC	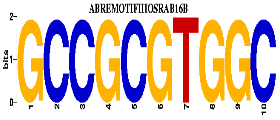
EMBP1TAEM	1.6241e-06	CSGNCGCGTG<br>--GCCACGTG	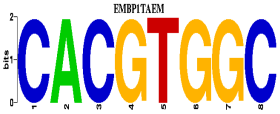
CPRFPCCHS	1.6556e-06	CSGNCGCGTG-<br>-GGCCACGTGG	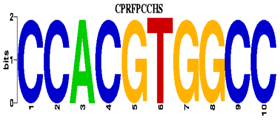
IDRSZMFER1	4.3073e-06	----CSGNCGCGTG<br>GTGGMGGSCTCGTG	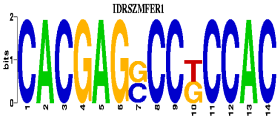
ABRE2HVA1	5.8630e-06	CSGNCGCGTG--<br>CCGCCACGTAGG	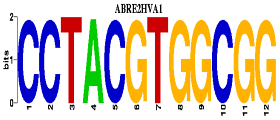

Motif10

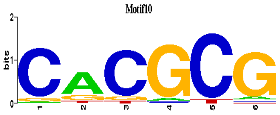

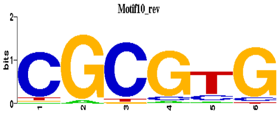

forward *reverse compliment*

Name	*E value*	*Alignment*	*Motif*
ABREMOTIFIIIOSRAB16B	3.5108e-08	--CACGCG--<br>GCCACGCGGC	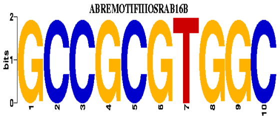
CE3OSOSEM	3.5108e-08	--CACGCG--<br>GACACGCGTT	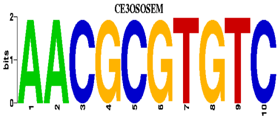
ABADESI2	6.8137e-08	--CACGCG---<br>GCCACGCGTCC	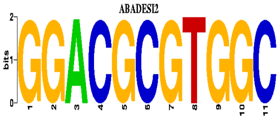
OCETYPEIINTHISTONE	6.8137e-08	-CACGCG----<br>TCACGCGGATC	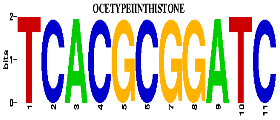

ABRECE3HVA1	1.1643e-07	-----CACGCG-<br>GAGGACACGCGT	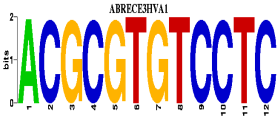

Motif5

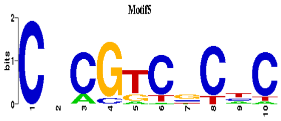

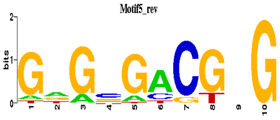

forward *reverse compliment*

Name	*E value*	*Alignment*	*Motif*
EMBP1TAEM	1.3216e-06	GNGNGACGNG<br>--GCCACGTG	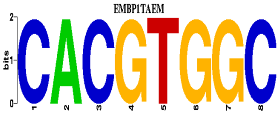
O2F1BE2S1	2.3655e-06	GNGNGACGNG--<br>--TCGACGTGGA	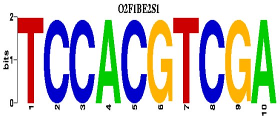
ABRE3HVA1	8.5067e-06	--CNCGTCNCNC<br>GACACGTTGC--	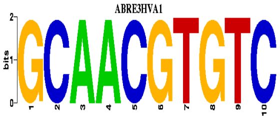
GBOXLERBCS	9.3407e-06	GNGNGACGNG-<br>--GCCACGTGK	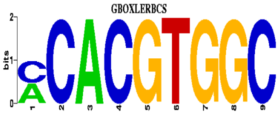
CGACGOSAMY3	1.8685e-05	CNCGTCNCNC<br>--CGTCG---	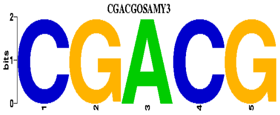

Motif13

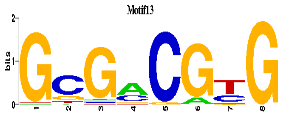

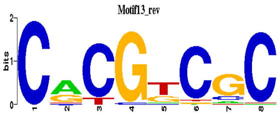

forward *reverse compliment*

Name	*E value*	*Alignment*	*Motif*

EMBP1TAEM	4.0159e-08	GCGMCGYG<br>GCCACGTG	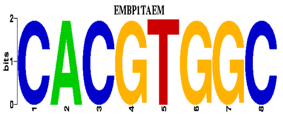
O2F1BE2S1	5.7372e-08	GCGMCGYG--<br>TCGACGTGGA	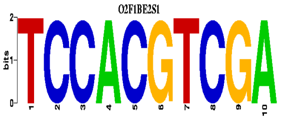
GBOXLERBCS	2.1871e-07	GCGMCGYG-<br>GCCACGTGK	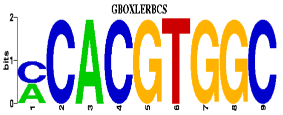
ABRE3HVA1	6.5811e-07	--CRCGKCGC<br>GACACGTTGC	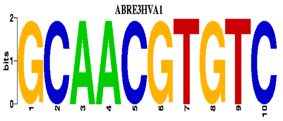
ABREAZMRAB28	6.5811e-07	--CRCGKCGC<br>CCCACGTGGC	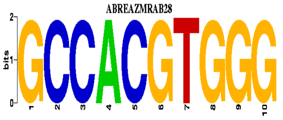

Motif3

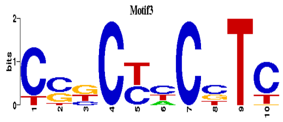

forward

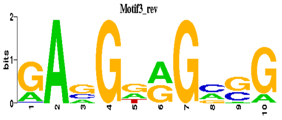

reverse compliment

Name	*E value*	*Alignment*	*Motif*
ABRECE3ZMRAB28	4.9678e-06	GANGNRGNSG--<br>GAGGAGGCGCGT	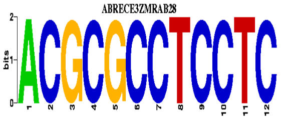
O2F2BE2S1	2.8892e-04	--GANGNRGNSG<br>ATGAGGTGGC--	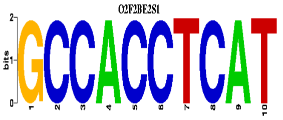
ABRE2HVA1	7.8134e-04	CSNCYNCNTC--<br>CCGCCACGTAGG	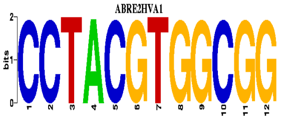
BOXCPSAS1_3	1.5608e-03	GANGNRGNSG<br>GTGGGAG---	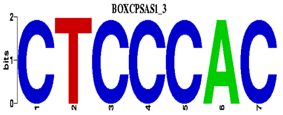

SORLIP5AT	1.5608e-03	CSNCYNCNTC<br>---CTCACTC	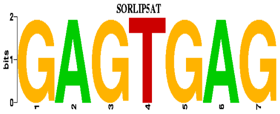

Motif4

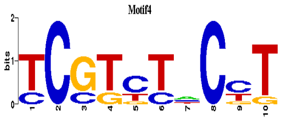
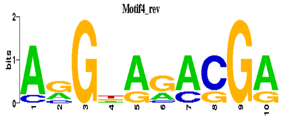

forward *reverse compliment*

Name	*E value*	*Alignment*	*Motif*
TCA1MOTIF	4.6937e-05	TCGTCTNCYT<br>TCATCTTCTT	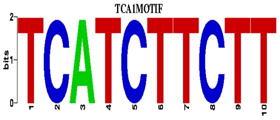
GLUTEBOX2OSGT3	1.3693e-04	--ARGNAGACGA---<br>TAAGGTACACAAAAG	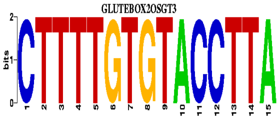
GLUTECOREOS	1.9048e-04	ARGNAGACGA---<br>--GTACACGAAAG	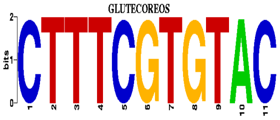
GLUTEBOX2OSGT2	2.5092e-04	ARGNAGACGA-<br>TGGTACACGGA	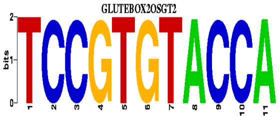
NONAMERATH4	5.8435e-04	TCGTCTNCYT<br>-CGTCGATCT	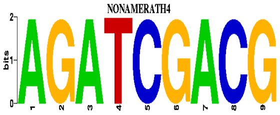

Motif12

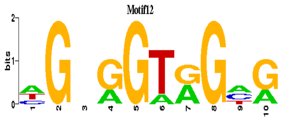
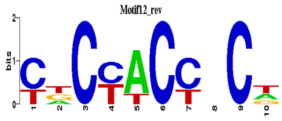

forward *reverse compliment*

Name	*E value*	*Alignment*	*Motif*

O2F2BE2S1	2.6096e-07	-NGNGGTRGNG<br>ATGAGGTGGC-	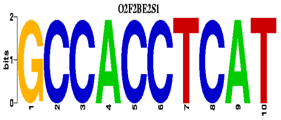
ABRECE3ZMRAB28	3.1009e-05	NGNGGTRGNG---<br>-GAGGAGGCGCGT	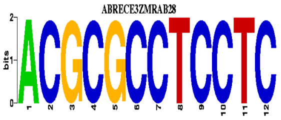
ABREBZMRAB28	4.5803e-05	-NGNGGTRGNG<br>GAGACGTGGA-	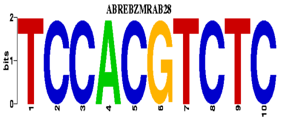
SBOXATRBCS	3.1725e-04	-NGNGGTRGNG<br>TGGAGGTG---	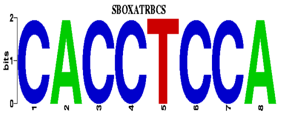
SITEIOSPCNA	3.1725e-04	NGNGGTRGNG<br>CCAGGTGG--	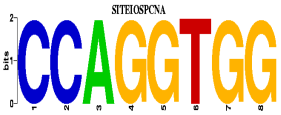

Sequence logo generation powered by weblogo
STAMP is written by Shaun Mahony
