# Supplementary material for: Promzea: a pipeline for discovery of co-regulatory motifs in maize and other plant species and its application to the anthocyanin and phlobaphene biosynthetic pathways and the Maize Development Atlas
Source: BMC Plant Biol. 2013 Mar 15;13:42. doi: 10.1186/1471-2229-13-42 (PMC3658923; doi:10.1186/1471-2229-13-42)
Supplement: Additional file 7 — Supplemental files for testing Promzea with data sets from the Maize Development Atlas. The zip folder contains 3 folders. The first contains the promoter input for Promzea for each maize tissue; the second folder has all the outputs from Promzea; the third folder contains the STAMP website outputs for comparisons of the predicted motifs with experimentally defined motifs. [file 1471-2229-13-42-S7.zip › Supplemental files 3 -Case study 3/3-Promzea similarity STAMP/STAMP-endosperm.pdf]

Jump to: Multiple Alignment Motif Tree Motif Matching
Input file: 12 motifs loaded
Settings: Metric=PCC, Alignment=SWU, Gap-open=1000, Gap-extend=1000, -nooverlapalign
Multiple Alignment=IR, Tree=UPGMA, Matching against: Place
Note: All results files are removed nightly at midnight EST. Please save your results by saving "Webpage, complete".
Download results as a PDF
Click here to run STAMP again.

Multiple Alignment

(Consensus sequence representations shown, but multiple alignment was carried out on the matrices)

```
Motif1:                 TCTMTCTATC-----
Motif2:                 ----TCTATCTC---
Motif3:                 ---RGCTATA-----
Motif4:                 -----CTATCT----
Motif5:                 -CTMTCTNTCY----
Motif6:                 -----NTATAWATAN
Motif7:                 ----TCTATCTMT--
Motif8:                 --TMTCTATC-----
Motif9:                 ----TCTATC-----
Motif10:                ----GCTATA-----
Motif11:                --TNTCTATC-----
Motif12:                -----MTATMTAT--
```

Familial Profile:
(click for matrix)

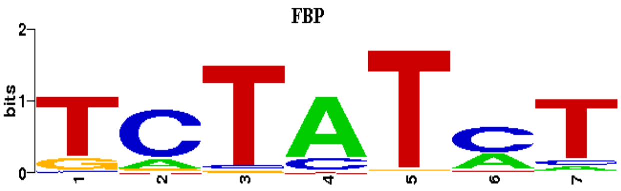

Motif Tree

Tree (drawn by Phylip)
Click here for Newick-format tree (viewable with MEGA)

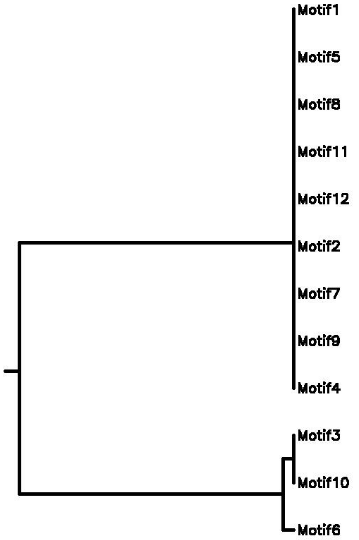

Input Motif	Best match in Place
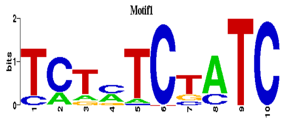 Motif1	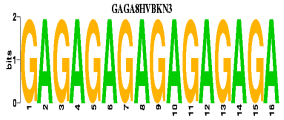 GAGA8HVBKN3 (E val: 2.6031e-09)
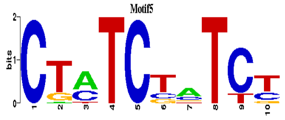 Motif5	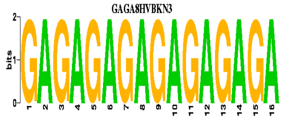 GAGA8HVBKN3 (E val: 1.0986e-07)
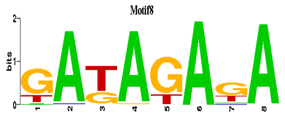 Motif8	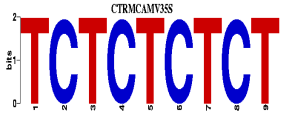 CTRMCAMV35S (E val: 2.0995e-09)
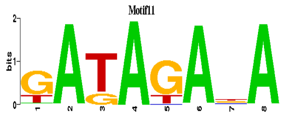 Motif11	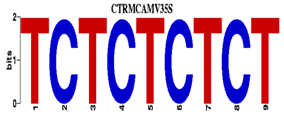 CTRMCAMV35S (E val: 5.2202e-07)
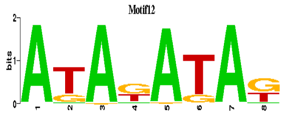 Motif12	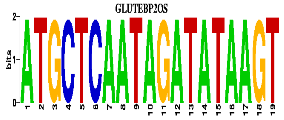 GLUTEBP2OS (E val: 2.5633e-07)
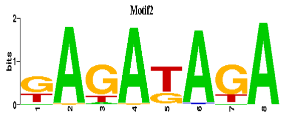 Motif2	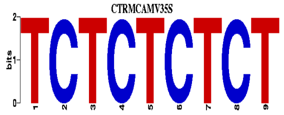 CTRMCAMV35S (E val: 3.3848e-09)
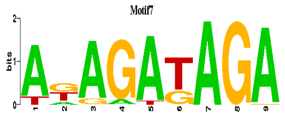 Motif7	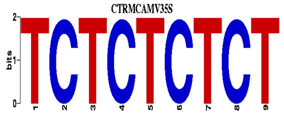 CTRMCAMV35S (E val: 5.6102e-11)
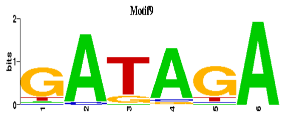 Motif9	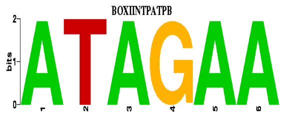 BOXIINTPATPB

	(E val: 1.1143e-06)
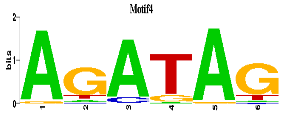 Motif4	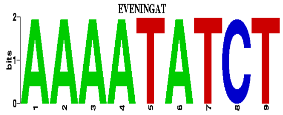 EVENINGAT (E val: 4.6380e-05)
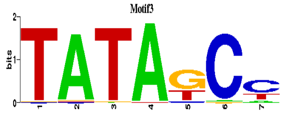 Motif3	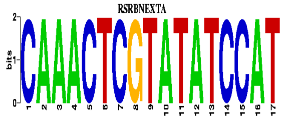 RSRBNEXTA (E val: 1.4274e-05)
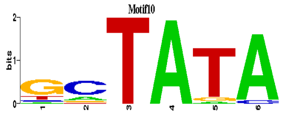 Motif10	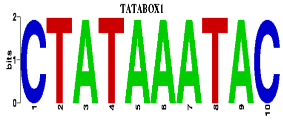 TATABOX1 (E val: 7.9785e-05)
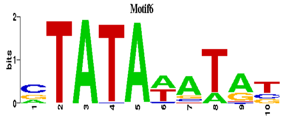 Motif6	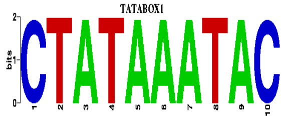 TATABOX1 (E val: 2.1685e-11)

Motif Similarity Matches

Motif1

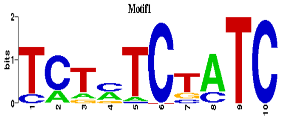

forward

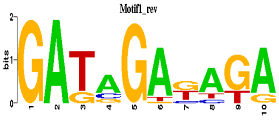

reverse compliment

Name	*E value*	*Alignment*	*Motif*
GAGA8HVBKN3	2.6031e-09	TCTMTCTATC------<br>TCTCTCTCTCTCTCTC	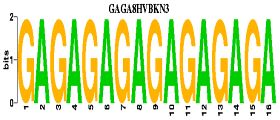
CTRMCAMV35S	3.5671e-09	TCTMTCTATC<br>TCTCTCTCT-	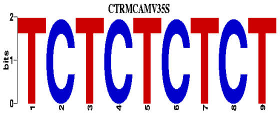
GAGAGMGSA1	6.6595e-09	TCTMTCTATC--------<br>TCTCTCTCTCTCTCTCTC	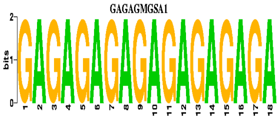

GLUTEBP2OS	7.9339e-06	---TCTMTCTATC------<br>ACTTATATCTATTGAGCAT	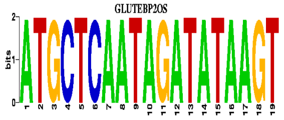
LREBOX3PSRBCS3	1.1057e-04	-----TCTMTCTATC<br>ACTATTTTCACTATC	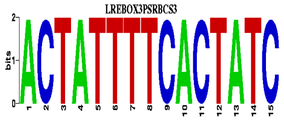

Motif5

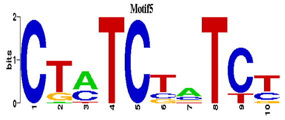

forward

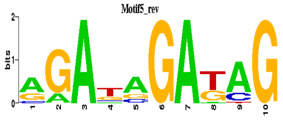

reverse compliment

Name	*E value*	*Alignment*	*Motif*
GAGA8HVBKN3	1.0986e-07	-CTMTCTNTCY-----<br>TCTCTCTCTCTCTCTC	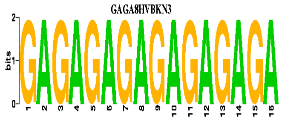
CTRMCAMV35S	1.8431e-07	RGANAGAKAG<br>AGAGAGAGA-	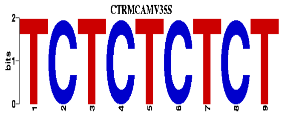
GAGAGMGSA1	2.5376e-07	-CTMTCTNTCY-------<br>TCTCTCTCTCTCTCTCTC	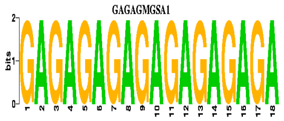
3AF1BOXPSRBCS3	7.2207e-06	-----------CTMTCTNTCY<br>AATGTTTTTATTTATCTATTT	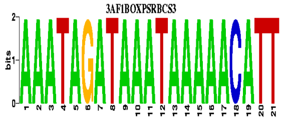
AMYBOX2	1.3321e-05	RGANAGAKAG<br>--ATGGATA-	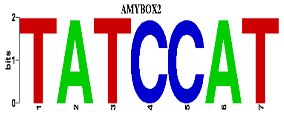

Motif8

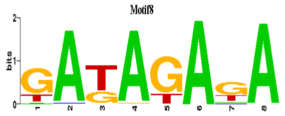

forward

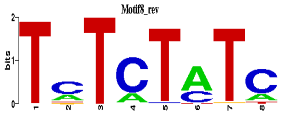

reverse compliment

Name	*E value*	*Alignment*	*Motif*
CTRMCAMV35S	2.0995e-09	TMTCTATC-<br>TCTCTCTCT	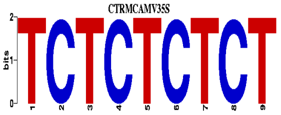
GAGA8HVBKN3	2.5639e-07	TMTCTATC--------<br>TCTCTCTCTCTCTCTC	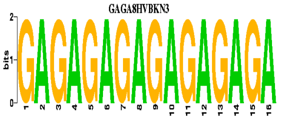
GAGAGMGSA1	4.2099e-07	TMTCTATC----------<br>TCTCTCTCTCTCTCTCTC	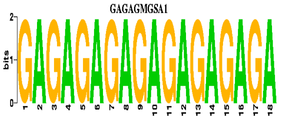
LREBOX3PSRBCS3	1.2940e-05	-------TMTCTATC<br>ACTATTTTCACTATC	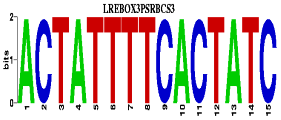
BOXIINTPATPB	5.2418e-05	TMTCTATC<br>-TTCTAT-	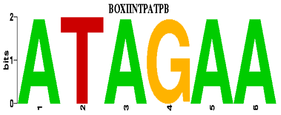

Motif11

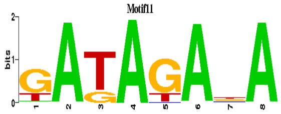

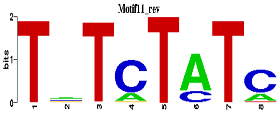

forward *reverse compliment*

Name	*E value*	*Alignment*	*Motif*
CTRMCAMV35S	5.2202e-07	-GATAGANA<br>AGAGAGAGA	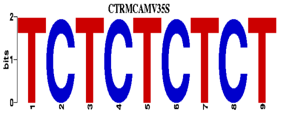
GLUTEBP2OS	2.1853e-05	-----TNTCTATC------<br>ACTTATATCTATTGAGCAT	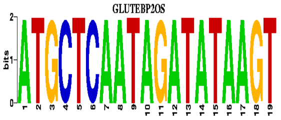
GAGA8HVBKN3	2.6158e-05	GATAGANA--------<br>GAGAGAGAGAGAGAGA	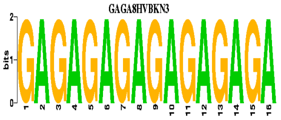
BOXIINTPATPB	3.0560e-05	GATAGANA<br>-ATAGAA-	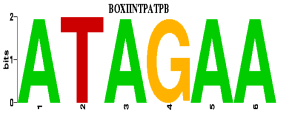

3AF1BOXPSRBCS3	3.2196e-05	------------TNTCTATC-<br>AATGTTTTTATTTATCTATTT	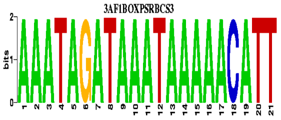

Motif12

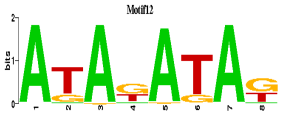

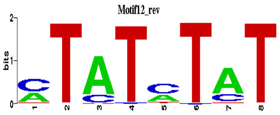

forward *reverse compliment*

Name	*E value*	*Alignment*	*Motif*
GLUTEBP2OS	2.5633e-07	-------ATAKATAK----<br>ATGCTCAATAGATATAAGT	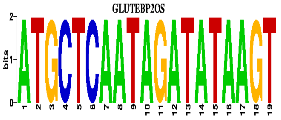
SORLREP3AT	9.5159e-07	ATAKATAK-<br>ATATATACA	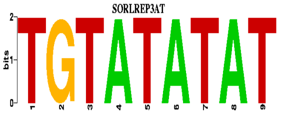
3AF1BOXPSRBCS3	4.2714e-06	--ATAKATAK-----------<br>AAATAGATAAATAAAAACATT	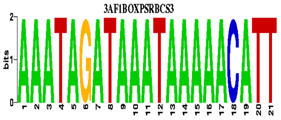
BOXINTPATPB	4.2714e-06	-----------ATAKATAK--<br>AATTCCATAGAATAGATAATA	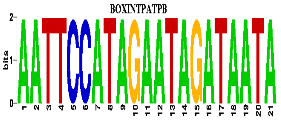
AMYBOX2	1.1186e-05	ATAKATAK<br>ATGGATA-	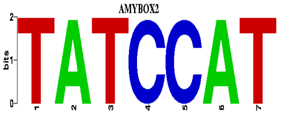

Motif2

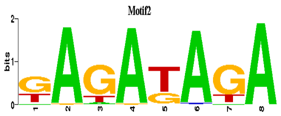

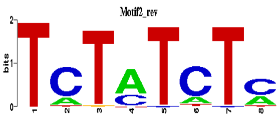

forward *reverse compliment*

Name	*E value*	*Alignment*	*Motif*

CTRMCAMV35S	3.3848e-09	-GAGATAGA<br>AGAGAGAGA	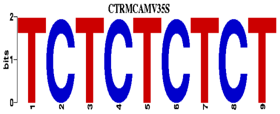
GAGA8HVBKN3	3.8428e-07	TCTATCTC--------<br>TCTCTCTCTCTCTCTC	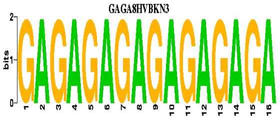
GAGAGMGSA1	6.2857e-07	TCTATCTC----------<br>TCTCTCTCTCTCTCTCTC	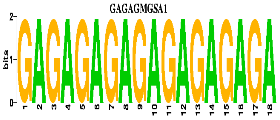
BOXIINTPATPB	4.3895e-05	-TCTATCTC<br>TTCTAT---	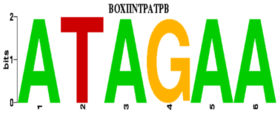
NONAMERATH4	9.7255e-05	--TCTATCTC<br>CGTCGATCT-	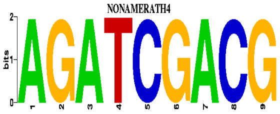

Motif7

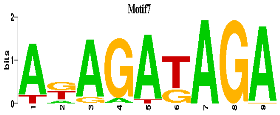

forward

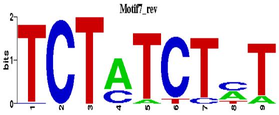

reverse compliment

Name	*E value*	*Alignment*	*Motif*
CTRMCAMV35S	5.6102e-11	TCTATCTMT<br>TCTCTCTCT	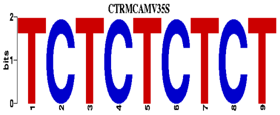
GAGA8HVBKN3	4.2903e-08	TCTATCTMT-------<br>TCTCTCTCTCTCTCTC	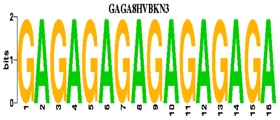
GAGAGMGSA1	8.5009e-08	TCTATCTMT---------<br>TCTCTCTCTCTCTCTCTC	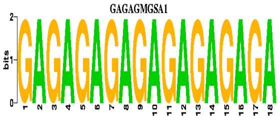
GLUTEBP2OS	8.5087e-06	---TCTATCTMT-------<br>ACTTATATCTATTGAGCAT	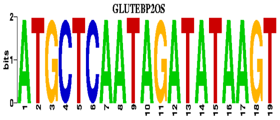

3AF1BOXPSRBCS3	1.1619e-05	----------TCTATCTMT--<br>AATGTTTTTATTTATCTATTT	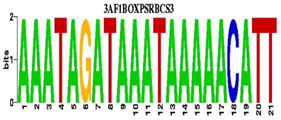

Motif9

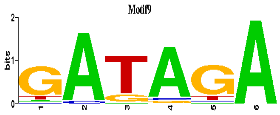

forward

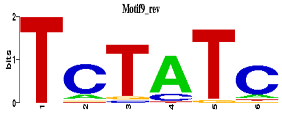

reverse compliment

Name	*E value*	*Alignment*	*Motif*
BOXIINTPATPB	1.1143e-06	-TCTATC<br>TTCTAT-	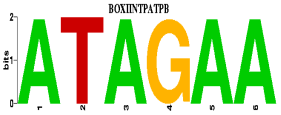
SURE1STPAT21	4.7281e-05	---TCTATC<br>TTTTCTATT	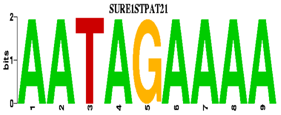
LS7ATPR1	8.1285e-05	TCTATC----<br>TCTATGACGT	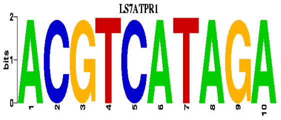
CTRMCAMV35S	1.1498e-04	-GATAGA--<br>AGAGAGAGA	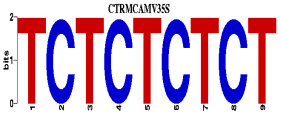
BOX1PSGS2	1.3065e-04	------TCTATC<br>TTGATTTCTAT-	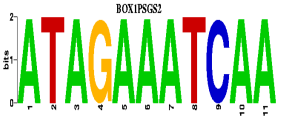

Motif4

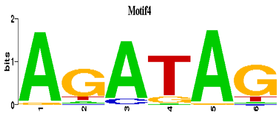

forward

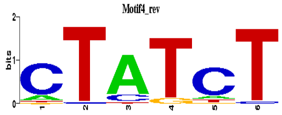

reverse compliment

Name	*E value*	*Alignment*	*Motif*

EVENINGAT	4.6380e-05	AGATAG---<br>AGATATTTT	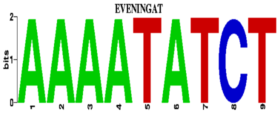
RGATAOS	4.6380e-05	CTATCT----<br>-TATCTTCTG	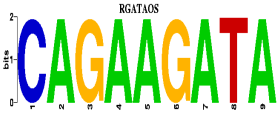
CTRMCAMV35S	1.3832e-04	AGATAG---<br>AGAGAGAGA	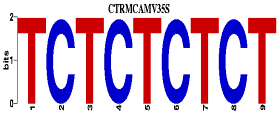
IBOXLSCMCUCUMISIN	2.3980e-04	-------CTATCT<br>TTTTATCATATCT	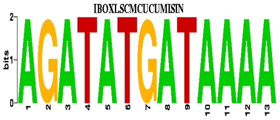
GATABOX	4.1450e-04	CTATCT<br>-TATC-	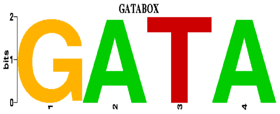

Motif3

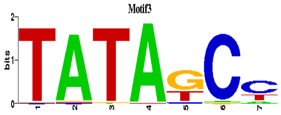

forward

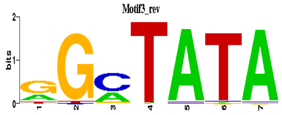

reverse compliment

Name	*E value*	*Alignment*	*Motif*
RSRBNEXTA	1.4274e-05	--RGCTATA--------<br>ATGGATATACGAGTTTG	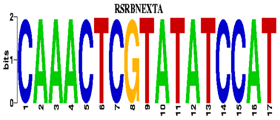
LEGUMINBOXLEGA5	1.6713e-04	-------------------RGCTATA--<br>GACATTCTKCAGYWTGCATGGCTATGGA	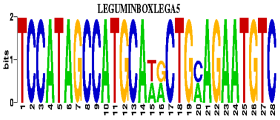
5256BOXLELAT5256	2.9756e-04	--TATAGCY---<br>TATATAACCACA	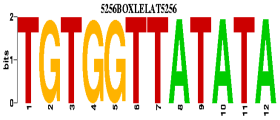
MYBST1	8.0741e-04	TATAGCY<br>--TATCC	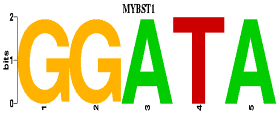

TATABOX1	1.1867e-03	-----TATAGCY<br>GTATTTATAG--	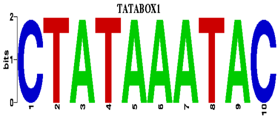

Motif10

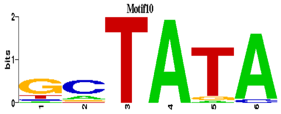

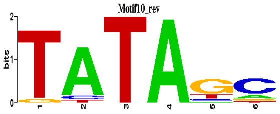

forward *reverse compliment*

Name	*E value*	*Alignment*	*Motif*
TATABOX1	7.9785e-05	-----TATAGC<br>GTATTTATAG-	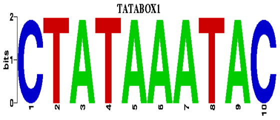
AACAOSGLUB1	3.0546e-04	--TATAGC------<br>GATATAGTTTGTTG	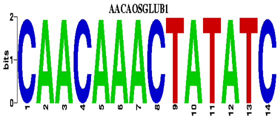
BBOXSITE1STPAT	5.5927e-04	----TATAGC<br>ATTGTTTAGC	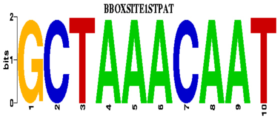
PE1ASPHYA3	1.1429e-03	--------------GCTATA--<br>TATTTTTAACATTTGCTATTTC	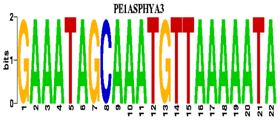
RSRBNEXTA	1.1667e-03	---GCTATA--------<br>ATGGATATACGAGTTTG	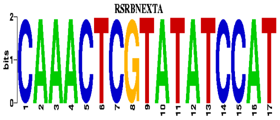

Motif6

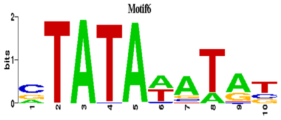

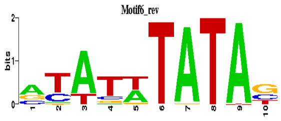

forward *reverse compliment*

Name	*E value*	*Alignment*	*Motif*

TATABOX1	2.1685e-11	NTATWTATAN<br>GTATTTATAG	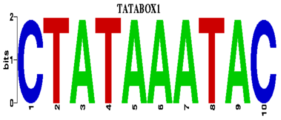
SEF1MOTIF	1.7613e-09	NTATAWATAN<br>-WWTAAATAT	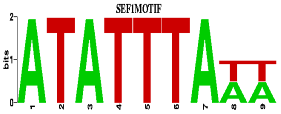
TATABOX2	1.0288e-08	NTATWTATAN<br>--ATTTATA-	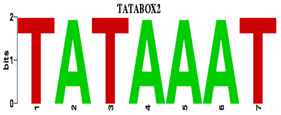
SORLREP3AT	2.0960e-07	NTATWTATAN<br>ATATATACA-	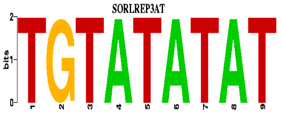
TATAPVTRNALEU	1.2766e-06	NTATAWATAN<br>-TATATAAA-	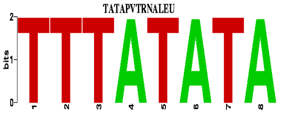

Sequence logo generation powered by weblogo
STAMP is written by Shaun Mahony
