# Supplementary material for: Promzea: a pipeline for discovery of co-regulatory motifs in maize and other plant species and its application to the anthocyanin and phlobaphene biosynthetic pathways and the Maize Development Atlas
Source: BMC Plant Biol. 2013 Mar 15;13:42. doi: 10.1186/1471-2229-13-42 (PMC3658923; doi:10.1186/1471-2229-13-42)
Supplement: Additional file 7 — Supplemental files for testing Promzea with data sets from the Maize Development Atlas. The zip folder contains 3 folders. The first contains the promoter input for Promzea for each maize tissue; the second folder has all the outputs from Promzea; the third folder contains the STAMP website outputs for comparisons of the predicted motifs with experimentally defined motifs. [file 1471-2229-13-42-S7.zip › Supplemental files 3 -Case study 3/3-Promzea similarity STAMP/STAMP-internode.pdf]

Jump to: Multiple Alignment Motif Tree Motif Matching

Input file: 14 motifs loaded

Settings: Metric=PCC, Alignment=SWU, Gap-open=1000, Gap-extend=1000, -nooverlapalign

Multiple Alignment=IR, Tree=UPGMA, Matching against: ALL

Note: All results files are removed nightly at midnight EST. Please save your results by saving "Webpage, complete".

Download results as a PDF

Click here to run STAMP again.

Multiple Alignment

(Consensus sequence representations shown, but multiple alignment was carried out on the matrices)

```
Motif1:     ---CATGGAATYA-
Motif2:     ---CATGGNMT---
Motif3:     ----GTGGTG----
Motif4:     ----GTGNTGCWGG
Motif5:     ---GGYGGKGSCG-
Motif6:     --GTGTGGG-----
Motif7:     ----GNKNGAGG--
Motif8:     --GTGTGGGCT---
Motif9:     GGCGGKGNCG----
Motif10:    GGCGGKGNCG----
Motif11:    ---GGTGGACA---
Motif12:    -AGTGTGTR-----
Motif13:    ---TGTGGG-----
Motif14:    ---AGTGTG-----
```

Familial Profile:
(click for matrix)

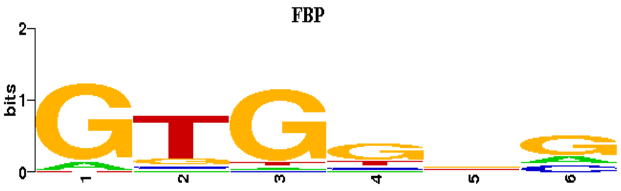

Motif Tree

Tree (drawn by Phylip)

Click here for Newick-format tree (viewable with MEGA)

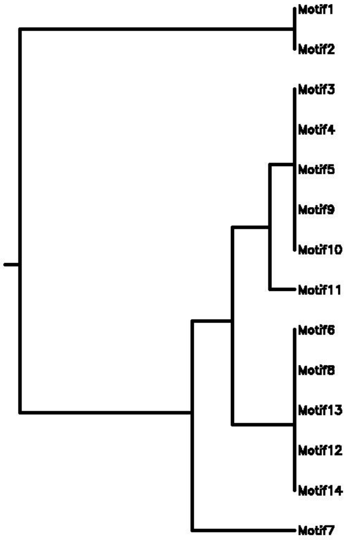

Input Motif	Best match in ALL
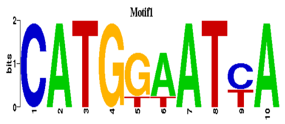 Motif1	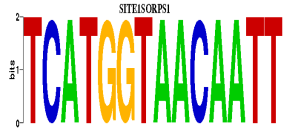 SITE1SORPS1 (E val: 2.2389e-06)
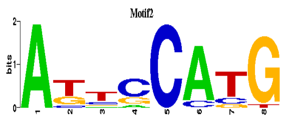 Motif2	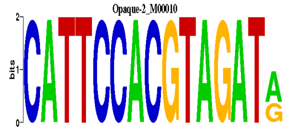 Opaque-2_M00010 (E val: 2.4183e-06)
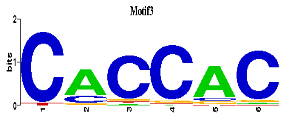 Motif3	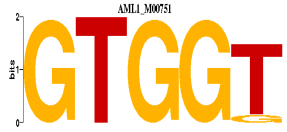 AML1_M00751 (E val: 1.4256e-07)
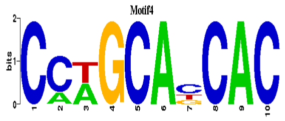 Motif4	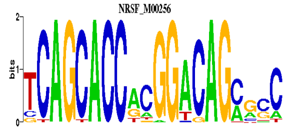 NRSF_M00256 (E val: 4.6695e-08)
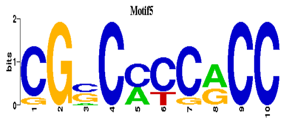 Motif5	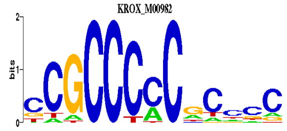 KROX_M00982 (E val: 7.0532e-11)
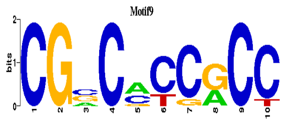 Motif9	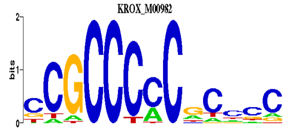 KROX_M00982 (E val: 2.4470e-10)
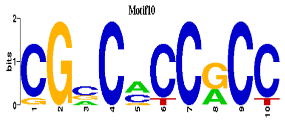 Motif10	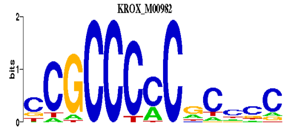 KROX_M00982 (E val: 7.4037e-12)

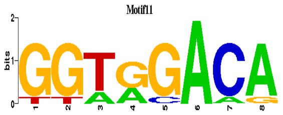 Motif11	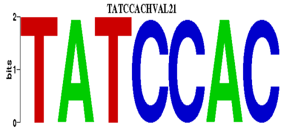 TATCCACHVAL21 (E val: 2.0407e-05)
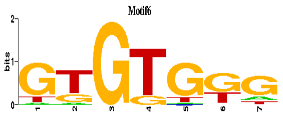 Motif6	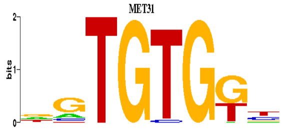 MET31 (E val: 5.5123e-07)
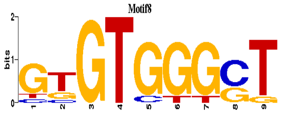 Motif8	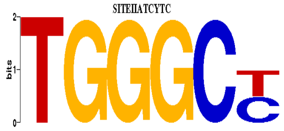 SITEIIATCYTC (E val: 4.4312e-06)
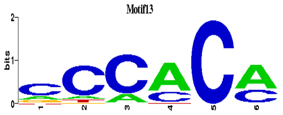 Motif13	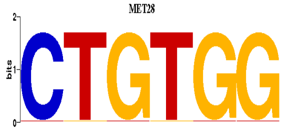 MET28 (E val: 7.6540e-06)
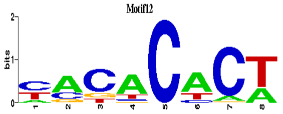 Motif12	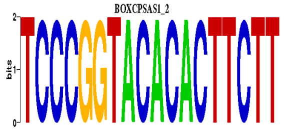 BOXCPSAS1_2 (E val: 3.1708e-07)
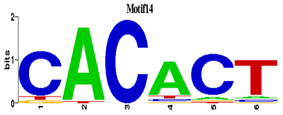 Motif14	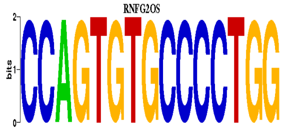 RNFG2OS (E val: 2.3832e-07)
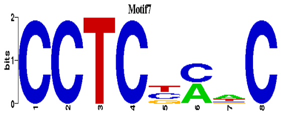 Motif7	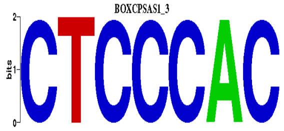 BOXCPSAS1_3 (E val: 1.5564e-07)

Motif Similarity Matches

Motif1

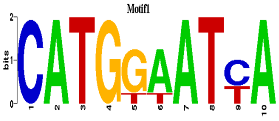

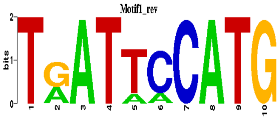

forward *reverse compliment*

Name	*E value*	*Alignment*	*Motif*
SITE1SORPS1	2.2389e-06	---TRATTCCATG-<br>AATTGTTACCATGA	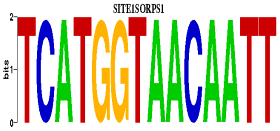
S1FSORPL21	3.0292e-06	TRATTCCATG<br>-AATACCAT-	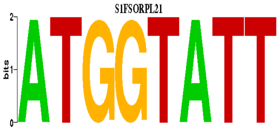
ESPASGL01	3.5612e-06	---TRATTCCATG-<br>ACATGATGACATGT	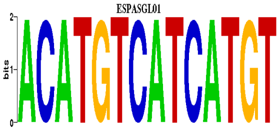
STM1555	1.0838e-05	-CATGGAATYA---------<br>TCATGTAAKCGSTTWCATGA	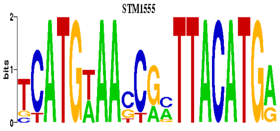
RbsR_SX	1.2061e-05	-CATGGAATYA---------<br>TCGTGGAATCGATTACAYSG	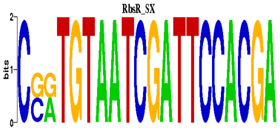

Motif2

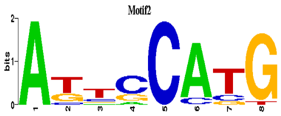

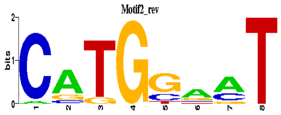

forward *reverse compliment*

Name	*E value*	*Alignment*	*Motif*
Opaque-2_M00010	2.4183e-06	------CATGGNMT-<br>YATCTACGTGGAATG	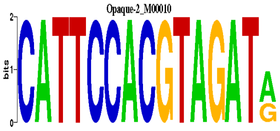
BOXINTPATPB	3.3819e-06	------------CATGGNMT-<br>TATTATCTATTCTATGGAATT	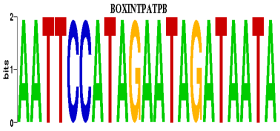
RbsR_SX	8.5149e-06	-----------AKNCCATG-<br>CSRTGTAATCGATTCCACGA	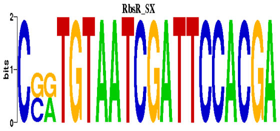

SPHCOREZMC1	9.7815e-06	AKNCCATG---<br>--TCCATGCAT	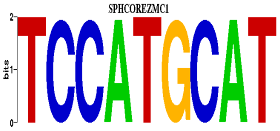
SPHZMC1	2.3777e-05	---CATGGNMT<br>ATGCATGGACG	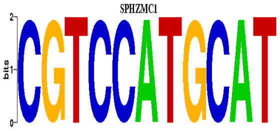

Motif3

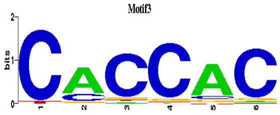

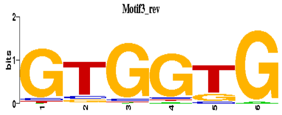

forward *reverse compliment*

Name	*E value*	*Alignment*	*Motif*
AML1_M00751	1.4256e-07	CACCAC<br>-ACCAC	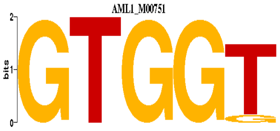
AML1a_M00271	3.1268e-07	CACCAC<br>-ACCAC	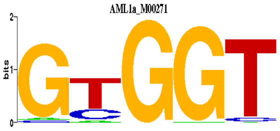
NRSF_M00256	1.8725e-06	----------GTGGTG----<br>GSYGCTGTCCGTGGTGCTGA	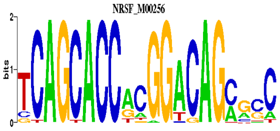
R_M00273	2.5453e-06	--CACCAC------------<br>NRCACCRCRNNNSGNGGMCN	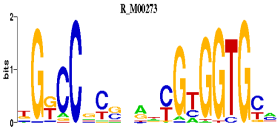
core-binding_M00722	9.7582e-06	-CACCAC<br>WNACCAC	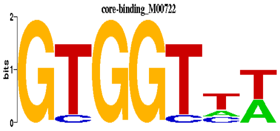

Motif4

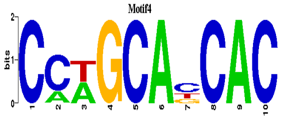

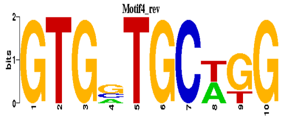

forward *reverse compliment*

Name	*E value*	*Alignment*	*Motif*
NRSF_M00256	4.6695e-08	----------GTGNTGCWGG<br>GSYGCTGTCCGTGGTGCTGA	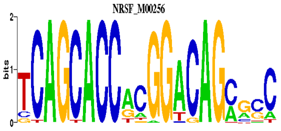
NRSF_M01028	3.5453e-07	CCWGCANCAC--------<br>TCAGCACCRYGGACAGNR	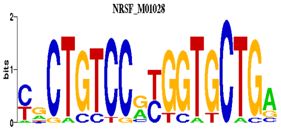
INTRONLOWER	1.5274e-06	CCWGCANCAC<br>CCTGCA----	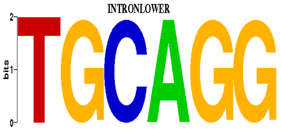
NRSE_M00325	1.5344e-05	----------GTGNTGCWGG<br>GGCRCTCTCCGNGGTGCTGA	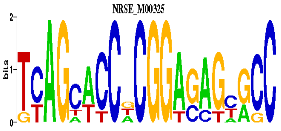
REST	1.6313e-05	CCWGCANCAC---------<br>TCAGCACCWNGGACAGCNC	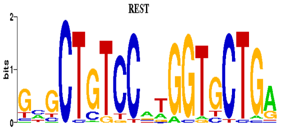

Motif5

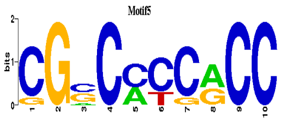

forward

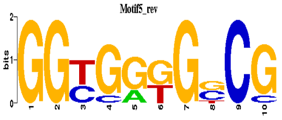

reverse compliment

Name	*E value*	*Alignment*	*Motif*
KROX_M00982	7.0532e-11	--GGYGGKGSCG-<br>GNNGNGGGGGCGG	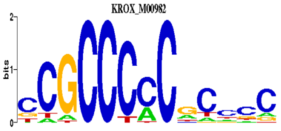
Alfin1_M00479	2.4678e-09	CGSCMCCRCC----<br>MNNCCCCACNYNYN	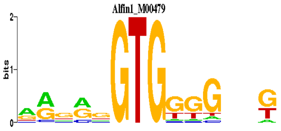
REGION1OSOSEM	3.8442e-07	---GGYGGKGSCG---<br>CGTGGCGAGGCCGCCG	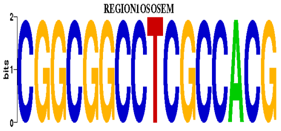
GCBP2ZMGAPC4	2.0782e-06	GGYGGKGSCG<br>-GTGGGCCCG	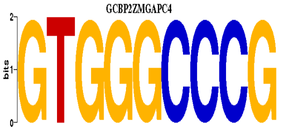

Egr_M00807	1.1485e-05	--CGSCMCCRCC<br>NYYGCSCCCA--	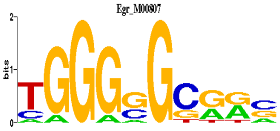

Motif9

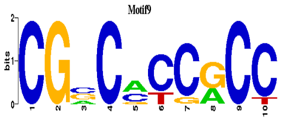

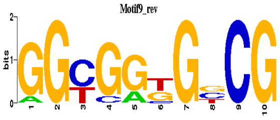

forward *reverse compliment*

Name	*E value*	*Alignment*	*Motif*
KROX_M00982	2.4470e-10	--GGCGGKGNCG-<br>GNNGNGGGGGCGG	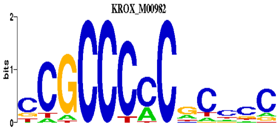
ABI4_M00958	6.8020e-07	-GGCGGKGNCG<br>GGGCGGTGCN-	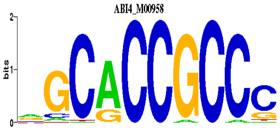
REGION1OSOSEM	7.2914e-07	---CGNCMCCGCC---<br>CGGCGGCCTCGCCACG	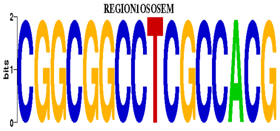
Alfin1_M00479	1.3181e-06	CGNCMCCGCC----<br>MNNCCCCACNYNYN	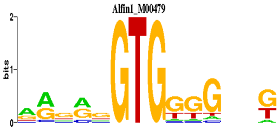
E12_M00693	3.2056e-05	GGCGGKGNCG<br>RCAGGTGNNN	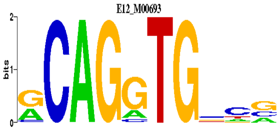

Motif10

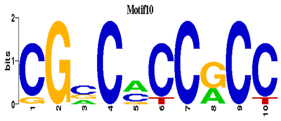

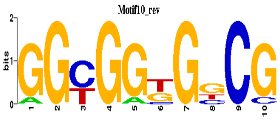

forward *reverse compliment*

Name	*E value*	*Alignment*	*Motif*

KROX_M00982	7.4037e-12	--GGCGGKGNCG-<br>GNNGNGGGGGCGG	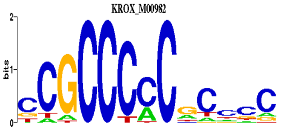
Alfin1_M00479	7.0547e-06	----GGCGGKGNCG<br>NRNRNGTGGGGNNK	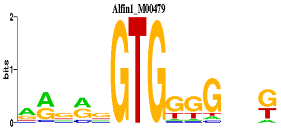
ABRECE1HVA22	8.2497e-06	GGCGGKGNCG<br>-CCGGTGGCA	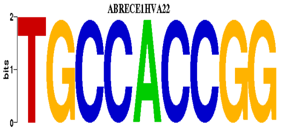
ABI4_M00958	9.0890e-06	-GGCGGKGNCG<br>GGGCGGTGCN-	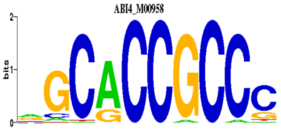
REGION1OSOSEM	3.9124e-05	---CGNCMCCGCC---<br>CGGCGGCCTCGCCACG	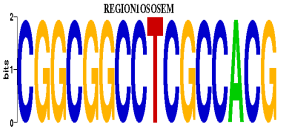

Motif11

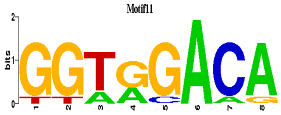

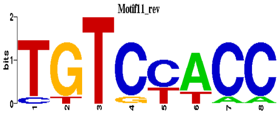

forward *reverse compliment*

Name	*E value*	*Alignment*	*Motif*
TATCCACHVAL21	2.0407e-05	GGTGGACA<br>-GTGGATA	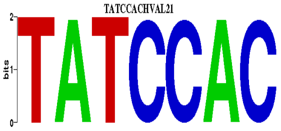
POLLEN2LELAT52	2.7874e-05	---GGTGGACA<br>TATGGTGGA--	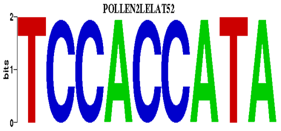
UPRE1AT	1.2281e-04	GGTGGACA---<br>CGTGGACCAAT	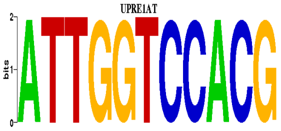
ZNF354C	1.3468e-04	GGTGGACA<br>-GTGGAK-	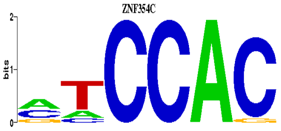

GLUTEBOX2OSGT3	1.5062e-04	---GGTGGACA----<br>TAAGGTACACAAAAG	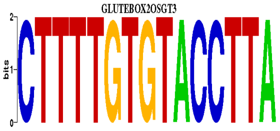

Motif6

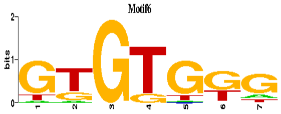

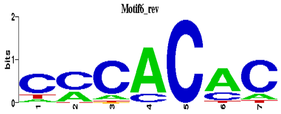

forward *reverse compliment*

Name	*E value*	*Alignment*	*Motif*
MET31	5.5123e-07	CCCACAC-<br>NMCACACN	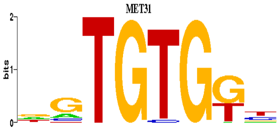
CAC-binding_M00720	3.0926e-05	CCCACAC-<br>CCCASCCY	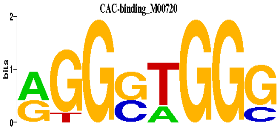
Tra-1_M01049	3.1354e-05	CCCACAC<br>ACCNCCC	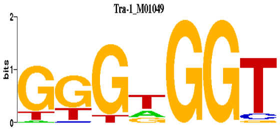
RREB-1_M00257	3.2644e-05	CCCACAC------<br>CCCAAACCACCCC	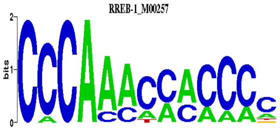
CACD_M01113	3.3594e-05	GTGTGGG<br>GGGYGTG	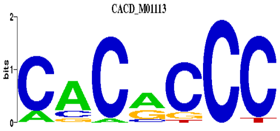

Motif8

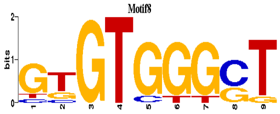

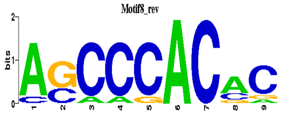

forward *reverse compliment*

Name	*E value*	*Alignment*	*Motif*

SITEIIATCYTC	4.4312e-06	GTGTGGGCT<br>---TGGGCY	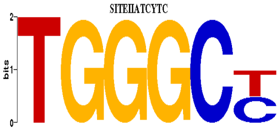
SREBP-1_M00221	1.7310e-05	GTGTGGGCT---<br>--GTGGGGTGAT	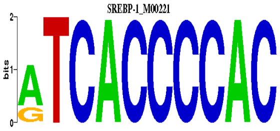
MET31	2.9701e-05	AGCCCACAC-<br>--NMCACACN	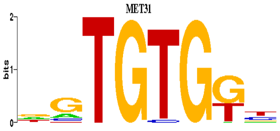
Egr-3_M00245	3.2906e-05	-AGCCCACAC-<br>ACGCCCACGCA	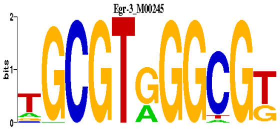
PCF2_M00948	4.2426e-05	--AGCCCACAC<br>NGGNCCCAC--	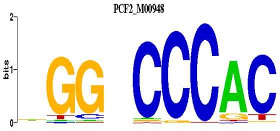

Motif13

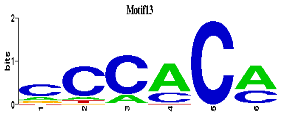 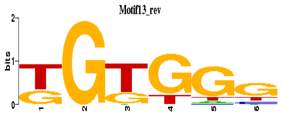

forward *reverse compliment*

Name	*E value*	*Alignment*	*Motif*
MET28	7.6540e-06	CCCACA-<br>-CCACAG	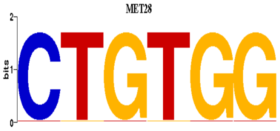
SE1PVGRP18	9.3598e-06	--------------TGTGGG----<br>ATAATGGGCCACACTGTGGGGCAT	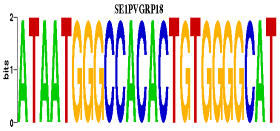
BOXCPSAS1_3	1.5985e-05	TGTGGG--<br>-GTGGGAG	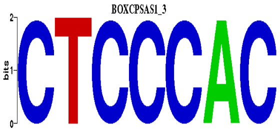
Su_H_M00234	2.4931e-05	-----CCCACA-<br>NGKTTCCCACRN	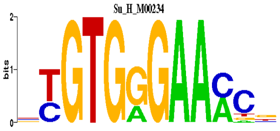

RBP-Jkappa_M01111	3.1598e-05	TGTGGG-<br>NGTGGGA	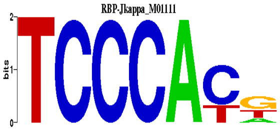

Motif12

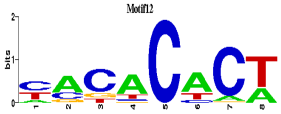

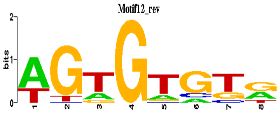

forward *reverse compliment*

Name	*E value*	*Alignment*	*Motif*
BOXCPSAS1_2	3.1708e-07	----AGTGTGTR------<br>AAGAAGTGTGTACCGGGA	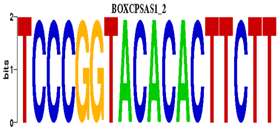
Hmx3_M00433	8.8712e-06	YACACACT-<br>CACGCACTT	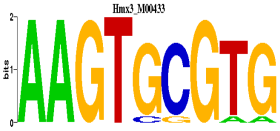
SP8BFIBSP8AIB	1.0730e-05	YACACACT<br>TACACAGT	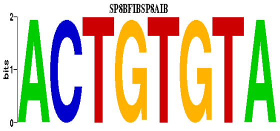
MET31	5.0179e-05	YACACACT<br>NMCACACN	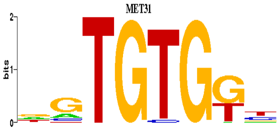
NAPINMOTIFBN	5.8885e-05	AGTGTGTR<br>-ATGTGTA	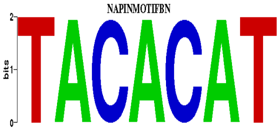

Motif14

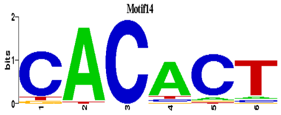

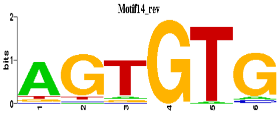

forward *reverse compliment*

Name	*E value*	*Alignment*	*Motif*

RNFG2OS	2.3832e-07	--------CACACT--<br>CCAGGGGCACACTGG	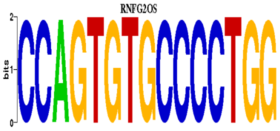
BOXCPSAS1_2	3.6365e-07	----AGTGTG--------<br>AAGAAGTGTGTACCGGGA	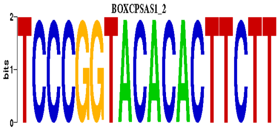
SE1PVGRP18	9.5572e-07	----------AGTGTG---------<br>ATGCCCCACAGTGTGGCCCATTAT	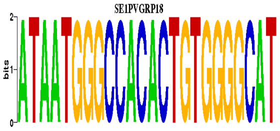
Pax-3_M00360	2.2997e-05	---AGTGTG---<br>KNAAGYGTGACG	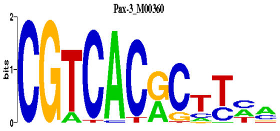
MET31	2.3063e-05	--CACACT<br>NMCACACN	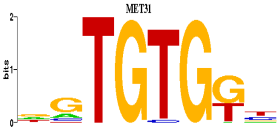

Motif7

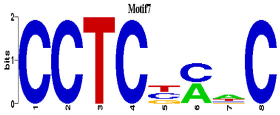

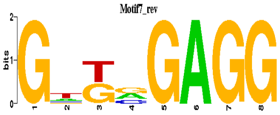

forward *reverse compliment*

Name	*E value*	*Alignment*	*Motif*
BOXCPSAS1_3	1.5564e-07	GNKNGAGG<br>GTGGGAG-	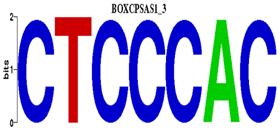
Su_H_	6.9397e-06	CCTCNMNC<br>-YTCYCAY	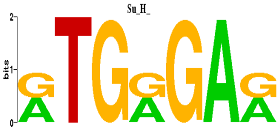
TFII-I_M00706	1.9496e-05	GNKNGAGG<br>GANGKAGG	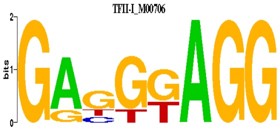
NONAMERMOTIFTAH3H4	4.3198e-05	-GNKNGAGG<br>CGTTGGATG	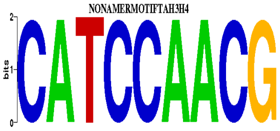

L4DCPAL1	4.7159e-05	--GNKNGAGG--<br>TGGTTGGAGATT	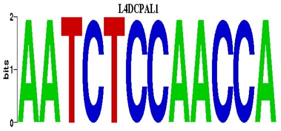

Sequence logo generation powered by weblogo
STAMP is written by Shaun Mahony
