# Supplementary material for: Promzea: a pipeline for discovery of co-regulatory motifs in maize and other plant species and its application to the anthocyanin and phlobaphene biosynthetic pathways and the Maize Development Atlas
Source: BMC Plant Biol. 2013 Mar 15;13:42. doi: 10.1186/1471-2229-13-42 (PMC3658923; doi:10.1186/1471-2229-13-42)
Supplement: Additional file 7 — Supplemental files for testing Promzea with data sets from the Maize Development Atlas. The zip folder contains 3 folders. The first contains the promoter input for Promzea for each maize tissue; the second folder has all the outputs from Promzea; the third folder contains the STAMP website outputs for comparisons of the predicted motifs with experimentally defined motifs. [file 1471-2229-13-42-S7.zip › Supplemental files 3 -Case study 3/3-Promzea similarity STAMP/STAMP-leaf.pdf]

Jump to: Multiple Alignment Motif Tree Motif Matching
Input file: 6 motifs loaded
Settings: Metric=PCC, Alignment=SWU, Gap-open=1000, Gap-extend=1000, -nooverlapalign
Multiple Alignment=IR, Tree=UPGMA, Matching against: Place
Note: All results files are removed nightly at midnight EST. Please save your results by saving "Webpage, complete".
Download results as a PDF
Click here to run STAMP again.

Multiple Alignment

(Consensus sequence representations shown, but multiple alignment was carried out on the matrices)

```
Motif1:     --TCGATCGC-
Motif2:     TYYCNTTC---
Motif3:     -NYCNMTCSMT
Motif4:     --RCGCGCGY-
Motif5:     GNTMGNTMG--
Motif6:     -TACGCG----
```

Familial Profile:
(click for matrix)

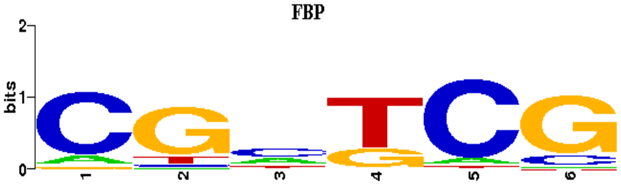

Motif Tree

Tree (drawn by Phylip) Click here for Newick-format tree (viewable with MEGA)	Input Motif	Best match in Place

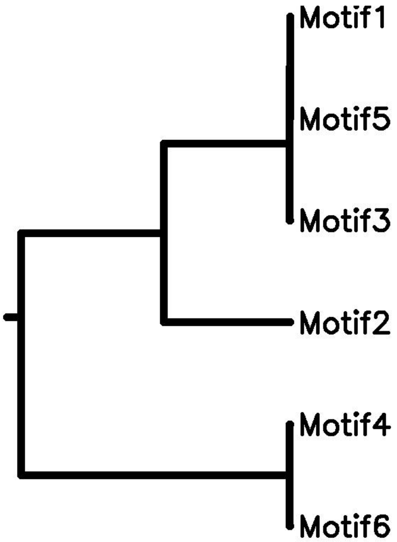

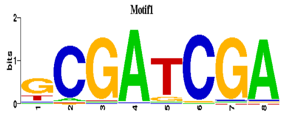 Motif1	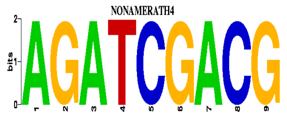 NONAMERATH4 (E val: 5.0528e-06)
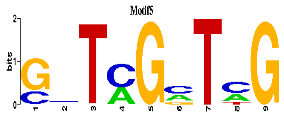 Motif5	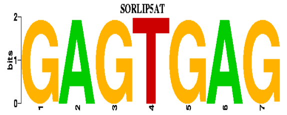 SORLIP5AT (E val: 6.2860e-04)
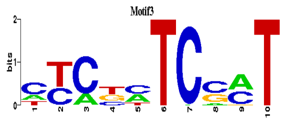 Motif3	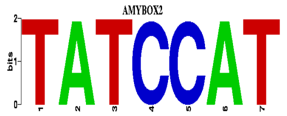 AMYBOX2 (E val: 3.6375e-05)
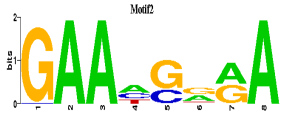 Motif2	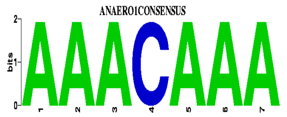 ANAERO1CONSENSUS (E val: 1.8981e-04)
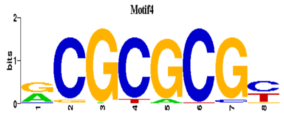 Motif4	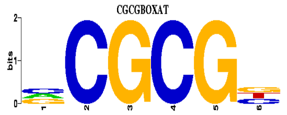 CGCGBOXAT (E val: 2.0940e-05)
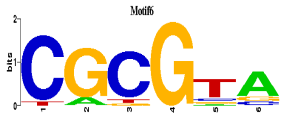 Motif6	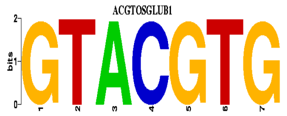 ACGTOSGLUB1 (E val: 2.6104e-05)

Motif Similarity Matches

Motif1

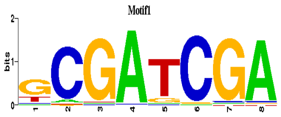

forward

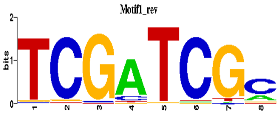

reverse compliment

Name	*E value*	*Alignment*	*Motif*

NONAMERATH4	5.0528e-06	--TCGATCGC<br>CGTCGATCT-	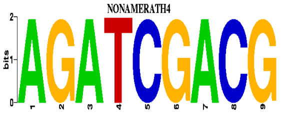
RNFG1OS	2.6129e-05	-----TCGATCGC<br>GATCATCGATC--	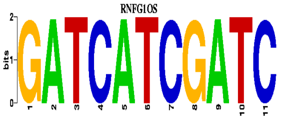
SUREAHVISO1	2.8967e-03	---TCGATCGC--------------<br>TTTTCCATCGGTCTTTCTTAGTTTT	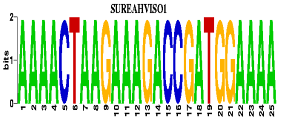
AGMOTIFNTMYB2	2.9785e-03	-TCGATCGC<br>TTGGATCT-	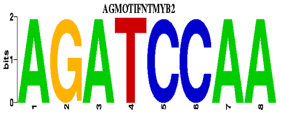
CBFHV	3.8991e-03	-TCGATCGC<br>GTCGRY---	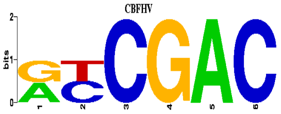

Motif5

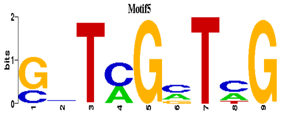

forward

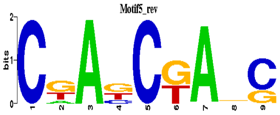

reverse compliment

Name	*E value*	*Alignment*	*Motif*
SORLIP5AT	6.2860e-04	GNTMGNTMG<br>-CTCACTC-	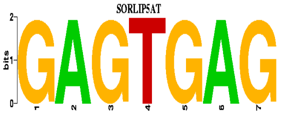
DRE1COREZMRAB17	2.8458e-03	GNTMGNTMG<br>TCTCGGT--	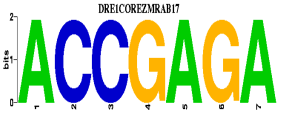
GLUTEBP2OS	3.7584e-03	---------CKANCKANC--<br>ACTTATATCTATTGAGCAT	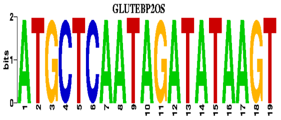
CTRMCAMV35S	5.7270e-03	GNTMGNTMG<br>TCTCTCTCT	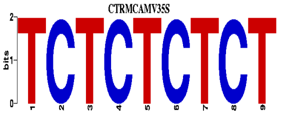

VSF1PVGRP18	5.7705e-03	CKANCKANC<br>CAACGGAGC	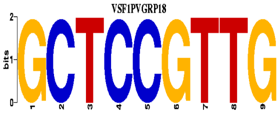

Motif3

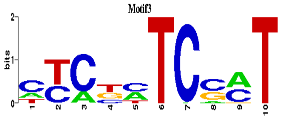

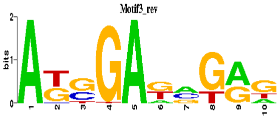

forward *reverse compliment*

Name	*E value*	*Alignment*	*Motif*
AMYBOX2	3.6375e-05	NYCNMTCSMT<br>---TATCCAT	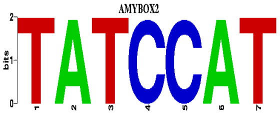
CTRMCAMV35S	1.4994e-04	AKSGAKNGRN-<br>--AGAGAGAGA	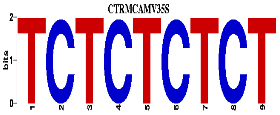
GAGA8HVBKN3	2.1663e-04	-NYCNMTCSMT-----<br>TCTCTCTCTCTCTCTC	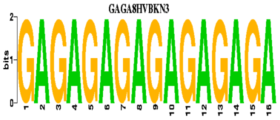
GAGAGMGSA1	3.8837e-04	-NYCNMTCSMT-------<br>TCTCTCTCTCTCTCTCTC	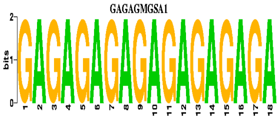
TATCCAYMOTIFOSRAMY3D	4.3726e-04	NYCNMTCSMT<br>---TATCCAY	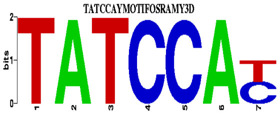

Motif2

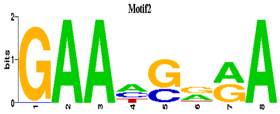

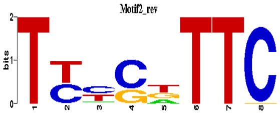

forward *reverse compliment*

Name	*E value*	*Alignment*	*Motif*

ANAERO1CONSENSUS	1.8981e-04	TYYCNTTC<br>TTTGTTT-	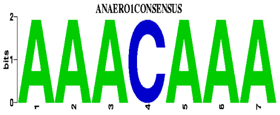
CTRMCAMV35S	4.4213e-04	-GAANGRRA<br>AGAGAGAGA	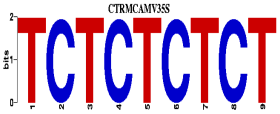
MRNA3ENDTAH3	1.1837e-03	---TYYCNTTC<br>CATTTCCATT-	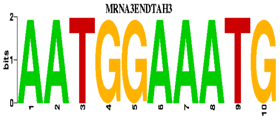
CARG2ATAP3	1.6757e-03	---------GAANGRRA--<br>TAATCCATGAAAGGTAAG	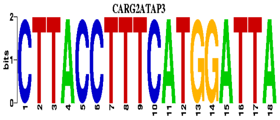
PYRIMIDINEBOXOSRAMY1A	2.4455e-03	GAANGRRA<br>AAAAGG--	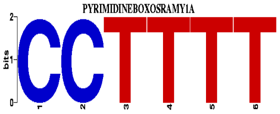

Motif4

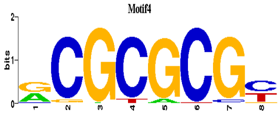

forward

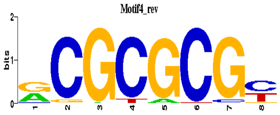

reverse compliment

Name	*E value*	*Alignment*	*Motif*
CGCGBOXAT	2.0940e-05	RCGCGCGY<br>NCGCGN--	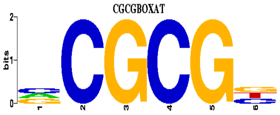
CE3OSOSEM	1.8054e-04	-RCGCGCGY-<br>GACACGCGTT	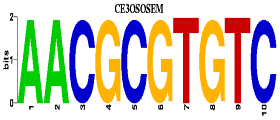
ABRECE3ZMRAB28	4.1317e-04	----RCGCGCGY<br>GAGGAGGCGCGT	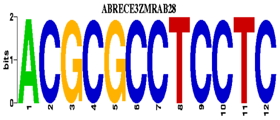
ABRECE3HVA1	5.3023e-04	----RCGCGCGY<br>GAGGACACGCGT	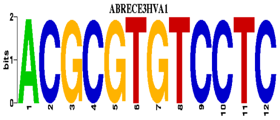

PE2FNTRNR1A	6.3815e-04	--RCGCGCGY<br>ATTCGCGC--	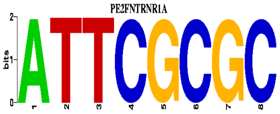

Motif6

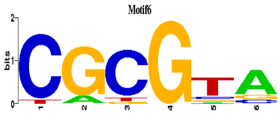

forward

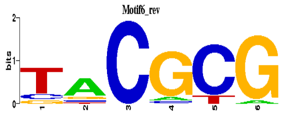

reverse compliment

Name	*E value*	*Alignment*	*Motif*
ACGTOSGLUB1	2.6104e-05	CGCGTA-<br>CACGTAC	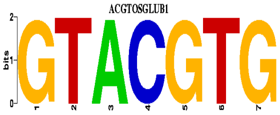
ABRERATCAL	5.2744e-05	-CGCGTA<br>NCRCGTK	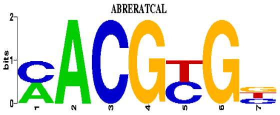
ABREMOTIFAOSOSEM	6.7415e-05	--CGCGTA<br>GACACGTA	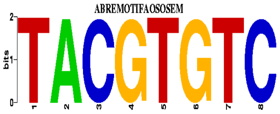
ACGTABREMOTIFAOSOSEM	6.7415e-05	--CGCGTA<br>GACACGTA	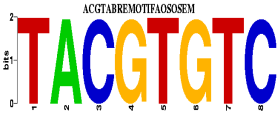
ZDNAFORMINGATCAB1	6.7415e-05	-CGCGTA-<br>ACACGTAT	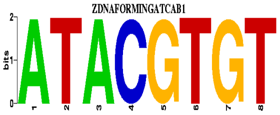

Sequence logo generation powered by weblogo
STAMP is written by Shaun Mahony
