# Supplementary material for: Promzea: a pipeline for discovery of co-regulatory motifs in maize and other plant species and its application to the anthocyanin and phlobaphene biosynthetic pathways and the Maize Development Atlas
Source: BMC Plant Biol. 2013 Mar 15;13:42. doi: 10.1186/1471-2229-13-42 (PMC3658923; doi:10.1186/1471-2229-13-42)
Supplement: Additional file 7 — Supplemental files for testing Promzea with data sets from the Maize Development Atlas. The zip folder contains 3 folders. The first contains the promoter input for Promzea for each maize tissue; the second folder has all the outputs from Promzea; the third folder contains the STAMP website outputs for comparisons of the predicted motifs with experimentally defined motifs. [file 1471-2229-13-42-S7.zip › Supplemental files 3 -Case study 3/3-Promzea similarity STAMP/STAMP-roots.pdf]

Jump to: Multiple Alignment Motif Tree Motif Matching
Input file: 11 motifs loaded
Settings: Metric=PCC, Alignment=SWU, Gap-open=1000, Gap-extend=1000, -nooverlapalign
Multiple Alignment=IR, Tree=UPGMA, Matching against: Place
Note: All results files are removed nightly at midnight EST. Please save your results by saving "Webpage, complete".
Download results as a PDF
Click here to run STAMP again.

Multiple Alignment

(Consensus sequence representations shown, but multiple alignment was carried out on the matrices)

```
Motif1:      TATATATAKM-
Motif2:      ---AGCTAGCT
Motif3:      ---AKCKAGCT
Motif4:      ----CCGATC-
Motif5:      -----CGATCG
Motif6:      ----TCGATC-
Motif7:      CYNNYCNNTC-
Motif8:      ---AGCTAGCT
Motif9:      ANNYCCNWWC-
Motif10:     -----CATGCA
Motif11:     -----CTAGCT
```

Familial Profile:
(click for matrix)

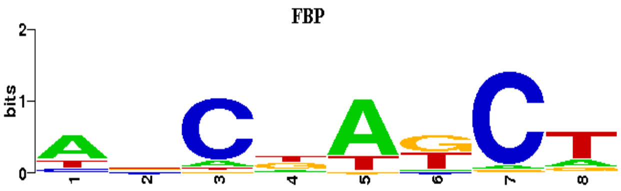

Motif Tree

Tree (drawn by Phylip)<br>Click here for Newick-format tree (viewable with MEGA)	Input Motif	Best match in Place

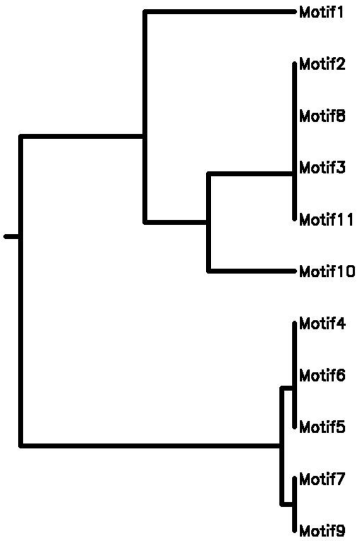

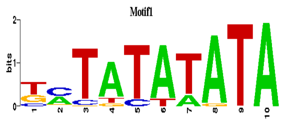 Motif1	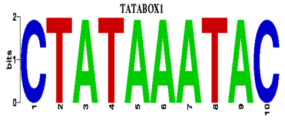 TATABOX1 (E val: 8.2269e-09)
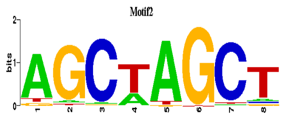 Motif2	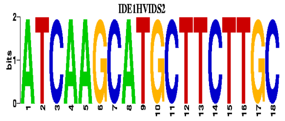 IDE1HVIDS2 (E val: 6.4178e-04)
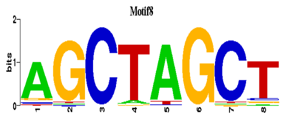 Motif8	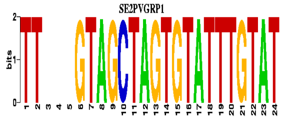 SE2PVGRP1 (E val: 6.3397e-04)
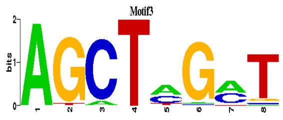 Motif3	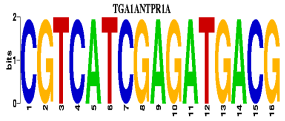 TGA1ANTPR1A (E val: 7.0148e-04)
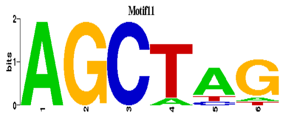 Motif11	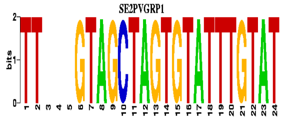 SE2PVGRP1 (E val: 1.2458e-06)
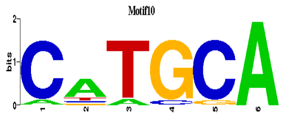 Motif10	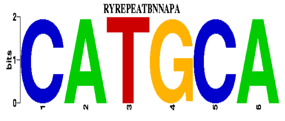 RYREPEATBNNAPA (E val: 5.0828e-11)
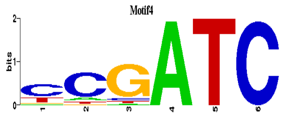 Motif4	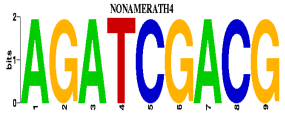 NONAMERATH4 (E val: 3.9641e-05)
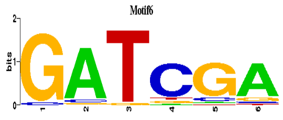 Motif6	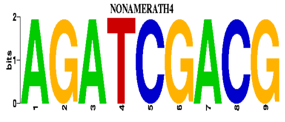 NONAMERATH4 (E val: 1.2995e-08)

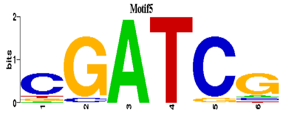 Motif5	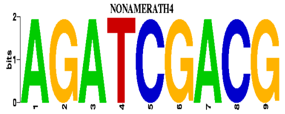 NONAMERATH4 (E val: 4.1159e-05)
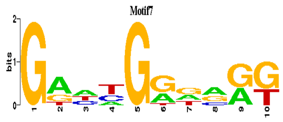 Motif7	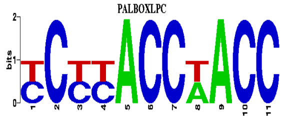 PALBOXLPC (E val: 3.0685e-05)
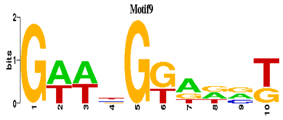 Motif9	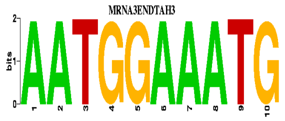 MRNA3ENDTAH3 (E val: 1.0627e-05)

Motif Similarity Matches

Motif1

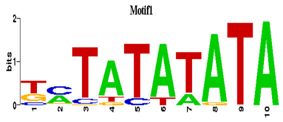

forward

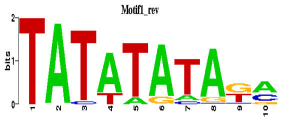

reverse compliment

Name	*E value*	*Alignment*	*Motif*
TATABOX1	8.2269e-09	-TATATATAKM<br>GTATTTATAG-	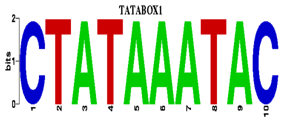
SORLREP3AT	2.1701e-08	KMTATATATA<br>TGTATATAT-	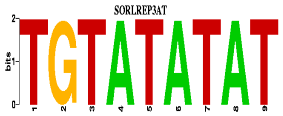
TATAPVTRNALEU	8.0272e-08	KMTATATATA<br>--TTTATATA	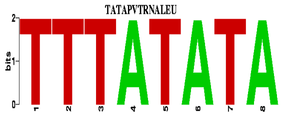
D2GMAUX28	1.3701e-06	---KMTATATATA<br>ATTTATATAAAT-	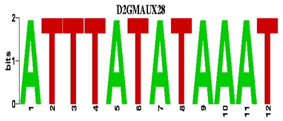

TATABOX4	1.9373e-06	KMTATATATA<br>---TTATATA	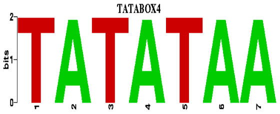

Motif2

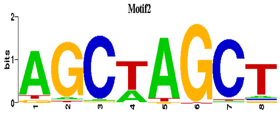

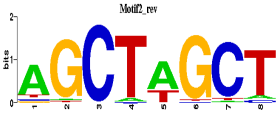

forward *reverse compliment*

Name	*E value*	*Alignment*	*Motif*
IDE1HVIDS2	6.4178e-04	------AGCTAGCT----<br>GCAAGAAGCATGCTTGAT	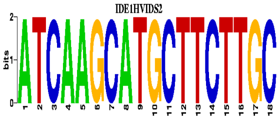
SE2PVGRP1	1.0105e-03	----------AGCTAGCT-------<br>ATACAAATACACTAGCTACNNNNAA	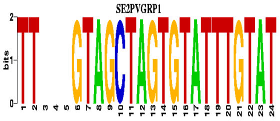
SITE3SORPS1	3.8352e-03	------AGCTAGCT<br>TCTTTTAACTAACT	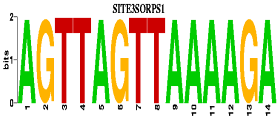
MYB1LEPR	7.4216e-03	AGCTAGCT<br>AACTAAC-	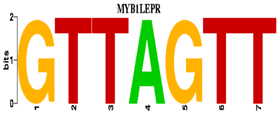
ALF1NTPARC	1.3151e-02	------AGCTAGCT---<br>TGTCATTGCTTGCGTAA	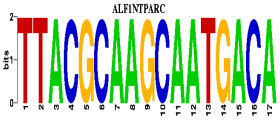

Motif8

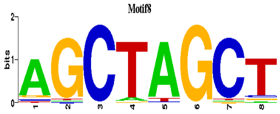

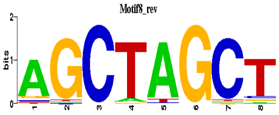

forward *reverse compliment*

Name	*E value*	*Alignment*	*Motif*

SE2PVGRP1	6.3397e-04	----------AGCTAGCT-------<br>ATACAAATACACTAGCTACNNNAA	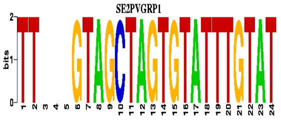
SITE3SORPS1	2.6621e-03	------AGCTAGCT<br>TCTTTTAACTAACT	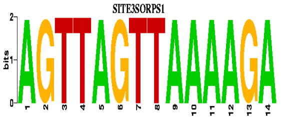
IDE1HVIDS2	3.3667e-03	------AGCTAGCT----<br>GCAAGAAGCATGCTTGAT	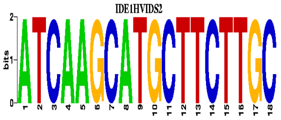
MYB1LEPR	5.1564e-03	AGCTAGCT<br>AACTAAC-	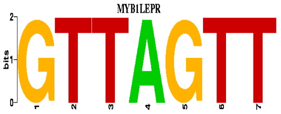
MYB26PS	1.3558e-02	-AGCTAGCT<br>AACCTAAC-	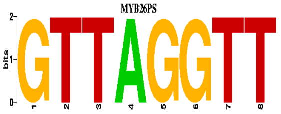

Motif3

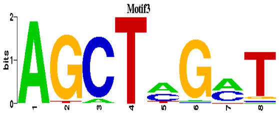

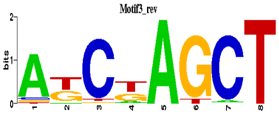

forward *reverse compliment*

Name	*E value*	*Alignment*	*Motif*
TGA1ANTPR1A	7.0148e-04	----AKCKAGCT----<br>CGTCATCGAGATGACG	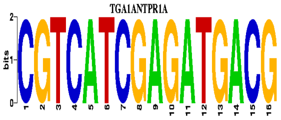
SE2PVGRP1	2.2985e-03	----------AKCKAGCT-------<br>ATACAAATACACTAGCTACNNNAA	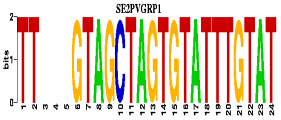
NONAMERATH4	4.2383e-03	-AKCKAGCT<br>CGTCGATCT	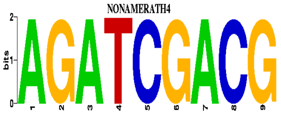
GLUTEBOX1OSGT3	4.9552e-03	-----------AGCTMGMT-<br>TGAAGTGACTCACTAGATA	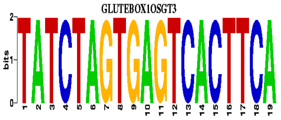

IDE1HVIDS2	6.4103e-03	----------AGCTMGMT<br>GCAAGAAGCATGCTTGAT	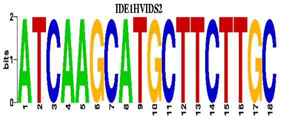

Motif11

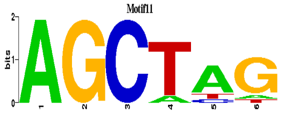

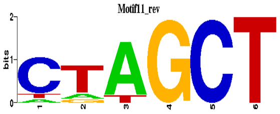

forward *reverse compliment*

Name	*E value*	*Alignment*	*Motif*
SE2PVGRP1	1.2458e-06	------------CTAGCT-------<br>ATACAAATACACTAGCTACNNNAA	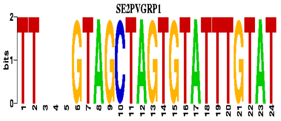
20NTNTNOS	9.2060e-04	-------------CTAGCT--<br>TGACGTATGTGCTTAGCTCA	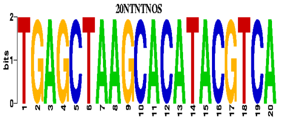
ASF1ATNOS	9.9922e-04	--------------CTAGCT--<br>CTGACGTATGTGCTTAGCTCA	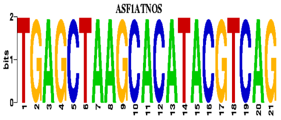
SITE3SORPS1	2.1706e-03	---------CTAGCT<br>TCTTTTAACTAACT	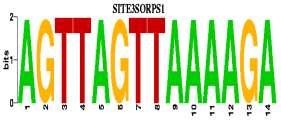
GLUTEBOX1OSGT3	4.2972e-03	-------------CTAGCT-<br>TGAAGTGACTCACTAGATA	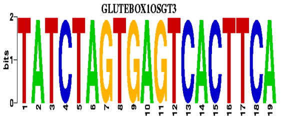

Motif10

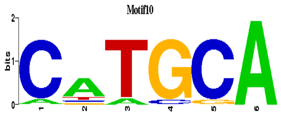

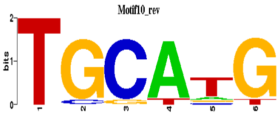

forward *reverse compliment*

Name *E value* *Alignment* *Motif*

RYREPEATBNNAPA	5.0828e-11	CATGCA<br>CATGCA	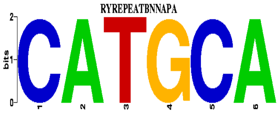
RYREPEATGMGY2	1.0535e-09	CATGCA-<br>CATGCAT	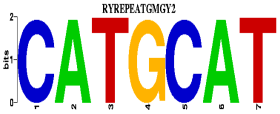
RYREPEATLEGUMINBOX	1.0535e-09	CATGCA-<br>CATGCAY	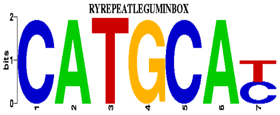
RYREPEATVFLEB4	4.5886e-09	CATGCA--<br>CATGCATG	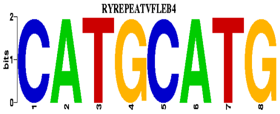
SPHCOREZMC1	1.4658e-08	--CATGCA-<br>TCCATGCAT	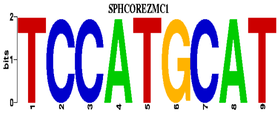

Motif4

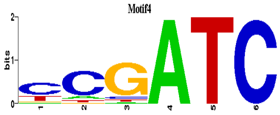

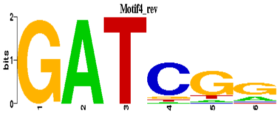

forward *reverse compliment*

Name	*E value*	*Alignment*	*Motif*
NONAMERATH4	3.9641e-05	--CCGATC-<br>CGTCGATCT	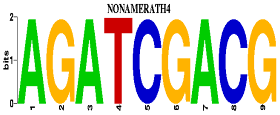
RNFG1OS	1.1067e-04	GATCGG-----<br>GATCGATGATC	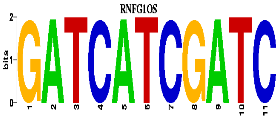
OCTAMERMOTIFTAH3H4	3.3704e-04	GATCGG--<br>GATCCGCG	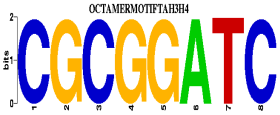
CAATBOX2	4.9243e-04	-GATCGG--<br>AGATTGGCC	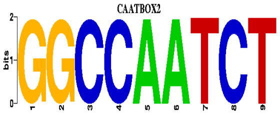

LTRECOREATCOR15	5.1924e-04	GATCGG<br>-GTCGG	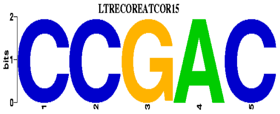

Motif6

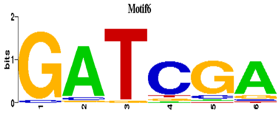

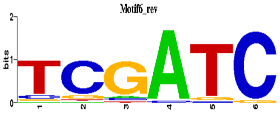

forward *reverse compliment*

Name	*E value*	*Alignment*	*Motif*
NONAMERATH4	1.2995e-08	--TCGATC-<br>CGTCGATCT	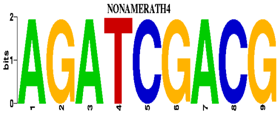
RNFG1OS	5.3998e-08	GATCGA-----<br>GATCGATGATC	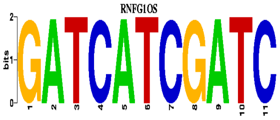
AGMOTIFNTMYB2	1.1033e-04	-TCGATC-<br>TTGGATCT	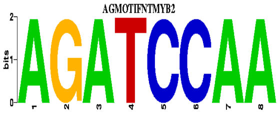
TGA1ANTPR1A	3.8891e-04	-------TCGATC---<br>CGTCATCTCGATGACG	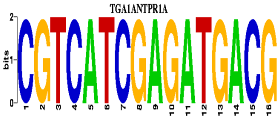
CBFHV	4.9043e-04	-TCGATC<br>GTCGRY-	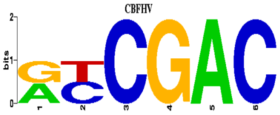

Motif5

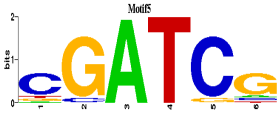

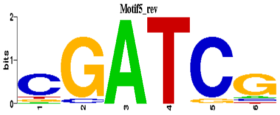

forward *reverse compliment*

Name	*E value*	*Alignment*	*Motif*

NONAMERATH4	4.1159e-05	---CGATCG<br>CGTCGATCT	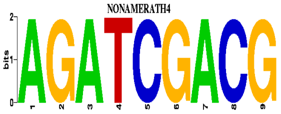
RNFG1OS	1.1466e-04	CGATCG------<br>-GATCGATGATC	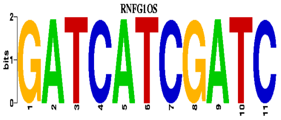
OCETYPEINTHISTONE	5.3907e-04	---CGATCG---------<br>CGCGGATCGNTGACGTGG	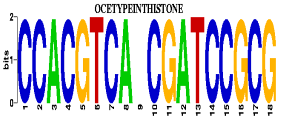
SUREAHVISO1	4.3863e-03	----CGATCG---------------<br>TTTTCCATCGGTCTTTCTTAGTTTT	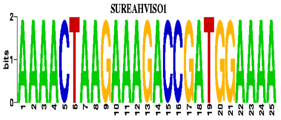
AGMOTIFNTMYB2	6.5792e-03	--CGATCG<br>TTGGATCT	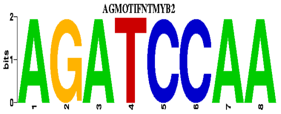

Motif7

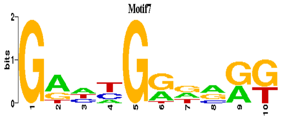

forward

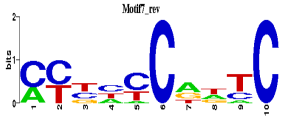

reverse compliment

Name	*E value*	*Alignment*	*Motif*
PALBOXLPC	3.0685e-05	GANNGRNNRG-<br>GGTWGGTRRGR	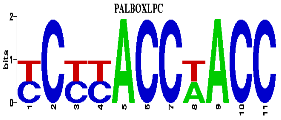
ARELIKEGHPGDFR2	3.5776e-05	----GANNGRNNRG---<br>AGTTGAATGGGGGTGCA	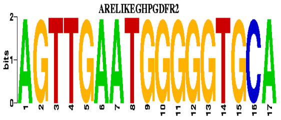
MRNA3ENDTAH3	6.4136e-04	GANNGRNNRG-<br>-AATGGAAATG	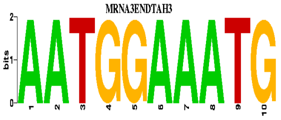
CARG3ATAP3	1.5953e-03	---------GANNGRNNRG<br>GTTACTAAAAATGGAAAG	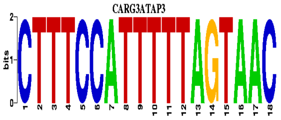

CARG2ATAP3	1.6099e-03	--------GANNGRNNRG<br>TAATCCATGAAAGGTAAG	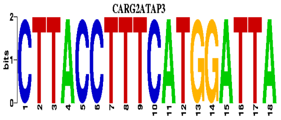

Motif9

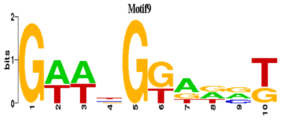

forward

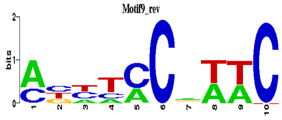

reverse compliment

Name	*E value*	*Alignment*	*Motif*
MRNA3ENDTAH3	1.0627e-05	-ANNYCCNWWC<br>CATTTCCATT-	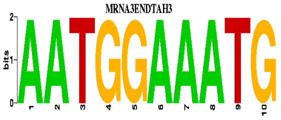
ARELIKEGHPGDFR2	2.9076e-04	----GWWNGGRNNT---<br>AGTTGAATGGGGGTGCA	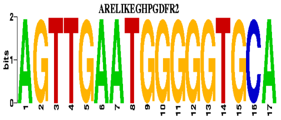
L4DCPAL1	6.2965e-04	-ANNYCCNWWC-<br>AATCTCCAACCA	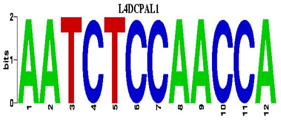
SBOXATRBCS	1.0283e-03	GWWNGGRNNT-<br>---TGGAGGTG	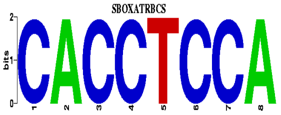
AGMOTIFNTMYB2	3.6599e-03	GWWNGGRNNT<br>--TTGGATCT	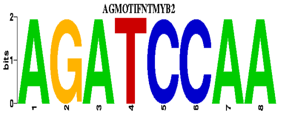

Sequence logo generation powered by weblogo
STAMP is written by Shaun Mahony
