# Supplementary material for: Promzea: a pipeline for discovery of co-regulatory motifs in maize and other plant species and its application to the anthocyanin and phlobaphene biosynthetic pathways and the Maize Development Atlas
Source: BMC Plant Biol. 2013 Mar 15;13:42. doi: 10.1186/1471-2229-13-42 (PMC3658923; doi:10.1186/1471-2229-13-42)
Supplement: Additional file 7 — Supplemental files for testing Promzea with data sets from the Maize Development Atlas. The zip folder contains 3 folders. The first contains the promoter input for Promzea for each maize tissue; the second folder has all the outputs from Promzea; the third folder contains the STAMP website outputs for comparisons of the predicted motifs with experimentally defined motifs. [file 1471-2229-13-42-S7.zip › Supplemental files 3 -Case study 3/3-Promzea similarity STAMP/STAMP-silk.pdf]

Jump to: Multiple Alignment Motif Tree Motif Matching
Input file: 10 motifs loaded
Settings: Metric=PCC, Alignment=SWU, Gap-open=1000, Gap-extend=1000, -nooverlapalign
Multiple Alignment=IR, Tree=UPGMA, Matching against: Place
Note: All results files are removed nightly at midnight EST. Please save your results by saving "Webpage, complete".
Download results as a PDF
Click here to run STAMP again.

Multiple Alignment

(Consensus sequence representations shown, but multiple alignment was carried out on the matrices)

```
Motif1:     --ANATAAAAMA---
Motif2:     --CGATCGAGN----
Motif3:     --GGMACGCGMS---
Motif4:     -----TSGCCNCNGC
Motif5:     --CGATCGAG-----
Motif6:     -CCCATCNC------
Motif7:     CNANATAWNA-----
Motif8:     ----ATCGCA-----
Motif9:     --CGANCGNG-----
Motif10:    --CGATCGC------
```

Familial Profile:
(click for matrix)

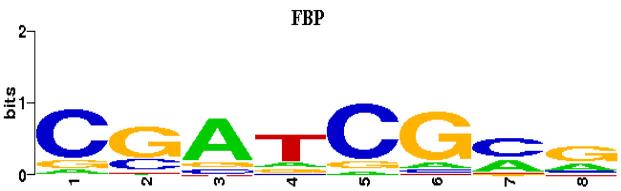

Motif Tree

**Tree (drawn by Phylip)**<br>Click here for Newick-format tree (viewable with MEGA)	**Input Motif**	**Best match in Place**
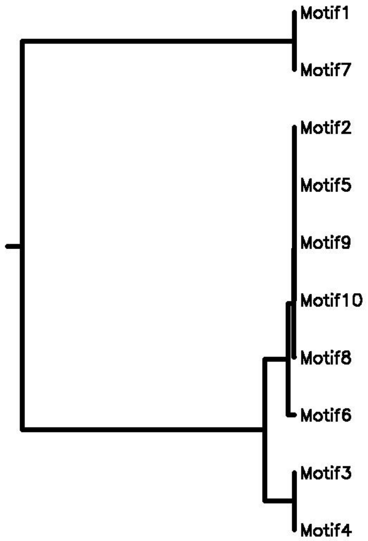	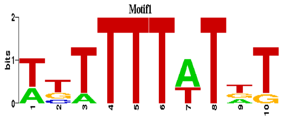 Motif1	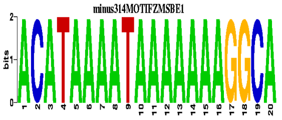 minus314MOTIFZMSBE1 (E val: 2.1430e-11)
	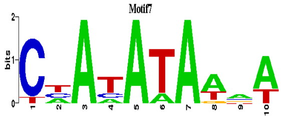 Motif7	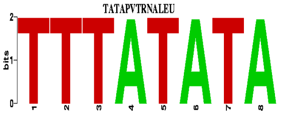 TATAPVTRNALEU (E val: 4.8757e-10)
	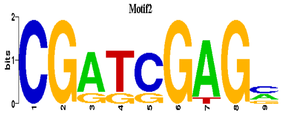 Motif2	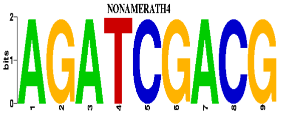 NONAMERATH4 (E val: 5.2219e-05)
	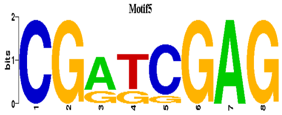 Motif5	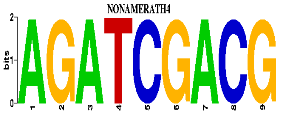 NONAMERATH4 (E val: 1.5766e-05)
	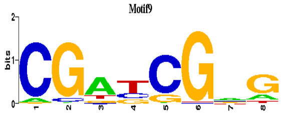 Motif9	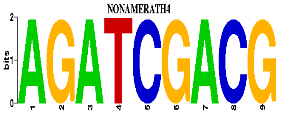 NONAMERATH4 (E val: 2.3781e-05)
	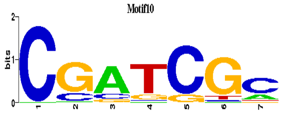 Motif10	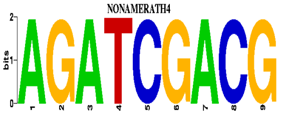 NONAMERATH4 (E val: 6.0460e-04)
	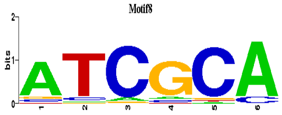 Motif8	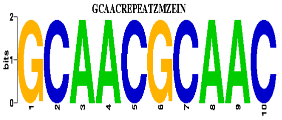 GCAACREPEATZMZEIN (E val: 6.4631e-04)
	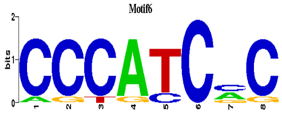 Motif6	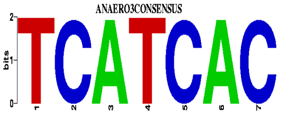 ANAERO3CONSENSUS (E val: 7.8838e-06)

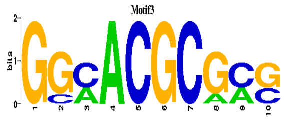 Motif3	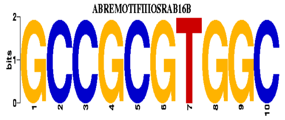 ABREMOTIFIIIOSRAB16B (E val: 4.9670e-06)
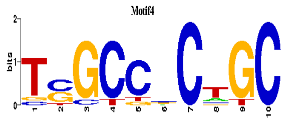 Motif4	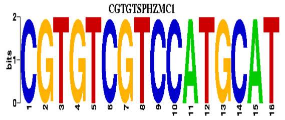 CGTGTSPHZMC1 (E val: 5.7937e-04)

Motif Similarity Matches

Motif1

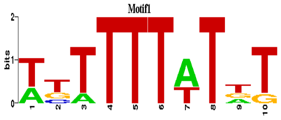

forward

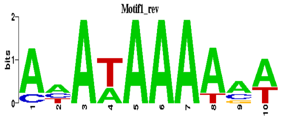

reverse compliment

Name	*E value*	*Alignment*	*Motif*
minus314MOTIFZMSBE1	2.1430e-11	-----ANATAAAAMA-----<br>ACATAAAATAAAAAAAGGCA	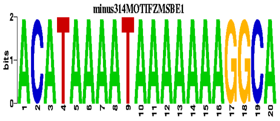
COREOS	9.3008e-09	---------TKTTTTATNT----------<br>TAWATAAAAKTTTTATWTAYRWATTMTT	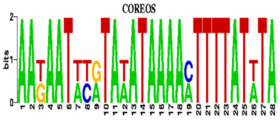
MARTBOX	9.9009e-09	TKTTTTATNT<br>TTWTWTTWTT	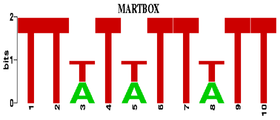
3AF1BOXPSRBCS3	1.7256e-08	---------ANATAAAAMA---<br>AAATAGATAAATAAAAACATT	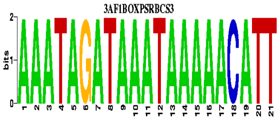
ELEMENT2GMLBC3	2.6032e-08	ANATAAAAMA------<br>AAATAAATAATTTAAG	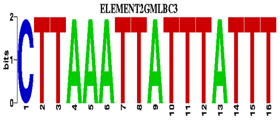

Motif7

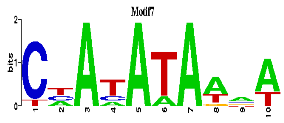

forward

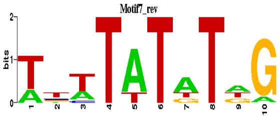

reverse compliment

Name	*E value*	*Alignment*	*Motif*
TATAPVTRNALEU	4.8757e-10	CNANATAWNA<br>-TATATAAA-	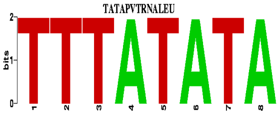
MARARS	4.1128e-08	-CNANATAWNA<br>WAAAYATAAAW	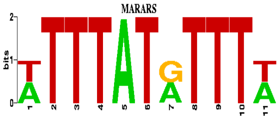
TATABOX4	7.2809e-08	CNANATAWNA<br>-TATATAA--	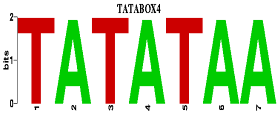
D2GMAUX28	3.3979e-07	--CNANATAWNA<br>ATTTATATAAAT	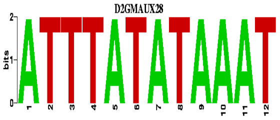
COREOS	5.0374e-07	CNANATAWNA-------------------<br>-TAWATAAAAKTTTTATWTAYRWATTMTT	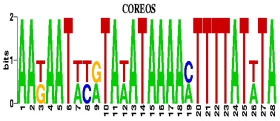

Motif2

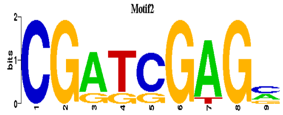

forward

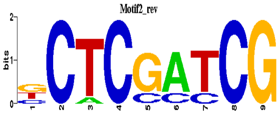

reverse compliment

Name	*E value*	*Alignment*	*Motif*
NONAMERATH4	5.2219e-05	NCTCGATCG<br>CGTCGATCT	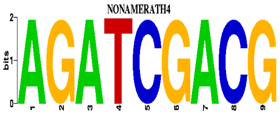
RNFG1OS	2.9487e-04	CGATCGAGN---<br>-GATCGATGATC	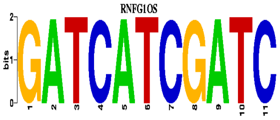

TGA1ANTPR1A	5.0008e-04	-----NCTCGATCG--<br>CGTCATCTCGATGACG	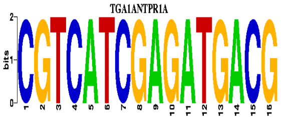
DRE1COREZMRAB17	1.4258e-03	NCTCGATCG<br>TCTCGGT--	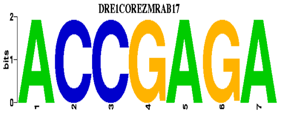
GLUTEBP2OS	4.2162e-03	--NCTCGATCG--------<br>ATGCTCAATAGATATAAGT	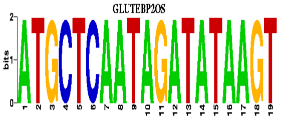

Motif5

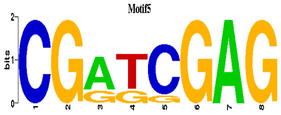

forward

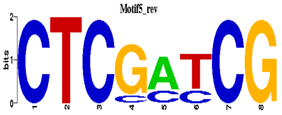

reverse compliment

Name	*E value*	*Alignment*	*Motif*
NONAMERATH4	1.5766e-05	-CTCGATCG<br>CGTCGATCT	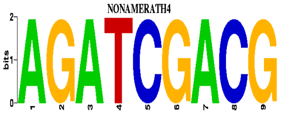
RNFG1OS	7.3344e-05	CGATCGAG----<br>-GATCGATGATC	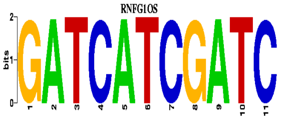
TGA1ANTPR1A	4.2493e-04	------CTCGATCG--<br>CGTCATCTCGATGACG	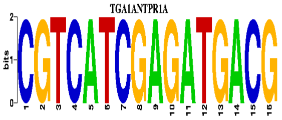
CYTOSITECSHPRA	1.3078e-03	CTCGATCG-----<br>CTCAATCAATCTT	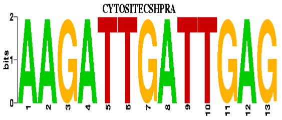
SORLIP5AT	1.6225e-03	CTCGATCG<br>CTCACTC-	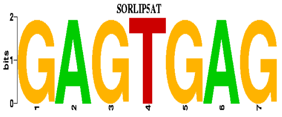

Motif9

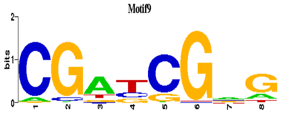

forward

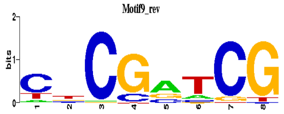

reverse compliment

Name	*E value*	*Alignment*	*Motif*
NONAMERATH4	2.3781e-05	`CGANCGNG-`<br>`AGATCGACG`	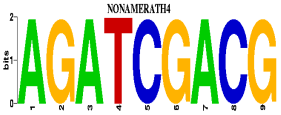
RNFG1OS	1.0639e-04	`CGANCGNG----`<br>`-GATCGATGATC`	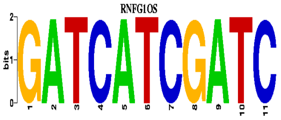
DRE1COREZMRAB17	4.4496e-04	`-CNCGNTCG`<br>`TCTCGGT--`	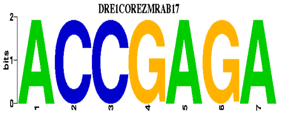
TGA1ANTPR1A	6.8906e-04	`-------CNCGNTCG--`<br>`CGTCATCTCGATGACG`	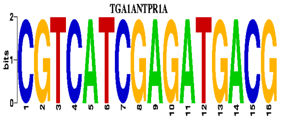
CBFHV	1.9541e-03	`CNCGNTCG`<br>`GTCGRY--`	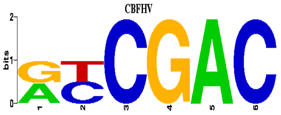

Motif10

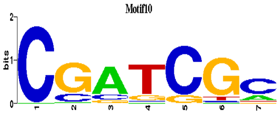

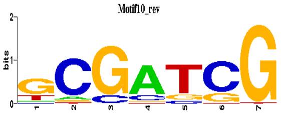

forward *reverse compliment*

Name	*E value*	*Alignment*	*Motif*
NONAMERATH4	6.0460e-04	`---CGATCGC`<br>`CGTCGATCT-`	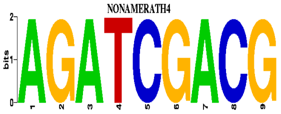
RNFG1OS	1.6125e-03	`GCGATCG------`<br>`--GATCGATGATC`	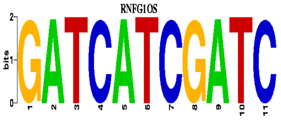

PIATGAPB	2.1574e-03	-CGATCGC<br>GTGATCAC	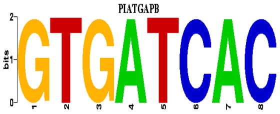
ABRECE1HVA22	5.2269e-03	-CGATCGC-<br>CCGGTGGCA	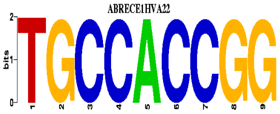
OCETYPEINTHISTONE	7.1427e-03	--GCGATCG---------<br>CGCGGATCGNTGACGTGG	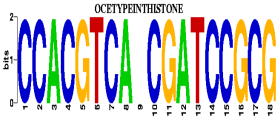

Motif8

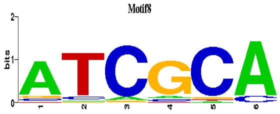

forward

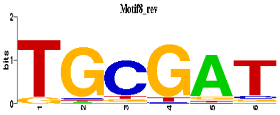

reverse compliment

Name	*E value*	*Alignment*	*Motif*
GCAACREPEATZMZEIN	6.4631e-04	--TGCGAT--<br>GTTGCGTTGC	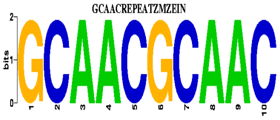
RBCSGBOXPS	8.5525e-04	--TGCGAT---<br>AGTGCCATGTG	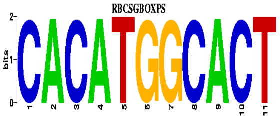
BOX2PVCHS15	1.6699e-03	-------ATCGCA-<br>ATATTTAATCACAG	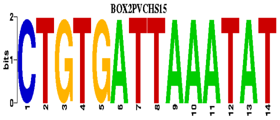
SRENTTTO1	1.9279e-03	-------TGCGAT<br>TGGTAGGTGAGAT	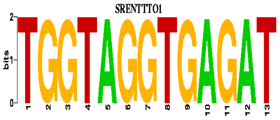
ANAERO4CONSENSUS	2.2266e-03	-TGCGAT--<br>TTGCNAAAC	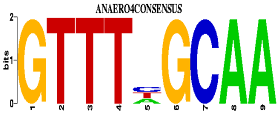

Motif6

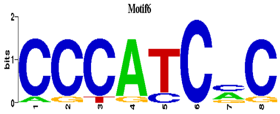

forward

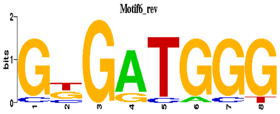

reverse compliment

Name	*E value*	*Alignment*	*Motif*
ANAERO3CONSENSUS	7.8838e-06	GNGATGGG<br>GTGATGA-	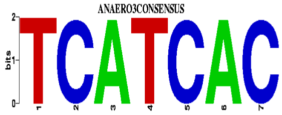
PE3ASPHYA3	4.4483e-05	----------------CCCATCNC-------<br>CAGCTCCCATGGCTCTCCCATCCGCGCCGGT	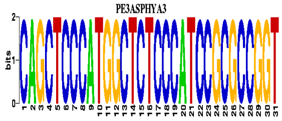
SORLIP4AT	1.2796e-04	CCCATCNC--<br>-CCATCATAC	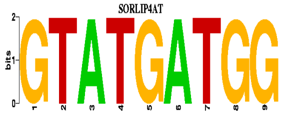
AUXREPSIAA4	8.1333e-04	GNGATGGG---<br>---ATGGGACM	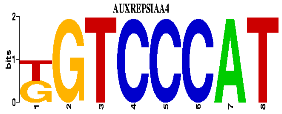
GGTCCCATGMSAUR	8.1333e-04	GNGATGGG---<br>---ATGGGACC	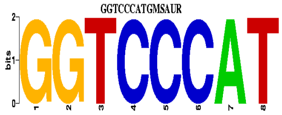

Motif3

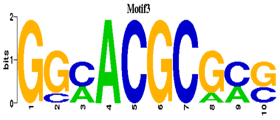

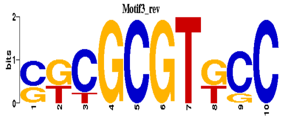

forward *reverse compliment*

Name	*E value*	*Alignment*	*Motif*
ABREMOTIFIIIOSRAB16B	4.9670e-06	GGMACGCGMS<br>GCCACGCGGC	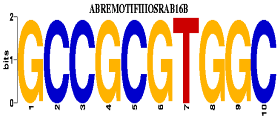
ABRE2HVA22	1.1005e-05	GGMACGCGMS<br>GACACGTGCG	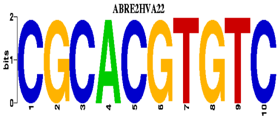

ABADESI2	1.5548e-05	GGMACGCGMS-<br>GCCACGCGTCC	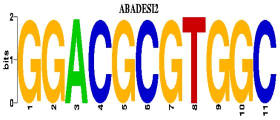
GBOX10NT	1.9017e-05	SKCGCGTKCC<br>GGCACGTGGC	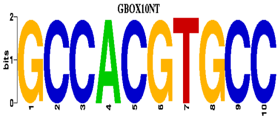
ABRERATCAL	3.4808e-05	SKCGCGTKCC<br>-NCRCGTK--	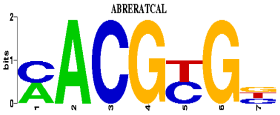

Motif4

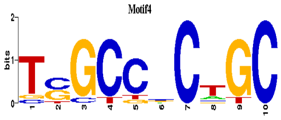

forward

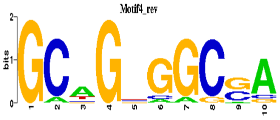

reverse compliment

Name	*E value*	*Alignment*	*Motif*
CGTGTSPHZMC1	5.7937e-04	--GCNGNGGCSA----<br>ATGCATGGACGACACG	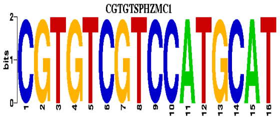
SORLIP1AT	7.0656e-04	GCNGNGGCSA<br>---GTGGC--	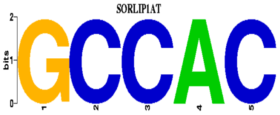
RNFG2OS	8.7676e-04	-----TSGCCNCNGC<br>CCAGTGTGCCCCTGG	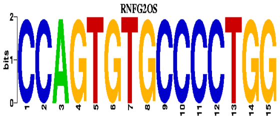
GBOXRELOSAMY3	1.6822e-03	TSGCCNCNGC-<br>TGGCCACGTAG	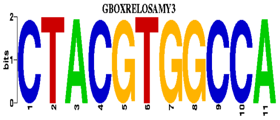
AMMORESVDCRNIA1	2.1110e-03	TSGCCNCNGC<br>-GGCCCCGGG	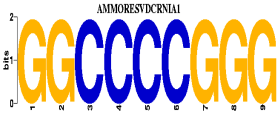

Sequence logo generation powered by weblogo
STAMP is written by Shaun Mahony
