# Supplementary material for: Promzea: a pipeline for discovery of co-regulatory motifs in maize and other plant species and its application to the anthocyanin and phlobaphene biosynthetic pathways and the Maize Development Atlas
Source: BMC Plant Biol. 2013 Mar 15;13:42. doi: 10.1186/1471-2229-13-42 (PMC3658923; doi:10.1186/1471-2229-13-42)
Supplement: Additional file 7 — Supplemental files for testing Promzea with data sets from the Maize Development Atlas. The zip folder contains 3 folders. The first contains the promoter input for Promzea for each maize tissue; the second folder has all the outputs from Promzea; the third folder contains the STAMP website outputs for comparisons of the predicted motifs with experimentally defined motifs. [file 1471-2229-13-42-S7.zip › Supplemental files 3 -Case study 3/3-Promzea similarity STAMP/STAMP-tassel.pdf]

Jump to: Multiple Alignment Motif Tree Motif Matching
Input file: 11 motifs loaded
Settings: Metric=PCC, Alignment=SWU, Gap-open=1000, Gap-extend=1000, -nooverlapalign
Multiple Alignment=IR, Tree=UPGMA, Matching against: ALL
Note: All results files are removed nightly at midnight EST. Please save your results by saving "Webpage, complete".
Download results as a PDF
Click here to run STAMP again.

Multiple Alignment

(Consensus sequence representations shown, but multiple alignment was carried out on the matrices)

Motif1:	--GCRTGCAC-
Motif2:	--CCNWCWNCT
Motif3:	---CRTGCA--
Motif4:	-KGCATG----
Motif5:	NNGARWNGGW-
Motif6:	--GCGTGNGC-
Motif7:	-KGCATGCA--
Motif8:	-TGCRTG----
Motif9:	--GCGTGA---
Motif10:	---CGYGMGCG
Motif11:	-TKAGWTGGA-

Familial Profile:
(click for matrix)

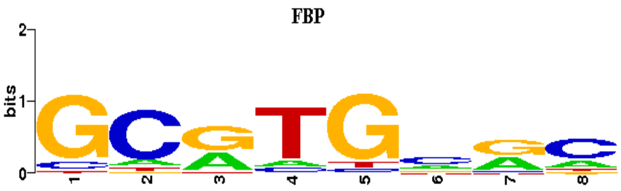

Motif Tree

Tree (drawn by Phylip)<br>Click here for Newick-format tree (viewable with MEGA)	Input Motif	Best match in ALL

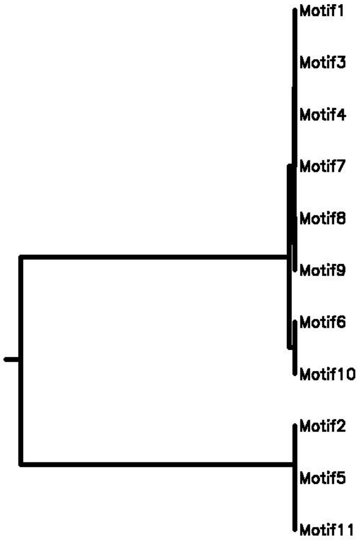

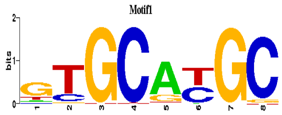 Motif1	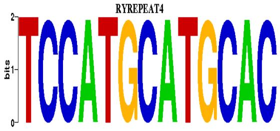 RYREPEAT4 (E val: 7.8027e-10)
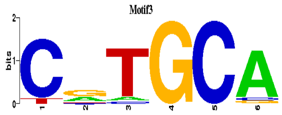 Motif3	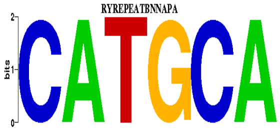 RYREPEATBNNAPA (E val: 1.0924e-07)
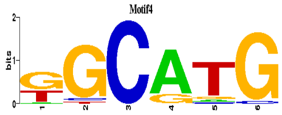 Motif4	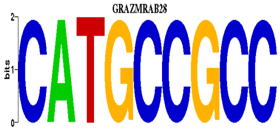 GRAZMRAB28 (E val: 8.9886e-08)
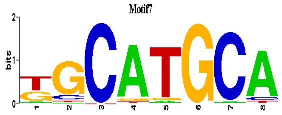 Motif7	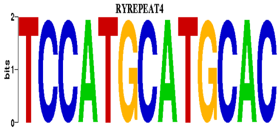 RYREPEAT4 (E val: 1.0907e-10)
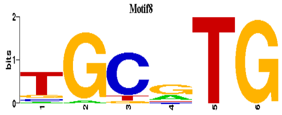 Motif8	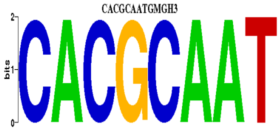 CACGCAATGMGH3 (E val: 9.9774e-09)
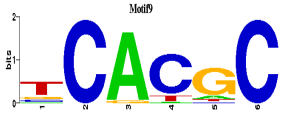 Motif9	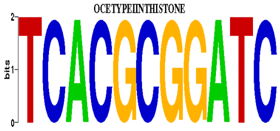 OCETYPEIINTHISTONE (E val: 5.9029e-08)
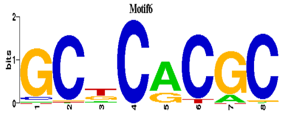 Motif6	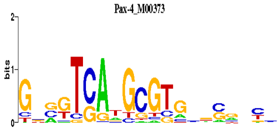 Pax-4_M00373 (E val: 5.5530e-07)
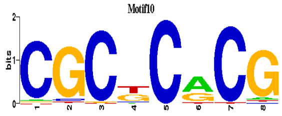 Motif10	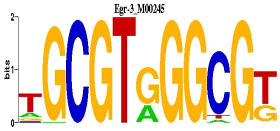 Egr-3_M00245 (E val: 6.9707e-06)

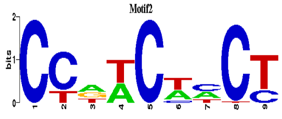 Motif2	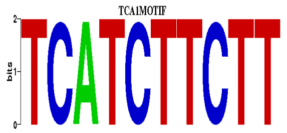 TCA1MOTIF (E val: 2.9665e-04)
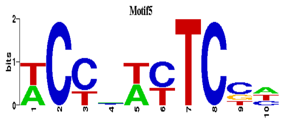 Motif5	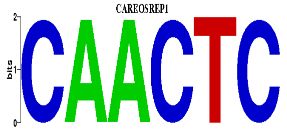 CAREOSREP1 (E val: 8.9509e-05)
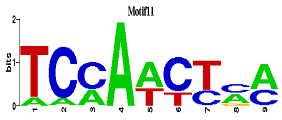 Motif11	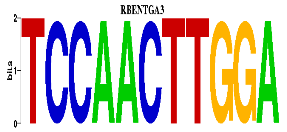 RBENTGA3 (E val: 2.4345e-05)

Motif Similarity Matches

Motif1

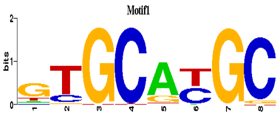

forward

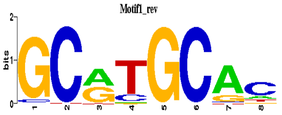

reverse compliment

Name	*E value*	*Alignment*	*Motif*
RYREPEAT4	7.8027e-10	-----GCRTGCAC<br>TCCATGCATGCAC	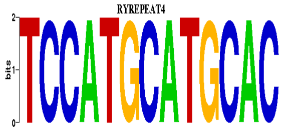
Nrf-1_M00652	2.1611e-08	-GTGCAYGC<br>YGCGCAYGC	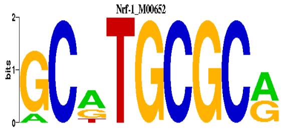
RYREPEATLEGUMINBOX	5.3966e-08	GCRTGCAC<br>-CATGCAY	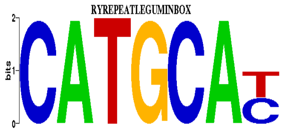
RYREPEATBNNAPA	2.1809e-07	GCRTGCAC<br>-CATGCA-	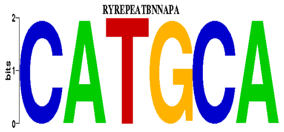

ABI3	5.5693e-07	GCRTGCAC--<br>GCATGCATNN	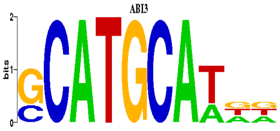

Motif3

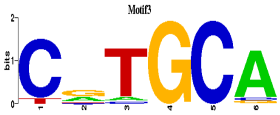

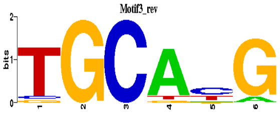

forward *reverse compliment*

Name	*E value*	*Alignment*	*Motif*
RYREPEATBNNAPA	1.0924e-07	TGCAYG<br>TGCATG	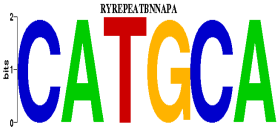
RYREPEATGMGY2	9.8880e-07	-TGCAYG<br>ATGCATG	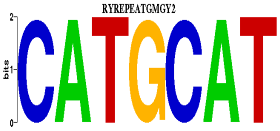
RYREPEATLEGUMINBOX	9.8880e-07	-TGCAYG<br>RTGCATG	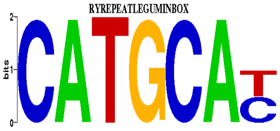
RY-repeat	3.0658e-06	CRTGCA--<br>CATGCATG	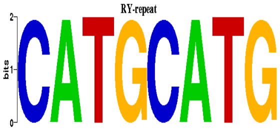
RYREPEATVFLEB4	3.0658e-06	CRTGCA--<br>CATGCATG	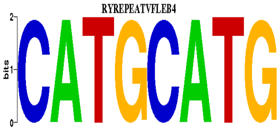

Motif4

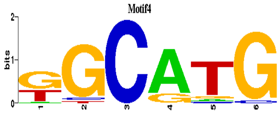

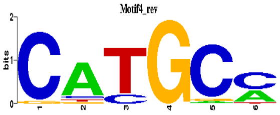

forward *reverse compliment*

Name	*E value*	*Alignment*	*Motif*

GRAZMRAB28	8.9886e-08	CATGCM---<br>CATGCCGCC	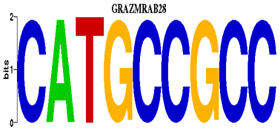
RYREPEATBNNAPA	9.3189e-08	CATGCM<br>CATGCA	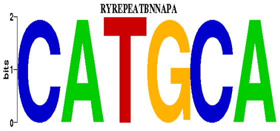
RYREPEATGMGY2	8.5857e-07	CATGCM-<br>CATGCAT	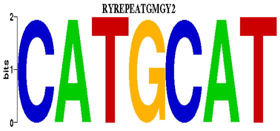
RYREPEATLEGUMINBOX	8.5857e-07	CATGCM-<br>CATGCAY	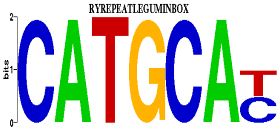
p53_M00034	2.4956e-06	-----------KGCATG---<br>GACATGCCCGGGCATGTCY	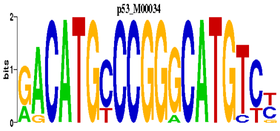

Motif7

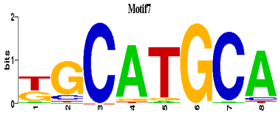

forward

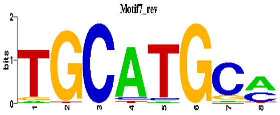

reverse compliment

Name	*E value*	*Alignment*	*Motif*
RYREPEAT4	1.0907e-10	-KGCATGCA----<br>GTGCATGCATGGA	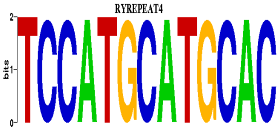
RYREPEATBNNAPA	3.8234e-09	TGCATGCM<br>TGCATG--	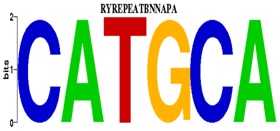
ABI3	1.6412e-08	KGCATGCA---<br>-GCATGCATNN	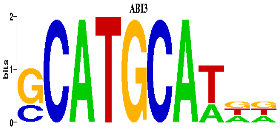
SPHCOREZMC1	1.0650e-07	KGCATGCA-<br>TCCATGCAT	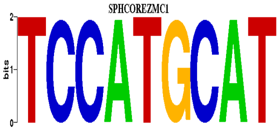

FUS3	1.2951e-07	---TGCATGCM<br>NNATGCATGS-	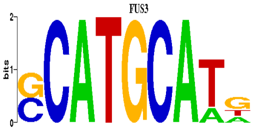

Motif8

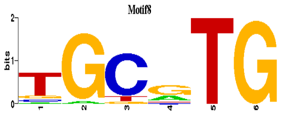

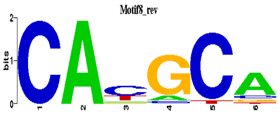

forward *reverse compliment*

Name	*E value*	*Alignment*	*Motif*
CACGCAATGMGH3	9.9774e-09	CAYGCA--<br>CACGCAAT	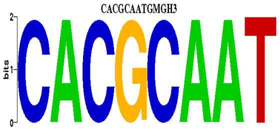
Hmx3_M00433	3.7717e-08	CAYGCA---<br>CACGCACTT	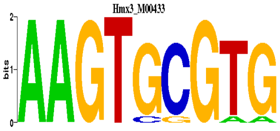
ANAC019_oneSite	6.4481e-08	---CAYGCA-<br>TAACACGCAT	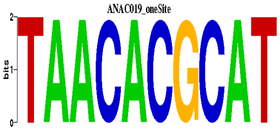
ANAC055_oneSite	6.4481e-08	---CAYGCA-<br>TAACACGCAT	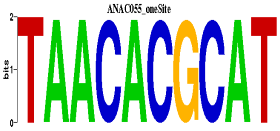
ANAC072_oneSite	6.4481e-08	---CAYGCA-<br>TAACACGCAT	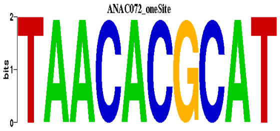

Motif9

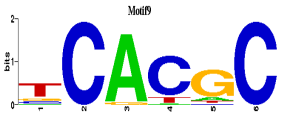

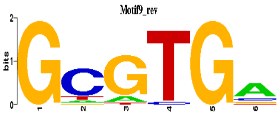

forward *reverse compliment*

Name	*E value*	*Alignment*	*Motif*

OCETYPEIINTHISTONE	5.9029e-08	-----GCGTGA<br>GATCCGCGTGA	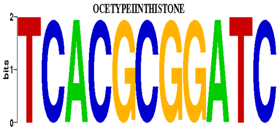
Pax6	3.8495e-07	--------GCGTGA-<br>MANTSAWGCGTGAA	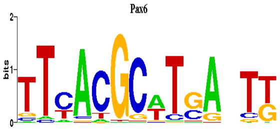
AhR_M00139	4.2072e-07	--TCACGC---------<br>TSTCACGCNASNYNGRG	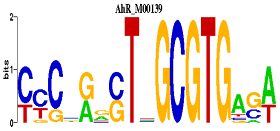
Pax-8_M00717	7.6380e-07	--TCACGC------<br>NNTCAYNCNNNNNN	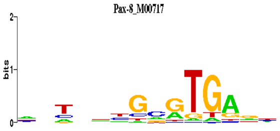
Pax-3_M00360	8.6917e-07	----GCGTGA--<br>KNAAGYGTGACG	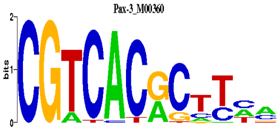

Motif6

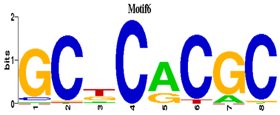

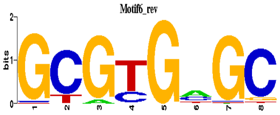

forward *reverse compliment*

Name	*E value*	*Alignment*	*Motif*
Pax-4_M00373	5.5530e-07	----GCNCACGC--------<br>NNNNSNNCACGCNTGAMNNC	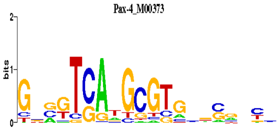
Nrf-1_M00652	7.6273e-07	-GCNCACGC<br>YGCGCAYGC	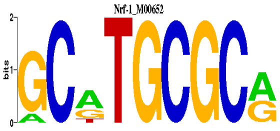
AhR_M00139	7.2492e-06	---------GCGTGNGC<br>CYCNRNSTNGCGTGASA	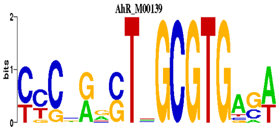
Egr-3_M00245	1.3264e-05	--GCNCACGC-<br>ACGCCCACGCA	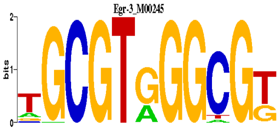

Egr-1_M00243	1.6826e-05	--GCNCACGC-<br>MCGCCCACGCA	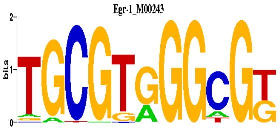

Motif10

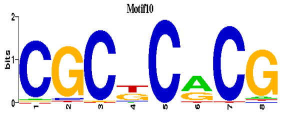

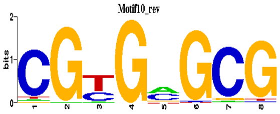

forward *reverse compliment*

Name	*E value*	*Alignment*	*Motif*
Egr-3_M00245	6.9707e-06	-CGCKCRCG--<br>ACGCCCACGCA	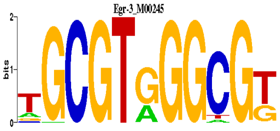
Nrf-1_M00652	8.8016e-06	CGCKCRCG-<br>YGCGCAYGC	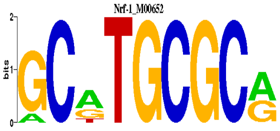
Egr-1_M00243	1.0260e-05	-CGCKCRCG--<br>MCGCCCACGCA	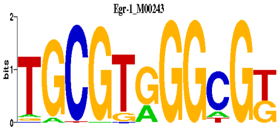
Egr-2_M00246	1.1590e-05	-CGCKCRCG--<br>MCGCCCACGCA	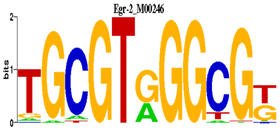
NGFI-C_M00244	2.0442e-05	-CGCKCRCG--<br>CCRCCCACGCA	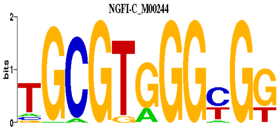

Motif2

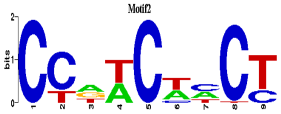

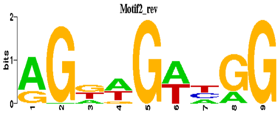

forward *reverse compliment*

Name	*E value*	*Alignment*	*Motif*

TCA1MOTIF	2.9665e-04	-AGNWGWNGG<br>AAGAAGATGA	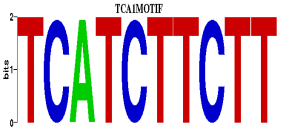
Aef1	4.0265e-04	AGNWGWNGG<br>TGTTGTTG-	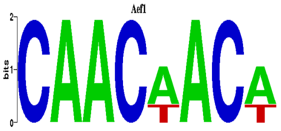
LUN-1_M00480	8.9809e-04	-CCNWCWNCT------<br>CCCAGCTACTTTGGGA	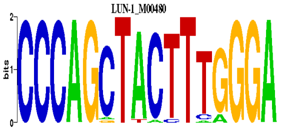
HOXA5_M00023	1.9190e-03	-------------------AGNWGWNGG-<br>NGRNNNNNNNNNANTAATGRNGNNNNNNGC	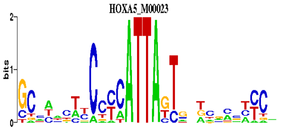
CAREOSREP1	2.0068e-03	CCNWCWNCT<br>-CAACTC--	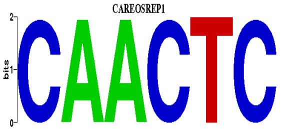

Motif5

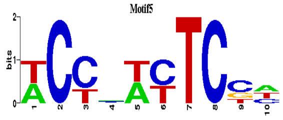

forward

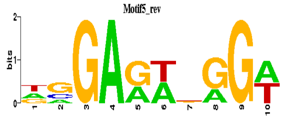

reverse compliment

Name	*E value*	*Alignment*	*Motif*
CAREOSREP1	8.9509e-05	WCCNWYTCNN<br>--CAACTC--	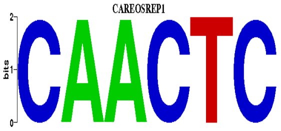
Pax-4_M00380	1.1169e-04	----NNGARWNGGW---------------<br>NGNGTGNNNNNNNNNNNNNNNNNTAWTTTN	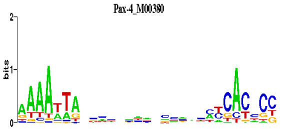
MRNA3ENDTAH3	3.0604e-04	WCCNWYTCNN--<br>--CATTTCCATT	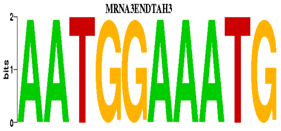
Nanog_M01123	3.1582e-04	NNGARWNGGW--<br>-GGMAATGGNCC	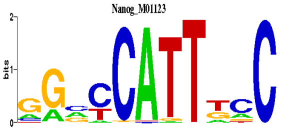

ACIIPVPAL2	4.3316e-04	NNGARWNGGW--<br>GGGGGTTGGTGG	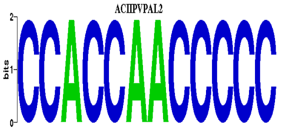

Motif11

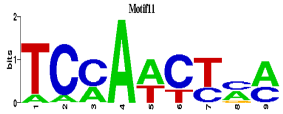

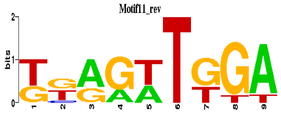

forward *reverse compliment*

Name	*E value*	*Alignment*	*Motif*
RBENTGA3	2.4345e-05	--TKAGWTGGA<br>TCCAAGTTGGA	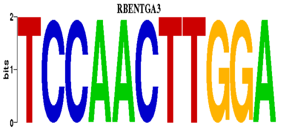
CAREOSREP1	6.9905e-05	TCCAWCTMA<br>--CAACTC-	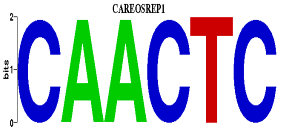
L4DCPAL1	7.5343e-05	----TCCAWCTMA<br>AATCTCCAACCA-	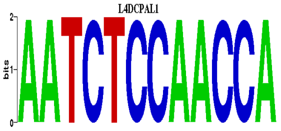
Nkx6-1_M00424	1.3189e-04	-TCCAWCTMA--<br>AANCAATTAAAW	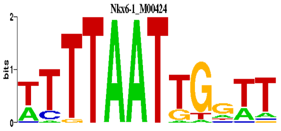
ELRE1PCPAL1	3.1854e-04	-------TKAGWTGGA-<br>GAAGGGGTTTGTTGGAG	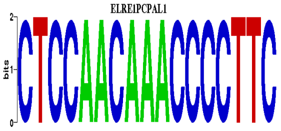

Sequence logo generation powered by weblogo
STAMP is written by Shaun Mahony
